# Age-related changes in the transcriptome of antibody-secreting cells

**DOI:** 10.18632/oncotarget.7958

**Published:** 2016-03-07

**Authors:** Senthil Kannan, Noor Dawany, Raj Kurupati, Louise C. Showe, Hildegund C.J. Ertl

**Affiliations:** ^1^ Gene Therapy and Vaccines Program, University of Pennsylvania School of Medicine, Philadelphia, Pennsylvania, USA; ^2^ The Wistar Institute Vaccine Center, Philadelphia, Pennsylvania, USA; ^3^ Department of Biomedical and Health Informatics, The Children's Hospital of Philadelphia, Philadelphia, Pennsylvania, USA

**Keywords:** ASCs, B cells, cell metabolism, programmed cell death, oxidative phosphorylation, Gerotarget

## Abstract

We analyzed age-related defects in B cell populations from young and aged mice. Microarray analysis of bone marrow resident antibody secreting cells (ASCs) showed significant changes upon aging, affecting multiple genes, pathways and functions including those that play a role in immune regulation, humoral immune responses, chromatin structure and assembly, cell metabolism and the endoplasmic reticulum (ER) stress response. Further analysis showed upon aging defects in energy production through glucose catabolism with reduced oxidative phosphorylation. In addition aged B cells had increased levels of reactive oxygen-species (ROS), which was linked to enhanced expression of the co-inhibitor programmed cell death (PD)-1.

## INTRODUCTION

Aging is characterized by a gradual deterioration of biological processes. The genome becomes unstable and accumulates mutations due to exogenous insults as well as endogenous sources of DNA damage such as increased production of reactive oxygen species (ROS), replication errors and declines in DNA repair mechanisms [1, 2]. In the same token mitochondrial DNA increasingly becomes damaged [3, 4] and energy production through the respiratory chain deteriorates [5]. As a consequence the aged become increasingly vulnerable to environmental challenges such as infections [6, 7]. Due to the numerous defects, functions of the immune system become impaired [8-10] during aging, leaving the elderly more susceptible to infection and less responsive to vaccines. The output of naïve cells of the adaptive immune system gradually declines [11]. T and B cell repertoires become more restricted [12-13] and CD4^+^ T cells loose the ability to provide appropriate help for differentiation of B cells into ASCs [14]. This together with intrinsic B cell defects reduces antibody responses in the aged [15].

Here we further elucidated in mice the age-related defects in B cell populations. Whole genome expression arrays comparing bone marrow-derived ASCs from young and aged mice showed disparity of expression in over 1500 genes and distinct clustering. Genes that were differentially expressed participate in numerous pathways and functions including immunological and metabolic functions. Functional analyses showed severe reductions in oxidative phosphorylation (OXPHOS), which was linked to increased levels of transcripts for lactose dehydrogenase (Ldh-a) with concomitant reduction in pyruvate dehydrogenase (Pdhx) suggestive of diminished use of glucose for energy production through the tricarboxylic acid (TCA) cycle. This was combined with increased expression of PD-1 and levels of cellular (c)ROS.

## RESULTS

### ASCs and their precursor populations are decreased in aged mice

**Figure 1:**
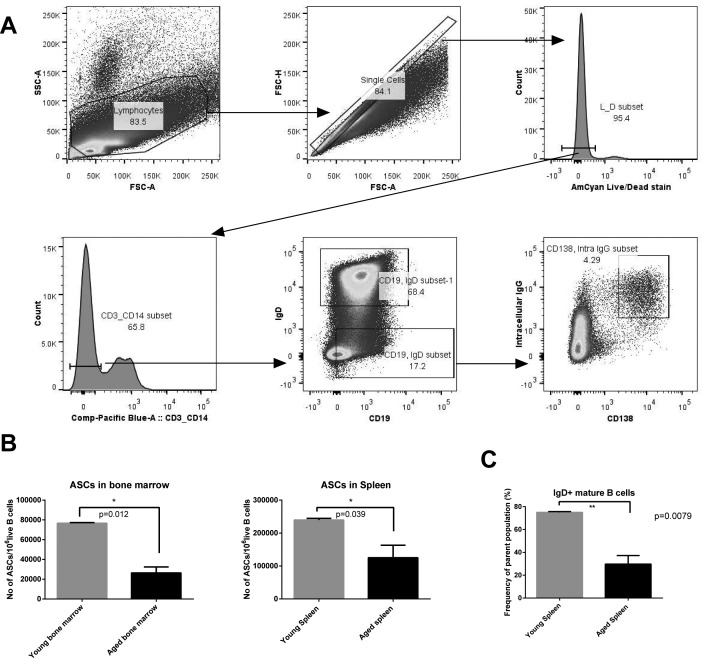
Gating strategy and numbers of ASC and mature B cells **A.** shows the gating strategy used to identify ASCs (Antibody Secreting Cells) - CD3^−^ CD14^−^ Live IgD^−^ CD19^+^ CD138^+^ Intracellular IgG^+^. **B.** shows numbers of ASCs in the spleen and bone marrow of young and aged mice. **C.** shows frequencies of mature naïve B cells. Aged mice are shown in black columns and young mice in grey columns. * denotes significance by *t* tests (ASCs in bone marrow: *p* = 0.0012; ASCs in spleen: *p* = 0.03; Frequencies of mature B cells: *p* = 0.0079.

We measured the number of ASCs in the spleens and bone marrow of young and aged mice using multicolor flow cytometry (Figure 1A). ASCs were significantly reduced in the aged mice in both the bone marrow and the spleen - the two primary reservoirs for ASCs (Figure 1B). The reduction was especially pronounced in the bone marrow, suggesting either reduced generation of long-lived ASCs or a decline in their life span.

In order to see if this discrepancy in ASC numbers was a remnant of a difference in their precursor population, we enumerated percentages of mature naive B cells within the entire B cell population. We noticed significantly lower percentages of mature B cells in the aged mice (Figure 1C). This corroborates previously observed data showing that bone marrow output of naïve B cells is decreased upon aging [11].

### Microarray analysis of ASCs shows multiple differences in transcriptome

**Figure 2:**
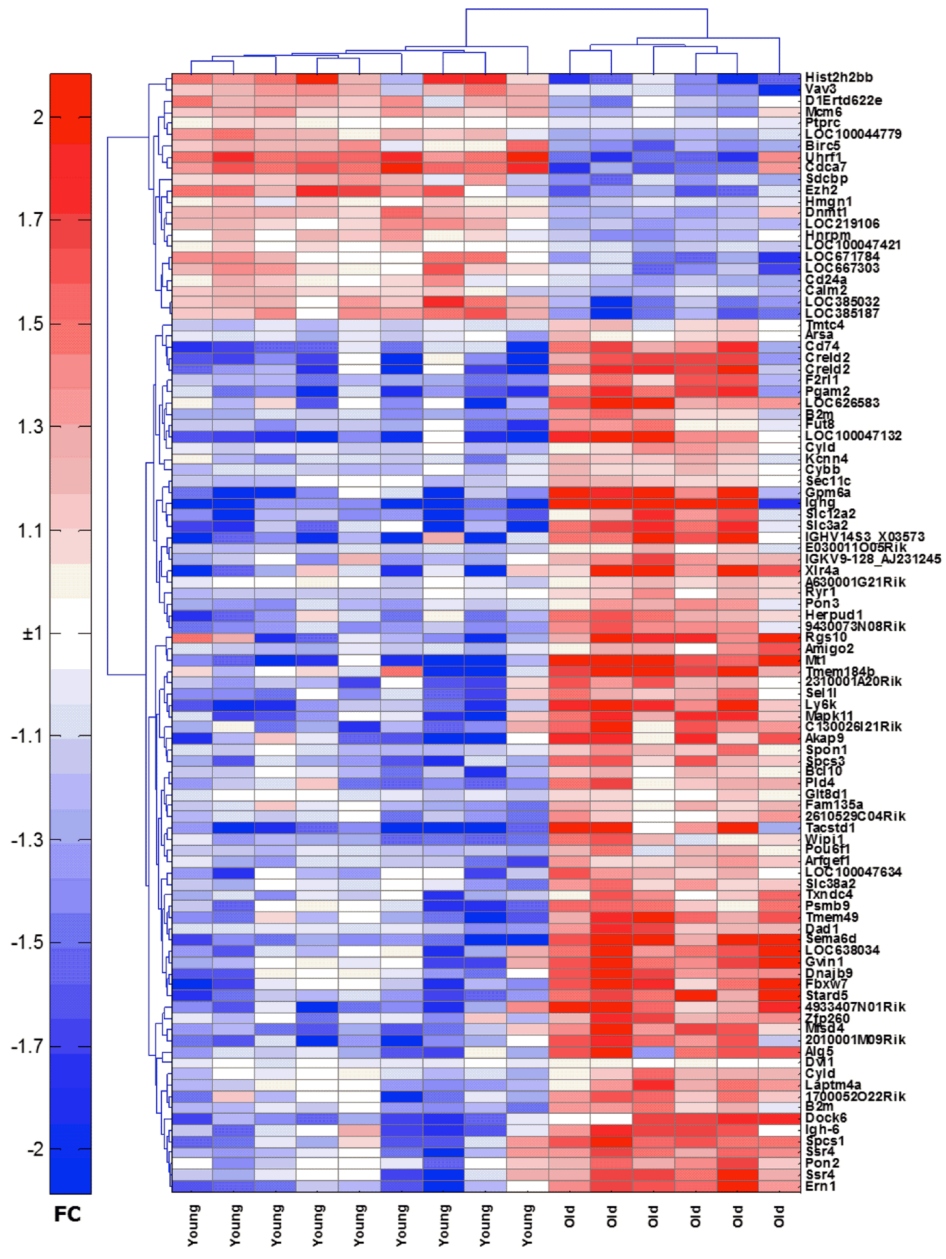
Heatmap of the top 100 genes that are differentially expressed between young and aged ASCs Transcript names are shown along the right axis. Red: increased expression, blue: decreased expression.

To elucidate age-related changes in ASCs, we isolated bone marrow resident ASCs from 9 young and 10 aged mice by cell sorting and profiled whole genome cDNA expression using Illumina Bead Arrays. We tested cells from individual mice or if cell numbers were low from pooled samples as indicated. The aim of these experiments was to compare cells at the gene expression level and identify differently expressed genes, pathways and functions. A total of 2175 probes were differentially expressed in ASCs from young mice compared to aged mice (Suppl. Table 1), at a p-value of 0.05. Of these probes, 1411 (65%) were more highly expressed in the aged mice while 764 (35%) had higher expression in the young mice. Principle component analysis [16] using 715 (*p* < 0.01) gene probes showed that young and aged ASCs clustered as 2 well separated groups (Suppl. Figure 1). A heatmap of the expression of the 100 genes that were the most significantly differentially expressed (by p-value) between the two groups is shown in Figure 2. Using Ingenuity Pathway Analysis, a number of functions distinguished old from young mice (Suppl. Table 2). The most significant differences were observed for cell death and survival, cellular growth and proliferation, hematological system development and function, tissue morphology, cellular development, humoral immune response and protein synthesis.

The aged mice had decreased expression of transcripts of histone clusters 1-3 (26/28) suggesting age-related differences in chromatin structure and transcriptional control, as has been reported [17]. This was further confirmed by the enrichment of the nucleosome assembly and chromatin organization gene ontology biological processes, using DAVID [18] (Suppl. Figure 2).

**Figure 3:**
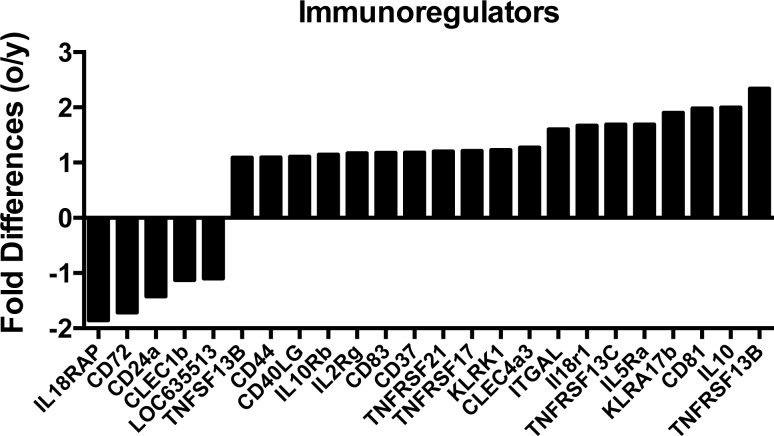
Differences in the expression of transcripts that encode immunoregulators between young and aged ASCs Positive numbers show higher expression in the aged ASCs, negative numbers show high expression in young ASCs.

**Figure 4:**
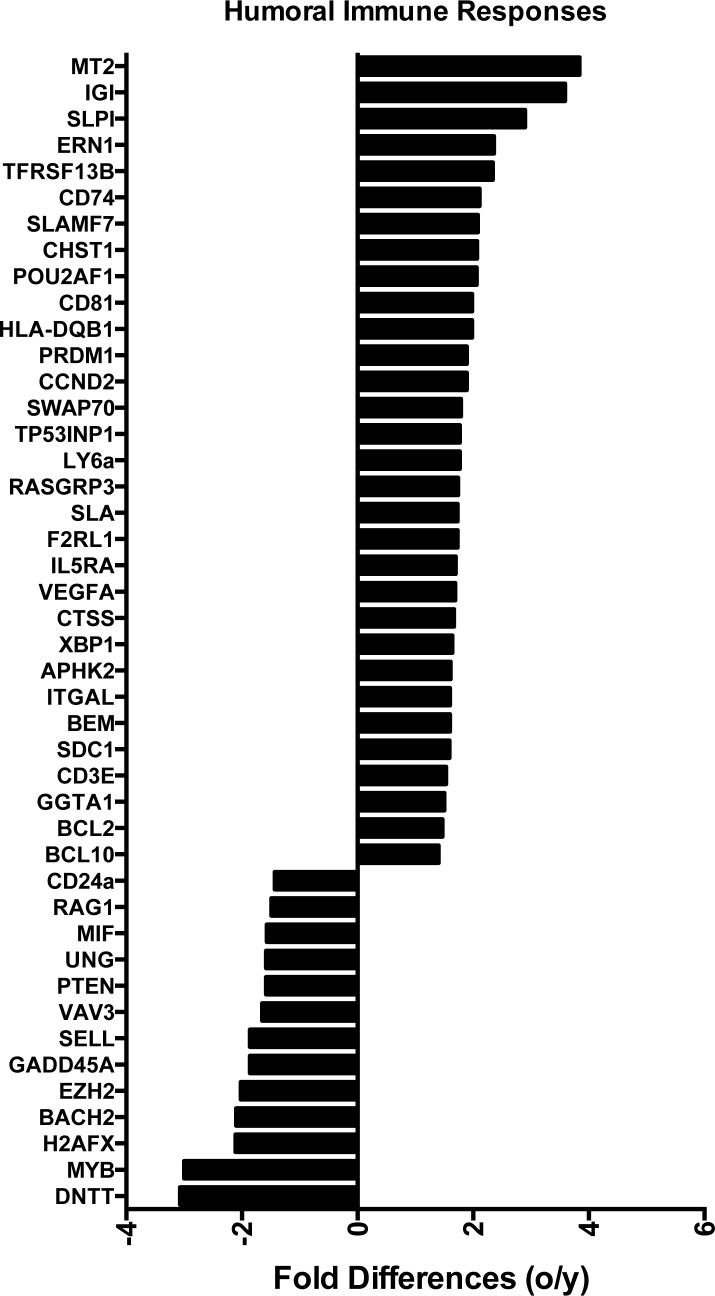
Differences in the expression of genes involved in the humoral immune responses biological function identified using Ingenuity Positive numbers show higher expression in the aged ASCs, negative numbers show higher expression in young ASCs.

Transcripts for a number of immunoregulators including CD markers, interleukin receptors, natural killer cell receptors and members of the TNF family were differentially expressed in aged and young ASCs (Figure 3). Most were higher in the aged (19/24). Several are known to affect B cell fate decisions. BACH2, which is essential for class switching [19], was expressed higher in young ASCs. XBP1, which becomes crucial at late stages of plasma cell development [20], was higher in aged ASCs. IRF4 and IRF8 were also differentially expressed; the former was overexpressed and the latter underexpressed in aged ASCs. Both play critical non-redundant roles in plasma cell development and germinal center formation. IRF8 induces expression of Bcl6 [21]. IRF4 down-regulates Bcl6 and instead induces Blimp-1 [22] encoded by the PRDM1 gene. Transcripts for PRDM1 were increased in aged ASCs. While Bcl6 promotes germinal center formation and proliferation of B cells Blimp-1 drives terminal differentiation of plasma cells [23]. These data support reduced class-switching in aged B cells and more terminal differentiation of aged plasma cells. Enrichment of genes involved in the humoral immune responses (Figure 4) were identified by Ingenuity Pathway Analysis (IPA) [24], and again most of the involved genes were higher expressed in aged ASCs.

**Figure 5:**
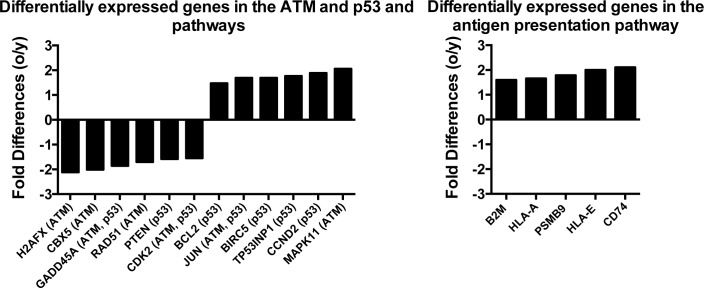
Differences in the expression of genes in the three most significantly altered canonical pathways identified using Ingenuity Positive numbers show higher expression in the aged ASCs, negative numbers show high expression in young ASCs.

IPA showed significant (*p* ≤ 0.01) differences in ATM and p53 signaling and antigen presentation (Figure 5). Most of the differentially expressed transcript encoding proteins involved in ATM signaling, which is activated by double-stranded DNA breaks, were higher in young mice (5/7) while transcripts for proteins of the p53 pathway were more commonly high in aged cells (5/8). Transcripts for antigen presentation pathways, such as those encoding histocompatibility antigens, were higher in aged ASCs.

**Figure 6:**
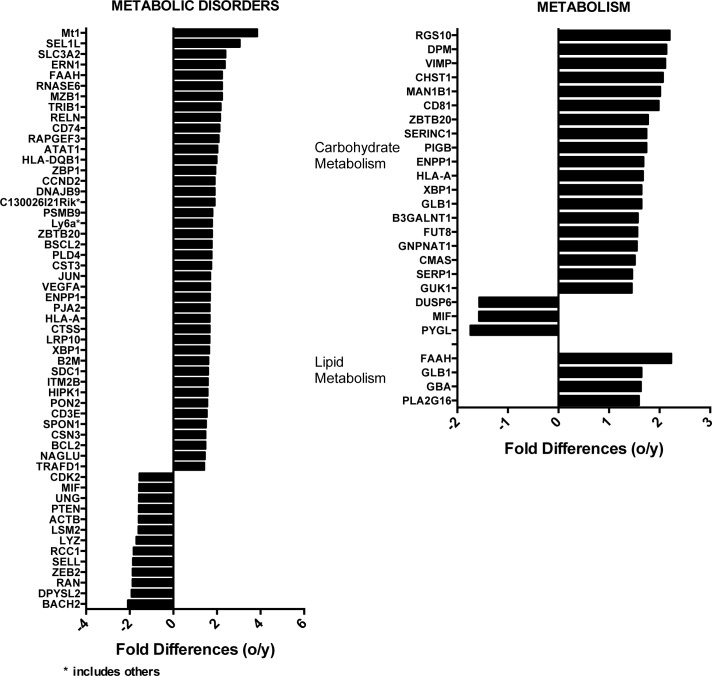
Differences in the expression of genes involved in metabolic disorders (on the left) or lipid and carbohydrate metabolism (right) identified using Ingenuity Positive numbers show higher expression in the aged ASCs, negative numbers show high expression in young ASCs.

Otherwise noteworthy were differences in metabolic diseases, carbohydrate and lipid metabolism (Figure 6). Most transcripts for metabolic disorders (42/55), carbohydrate (19/22) and lipid (4/4) metabolisms were expressed at higher levels in aged as compared to young ASCs. Several other genes involved in lipid and carbohydrate metabolism were also differentially expressed between young and aged ASCs at a more lenient p-value threshold of 0.05 and without fold change restrictions (Suppl. Table 1). These include ACLS1 (−1.25), involved in lipid biosynthesis, which was higher in younger ASCs. ACADS (1.39), ACADVL (1.35), ACSM2 (1.15), LONP2 (1.2) and ACSL4 (1.18), which participate in fatty acid degradation, were higher in aged ASCs as was OXCT1 (1.37), which catabolizes ketone bodies [25], a byproduct of FAO when carbohydrates are limited and STARD5 [26] (2.41) involved in intracellular fatty acid transport. Two enzymes of the tricarboxylic acid (TCA) cycle, i.e., ACO2 (1.49) and IDH (1.3) were also more highly expressed in aged ASCs.

A number of genes encoding mitochondrial proteins were differentially expressed such as those encoding components of the respiratory chain (Suppl. Figure 3), and mitochondrial fission (FIS1), which affects mitochondrial morphology [27]. Again, these genes were more highly expressed in aged ASCs. One mitochondrial ribosomal protein [MRLP33 (−1.59)] and one key ATP synthase [ATP5j (−1.48)] were underexpressed in aged ASCs.

Differences were also seen in genes controlling functions of peroxisomes, which are involved in fatty acid metabolism and reduction of ROS. These included VCP, (1.61) involved in assembly of peroxisome [28], PPARβ/δ (1.24), a regulator of energy metabolism [29] and ACBD5 (1.27), a peroxisome receptor for acyl-CoA-esters [30].

Functional analysis by DAVID showed significant differences in expression levels for the endoplasmic reticulum with most genes having higher expression in the aged (51/52). At a lower significance threshold (genes with *p* < 0.05), transcripts indicative of ER stress were increased in the aged (5/5) while those involved in protein folding were in part higher in the younger (4/10 Suppl. Figure 4).

We selected a number of immunoregulators that were differentially expressed in the microarray, and measured their protein levels by flow cytometry. Flow cytometry analysis confirmed differences that were observed at the transcriptome level. Mainly, CD52 and CD98 were significantly higher on the aged ASCs (Suppl. Figure 5), and CD72 was significantly lower on aged ASCs (data not shown).

### Aged B cells upon activation exhibit defects in mitochondrial respiration

**Figure 7:**
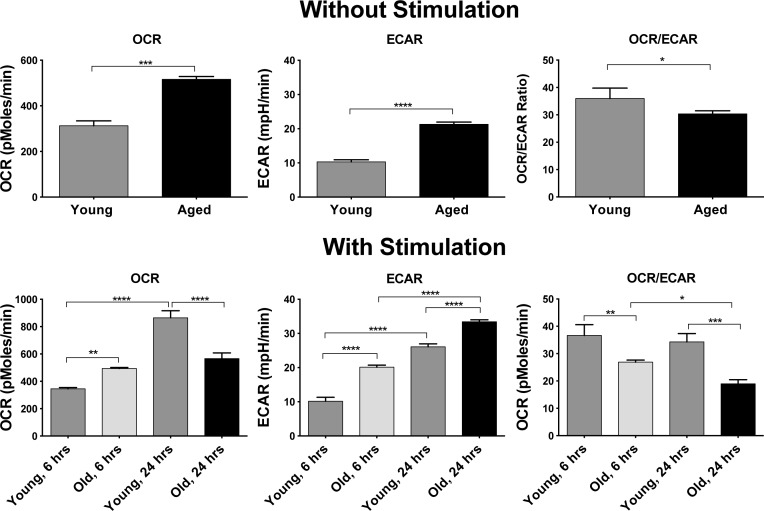
OCR and ERAR as well as ratios of OCR/ECAR are shown upon measuring splenic B cells without stimulation (top panels) or after stimulation for 6 or 24 hours (lower panels) Lines indicate significant differences between groups calculated by *t*-tests or 1-way Anova. *p values between 0.01 - 0.05, **p-values between 0.001 - 0.01, *** *p*-values between 0.0001 - 0.001, **** *p* - values < 0.0001.

The arrays indicated marked differences in carbohydrate and lipid metabolism as well as in the electron transfer chain between ASCs from young and aged mice. To further assess metabolic differences we tested bead purified B cells from young and aged mice for basal respiration and lactate production by Seahorse XF flux analyzer [31] (Figure 7). Without stimulation aged B cells had higher oxygen consumption rates (OCR), which mainly measures oxidative phosphorylation (OXPHOS) through the electron transfer chain. In addition, there was higher extracellular acidification rates (ECAR), as a measure of lactate production by glycolysis, in B cells of aged mice compared to young B cells, suggestive of higher metabolic activity of aged compared to younger B cells. The OCR to ECAR ratio was slightly higher in younger B cells indicating a higher contribution of OXPHOS to their overall energy production. B cells were then stimulated with a cocktail of mitogens and tested 6 or 24 hours later. After 6 hours OCR and ECAR remained comparable to those of unstimulated young and old B cells. After 24 hours OCR markedly increased in younger but not aged B cells, while ECAR increased in both. These data indicate that upon aging, OXPHOS cannot satisfy increases in energy demands under challenging conditions.

### Glucose metabolism changes upon aging in B cells

**Figure 8:**
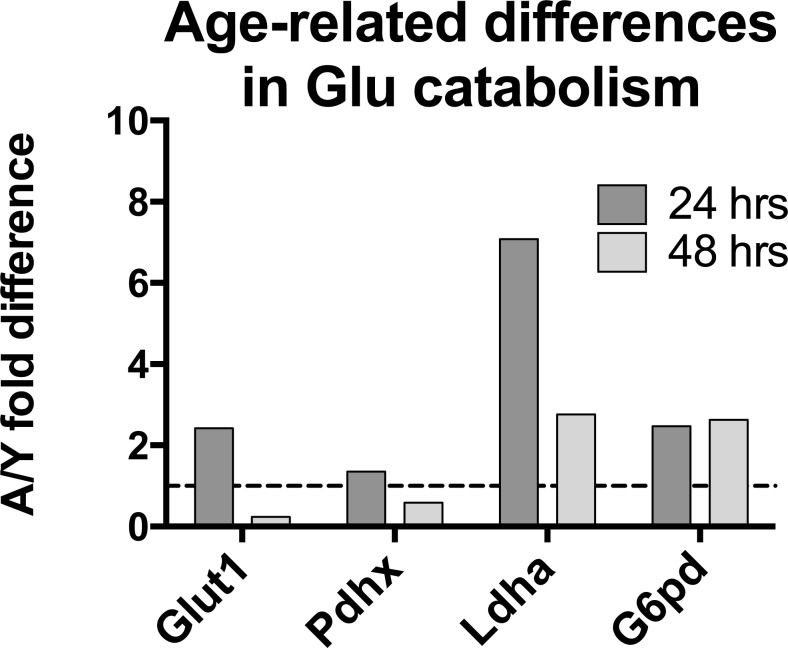
Levels of transcripts of cells stimulated for 24 or 48 hours were normalized to GAPDH They were then within each age-group normalized to levels at baseline. The graph shows differences in fold changes over baseline comparing aged to young B cells. Results are based on 3 mice per group.

We conducted additional studies by comparative reverse transcription (c)PCR probing levels of transcripts for factors involved in glucose metabolism at baseline and after 24 or 48 hours of culture with mitogens comparing stimulation-induced changes of aged to young mouse B cells. Transcripts for Glut1, the main receptor that is used by B cells for glucose uptake, increased after 24 hours more pronounced in old than young B cells; this was reversed after 48 hours, indicating that aged B cells could not maintain their demands on energy through increased consumption of glucose. Transcripts for Pdhx, a component of the pyruvate dehydrogenase complex, which converts pyruvate into acetyl-CoA that is then used in the Krebs cycle, were at 24 hours similar to those at baseline and showed at that time no difference between the two age-groups. By 48 hours Pdhx transcripts were markedly lower in aged B suggesting that the aged may lack glucose-derived substrate to fuel OXPHOS. Increases in transcripts for Ldha which converts pyruvate into lactate, were higher in aged B cells at both time points. Transcripts for glucose-6-phosphate dehydrogenase (G6pd), which shunts the glycolysis metabolite glucose-6-phosphate into the anabolic pentose phosphate pathway, increased in both cohorts after stimulation and increases were more pronounced in the aged (Figure 8). These data suggest that upon aging glucose catabolism becomes impaired.

### PD-1 expression is higher on aged ASCs

**Figure 9:**
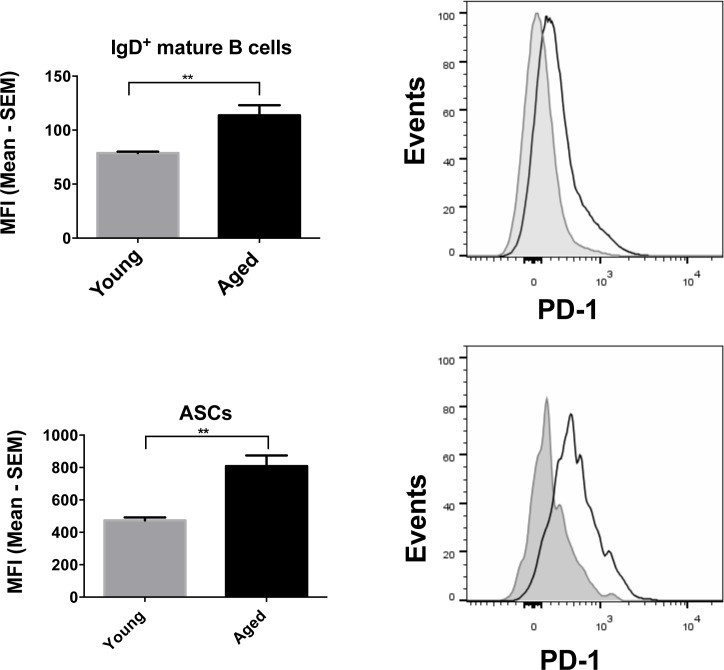
PD-1 expression on mature B cells and ASCs Graphs on the left show the MFI of PD-1 expression, with error bars representing SEM. Aged samples are shown in black and the young, in grey. Representative histograms are shown alongside with the aged samples in a solid black line and the young samples in grey. * denotes significant differences as determined by Mann-Whitney test. (Mature B cells: *p* = 0.0079; ASCs: *p* = 0.0079).

To assess if energy production through glucose might be affected by cell surface markers we probed young and aged B cell populations for expression of PD-1 an immunoregulator that has been shown to have significant metabolic functions by inhibiting glycolysis in activating T cells [32]. To assess if PD-1 plays a role in regulating metabolism in ASCs, we measured its expression levels and observed that PD-1 levels were significantly higher on aged ASCs, a difference that was also observed on mature naïve B cells (Figure 9).

### Aged ASCs have high cellular ROS levels, which correlate with PD1 expression

**Figure 10:**
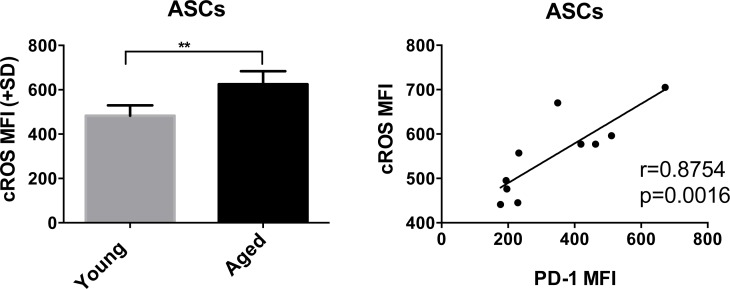
A shows cROS levels in young and aged ASCs Samples from aged mice are shown in black and from younger mice in grey. Graph on left shows MFI, with error bars indicating standard error of the mean. * denotes significant differences as determined by Mann-Whitney test. (*p* = 0.0079). B shows the positive correlation between cROS and cell surface expressed PD-1 using ASC samples from young and aged mice. Correlation was determined by Spearman. R and *p*-values are shown within the graphs.

Since PD-1 plays a role in cellular metabolism of T cells and is expressed higher on aged B cells, we tested splenic ASCs for metabolic parameters and found that they carry higher levels of cellular reactive oxygen species (cROS), indicative of increased oxidative stress compared to cells from young mice (Figure 10a). ROS is produced within cells by numerous pathways with major sources being the mitochondrial electron transport chain, peroxisomes and the endoplasmic reticulum (ER) [33-35]. Peroxisomes, which play a central role in lipid metabolism, contain a number of ROS producing and detoxifying enzymes. Again, genes that control peroxisome assembly and size were differentially expressed between ASCs of the two age groups. Two enzymes that participate in cytoplasmic, peroxisomal and mitochondrial ROS metabolism were higher in the aged. These were PRDX4 [36] (1.85), which reduces hydrogen peroxide to water and CCS [37] (1.25), a copper chaperone for superoxide dismutase (SOD), a key group of enzymes that destroy ROS in the cytoplasm, mitochondria and peroxisomes. During oxidative protein folding, ROS such as H_2_O_2_ is produced in the ER. Accumulation of misfolded proteins triggers an ER stress response [38] to reduce protein production, enhance production of chaperones that assist in protein folding, increase production of enzymes that ubiquinate and degrade misfolded proteins and initiate apoptosis. Levels of cellular ROS in ASCs correlated with PD-1 expression indicating a link between the immunoregulator and metabolism (Figure 10b).

## DISCUSSION

The main focus of our study was to further elucidate the effect of immunosenescence on ASCs, which are present at significantly reduced numbers in aged as compared to young mice. Previous studies showed impaired antibody responses in the aged [6, 39]. Upon antigenic stimulation, increases in the numbers of ASCs are less pronounced in elderly compared to young individuals, which may in part reflect impaired formation of germinal centers [40]. Class switching [41] and hypermutation of immunoglobulin genes [42] are also diminished resulting in the production of low affinity antibodies.

Microarray analysis offers an unprecedented tool to elucidate differences in gene expression, which then upon pathway analysis gives some insight into functional variations. Previous studies have used gene expression profiling to elucidate changes upon stimulation of B cells *in vitro* or *in vivo* [43-45].

We used microarrays to identify age-related changes in ASCs from bone marrow where most long-lived plasma cells reside. As expected, many genes involved in numerous pathways and functions were differentially expressed between young and aged ASCs. It is accepted that upon aging, cells accumulate DNA mutations, which may lead to structural changes of DNA [1]. Our array data show reduced ATM signaling in the aged. The ATM pathway is induced by DNA damage; it prevents cells from progressing through the cell cycle, increases DNA repair mechanisms or drives cells to undergo apoptosis [46]. ATM regulates apoptosis through p53, a pathway, which was also affected in aged ASCs. Reduced ATM signaling in turn could contribute to increased DNA damage. An analysis of differences in significant biological functions accordingly showed the most significant differences for cell survival and death and cell growth and proliferation. Although it is assumed that long-lived bone marrow derived plasma cells are terminally differentiated, and thereby incapable of proliferation, recent gene expression array analyses indicate that although bone marrow-derived ASCs are quiescent they are capable of undergoing some replicative cell renewal [47]. Reduced proliferation in the aged combined with increased rates of apoptosis could thus explain the marked reduction of numbers of ASCs especially of those residing in the bone marrow.

Immunological functions and pathways in ASCs are affected by immunosenesence. The ASCs' ability to serve as antigen-presenting cells declines due to reduced expression of MHC antigens and LMP2, a key proteasome needed for the generation of MHC class I binding peptides. Ingenuity analysis for significant functions revealed differences in humoral immune responses. Four of the 13 genes with lower expression in the aged play a role in DNA recombination or generation of antibody diversity confirming previous studies that upon aging antibody responses loose breadth [12].

Differential expression of transcripts between the two age groups was observed for the ER stress response. Previous studies showed that the ER stress response also referred to as the unfolded protein response increases in the aged due to loss of chaperone proteins [48]. This again increases apoptosis and may contribute to the reduced numbers of ASCs.

Metabolism becomes impaired upon aging in part due to increased resistance to insulin [49] and in part due to deleterious alterations of mitochondria caused, for example, by the disorganization of their structure [50]. This is evidenced in our array data by the increase in expression of FIS1, which regulates mitochondrial fission and thereby affects mitochondrial morphology. Upon aging, mutations accumulate in mitochondrial DNA^4^. ROS increases [51], which again was recapitulated in our studies. Mitochondria loose the ability to efficiently generate energy through OXPHOS [52]. Again our array analysis indicated marked differences in transcripts encoding enzymes of the TCA cycle, which fuels the electron transport chain and components directly involved in electron transport. A functional analysis by Seahorse, which was conducted with B cell populations as surrogates for ASCs since the paucity of such cells precludes their direct analysis for OCR or ECAR, showed that aged B cells without further activation appear to be metabolically more active. Upon stimulation, B cells, similar to T cells, increase glycolysis, which is relatively inefficient for generating energy but provides building blocks needed for cell division and effector function. Aged B cells, upon stimulation, increased glycolysis as evidenced by an increase in lactate production, and by Seahorse flux analysis increases over resting B cells were comparable to those observed in young B cells. An analysis of transcripts indicated that aged B cells convert more pyruvate to lactate rather than acetyl-CoA for entrance into the TCA cycle than young B cells, which was confirmed by differences in OCR. This parameter of metabolism only increased in young but not old B cells after mitogen-mediated stimulation, demonstrating severe defects in the aged cells ability to gain energy through OXPHOS. This only became obvious once cells had to increase energy production to allow for cell proliferation and effector function. OXPHOS can be fueled by numerous substrates including carbohydrates, amino acids and lipids, which generate NADH and FADH_2_ upon degradation in the TCA cycle. In turn, NADH and FADH_2_ are essential for the conversion of ADP to ATP, the energy currency of life, in the energy transport chain, which according to the array data was significantly altered upon aging.

Another interesting finding was increased PD-1 expression on aged as compared to younger B cell populations as has been observed on T cells [53]. On T cells PD-1 is a well-described activation/exhaustion marker that dampens downstream signaling upon receptor/co-receptor ligation by blocking PI3K/Akt activation. This in turn decreases glucose uptake and glycolysis [32, 54]. Less is known abut the role of PD-1 on B cells although available evidence suggest that it also serves as an immunoinhibitors on these cells [55]. We observed the former when upon stimulation aged B cells showed less sustained Glut1 expression but not the latter as the ratio of glycolysis over OXPHOS clearly increased in aged B cells. As described previously for T cells [56], PD-1 was linked to increased levels of ROS, which may reflect increased oxidization of fatty acids to compensated for lack of glucose derived substrate fueling OXPHOS.

In summary, bone marrow-derived ASCs, which are generated throughout a lifespan of an individual show substantial differences in their gene expression profiles in aged as compared to young mice. In addition aged B engaged distinct metabolic pathways to produce energy upon stimulation, both of which may explain their deteriorating functions.

## MATERIALS AND METHODS

### Mice

Mice were obtained from Charles River labs and housed in the Wistar Institute's animal facility. Young mice were 6-8 weeks of age, and aged mice were > 17 months of age. All procedures followed approved protocols.

### Sample collection

Mice were euthanized by CO_2_ asphyxiation, and samples were collected. Bone marrow samples were isolated from the bones of the hind legs of each mouse. Cells within the marrow were flushed out using a 27 ½ G syringe, and resuspended in prewarmed L-15 media (Life technologies, CA). Whole spleens and PBMCs were also collected. Spleen samples were ground up in L-15 media, and filtered through BD Falcon cell strainers. Cells from both samples were then counted using a haemocytometer, and resuspended in DMEM (Life technologies, CA) media containing 10% Fetal Bovine Serum, Antibiotics (Penicillin and Streptomycin).

### B cell analysis by flow cytometry

The following fluorochrome conjugated antibodies were used: CD3-Pacific Blue, CD14-Pacific Blue, AmCyan Aqua Blue Live/Dead stain, CD19, B220, IgD and CD138. All antibodies were obtained from BD Biosciences (San Jose, CA) unless otherwise specified.

Samples were stained for 30 minutes at room temperature and then washed with PBS. The stained samples were analyzed in a LSRII flow cytometer (BD Biosciences, San Jose, CA).

### Microarrays

Bone marrow resident cells were isolated as described above, and stained for extracellular markers. Cells were then sorted on a FACS ARIA fluorescent cell sorter for ASCs. Total RNA and small RNA were isolated from sorted ASCs using the modified protocol of Ambion RNAqueous -Micro kit (cat #AM1931) to recover the small RNAs. The amount of total RNA was measured by Nanodrop and run on Agilent RNA 6000 pico kit (cat # 5067-1513). Total RNA at 10ng was amplified with NuGEN Ovation PicoSL WTA system (cat # 3310-48) to generate amplified cDNA which was then labeled with Biotin (NuGEN Encore BiotinIL Module, cat # 4210-48).

Biotin labeled cDNA at 750ng obtained from ASCs of young and aged mice was hybridized to Illumina MouseWG-6 v2 whole genome BeadChips. All arrays were processed in the Wistar Institute Genomics Facility.

### Data processing and analysis

Arrays were quantile normalized and the data filtered to remove non-informative probes that were expressed at background levels in the majority of the samples or had a maximum fold change below 1.2 between any two samples. A total of 25,988 probes were retained for further analysis. Replicates available for 2 samples were averaged prior to statistical analysis. A *t*-test with a p-value cutoff of 0.05 was used to identify differentially expressed genes between the aged and young mice. All preprocessing and microarray data analysis were conducted in MATLAB 8.0.0.783.

Probes with a *p*-value < 0.01 (unless otherwise noted) and a minimum fold change of 1.4 were selected for functional analysis. Ingenuity Pathway Analysis (IPA) and DAVID were used to identify enriched pathways and biological functions at a p-value cutoff of 0.05 after multiple-test correction using the Benjamini procedure.

### Quantitative reverse-transcription PCR

Splenic pan B cells were isolated from three young (14-16 wk old) and three aged (> 18 months old) C57BL/6J mice using negative selection Pan B cell isolation kit II (Miltenyi Biotec, Auburn, CA) according to manufacturer's instructions. An aliquot of B cells was frozen in RLT buffer as baseline samples (0 hr stimulation) and rest of the cells were stimulated for 24 hr or 48 hr in RPMI medium with 10% FBS containing a cocktail of 100 ng/mL pokeweed mitogen extract (Sigma-Aldrich, Saint Louis, MO), 1/10,000 dilution of heat-killed, formalin fixed *Staphylococcus aureus* Cowan I cells/PANSORBIN Cells (EMD Millipore, Billercia, MA), 2 μg/ml of oligo CpG ODN1826 and 1 μg/ml of Anti-CD40 (BioXcell, West Lebanon, NH). RNA was isolated following stimulation using RNeasy mini Kit (Qiagen, Valencia, CA) and cDNA was synthesized using High-Capacity cDNA Reverse Transcription Kit (Applied Biosystems, Foster City, CA) according to the manufacturer's instructions. The qPCR reactions were performed in 20 ul volume containing the 2.5 pm/μl primers with 2x Fast SYBR^®^ Green Master Mix (Applied Biosystems, Foster City, CA) in a Optical 96-Well Fast Clear Reaction Plates (Applied Biosystems, Foster City, CA). All reactions were performed in duplicates and the relative quantification of gene expression was calculated by using a ΔCT method. The following primers were used: Glut1 (Solute Carrier Family 2 (Facilitated Glucose Transporter), Member 1): forward: TGTGGGAGGAGCAGTGCTCG, reverse: TGGGCTCTCCGTAGCGGTG; Ldha (L-lactate dehydrogenase A chain;Ldha;ortholog), forward: AGAGCCGGCTCAACCTGGTC, reverse: TGGGTTAAGAGACTTCAGGGAGAC; Pdhx (Pyruvate dehydrogenase protein X component): forward: TGGTTGAAGAAGGGGAAGATTGG, reverse: AATCTGCGGCTGTGGAGAGG; G6pdx (Glucose-6-phosphate 1-dehydrogenase X), forward: ATGCCCGCTCACGACTCACAG, reverse: AGGAATTACGGGCAAAGAACTCC.

## SUPPLEMENTARY MATERIAL FIGURES AND TABLES






