# Supplementary material for: Age-related changes in the transcriptome of antibody-secreting cells
Source: Oncotarget. 2016 Mar 7;7(12):13340–53. doi: 10.18632/oncotarget.7958 (PMC4924646; doi:10.18632/oncotarget.7958)
Supplement: Supplementary file 1 [file oncotarget-07-13340-s001.pdf]

Age-related changes in the transcriptome of antibody-secreting cells

Supplementary Material

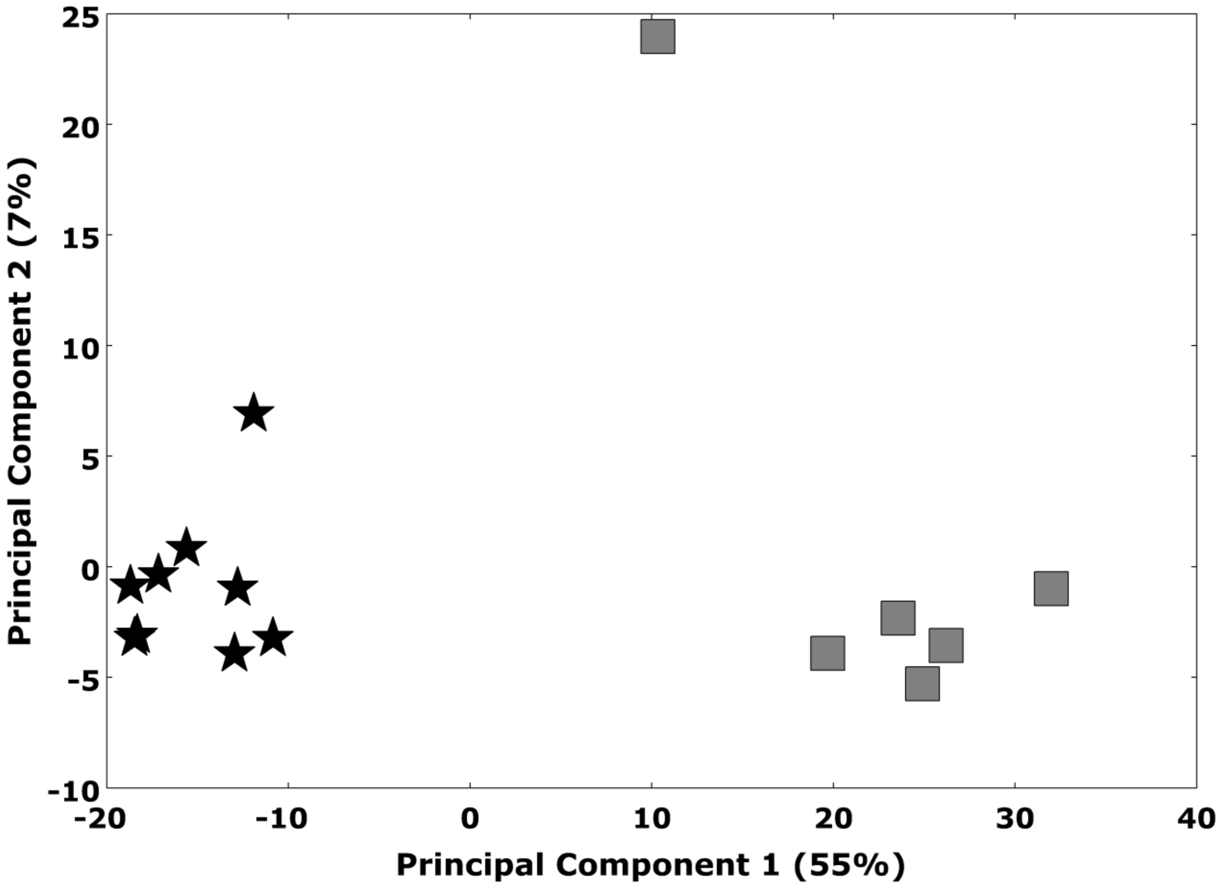

Suppl. Fig. 1 shows clustering of genes significant at a p-value of 0.01 along the first two principal components.

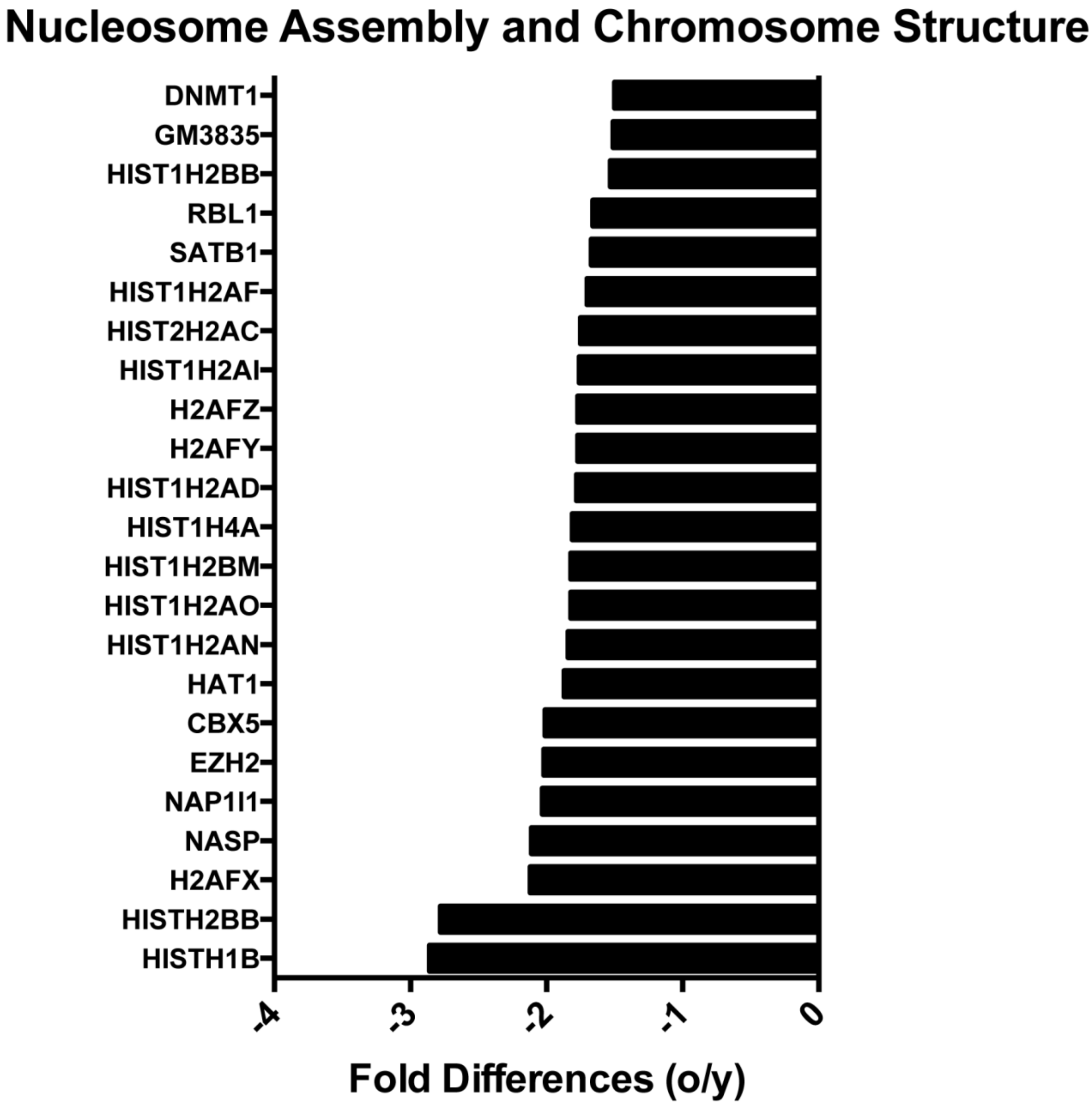

Suppl. Fig. 2 shows differences in the expression of genes involved in nucleosome assembly and chromosome structure using DAVID. Positive numbers show higher expression in the aged ASCs, negative numbers show higher expression in young ASCs.

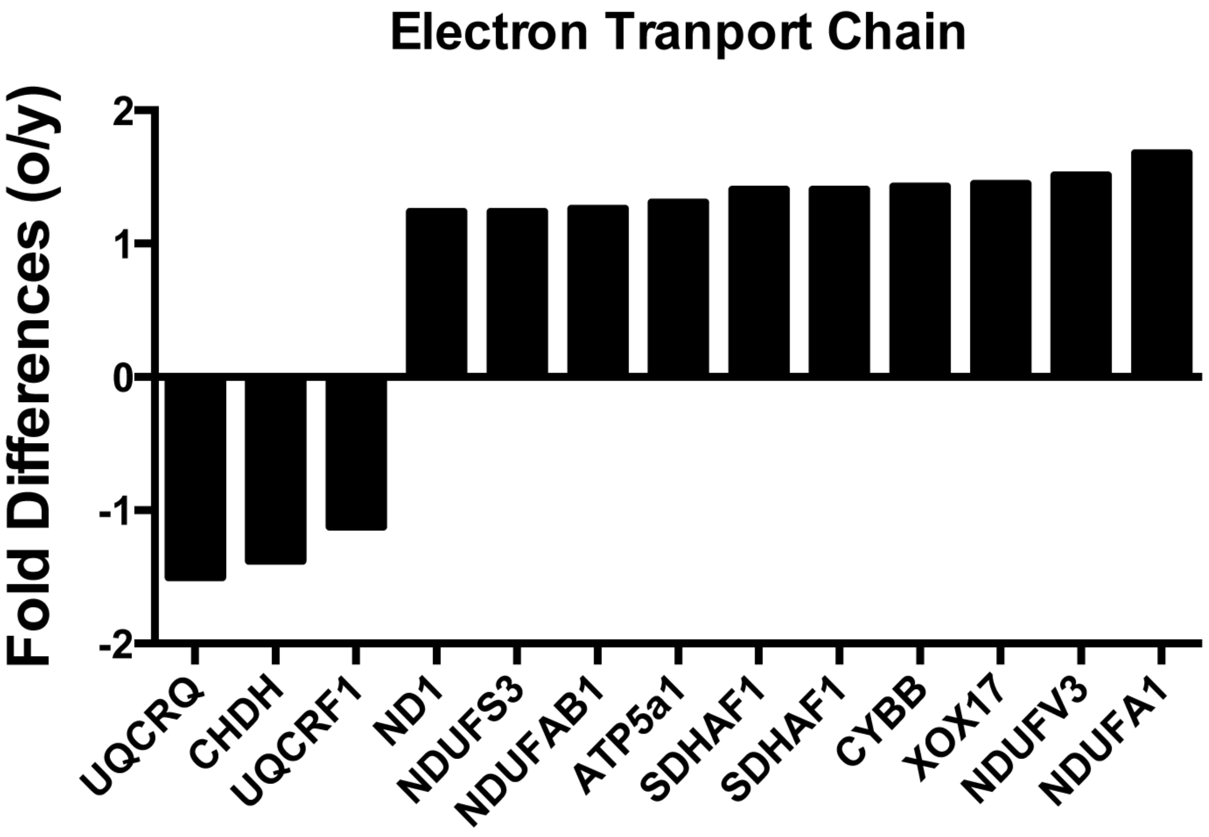

Suppl. Fig. 3 shows differences in the expression of genes involved in the electron transport chain. Positive numbers show higher expression in the aged ASCs, negative numbers show higher expression in young ASCs.

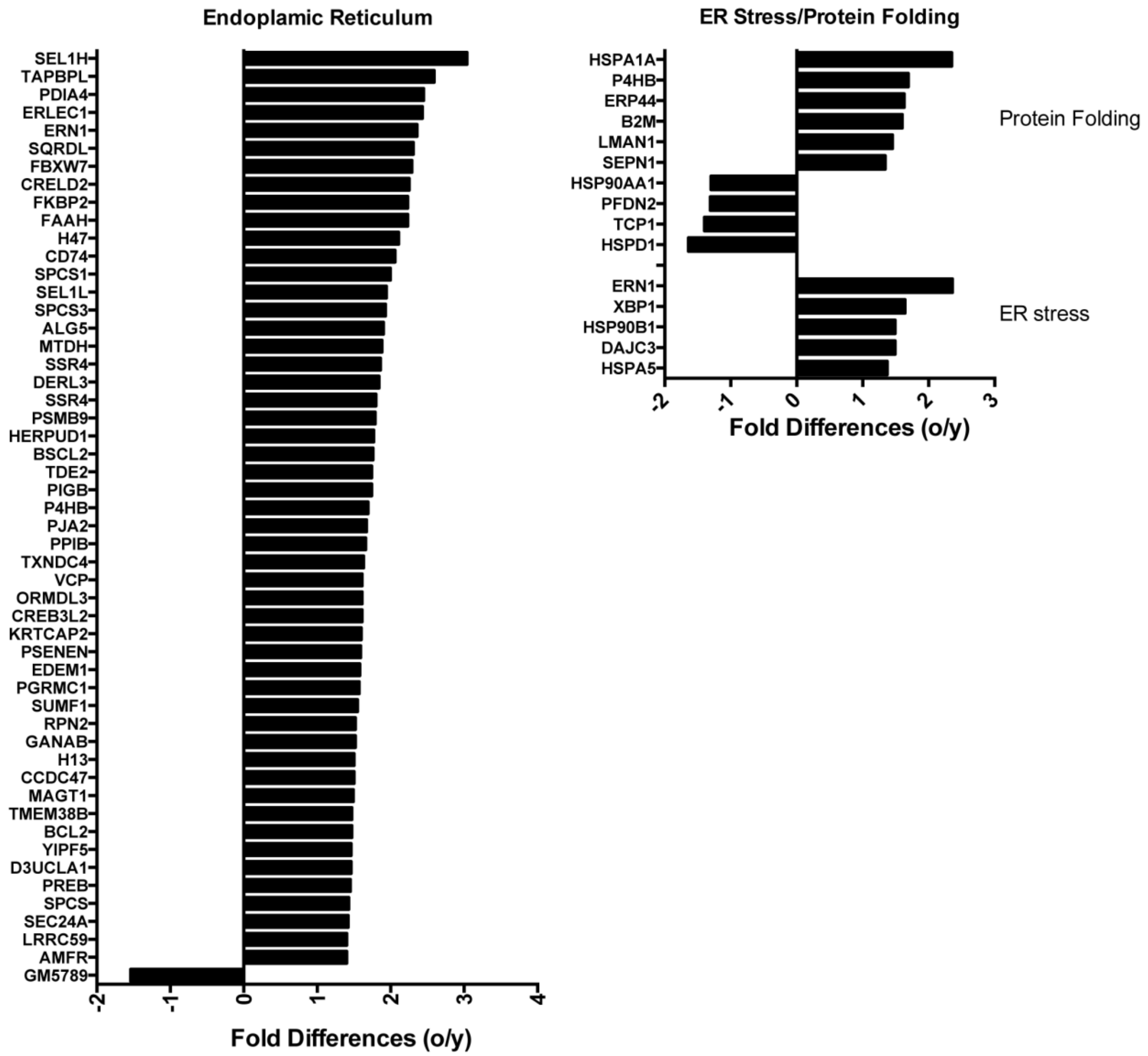

Suppl. Fig. 4 shows differences in the expression of genes associated with the endoplasmic reticulum (left) using DAVID. The bar graph on the right shows differential expression of transcripts encoding proteins involved in the ER stress response or protein folding. Positive numbers show higher expression in the aged ASCs, negative numbers show higher expression in young ASCs.

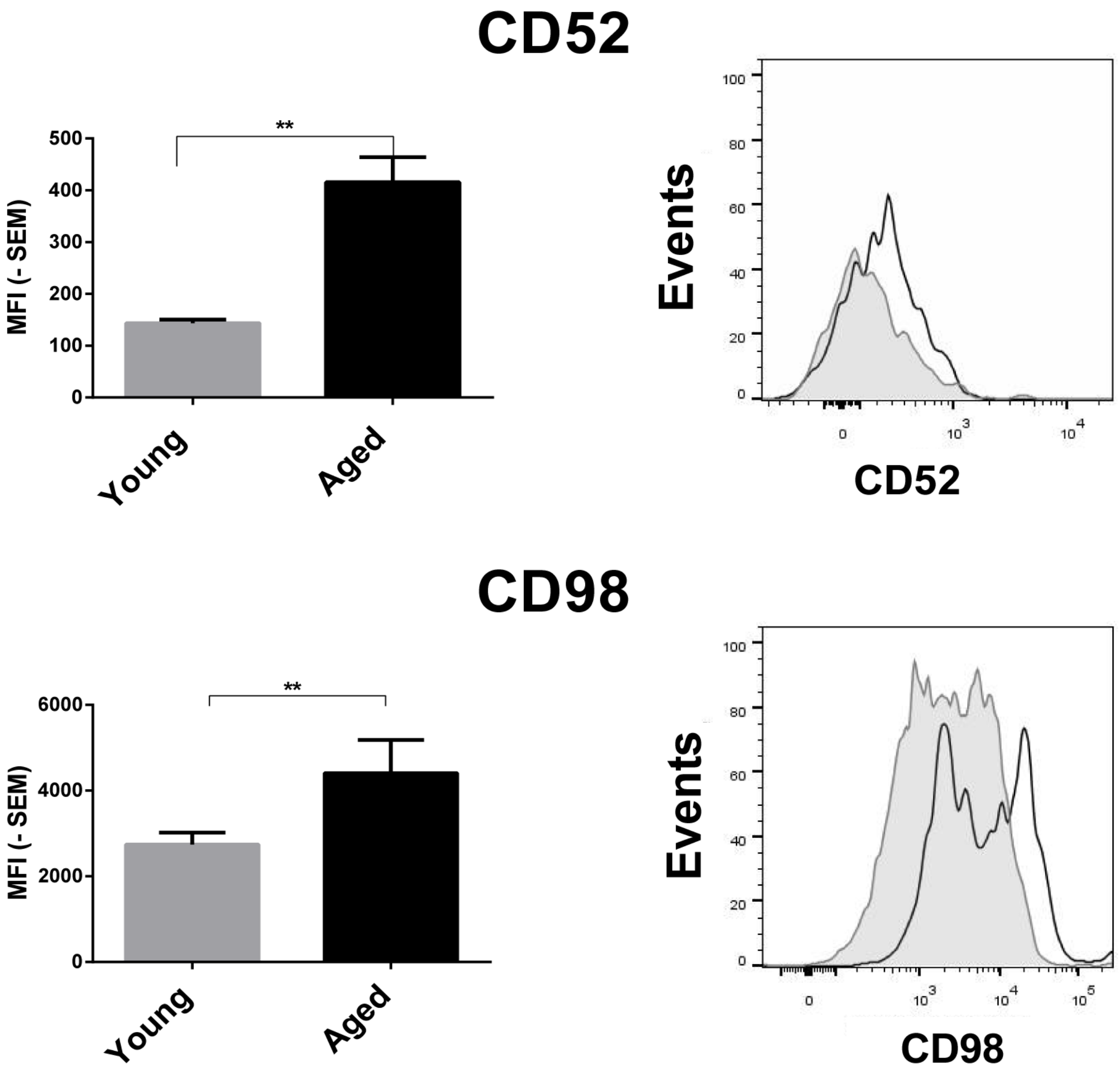

Suppl. Fig. 5. Expression levels of CD52 and CD98 on bone marrow derived ASCs from young and aged mice. Graphs on the left show Mean Fluorescent Intensity (MFI) of the specified molecule. Samples from aged mice are in black, and from young mice in grey. Error bars represent standard error of the mean (SEM). Representative histograms are shown on the right. Samples from aged mice are shown with a solid black line. Samples from young mice are shown in grey. * denotes significant differences as determined by Mann-Whitney test. (CD52: p=0.0079; CD98: p=0.005).
