# Supplementary material for: Age-related changes in the transcriptome of antibody-secreting cells
Source: Oncotarget. 2016 Mar 7;7(12):13340–53. doi: 10.18632/oncotarget.7958 (PMC4924646; doi:10.18632/oncotarget.7958)
Supplement: Supplementary file 2 [file oncotarget-07-13340-s002.pdf]

Supplemental Table 1

Illumina ID	Accession	Gene Name	Gene Symbol	Gene Name 2	Gene Symbol 2	P-Value	Ratio
ILMN_1250340	AK042059	NaN (S)	A630055A13Rik	nei like 3 (E. coli)	Neil3	8.14E-03	-3.349
ILMN_2436424	NaN	NaN (S)	Igl-5	n/a	n/a	1.47E-03	-3.078
ILMN_1232383	NM_001043228	deoxynucleotidyltransferase, terminal (Dntt), transcript variant 2, mRNA. (S)	Dntt	deoxynucleotidyltransferase, terminal	DNTT	3.92E-03	-3.068
ILMN_2683910	NM_010848	myeloblastosis oncogene (Myb), mRNA. (S)	Myb	myeloblastosis oncogene	myb	4.61E-03	-2.979
ILMN_2720813	NM_183428	erythrocyte protein band 4.1 (Epb4.1), mRNA. (S)	Epb4.1	erythrocyte protein band 4.1	Epb4.1	7.64E-03	-2.892
ILMN_2664593	NM_020034	histone cluster 1, H1b (Hist1h1b), mRNA. (S)	Hist1h1b	histone cluster 1, H1b	Hist1h1b	2.21E-03	-2.856
ILMN_2846485	NM_016982	pre-B lymphocyte gene 1 (Vpreb1), mRNA. (S)	Vpreb1	pre-B lymphocyte gene 1	Vpreb1	1.24E-02	-2.843
ILMN_1257463	BC051418	NaN (S)	Bcl11a	B-cell CLL/lymphoma 11A (zinc finger protein)	BCL11A	3.05E-02	-2.641
ILMN_2761370	NM_010229	FMS-like tyrosine kinase 3 (Flt3), mRNA. (S)	Flt3	FMS-like tyrosine kinase 3	FLT3	1.09E-02	-2.318
ILMN_1241177	AK051132	NaN (S)	D130003E24Rik	n/a	n/a	3.03E-02	-2.290
ILMN_2817214	NM_175666	histone cluster 2, H2bb (Hist2h2bb), mRNA. (S)	Hist2h2bb	histone cluster 1, H2bg; histone cluster 1, H2be; histone cluster 2, H2bb; histone cluster 1, H2bc	Hist1h2bc	3.70E-04	-2.275
ILMN_2692110	NM_009361	NaN (S)	Tfdp1	predicted gene 7390; transcription factor Dp 1; similar to Transcription factor Dp-1 (E2F dimerization partner 1) (DRTF1-polypeptide 1)	LOC674691	1.13E-02	-2.215
ILMN_2517041	NM_010931	ubiquitin-like, containing PHD and RING finger domains, 1 (Uhrf1), mRNA. (S)	Uhrf1	ubiquitin-like, containing PHD and RING finger domains, 1; predicted gene 5648; similar to nuclear zinc finger protein Np95	UHRF1	2.55E-05	-2.213
ILMN_1257520	NM_178201	histone cluster 1, H2bn (Hist1h2bn), mRNA. (S)	Hist1h2bn	similar to Hist1h2bj protein; histone cluster 1, H2bl; predicted gene, OTTMUSG00000013203; histone cluster 1, H2bj; histone cluster 1, H2bf; H2b histone family member; histone cluster 1, H2bn	Gm13646	1.35E-02	-2.157

ILMN_2542331	XM_358107	NaN (S)	LOC385187	n/a	n/a	4.85E-06	-2.125
ILMN_2907878	NM_010436	H2A histone family, member X (H2afx), mRNA. (S)	H2afx	H2A histone family, member X	H2afx	6.09E-03	-2.115
ILMN_1221568	NM_025866	cell division cycle associated 7 (Cdca7), mRNA. (S)	Cdca7	cell division cycle associated 7	cdca7	1.81E-05	-2.113
ILMN_1238894	NM_016777	nuclear autoantigenic sperm protein (histone-binding) (Nasp), transcript variant 2, mRNA. (S)	Nasp	nuclear autoantigenic sperm protein (histone-binding); similar to nuclear autoantigenic sperm protein; NASP	NASP	5.18E-04	-2.106
ILMN_2527129	XM_287286	NaN (S)	LOC329750	n/a	n/a	6.21E-03	-2.101
ILMN_2819558	NM_007521	BTB and CNC homology 2 (Bach2), mRNA. (S)	Bach2	BTB and CNC homology 2	Bach2	8.24E-03	-2.083
ILMN_2916035	NM_010721	lamin B1 (Lmnb1), mRNA. (S)	Lmnb1	lamin B1	LMNB1	1.03E-02	-2.074
ILMN_2755384	NM_025900	DEK oncogene (DNA binding) (Dek), mRNA. (S)	Dek	DEK oncogene (DNA binding)	Dek	1.56E-03	-2.065
ILMN_1252076	NM_017372	lysozyme 2 (Lyz2), mRNA. (S)	Lyz2	lysozyme 2	LYZ2	3.77E-02	-2.055
ILMN_1237993	NM_016777	nuclear autoantigenic sperm protein (histone-binding) (Nasp), transcript variant 2, mRNA. (S)	Nasp	nuclear autoantigenic sperm protein (histone-binding); similar to nuclear autoantigenic sperm protein; NASP	NASP	2.81E-03	-2.041
ILMN_1241857	NM_015781	nucleosome assembly protein 1-like 1 (Nap1l1), mRNA. (S)	Nap1l1	similar to nucleosome assembly protein 1-like 1; nucleosome assembly protein 1-like 1	NAP1L1	1.52E-03	-2.033
ILMN_2606429	NM_007971	enhancer of zeste homolog 2 (Drosophila) (Ezh2), mRNA. (S)	Ezh2	enhancer of zeste homolog 2 (Drosophila)	Ezh2	3.61E-04	-2.019
ILMN_1227305	XM_358117	NaN (S)	LOC385205	n/a	n/a	1.59E-03	-2.015
ILMN_3055904	NM_001076789	chromobox homolog 5 (Drosophila HP1a) (Cbx5), transcript variant 2, mRNA. (I)	Cbx5	chromobox homolog 5 (Drosophila HP1a)	CBX5	2.83E-03	-2.011
ILMN_2428117	XM_001478125	PREDICTED: zinc finger, CCHC domain containing 2 (Zcchc2), mRNA. (S)	Zcchc2	zinc finger, CCHC domain containing 2	ZCCHC2	1.45E-02	-1.994
ILMN_1222228	NM_007415	poly (ADP-ribose) polymerase family, member 1 (Parp1), mRNA. (S)	Parp1	poly (ADP-ribose) polymerase family, member 1	parp1	3.25E-02	-1.969

ILMN_2602855	XM_133073	PREDICTED: nucleoporin 205, transcript variant 1 (Nup205), mRNA. (S)	Nup205	nucleoporin 205	NUP205	8.09E-04	-1.958
ILMN_2541380	XM_358005	NaN (S)	LOC385032	n/a	n/a	1.85E-04	-1.938
ILMN_2719202	NM_178195	histone cluster 1, H2bf (Hist1h2bf), mRNA. (S)	Hist1h2bf	similar to Hist1h2bj protein; histone cluster 1, H2bl; predicted gene, OTTMUSG00000013203; histone cluster 1, H2bj; histone cluster 1, H2bf; H2b histone family member; histone cluster 1, H2bn	Gm13646	1.40E-02	-1.933
ILMN_1233116	NM_008696	mitogen-activated protein kinase kinase kinase kinase 4 (Map4k4), mRNA. (S)	Map4k4	mitogen-activated protein kinase kinase kinase kinase 4	MAP4K4	1.06E-02	-1.923
ILMN_1240571	NM_009955	NaN (S)	Dpysl2	dihydropyrimidinase-like 2	Dpysl2	2.53E-03	-1.916
ILMN_2987062	NM_144543	thymocyte nuclear protein 1 (Thyn1), mRNA. (S)	Thyn1	thymocyte nuclear protein 1	THYN1	2.86E-02	-1.916
ILMN_1250907	NM_008564	minichromosome maintenance deficient 2 mitotin (S. cerevisiae) (Mcm2), mRNA. (S)	Mcm2	minichromosome maintenance deficient 2 mitotin (S. cerevisiae)	mcm2	2.30E-02	-1.914
ILMN_2951691	NM_178205	histone cluster 1, H3e (Hist1h3e), mRNA. (S)	Hist1h3e	histone cluster 2, H3b; histone cluster 1, H3f; histone cluster 1, H3e; histone cluster 2, H3c1; histone cluster 1, H3d; histone cluster 1, H3c; histone cluster 1, H3b; histone cluster 2, H3c2; histone cluster 2, H2aa1; histone cluster 2, H2aa2	HIST2H3B	4.38E-02	-1.913
ILMN_2732795	NM_001085387	myosin light chain 2, precursor lymphocyte-specific (Mylc2pl), transcript variant 2, mRNA. (S)	Mylc2pl	myosin, light chain 10, regulatory	myl10	3.99E-02	-1.901
ILMN_1248132	NM_019641	NaN (S)	Stmn1	stathmin 1; predicted gene 11223; predicted gene 6393	Stmn1-rs2	2.91E-03	-1.883
ILMN_1232524	NM_175656	histone cluster 1, H4i (Hist1h4i), mRNA. (S)	Hist1h4i	histone cluster 1, H4k; histone cluster 1, H4m; histone cluster 4, H4; similar to germinal histone H4 gene; histone cluster 1, H4h; histone cluster 1, H4j; histone cluster 1, H4i; histone cluster 1, H4d; histone cluster 1, H4c; histone cluster 1, H4f; histone cluster 1, H4b; histone cluster 1, H4a; histone cluster 2, H4; similar to histone H4	Hist1h4c	1.91E-02	-1.883
ILMN_2721231	NM_009391	RAN, member RAS oncogene family (Ran), mRNA. (S)	Ran	RAS-like, family 2, locus 9; RAN, member RAS oncogene family; similar to RAN, member RAS oncogene family	LOC100045999	1.30E-03	-1.867

ILMN_2747651	NM_026115	histone aminotransferase 1 (Hat1), mRNA. (S)	Hat1	histone aminotransferase 1	HAT1	3.93E-03	-1.866
ILMN_1227793	NM_015753	zinc finger E-box binding homeobox 2 (Zeb2), transcript variant 2, mRNA. (S)	Zeb2	zinc finger E-box binding homeobox 2	Zeb2	2.09E-03	-1.864
ILMN_2985128	NM_175652	histone cluster 4, H4 (Hist4h4), mRNA. (S)	Hist4h4	histone cluster 1, H4k; histone cluster 1, H4m; histone cluster 4, H4; similar to germinal histone H4 gene; histone cluster 1, H4h; histone cluster 1, H4j; histone cluster 1, H4i; histone cluster 1, H4d; histone cluster 1, H4c; histone cluster 1, H4f; histone cluster 1, H4b; histone cluster 1, H4a; histone cluster 2, H4; similar to histone H4	Hist1h4c	1.30E-02	-1.862
ILMN_3004142	NM_021420	serine/threonine kinase 4 (Stk4), mRNA. (S)	Stk4	serine/threonine kinase 4	STK4	4.36E-02	-1.862
ILMN_1236125	NM_008567	minichromosome maintenance deficient 6 (MIS5 homolog, S. pombe) (S. cerevisiae) (Mcm6), mRNA. (S)	Mcm6	minichromosome maintenance deficient 6 (MIS5 homolog, S. pombe) (S. cerevisiae)	MCM6	4.03E-02	-1.856
ILMN_2572235	AK053176	NaN (S)	Il18rap	interleukin 18 receptor accessory protein	IL18RAP	5.77E-03	-1.856
ILMN_1213616	NM_008568	minichromosome maintenance deficient 7 (S. cerevisiae) (Mcm7), mRNA. (S)	Mcm7	minichromosome maintenance deficient 7 (S. cerevisiae)	MCM7	6.37E-03	-1.855
ILMN_2742152	NM_007836	growth arrest and DNA-damage-inducible 45 alpha (Gadd45a), mRNA. (S)	Gadd45a	growth arrest and DNA-damage-inducible 45 alpha	GADD45A	4.63E-03	-1.855
ILMN_3009860	NM_011346	selectin, lymphocyte (Sell), mRNA. (S)	Sell	selectin, lymphocyte	SELL	5.45E-03	-1.850
ILMN_1248830	NM_178184	histone cluster 1, H2an (Hist1h2an), mRNA. (S)	Hist1h2an	histone cluster 1, H2ad; histone cluster 1, H2ae; histone cluster 1, H2ag; histone cluster 1, H2ah; histone cluster 1, H2ai; similar to histone 2a; histone cluster 1, H2an; histone cluster 1, H2ao; histone cluster 1, H2ac; histone cluster 1, H2ab	Hist1h2ab	2.62E-03	-1.837

ILMN_1256989	NM_178210	histone cluster 1, H4j (Hist1h4j), mRNA. (S)	Hist1h4j	histone cluster 1, H4k; histone cluster 1, H4m; histone cluster 4, H4; similar to germinal histone H4 gene; histone cluster 1, H4h; histone cluster 1, H4j; histone cluster 1, H4i; histone cluster 1, H4d; histone cluster 1, H4c; histone cluster 1, H4f; histone cluster 1, H4b; histone cluster 1, H4a; histone cluster 2, H4; similar to histone H4	Hist1h4c	1.43E-02	-1.828
ILMN_1234959	XM_132006	NaN (S)	Whsc1	n/a	n/a	2.27E-02	-1.824
ILMN_2891646	NM_178200	histone cluster 1, H2bm (Hist1h2bm), mRNA. (S)	Hist1h2bm	histone cluster 1, H2bm	HIST1H2BM	7.74E-03	-1.823
ILMN_2740902	XM_001000692	PREDICTED: antigen identified by monoclonal antibody Ki 67 (Mki67), mRNA. (S)	Mki67	antigen identified by monoclonal antibody Ki 67	MKI67	2.29E-02	-1.820
ILMN_2525570	NM_178211	histone cluster 1, H4k (Hist1h4k), mRNA. (S)	Hist1h4k	histone cluster 1, H4k; histone cluster 1, H4m; histone cluster 4, H4; similar to germinal histone H4 gene; histone cluster 1, H4h; histone cluster 1, H4j; histone cluster 1, H4i; histone cluster 1, H4d; histone cluster 1, H4c; histone cluster 1, H4f; histone cluster 1, H4b; histone cluster 1, H4a; histone cluster 2, H4; similar to histone H4	Hist1h4c	4.37E-02	-1.817
ILMN_2745425	NM_133878	regulator of chromosome condensation 1 (Rcc1), mRNA. (S)	Rcc1	regulator of chromosome condensation 1	rcc1	1.00E-03	-1.817
ILMN_1228334	NM_023203	RIKEN cDNA 2410015N17 gene (2410015N17Rik), mRNA. (S)	2410015N17Rik	dCTP pyrophosphatase 1	DCTPP1	2.72E-02	-1.811
ILMN_2759159	NM_178192	histone cluster 1, H4a (Hist1h4a), mRNA. (S)	Hist1h4a	histone cluster 1, H4k; histone cluster 1, H4m; histone cluster 4, H4; similar to germinal histone H4 gene; histone cluster 1, H4h; histone cluster 1, H4j; histone cluster 1, H4i; histone cluster 1, H4d; histone cluster 1, H4c; histone cluster 1, H4f; histone cluster 1, H4b; histone cluster 1, H4a; histone cluster 2, H4; similar to histone H4	Hist1h4c	7.45E-03	-1.811
ILMN_1219094	AK085249	NaN (S)	D530050H15Rik	predicted gene 9938	Gm9938	4.85E-02	-1.810
ILMN_1232116	XM_195821	NaN (S)	LOC268569	n/a	n/a	3.69E-03	-1.807

ILMN_2836654	NM_178185	histone cluster 1, H2ao (Hist1h2ao), mRNA. (S)	Hist1h2ao	histone cluster 1, H2ad; histone cluster 1, H2ae; histone cluster 1, H2ag; histone cluster 1, H2ah; histone cluster 1, H2ai; similar to histone 2a; histone cluster 1, H2an; histone cluster 1, H2ao; histone cluster 1, H2ac; histone cluster 1, H2ab	Hist1h2ab	2.67E-03	-1.806
ILMN_2937548	NM_175657	histone cluster 1, H4m (Hist1h4m), mRNA. (S)	Hist1h4m	histone cluster 1, H4k; histone cluster 1, H4m; histone cluster 4, H4; similar to germinal histone H4 gene; histone cluster 1, H4h; histone cluster 1, H4j; histone cluster 1, H4i; histone cluster 1, H4d; histone cluster 1, H4c; histone cluster 1, H4f; histone cluster 1, H4b; histone cluster 1, H4a; histone cluster 2, H4; similar to histone H4	Hist1h4c	3.17E-02	-1.804
ILMN_2819319	NM_009104	ribonucleotide reductase M2 (Rrm2), mRNA. (S)	Rrm2	ribonucleotide reductase M2	rrm2	4.19E-04	-1.803
ILMN_2453874	NM_001098227	syndecan binding protein (Sdcbp), transcript variant 1, mRNA. (S)	Sdcbp	similar to syntenin; syndecan binding protein	LOC100047309	5.51E-03	-1.801
ILMN_2533143	XM_134040	NaN (S)	LOC234081	n/a	n/a	3.84E-02	-1.793
ILMN_1255581	XR_001819	PREDICTED: similar to nuclease sensitive element binding protein 1 (LOC630936), misc RNA. (S)	LOC630936	predicted gene 6540; predicted gene 11560; similar to nuclease sensitive element binding protein 1; Y box protein 1	Gm6540	3.67E-02	-1.787
ILMN_1215681	NM_007590	calmodulin 3 (Calm3), mRNA. (S)	Calm3	predicted gene 7743; calmodulin 3; calmodulin 2; calmodulin 1; predicted gene 7308	Gm7308	1.01E-03	-1.781
ILMN_1226839	NM_178188	histone cluster 1, H2ad (Hist1h2ad), mRNA. (S)	Hist1h2ad	histone cluster 1, H2ad; histone cluster 1, H2ae; histone cluster 1, H2ag; histone cluster 1, H2ah; histone cluster 1, H2ai; similar to histone 2a; histone cluster 1, H2an; histone cluster 1, H2ao; histone cluster 1, H2ac; histone cluster 1, H2ab	Hist1h2ab	2.78E-03	-1.781
ILMN_2716195	NM_178197	histone cluster 1, H2bh (Hist1h2bh), mRNA. (S)	Hist1h2bh	histone cluster 1, H2bh	Hist1h2bh	3.42E-02	-1.779
ILMN_2654932	NM_001033313	PDGFA associated protein 1 (Pdap1), mRNA. (S)	Pdap1	PDGFA associated protein 1	PDAP1	2.65E-02	-1.777
ILMN_1219433	XR_003570	PREDICTED: similar to High mobility group protein 1 (HMG-1) (High mobility group protein B1) (Amphoterin) (Heparin-binding protein p30) (LOC671784), mRNA. (S)	LOC671784	n/a	n/a	3.27E-04	-1.767

ILMN_1212989	XM_001476780	PREDICTED: similar to histone macroH2A1.2 (LOC100046770), mRNA. (S)	LOC100046770	H2A histone family, member Y	H2AFY	9.60E-04	-1.767
ILMN_2669404	NM_010722	lamin B2 (Lmnb2), mRNA. (S)	Lmnb2	lamin B2	Lmnb2	2.33E-02	-1.761
ILMN_2866970	NM_010615	kinesin family member 11 (Kif11), mRNA. (S)	Kif11	kinesin family member 11	KIF11	4.17E-02	-1.759
ILMN_1238276	NM_178182	histone cluster 1, H2ai (Hist1h2ai), mRNA. (S)	Hist1h2ai	histone cluster 1, H2ad; histone cluster 1, H2ae; histone cluster 1, H2ag; histone cluster 1, H2ah; histone cluster 1, H2ai; similar to histone 2a; histone cluster 1, H2an; histone cluster 1, H2ao; histone cluster 1, H2ac; histone cluster 1, H2ab	Hist1h2ab	9.34E-03	-1.759
ILMN_2742849	NM_008566	minichromosome maintenance deficient 5, cell division cycle 46 (S. cerevisiae) (Mcm5), mRNA. (S)	Mcm5	minichromosome maintenance deficient 5, cell division cycle 46 (S. cerevisiae)	Mcm5	8.06E-03	-1.758
ILMN_1218380	NM_175662	histone cluster 2, H2ac (Hist2h2ac), mRNA. (S)	Hist2h2ac	histone cluster 2, H2ac	Hist2h2ac	6.66E-03	-1.753
ILMN_2968907	NM_025372	timeless interacting protein (Tipin), mRNA. (S)	Tipin	timeless interacting protein	TIPIN	1.88E-02	-1.753
ILMN_1231138	NM_007891	E2F transcription factor 1 (E2f1), mRNA. (S)	E2f1	E2F transcription factor 1	E2F1	1.36E-02	-1.752
ILMN_2983686	NM_145520	TruB pseudouridine (psi) synthase homolog 2 (E. coli) (Trub2), mRNA. (S)	Trub2	TruB pseudouridine (psi) synthase homolog 2 (E. coli)	trub2	4.14E-02	-1.750
ILMN_1234257	NM_027901	general transcription factor IIIC, polypeptide 2, beta (Gtf3c2), mRNA. (S)	Gtf3c2	general transcription factor IIIC, polypeptide 2, beta; Mpv17 transgene, kidney disease mutant	MPV17	1.36E-02	-1.750
ILMN_1243654	NM_178211	histone cluster 1, H4k (Hist1h4k), mRNA. (S)	Hist1h4k	histone cluster 1, H4k; histone cluster 1, H4m; histone cluster 4, H4; similar to germinal histone H4 gene; histone cluster 1, H4h; histone cluster 1, H4j; histone cluster 1, H4i; histone cluster 1, H4d; histone cluster 1, H4c; histone cluster 1, H4f; histone cluster 1, H4b; histone cluster 1, H4a; histone cluster 2, H4; similar to histone H4	Hist1h4c	3.55E-02	-1.749

ILMN_2745212	NM_178182	histone cluster 1, H2ai (Hist1h2ai), mRNA. (S)	Hist1h2ai	histone cluster 1, H2ad; histone cluster 1, H2ae; histone cluster 1, H2ag; histone cluster 1, H2ah; histone cluster 1, H2ai; similar to histone 2a; histone cluster 1, H2an; histone cluster 1, H2ao; histone cluster 1, H2ac; histone cluster 1, H2ab	Hist1h2ab	3.81E-03	-1.748
ILMN_1255633	AK051805	NaN (S)	D130099D04Rik	sodium channel, voltage-gated, type III, beta	SCN3B	2.59E-02	-1.748
ILMN_1230265	NM_175655	histone cluster 1, H4f (Hist1h4f), mRNA. (S)	Hist1h4f	histone cluster 1, H4k; histone cluster 1, H4m; histone cluster 4, H4; similar to germinal histone H4 gene; histone cluster 1, H4h; histone cluster 1, H4j; histone cluster 1, H4i; histone cluster 1, H4d; histone cluster 1, H4c; histone cluster 1, H4f; histone cluster 1, H4b; histone cluster 1, H4a; histone cluster 2, H4; similar to histone H4	Hist1h4c	3.14E-02	-1.745
ILMN_2626190	XM_001474702	PREDICTED: similar to Nedd4 binding protein 2 (LOC100044124), mRNA. (S)	LOC100044124	similar to Nedd4 binding protein 2; NEDD4 binding protein 2	N4bp2	3.96E-02	-1.745
ILMN_2977624	NM_009103	ribonucleotide reductase M1 (Rrm1), mRNA. (S)	Rrm1	ribonucleotide reductase M1	Rrm1	9.20E-03	-1.741
ILMN_2622209	NM_026001	ribonuclease H2, subunit B (Rnaseh2b), mRNA. (S)	Rnaseh2b	ribonuclease H2, subunit B	RNASEH2B	4.84E-02	-1.739
ILMN_2615015	NM_133198	liver glycogen phosphorylase (Pygl), mRNA. (S)	Pygl	liver glycogen phosphorylase	PYGL	5.35E-03	-1.738
ILMN_3074985	NM_016750	H2A histone family, member Z (H2afz), mRNA. (I)	H2afz	H2A histone family, member Z; predicted gene 6722; predicted gene 8203	H2AFZ	5.86E-03	-1.738
ILMN_2830661	NM_011623	topoisomerase (DNA) II alpha (Top2a), mRNA. (S)	Top2a	topoisomerase (DNA) II alpha	TOP2A	8.06E-03	-1.729
ILMN_2602542	NM_008511	NaN (S)	Lrmp	lymphoid-restricted membrane protein	LRMP	1.33E-02	-1.725
ILMN_1241976	NM_133835	ubiquitin associated domain containing 1 (Ubac1), mRNA. (S)	Ubac1	ubiquitin associated domain containing 1	ubac1	9.82E-03	-1.723
ILMN_1237677	NM_025814	Serpine1 mRNA binding protein 1 (Serbp1), mRNA. (S)	Serbp1	serpine1 mRNA binding protein 1	serbp1	3.95E-02	-1.718
ILMN_2744398	NM_017375	osteoclast stimulating factor 1 (Ostf1), mRNA. (S)	Ostf1	osteoclast stimulating factor 1	ostf1	3.81E-02	-1.718
ILMN_2675669	NM_007654	CD72 antigen (Cd72), mRNA. (S)	Cd72	CD72 antigen	CD72	2.28E-02	-1.714

ILMN_2541505	NM_177184	vacuolar protein sorting 13C (yeast) (Vps13c), mRNA. (S)	Vps13c	vacuolar protein sorting 13C (yeast)	Vps13c	1.94E-02	-1.707
ILMN_1237672	NM_011234	RAD51 homolog (S. cerevisiae) (Rad51), mRNA. (S)	Rad51	RAD51 homolog (S. cerevisiae)	rad51	2.56E-03	-1.704
ILMN_2610744	NM_010858	myosin, light polypeptide 4 (Myl4), mRNA. (S)	Myl4	myosin, light polypeptide 4	MYL4	2.39E-02	-1.703
ILMN_1219574	NM_175661	histone cluster 1, H2af (Hist1h2af), mRNA. (S)	Hist1h2af	histone cluster 1, H2af	Hist1h2af	5.01E-03	-1.702
ILMN_1239878	NM_178372	protease, serine, 34 (Prss34), mRNA. (S)	Prss34	protease, serine, 34	Prss34	3.97E-02	-1.701
ILMN_3145213	NM_008210	H3 histone, family 3A (H3f3a), mRNA. (A)	H3f3a	predicted gene 14383; predicted gene 3835; predicted gene 14384; predicted gene 12950; predicted gene, 670915; H3 histone, family 3A; predicted gene 12657; predicted gene 6132; predicted gene 10257; predicted gene 7227; H3 histone, family 3B; predicted gene 6128; similar to histone; predicted gene 1986; predicted gene 6186; hypothetical protein LOC676337; predicted gene 6421; predicted gene 2198; predicted gene 6817; predicted gene 8095; predicted gene 12271; predicted gene 13529; predicted gene 8029; predicted gene 4938; predicted gene 7100; predicted gene 9014; similar to Histone H3.4 (Embryonic); predicted gene 7179; similar to H3 histone, family 3B; predicted gene 7900; predicted gene 2099; similar to H3 histone, family 3A; predicted gene 6749; predicted gene 6485; predicted gene 4028; predicted gene 7194	Gm3835	1.45E-02	-1.700
ILMN_2712751	XM_122922	NaN (S)	Hnrpa1	n/a	n/a	1.93E-03	-1.697
ILMN_1232189	XM_284494	PREDICTED: DEAD (Asp-Glu-Ala-Asp) box polypeptide 10 (Ddx10), mRNA. (S)	Ddx10	DEAD (Asp-Glu-Ala-Asp) box polypeptide 10	ddx10	5.29E-03	-1.697
ILMN_2636781	NM_175665	histone cluster 1, H2bk (Hist1h2bk), mRNA. (S)	Hist1h2bk	histone cluster 1, H2bk	HIST1H2BK	4.36E-02	-1.694
ILMN_2592253	NM_178206	histone cluster 1, H3h (Hist1h3h), mRNA. (S)	Hist1h3h	histone cluster 1, H3i; histone cluster 1, H3h; histone cluster 1, H3g; predicted gene 12260; histone cluster 1, H3a	hist1h3g	3.92E-02	-1.694

ILMN_2632712	NM_009689	baculoviral IAP repeat-containing 5 (Birc5), transcript variant 1, mRNA. (S)	Birc5	baculoviral IAP repeat-containing 5	BIRC5	4.52E-05	-1.693
ILMN_2674979	NM_139149	NaN (S)	Fus	fusion, derived from t(12;16) malignant liposarcoma (human)	fus	2.04E-02	-1.692
ILMN_2668243	NM_019648	methionine aminopeptidase 2 (Metap2), mRNA. (S)	Metap2	methionine aminopeptidase 2	metap2	3.17E-02	-1.691
ILMN_1225312	NM_007450	solute carrier family 25 (mitochondrial carrier, adenine nucleotide translocator), member 4 (Slc25a4), mRNA. (S)	Slc25a4	solute carrier family 25 (mitochondrial carrier, adenine nucleotide translocator), member 4	SLC25A4	3.95E-04	-1.690
ILMN_1247933	NM_013590	lysozyme 1 (Lyz1), mRNA. (S)	Lyz1	lysozyme 1	Lyz1	8.95E-03	-1.689
ILMN_1238026	AK050974	NaN (S)	D030048H11Rik	progesterone immunomodulatory binding factor 1	pibf1	1.29E-02	-1.687
ILMN_2622208	XM_127746	NaN (S)	2610207P08Rik	n/a	n/a	1.44E-02	-1.684
ILMN_2752817	NM_010848	myeloblastosis oncogene (Myb), mRNA. (S)	Myb	myeloblastosis oncogene	myb	1.37E-03	-1.678
ILMN_2635205	NM_011033	poly A binding protein, cytoplasmic 2 (Pabpc2), mRNA. (S)	Pabpc2	poly(A) binding protein, cytoplasmic 2	Pabpc2	1.53E-02	-1.678
ILMN_2742592	NM_178194	histone cluster 1, H2be (Hist1h2be), mRNA. (S)	Hist1h2be	histone cluster 1, H2bg; histone cluster 1, H2be; histone cluster 2, H2bb; histone cluster 1, H2bc	Hist1h2bc	1.18E-02	-1.675
ILMN_2639081	NM_016957	NaN (S)	Hmgn2	high mobility group nucleosomal binding domain 4; predicted gene 7931; predicted gene 10282; predicted gene 3338; high mobility group nucleosomal binding domain 2; predicted gene 6594; predicted gene 6750; predicted gene 10182; predicted gene 6724; predicted gene 4248; predicted gene 9525; predicted gene 15296; similar to Hmgn2 protein; predicted gene 7125; hypothetical protein LOC638323; predicted gene 6651; predicted gene 5899; predicted gene 14008; predicted gene 16494; similar to high mobility group nucleosomal binding domain 2	Gm10182	2.52E-03	-1.671
ILMN_2715802	NM_016660	high mobility group AT-hook 1 (Hmga1), transcript variant 1, mRNA. (S)	Hmga1	high mobility group AT-hook I, related sequence 1; high mobility group AT-hook 1	Hmga1-rs1	3.39E-02	-1.669

ILMN_2655577	NM_011131	polymerase (DNA directed), delta 1, catalytic subunit (Pold1), mRNA. (S)	Pold1	polymerase (DNA directed), delta 1, catalytic subunit	Pold1	2.66E-02	-1.666
ILMN_2664548	NM_025396	6-phosphogluconolactonase (Pgls), mRNA. (S)	Pgls	6-phosphogluconolactonase	pgls	4.62E-02	-1.666
ILMN_1257178	NM_175494	zinc finger protein 367 (Zfp367), mRNA. (S)	Zfp367	zinc finger protein 367	Zfp367	4.31E-03	-1.666
ILMN_2648376	XM_001481214	PREDICTED: similar to nuclear pore-targeting complex component of 58 kDa, transcript variant 1 (LOC100043906), mRNA. (S)	LOC100043906	similar to ribosomal protein L38; predicted gene 13020; ribosomal protein L38; predicted gene 4991; karyopherin (importin) alpha 2; predicted gene 9028; predicted gene 8129; predicted gene 7123; predicted gene 5832; predicted gene 10184; predicted gene 7379; predicted gene 10259	Gm7379	2.18E-02	-1.665
ILMN_1216380	NM_008383	centrosomal protein 250 (Cep250), mRNA. XM_902553 XM_907859 XM_920619 XM_920625 XM_920632 (S)	Cep250	centrosomal protein 250	CEP250	1.58E-02	-1.664
ILMN_2691192	NM_009031	retinoblastoma binding protein 7 (Rbbp7), mRNA. (S)	Rbbp7	retinoblastoma binding protein 7; predicted gene 6382	Gm6382	4.97E-02	-1.663
ILMN_1220726	NM_009122	special AT-rich sequence binding protein 1 (Satb1), mRNA. (S)	Satb1	special AT-rich sequence binding protein 1	Satb1	3.06E-03	-1.663
ILMN_3088934	NM_001037279	RIKEN cDNA 2700094K13 gene (2700094K13Rik), transcript variant 2, mRNA. (A)	2700094K13Rik	RIKEN cDNA 2700094K13 gene	2700094K13Rik	3.34E-02	-1.663
ILMN_2772930	NM_008114	NaN (S)	Gfi1b	growth factor independent 1B	GFI1B	4.05E-02	-1.653
ILMN_2627660	NM_010715	ligase I, DNA, ATP-dependent (Lig1), transcript variant 2, mRNA. (S)	Lig1	ligase I, DNA, ATP-dependent	lig1	1.71E-02	-1.653
ILMN_1222226	NM_020505	vav 3 oncogene (Vav3), transcript variant 1, mRNA. (S)	Vav3	vav 3 oncogene	VAV3	1.64E-04	-1.646
ILMN_2826027	NM_180600	ubiquitin-conjugating enzyme E2Q (putative) 2 (Ube2q2), mRNA. (S)	Ube2q2	ubiquitin-conjugating enzyme E2Q (putative) 2	UBE2Q2	1.39E-03	-1.644

ILMN_1246576	XM_001476063	PREDICTED: similar to histone H4 (LOC100041230), mRNA. (S)	LOC100041230	histone cluster 1, H4k; histone cluster 1, H4m; histone cluster 4, H4; similar to germinal histone H4 gene; histone cluster 1, H4h; histone cluster 1, H4j; histone cluster 1, H4i; histone cluster 1, H4d; histone cluster 1, H4c; histone cluster 1, H4f; histone cluster 1, H4b; histone cluster 1, H4a; histone cluster 2, H4; similar to histone H4	Hist1h4c	3.64E-02	-1.642
ILMN_2639036	NM_010477	heat shock protein 1 (chaperonin) (Hspd1), mRNA. (S)	Hspd1	predicted gene 12141; heat shock protein 1 (chaperonin)	Gm12141	3.43E-02	-1.641
ILMN_2533694	XR_031775	PREDICTED: similar to high-mobility group box 1 (LOC667303), misc RNA. (S)	LOC667303	predicted gene 13121; predicted gene 3160; high-mobility group (nonhistone chromosomal) protein 1-like 1; predicted gene 6090; predicted gene 3851; predicted gene 8967; predicted gene 7782; predicted gene 4587; predicted gene 4689; predicted gene 3307; predicted gene 13932; predicted gene 15059; predicted gene 3565; predicted gene 15447; predicted gene 12587; predicted gene 9012; predicted gene 6115; predicted gene 9480; high mobility group box 1; predicted gene 8423; predicted gene 5853; predicted gene 8288; predicted gene 7888; predicted gene 8594; predicted gene 15387; predicted gene 5473; predicted gene 8807; similar to high mobility group box 1; similar to 2810416G20Rik protein; predicted gene 8390; predicted gene, OTTMUSG00000005439; predicted gene 5842; predicted gene 5527; predicted gene 8563; predicted gene 2710; predicted gene 12331; predicted gene 5937; predicted gene 5504; similar to high-mobility group box 1; predicted gene 10361; predicted gene 2607; predicted gene 7422; predicted gene 10075; predicted gene 12568; predicted gene 6589; predicted gene 4383; predicted gene 8031; similar to High mobility group protein 1 (HMG-1) (High mobility group protein B1) (Amphoterin) (Heparin-binding protein p30); predicted gene 7468; predicted gene 8554	LOC674543	2.52E-04	-1.640
ILMN_1242842	NM_012015	H2A histone family, member Y (H2afy), mRNA. (S)	H2afy	H2A histone family, member Y	H2AFY	1.88E-02	-1.637
ILMN_2777804	NM_175251	AT rich interactive domain 2 (ARID, RFX-like) (Arid2), mRNA. (S)	Arid2	AT rich interactive domain 2 (ARID, RFX-like); RIKEN cDNA 1700124K17 gene	ARID2	4.06E-02	-1.636

ILMN_2858359	NM_175554	claspin homolog (Xenopus laevis) (Clspn), mRNA. (S)	Clspn	claspin homolog (Xenopus laevis)	clspn	2.37E-02	-1.635
ILMN_2837855	NM_022309	core binding factor beta (Cbfb), mRNA. (S)	Cbfb	core binding factor beta	Cbfb	3.97E-02	-1.634
ILMN_2652511	NM_178198	histone cluster 1, H2bj (Hist1h2bj), mRNA. (S)	Hist1h2bj	similar to Hist1h2bj protein; histone cluster 1, H2bl; predicted gene, OTTMUSG00000013203; histone cluster 1, H2bj; histone cluster 1, H2bf; H2b histone family member; histone cluster 1, H2bn	Gm13646	2.72E-02	-1.633
ILMN_3067068	NM_025284	thymosin, beta 10 (Tmsb10), mRNA. (I)	Tmsb10	predicted gene 3787; predicted gene 9844; predicted gene 8034; similar to thymosin, beta 10; thymosin, beta 10	LOC100048142	3.57E-02	-1.632
ILMN_2962958	NM_010948	nuclear distribution gene C homolog (Aspergillus) (Nudc), mRNA. (S)	Nudc	nuclear distribution gene C homolog (Aspergillus), pseudogene 1; nuclear distribution gene C homolog (Aspergillus)	nudC	4.61E-02	-1.631
ILMN_1251930	NM_016777	nuclear autoantigenic sperm protein (histone-binding) (Nasp), transcript variant 2, mRNA. (S)	Nasp	nuclear autoantigenic sperm protein (histone-binding); similar to nuclear autoantigenic sperm protein; NASP	NASP	1.21E-02	-1.627
ILMN_1244773	NM_001037279	RIKEN cDNA 2700094K13 gene (2700094K13Rik), transcript variant 2, mRNA. (S)	2700094K13Rik	RIKEN cDNA 2700094K13 gene	2700094K13Rik	2.17E-02	-1.623
ILMN_1219275	NM_009031	retinoblastoma binding protein 7 (Rbbp7), mRNA. (S)	Rbbp7	retinoblastoma binding protein 7; predicted gene 6382	Gm6382	3.15E-02	-1.622
ILMN_1260286	NM_177632	cDNA sequence BC022623 (BC022623), mRNA. (S)	BC022623	family with sequence similarity 43, member A	fam43a	1.53E-03	-1.618
ILMN_2751228	NM_026023	NudC domain containing 2 (Nudcd2), mRNA. (S)	Nudcd2	NudC domain containing 2	NUDCD2	1.15E-03	-1.617
ILMN_2597653	NM_175657	histone cluster 1, H4m (Hist1h4m), mRNA. (S)	Hist1h4m	histone cluster 1, H4k; histone cluster 1, H4m; histone cluster 4, H4; similar to germinal histone H4 gene; histone cluster 1, H4h; histone cluster 1, H4j; histone cluster 1, H4i; histone cluster 1, H4d; histone cluster 1, H4c; histone cluster 1, H4f; histone cluster 1, H4b; histone cluster 1, H4a; histone cluster 2, H4; similar to histone H4	Hist1h4c	3.64E-02	-1.616
ILMN_2621385	NM_007415	poly (ADP-ribose) polymerase family, member 1 (Parp1), mRNA. (S)	Parp1	poly (ADP-ribose) polymerase family, member 1	parp1	3.74E-02	-1.615

ILMN_1253477	XM_001474993	PREDICTED: RIKEN cDNA 2700029M09 gene (2700029M09Rik), mRNA. (S)	2700029M09Rik	RIKEN cDNA 2700029M09 gene	2700029M09Rik	1.38E-02	-1.615
ILMN_2679447	NM_028398	alanine-glyoxylate aminotransferase 2-like 2 (Agxt2l2), mRNA. (S)	Agxt2l2	alanine-glyoxylate aminotransferase 2-like 2	AGXT2L2	7.39E-03	-1.613
ILMN_2790373	NM_009223	stannin (Snn), mRNA. (S)	Snn	stannin	SNN	3.02E-02	-1.607
ILMN_2630521	NM_030609	histone cluster 1, H1a (Hist1h1a), mRNA. (S)	Hist1h1a	histone cluster 1, H1a	Hist1h1a	4.52E-02	-1.605
ILMN_3155245	NM_175476	Rho GTPase activating protein 25 (Arhgap25), transcript variant 2, mRNA. XM_899325 XM_899332 XM_899339 XM_899344 XM_914946 XM_923470 XM_923471 XM_923481 XM_923488 XM_923492 XM_923496 XM_923499 XM_923504 (A)	Arhgap25	Rho GTPase activating protein 25	Arhgap25	2.94E-02	-1.604
ILMN_2977903	NM_198631	cDNA sequence BC057627 (BC057627), mRNA. (S)	BC057627	zinc finger CCCH-type containing 4	Zc3h4	3.03E-03	-1.603
ILMN_2588337	NM_172681	NaN (S)	D930015E06Rik	RIKEN cDNA D930015E06 gene	D930015E06Rik	5.18E-03	-1.600
ILMN_2623056	NM_175554	claspin homolog (Xenopus laevis) (Clspn), mRNA. (S)	Clspn	claspin homolog (Xenopus laevis)	clspn	8.41E-03	-1.597
ILMN_3131063	NM_007632	cyclin D3 (Ccnd3), transcript variant 1, mRNA. (A)	Ccnd3	similar to Cyclin D3; cyclin D3	CCND3	1.03E-02	-1.596
ILMN_2800151	NM_030597	LSM2 homolog, U6 small nuclear RNA associated (S. cerevisiae) (Lsm2), mRNA. (S)	Lsm2	LSM2 homolog, U6 small nuclear RNA associated (S. cerevisiae)	LSM2	3.41E-03	-1.596
ILMN_2723881	NM_008224	host cell factor C1 (Hcfc1), mRNA. (S)	Hcfc1	similar to transcription factor C1 (HCF); host cell factor C1	LOC100046048	7.90E-03	-1.594
ILMN_1229110	NM_025796	mitochondrial ribosomal protein L33 (Mrpl33), nuclear gene encoding mitochondrial protein, mRNA. (S)	Mrpl33	mitochondrial ribosomal protein L33	MRPL33	3.80E-03	-1.594

ILMN_3086092	NM_146130	heterogeneous nuclear ribonucleoprotein A3 (Hnrpa3), transcript variant b, mRNA. (A)	Hnrpa3	predicted gene 7498; hypothetical protein LOC635773; predicted gene 9242; predicted gene 6793; predicted gene 14730; predicted gene 7551; predicted gene 14398; predicted gene 6528; predicted gene 6641; hypothetical protein LOC630507; predicted gene 5550; predicted gene 11847; predicted gene 6506; predicted gene 6153; predicted gene 5469; predicted gene 8991; similar to heterogeneous nuclear ribonucleoprotein A3; predicted gene 6758; predicted gene, OTTMUSG00000009698; heterogeneous nuclear ribonucleoprotein A3; predicted gene 5896	Gm5550	4.31E-03	-1.592
ILMN_2617496	NM_025900	DEK oncogene (DNA binding) (Dek), mRNA. (S)	Dek	DEK oncogene (DNA binding)	Dek	2.66E-02	-1.588
ILMN_2636456	NM_138593	NaN (S)	D3Wsu161e	similar to La ribonucleoprotein domain family, member 7; La ribonucleoprotein domain family, member 7	LARP7	5.82E-03	-1.588
ILMN_2912318	NM_009353	telomeric repeat binding factor 2 (Terf2), mRNA. (S)	Terf2	telomeric repeat binding factor 2	TERF2	4.21E-02	-1.587
ILMN_1245987	NM_025279	heterogeneous nuclear ribonucleoprotein K (Hnrnpk), mRNA. (S)	Hnrnpk	heterogeneous nuclear ribonucleoprotein K; predicted gene 7964	Gm7964	3.73E-03	-1.587
ILMN_2698799	NM_007393	actin, beta (Actb), mRNA. (S)	Actb	actin, beta	ACTB	2.58E-03	-1.587
ILMN_1229322	NM_011524	NaN (S)	Tacc3	n/a	n/a	3.94E-02	-1.584
ILMN_2837865	NM_026309	LSM3 homolog, U6 small nuclear RNA associated (S. cerevisiae) (Lsm3), mRNA. (S)	Lsm3	LSM3 homolog, U6 small nuclear RNA associated (S. cerevisiae)	LSM3	1.04E-02	-1.584
ILMN_1245081	NM_001081164	OTU domain containing 4 (Otud4), mRNA. (S)	Otud4	OTU domain containing 4	OTUD4	4.73E-03	-1.581
ILMN_2454823	XM_984113	PREDICTED: RIKEN cDNA 6720418B01 gene (6720418B01Rik), mRNA. (S)	6720418B01Rik	RIKEN cDNA 6720418B01 gene	6720418B01Rik	1.46E-03	-1.581
ILMN_2663570	NM_026217	autophagy-related 12 (yeast) (Atg12), mRNA. (S)	Atg12	autophagy-related 12 (yeast)	Atg12	3.85E-02	-1.580
ILMN_2933624	NR_002885	heterogeneous nuclear ribonucleoprotein A1 pseudogene (LOC654467) on chromosome 9. (S)	LOC654467	predicted gene 10052	Gm10052	8.54E-03	-1.580

ILMN_2795040	NM_178188	histone cluster 1, H2ad (Hist1h2ad), mRNA. (S)	Hist1h2ad	histone cluster 1, H2ad; histone cluster 1, H2ae; histone cluster 1, H2ag; histone cluster 1, H2ah; histone cluster 1, H2ai; similar to histone 2a; histone cluster 1, H2an; histone cluster 1, H2ao; histone cluster 1, H2ac; histone cluster 1, H2ab	Hist1h2ab	1.19E-02	-1.579
ILMN_2517228	NM_008960	phosphatase and tensin homolog (Pten), mRNA. (S)	Pten	phosphatase and tensin homolog	pten	6.47E-03	-1.578
ILMN_2905859	NM_019750	N-acetyltransferase 6 (Nat6), mRNA. (S)	Nat6	N-acetyltransferase 6	NAT6	9.72E-03	-1.578
ILMN_3133352	NM_011677	uracil DNA glycosylase (Ung), transcript variant 2, mRNA. (A)	Ung	uracil DNA glycosylase	ung	5.40E-04	-1.577
ILMN_2714565	NM_009104	NaN (S)	Rrm2	ribonucleotide reductase M2	rrm2	5.84E-03	-1.577
ILMN_1222589	AK082159	NaN (S)	C230016C14Rik	syntaxin binding protein 5-like	STXBP5L	2.10E-02	-1.575
ILMN_1252995	NM_178194	histone cluster 1, H2be (Hist1h2be), mRNA. (S)	Hist1h2be	histone cluster 1, H2bg; histone cluster 1, H2be; histone cluster 2, H2bb; histone cluster 1, H2bc	Hist1h2bc	2.98E-02	-1.573
ILMN_2921303	NR_000040	thymidylate synthase, pseudogene (Tyms-ps), non-coding RNA. (S)	Tyms-ps	thymidylate synthase, pseudogene	Tyms-ps	8.39E-03	-1.569
ILMN_2525620	NM_178193	histone cluster 1, H4b (Hist1h4b), mRNA. (S)	Hist1h4b	histone cluster 1, H4k; histone cluster 1, H4m; histone cluster 4, H4; similar to germinal histone H4 gene; histone cluster 1, H4h; histone cluster 1, H4j; histone cluster 1, H4i; histone cluster 1, H4d; histone cluster 1, H4c; histone cluster 1, H4f; histone cluster 1, H4b; histone cluster 1, H4a; histone cluster 2, H4; similar to histone H4	Hist1h4c	2.37E-02	-1.569
ILMN_2867835	NM_010798	macrophage migration inhibitory factor (Mif), mRNA. (S)	Mif	predicted gene 6097; macrophage migration inhibitory factor; similar to macrophage migration inhibitory factor; predicted gene 8762; predicted gene 10169; macrophage migration inhibitory factor-like	Gm16379	4.43E-03	-1.568
ILMN_1240408	AK033402	NaN (S)	Rbl1	retinoblastoma-like 1 (p107)	Rbl1	9.90E-03	-1.565
ILMN_2925711	NM_026268	dual specificity phosphatase 6 (Dusp6), mRNA. (S)	Dusp6	dual specificity phosphatase 6	dusp6	5.25E-03	-1.565

ILMN_1230808	XM_921371	PREDICTED: similar to Chromobox homolog 3 (HP1 gamma homolog, Drosophila), transcript variant 2 (LOC633016), mRNA. (S)	LOC633016	predicted gene 6917; similar to chromobox homolog 3; predicted gene 5792; predicted gene 7469; predicted gene 6901; predicted gene 7721; predicted gene 5196; complement component 7; chromobox homolog 3 (Drosophila HP1 gamma)	C7	4.47E-02	-1.564
ILMN_2755660	XR_030993	PREDICTED: similar to prothymosin alpha (LOC100044779), misc RNA. (S)	LOC100044779	predicted gene 12504; predicted gene 9800; predicted gene 4617; predicted gene 6625; predicted gene 7614; similar to prothymosin alpha; prothymosin alpha; predicted gene 9009	Gm7614	2.73E-05	-1.564
ILMN_1257579	NM_018815	nucleoporin 210 (Nup210), mRNA. (S)	Nup210	nucleoporin 210	Nup210	1.68E-02	-1.561
ILMN_2863965	NM_011072	profilin 1 (Pfn1), mRNA. (S)	Pfn1	profilin 1	pfn1	1.89E-02	-1.558
ILMN_1233064	NM_183417	cyclin-dependent kinase 2 (Cdk2), transcript variant 1, mRNA. (S)	Cdk2	cyclin-dependent kinase 2	Cdk2	9.27E-03	-1.552
ILMN_2617499	NM_025900	DEK oncogene (DNA binding) (Dek), mRNA. (S)	Dek	DEK oncogene (DNA binding)	Dek	2.04E-02	-1.550
ILMN_1237163	NM_010620	kinesin family member 15 (Kif15), mRNA. (S)	Kif15	kinesin family member 15	KIF15	4.31E-02	-1.543
ILMN_2526866	XR_030716	PREDICTED: similar to protein phosphatase 2A inhibitor-2 I-2PP2A (LOC544884), misc RNA. (S)	LOC544884	predicted gene, EG625349; predicted gene 5789; predicted gene 7085; predicted gene 5708; predicted gene 6847; SET translocation; cDNA sequence BC085271; predicted gene 7239; similar to protein phosphatase 2A inhibitor-2 I-2PP2A; predicted gene 9531	Gm5789	2.53E-03	-1.542
ILMN_1238074	XM_001478079	PREDICTED: hypothetical protein LOC100047402 (LOC100047402), mRNA. (S)	LOC100047402	n/a	n/a	1.01E-02	-1.541
ILMN_2960308	NM_008303	heat shock protein 1 (chaperonin 10) (Hspe1), mRNA. (S)	Hspe1	heat shock protein 1 (chaperonin 10); predicted gene, EG628438; heat shock protein 1 (chaperonin 10), related sequence 1; predicted gene 2903	Gm2903	9.86E-03	-1.540
ILMN_1225085	NM_008855	protein kinase C, beta (Prkcb), mRNA. (S)	Prkcb	protein kinase C, beta	PRKCB	1.50E-02	-1.540
ILMN_1227914	XM_990977	PREDICTED: hypothetical LOC667337 (LOC667337), mRNA. (S)	LOC667337	n/a	n/a	5.97E-03	-1.536
ILMN_2754092	NM_175664	histone cluster 1, H2bb (Hist1h2bb), mRNA. (S)	Hist1h2bb	histone cluster 1, H2bb	Hist1h2bb	8.17E-03	-1.534

ILMN_2695008	NM_019744	NaN (S)	Ncoa4	predicted gene 6768; nuclear receptor coactivator 4	NCOA4	9.10E-04	-1.534
ILMN_1247066	NM_016739	cell cycle associated protein 1 (Caprin1), mRNA. (S)	Caprin1	cell cycle associated protein 1	Caprin1	2.70E-02	-1.533
ILMN_2757197	NM_028597	THO complex 3 (Thoc3), mRNA. (S)	Thoc3	THO complex 3	THOC3	4.99E-02	-1.533
ILMN_2660386	NM_183017	NaN (S)	BC055368	tubulin tyrosine ligase-like family, member 12	TTLL12	4.02E-02	-1.532
ILMN_2525605	XR_035278	PREDICTED: predicted gene, EG667728 (EG667728), misc RNA. (S)	EG667728	histone cluster 1, H2al	HIST1H2AL	3.13E-02	-1.532
ILMN_2628629	NM_009864	cadherin 1 (Cdh1), mRNA. (S)	Cdh1	cadherin 1	CDH1	4.15E-02	-1.532
ILMN_2688607	NM_177242	PTC7 protein phosphatase homolog (S. cerevisiae) (Pptc7), mRNA. (S)	Pptc7	PTC7 protein phosphatase homolog (S. cerevisiae)	Pptc7	2.00E-02	-1.531
ILMN_2596761	NM_025364	RIKEN cDNA 1110005A23 gene (1110005A23Rik), mRNA. (S)	1110005A23Rik	predicted gene 6563; SAP domain containing ribonucleoprotein	SARNP	3.11E-02	-1.530
ILMN_1215332	XM_122761	NaN (S)	LOC219145	n/a	n/a	6.81E-04	-1.529
ILMN_2677541	NM_177657	RIKEN cDNA D630003M21 gene (D630003M21Rik), transcript variant 1, mRNA. (S)	D630003M21Rik	RIKEN cDNA D630003M21 gene	D630003M21Rik	1.46E-02	-1.528
ILMN_2766408	NM_153533	tensin like C1 domain-containing phosphatase (Tenc1), mRNA. (S)	Tenc1	tensin like C1 domain-containing phosphatase	TENC1	1.84E-03	-1.527
ILMN_2528456	XM_139078	NaN (S)	LOC219106	n/a	n/a	4.83E-05	-1.525
ILMN_2729513	NM_016956	hemoglobin, beta adult minor chain (Hbb-b2), mRNA. (S)	Hbb-b2	hemoglobin, beta adult major chain; hemoglobin, beta adult minor chain	Hbb-b2	4.12E-02	-1.523
ILMN_2590884	NM_011113	plasminogen activator, urokinase receptor (Plaur), mRNA. (S)	Plaur	plasminogen activator, urokinase receptor	PLAUR	4.07E-02	-1.520
ILMN_1258515	NM_026420	polyadenylate-binding protein-interacting protein 2 (Paip2), mRNA. (S)	Paip2	polyadenylate-binding protein-interacting protein 2	Paip2	3.34E-02	-1.517
ILMN_1254898	AK036390	NaN (S)	9830004G04Rik	WD repeat and HMG-box DNA binding protein 1	Wdhd1	3.81E-02	-1.516

ILMN_2751761	NM_175294	nuclear casein kinase and cyclin-dependent kinase substrate 1 (Nucks1), mRNA. (S)	Nucks1	nuclear casein kinase and cyclin-dependent kinase substrate 1	Nucks1	1.75E-03	-1.514
ILMN_2633819	NM_133825	DNA segment, Chr 1, ERATO Doi 622, expressed (D1Ertd622e), mRNA. (S)	D1Ertd622e	DNA segment, Chr 1, ERATO Doi 622, expressed	D1Ertd622e	2.44E-04	-1.513
ILMN_2685507	NM_009672	acidic (leucine-rich) nuclear phosphoprotein 32 family, member A (Anp32a), mRNA. (S)	Anp32a	acidic (leucine-rich) nuclear phosphoprotein 32 family, member A	ANP32A	1.04E-02	-1.513
ILMN_2732689	NM_019710	structural maintenance of chromosomes 1A (Smc1a), mRNA. (S)	Smc1a	structural maintenance of chromosomes 1A	Smc1a	4.73E-02	-1.513
ILMN_1245504	XR_031764	PREDICTED: similar to high-mobility group box 1 (LOC100040413), misc RNA. (S)	LOC100040413	predicted gene 13121; predicted gene 3160; high-mobility group (nonhistone chromosomal) protein 1-like 1; predicted gene 6090; predicted gene 3851; predicted gene 8967; predicted gene 7782; predicted gene 4587; predicted gene 4689; predicted gene 3307; predicted gene 13932; predicted gene 15059; predicted gene 3565; predicted gene 15447; predicted gene 12587; predicted gene 9012; predicted gene 6115; predicted gene 9480; high mobility group box 1; predicted gene 8423; predicted gene 5853; predicted gene 8288; predicted gene 7888; predicted gene 8594; predicted gene 15387; predicted gene 5473; predicted gene 8807; similar to high mobility group box 1; similar to 2810416G20Rik protein; predicted gene 8390; predicted gene, OTTMUSG00000005439; predicted gene 5842; predicted gene 5527; predicted gene 8563; predicted gene 2710; predicted gene 12331; predicted gene 5937; predicted gene 5504; similar to high-mobility group box 1; predicted gene 10361; predicted gene 2607; predicted gene 7422; predicted gene 10075; predicted gene 12568; predicted gene 6589; predicted gene 4383; predicted gene 8031; similar to High mobility group protein 1 (HMG-1) (High mobility group protein B1) (Amphoterin) (Heparin-binding protein p30); predicted gene 7468; predicted gene 8554	LOC674543	1.30E-02	-1.511

ILMN_1258369	NM_001098227	syndecan binding protein (Sdcbp), transcript variant 1, mRNA. (S)	Sdcbp	similar to syntenin; syndecan binding protein	LOC100047309	3.11E-04	-1.511
ILMN_2867899	NM_023223	cell division cycle 20 homolog (S. cerevisiae) (Cdc20), mRNA. (S)	Cdc20	cell division cycle 20 homolog (S. cerevisiae)	cdc20	1.15E-02	-1.511
ILMN_2659182	NM_019434	minichromosome maintenance deficient 3 (S. cerevisiae) associated protein (Mcm3ap), mRNA. (S)	Mcm3ap	minichromosome maintenance deficient 3 (S. cerevisiae) associated protein	MCM3AP	1.12E-02	-1.511
ILMN_3123441	NM_001081019	predicted gene, OTTMUSG00000007855 (OTTMUSG00000007855), mRNA. (A)	OTTMUSG00000007855	predicted gene 14383; predicted gene 3835; predicted gene 14384; predicted gene 12950; predicted gene, 670915; H3 histone, family 3A; predicted gene 12657; predicted gene 6132; predicted gene 10257; predicted gene 7227; H3 histone, family 3B; predicted gene 6128; similar to histone; predicted gene 1986; predicted gene 6186; hypothetical protein LOC676337; predicted gene 6421; predicted gene 2198; predicted gene 6817; predicted gene 8095; predicted gene 12271; predicted gene 13529; predicted gene 8029; predicted gene 4938; predicted gene 7100; predicted gene 9014; similar to Histone H3.4 (Embryonic); predicted gene 7179; similar to H3 histone, family 3B; predicted gene 7900; predicted gene 2099; similar to H3 histone, family 3A; predicted gene 6749; predicted gene 6485; predicted gene 4028; predicted gene 7194	Gm3835	8.04E-03	-1.510
ILMN_2420095	NM_024189	YY1 associated factor 2 (Yaf2), mRNA. (S)	Yaf2	YY1 associated factor 2	YAF2	1.69E-02	-1.509
ILMN_1213257	NM_178193	histone cluster 1, H4b (Hist1h4b), mRNA. (S)	Hist1h4b	histone cluster 1, H4k; histone cluster 1, H4m; histone cluster 4, H4; similar to germinal histone H4 gene; histone cluster 1, H4h; histone cluster 1, H4j; histone cluster 1, H4i; histone cluster 1, H4d; histone cluster 1, H4c; histone cluster 1, H4f; histone cluster 1, H4b; histone cluster 1, H4a; histone cluster 2, H4; similar to histone H4	Hist1h4c	3.89E-02	-1.505
ILMN_1252263	AK017907	NaN (S)	Uqcrb	ubiquinol-cytochrome c reductase, complex III subunit VII	UQCRQ	6.49E-03	-1.503
ILMN_1232121	NM_015753	zinc finger E-box binding homeobox 2 (Zeb2), transcript variant 2, mRNA. (S)	Zeb2	zinc finger E-box binding homeobox 2	Zeb2	1.51E-02	-1.503

ILMN_1215011	NM_130889	acidic (leucine-rich) nuclear phosphoprotein 32 family, member B (Anp32b), mRNA. (S)	Anp32b	acidic (leucine-rich) nuclear phosphoprotein 32 family, member B	anp32b	1.21E-02	-1.502
ILMN_2720451	NM_010066	DNA methyltransferase (cytosine-5) 1 (Dnmt1), mRNA. (S)	Dnmt1	DNA methyltransferase (cytosine-5) 1	dnmt1	1.47E-04	-1.500
ILMN_2586158	AK089798	NaN (S)	F830022O08Rik	n/a	n/a	3.01E-02	-1.496
ILMN_2459155	NM_013915	zinc finger protein 238 (Zfp238), transcript variant 2, mRNA. (S)	Zfp238	zinc finger protein 238	Zfp238	2.68E-03	-1.495
ILMN_1220581	NM_024183	FIP1 like 1 (S. cerevisiae) (Fip1l1), mRNA. (S)	Fip1l1	FIP1 like 1 (S. cerevisiae)	Fip1l1	4.12E-03	-1.495
ILMN_2756008	NM_016777	NaN (S)	Nasp	nuclear autoantigenic sperm protein (histone-binding); similar to nuclear autoantigenic sperm protein; NASP	NASP	5.60E-03	-1.495
ILMN_2497581	NM_010931	ubiquitin-like, containing PHD and RING finger domains, 1 (Uhrf1), mRNA. (S)	Uhrf1	ubiquitin-like, containing PHD and RING finger domains, 1; predicted gene 5648; similar to nuclear zinc finger protein Np95	UHRF1	8.05E-03	-1.494
ILMN_2767615	NM_009721	ATPase, Na+/K+ transporting, beta 1 polypeptide (Atp1b1), mRNA. (S)	Atp1b1	ATPase, Na+/K+ transporting, beta 1 polypeptide	Atp1b1	1.31E-02	-1.493
ILMN_3022428	NM_001080129	thymopoietin (Tmpo), transcript variant 3, mRNA. (I)	Tmpo	thymopoietin	TMPO	2.11E-02	-1.491
ILMN_2598852	NM_011239	RAN binding protein 1 (Ranbp1), mRNA. (S)	Ranbp1	RAN binding protein 1	RanBP1	2.28E-02	-1.490
ILMN_1217622	NM_009019	recombination activating gene 1 (Rag1), mRNA. (S)	Rag1	recombination activating gene 1	RAG1	1.33E-03	-1.486
ILMN_3003290	NM_008231	hepatoma-derived growth factor (Hdgf), mRNA. (S)	Hdgf	hepatoma-derived growth factor	hdgf	1.84E-02	-1.484
ILMN_2534259	XM_111221	NaN (S)	LOC237877	n/a	n/a	1.15E-02	-1.482
ILMN_2612477	NM_019869	RNA binding motif protein 14 (Rbm14), mRNA. (S)	Rbm14	RNA binding motif protein 14	RBM14	2.31E-02	-1.481
ILMN_2773496	NM_130863	NaN (S)	Adrbk1	adrenergic receptor kinase, beta 1	ADRBK1	4.09E-02	-1.481
ILMN_2691187	NM_009031	retinoblastoma binding protein 7 (Rbbp7), mRNA. (S)	Rbbp7	retinoblastoma binding protein 7; predicted gene 6382	Gm6382	2.75E-02	-1.477
ILMN_2540183	XM_358717	NaN (S)	LOC382229	n/a	n/a	3.59E-02	-1.477
ILMN_1256883	NM_011234	RAD51 homolog (S. cerevisiae) (Rad51), mRNA. (S)	Rad51	RAD51 homolog (S. cerevisiae)	rad51	1.95E-02	-1.476

ILMN_2962632	NM_016755	ATP synthase, H+ transporting, mitochondrial F0 complex, subunit F (Atp5j), nuclear gene encoding mitochondrial protein, mRNA. (S)	Atp5j	ATP synthase, H+ transporting, mitochondrial F0 complex, subunit F pseudogene; similar to ATP synthase coupling factor 6, mitochondrial precursor (ATPase subunit F6); ATP synthase, H+ transporting, mitochondrial F0 complex, subunit F	LOC674583	2.93E-02	-1.475
ILMN_1251097	XM_884529	PREDICTED: predicted gene, EG620143 (EG620143), mRNA. (S)	EG620143	predicted gene 14383; predicted gene 3835; predicted gene 14384; predicted gene 12950; predicted gene, 670915; H3 histone, family 3A; predicted gene 12657; predicted gene 6132; predicted gene 10257; predicted gene 7227; H3 histone, family 3B; predicted gene 6128; similar to histone; predicted gene 1986; predicted gene 6186; hypothetical protein LOC676337; predicted gene 6421; predicted gene 2198; predicted gene 6817; predicted gene 8095; predicted gene 12271; predicted gene 13529; predicted gene 8029; predicted gene 4938; predicted gene 7100; predicted gene 9014; similar to Histone H3.4 (Embryonic); predicted gene 7179; similar to H3 histone, family 3B; predicted gene 7900; predicted gene 2099; similar to H3 histone, family 3A; predicted gene 6749; predicted gene 6485; predicted gene 4028; predicted gene 7194	Gm3835	3.62E-02	-1.474
ILMN_2543393	NM_026560	cell division cycle associated 8 (Cdca8), mRNA. (S)	Cdca8	cell division cycle associated 8	CDCA8	3.23E-02	-1.470
ILMN_2613841	NM_029157	splicing factor 3a, subunit 3 (Sf3a3), mRNA. XM_983572 XM_992567 XM_992590 XM_992606 (S)	Sf3a3	splicing factor 3a, subunit 3	sf3a3	2.70E-03	-1.470
ILMN_1213524	NM_197982	DEAD (Asp-Glu-Ala-Asp) box polypeptide 39 (Ddx39), mRNA. (S)	Ddx39	DEAD (Asp-Glu-Ala-Asp) box polypeptide 39	ddx39	1.87E-03	-1.468
ILMN_1222366	NM_144731	UDP-N-acetyl-alpha-D-galactosamine: polypeptide N-acetylgalactosaminyltransferase 7 (Galnt7), mRNA. (S)	Galnt7	UDP-N-acetyl-alpha-D-galactosamine: polypeptide N-acetylgalactosaminyltransferase 7	Galnt7	2.07E-02	-1.468
ILMN_3149944	NM_001037841	chemokine-like factor (Cklf), transcript variant 4, mRNA. (A)	Cklf	chemokine-like factor	Cklf	4.97E-02	-1.467
ILMN_1228585	NM_010876	neutrophil cytosolic factor 1 (Ncf1), mRNA. (S)	Ncf1	neutrophil cytosolic factor 1	ncf1	3.16E-02	-1.466

ILMN_1227164	NM_011655	tubulin, beta 5 (Tubb5), mRNA. (S)	Tubb5	tubulin, beta 5	Tubb5	1.11E-02	-1.466
ILMN_1234909	NM_025372	timeless interacting protein (Tipin), mRNA. (S)	Tipin	timeless interacting protein	TIPIN	3.76E-02	-1.465
ILMN_2719803	NM_019992	signal transducing adaptor family member 1 (Stap1), mRNA. (S)	Stap1	signal transducing adaptor family member 1; similar to stem cell adaptor protein STAP-1	STAP1	3.51E-02	-1.461
ILMN_2878071	NM_013590	lysozyme (Lyz), mRNA. (S)	Lyz	lysozyme 1	Lyz1	2.23E-02	-1.461
ILMN_2673369	NM_008320	interferon regulatory factor 8 (Irf8), mRNA. (S)	Irf8	interferon regulatory factor 8	IRF8	1.41E-02	-1.460
ILMN_2896797	NM_013522	FSHD region gene 1 (Frg1), mRNA. (S)	Frg1	FSHD region gene 1	frg1	3.45E-02	-1.458
ILMN_2980331	NM_133354	SMT3 suppressor of mif two 3 homolog 2 (yeast) (Sumo2), mRNA. (S)	Sumo2	predicted gene 11829; SMT3 suppressor of mif two 3 homolog 2 (yeast); predicted gene 12335; similar to Chain B, Crystal Structure Of Sumo-3-Modified Thymine-Dna Glycosylase; similar to SMT3 suppressor of mif two 3 homolog 2; predicted gene 13430; predicted gene 4905; hypothetical protein LOC100044181; predicted gene 10540; predicted gene 10241; similar to SMT3B protein; predicted gene 9836; predicted gene 12755; predicted gene 13690; predicted gene 11971	Gm10540	1.86E-02	-1.455
ILMN_2949596	NM_009955	dihydropyrimidinase-like 2 (Dpysl2), mRNA. (S)	Dpysl2	dihydropyrimidinase-like 2	Dpysl2	5.33E-03	-1.453
ILMN_1217331	NM_008567	minichromosome maintenance deficient 6 (MIS5 homolog, S. pombe) (S. cerevisiae) (Mcm6), mRNA. (S)	Mcm6	minichromosome maintenance deficient 6 (MIS5 homolog, S. pombe) (S. cerevisiae)	MCM6	4.94E-05	-1.452
ILMN_1245076	AK042925	NaN (S)	A730040I05Rik	early B-cell factor 1	EBF1	3.13E-02	-1.452
ILMN_1259041	XR_001767	PREDICTED: predicted gene, EG632352 (EG632352), misc RNA. (S)	EG632352	predicted gene, EG625349; predicted gene 5789; predicted gene 7085; predicted gene 5708; predicted gene 6847; SET translocation; cDNA sequence BC085271; predicted gene 7239; similar to protein phosphatase 2A inhibitor-2 I-2PP2A; predicted gene 9531	Gm5789	2.46E-02	-1.451
ILMN_2771260	NM_001077267	heterogeneous nuclear ribonucleoprotein D (Hnrnpd), transcript variant 4, mRNA. (S)	Hnrnpd	heterogeneous nuclear ribonucleoprotein D	HNRNPD	1.90E-02	-1.446

ILMN_1223699	XM_144101	NaN (S)	LOC242703	n/a	n/a	1.99E-02	-1.446
ILMN_1254883	NM_024183	FIP1 like 1 (S. cerevisiae) (Fip1l1), mRNA. (S)	Fip1l1	FIP1 like 1 (S. cerevisiae)	Fip1l1	1.47E-02	-1.445
ILMN_1218677	XM_917905	PREDICTED: WD repeat domain 43, transcript variant 9 (Wdr43), mRNA. (S)	Wdr43	WD repeat domain 43; RIKEN cDNA 2610029G23 gene; hypothetical protein LOC674157	LOC674157	2.44E-02	-1.443
ILMN_1234324	NM_001033222	PDZ domain containing 8 (Pdzd8), mRNA. (S)	Pdzd8	PDZ domain containing 8	PDZD8	3.22E-02	-1.442
ILMN_2768325	NM_025415	CDC28 protein kinase regulatory subunit 2 (Cks2), mRNA. (S)	Cks2	similar to Cyclin-dependent kinases regulatory subunit 2 (CKS-2); CDC28 protein kinase regulatory subunit 2; predicted gene 15452	LOC100044750	2.47E-02	-1.437
ILMN_2754425	NM_152810	cell division cycle 5-like (S. pombe) (Cdc5l), mRNA. (S)	Cdc5l	predicted gene 9040; predicted gene 9046; predicted gene 9029; predicted gene 9048; cell division cycle 5-like (S. pombe); predicted gene 9049; predicted gene 9044; predicted gene 9030; predicted gene 9045	Gm9040	6.77E-04	-1.437
ILMN_3006534	NM_198609	cDNA sequence BC003885 (BC003885), mRNA. (S)	BC003885	predicted gene 6457; cDNA sequence BC003885	Gm6457	1.19E-03	-1.436
ILMN_2709839	NM_010772	MYC-associated zinc finger protein (purine-binding transcription factor) (Maz), mRNA. (S)	Maz	MYC-associated zinc finger protein (purine-binding transcription factor)	MAZ	1.50E-02	-1.434
ILMN_2774825	NM_013716	Ras-GTPase-activating protein SH3-domain binding protein 1 (G3bp1), mRNA. (S)	G3bp1	Ras-GTPase-activating protein SH3-domain binding protein 1	G3BP1	2.57E-02	-1.433
ILMN_3097131	NM_011589	timeless homolog (Drosophila) (Timeless), transcript variant 2, mRNA. (A)	Timeless	timeless homolog (Drosophila)	TIMELESS	2.38E-02	-1.432
ILMN_2730329	NM_175659	histone cluster 1, H2ah (Hist1h2ah), mRNA. (S)	Hist1h2ah	histone cluster 1, H2ad; histone cluster 1, H2ae; histone cluster 1, H2ag; histone cluster 1, H2ah; histone cluster 1, H2ai; similar to histone 2a; histone cluster 1, H2an; histone cluster 1, H2ao; histone cluster 1, H2ac; histone cluster 1, H2ab	Hist1h2ab	4.80E-02	-1.430
ILMN_2774941	NM_009838	NaN (S)	Cct6a	chaperonin containing Tcp1, subunit 6a (zeta)	cct6a	2.40E-03	-1.429
ILMN_2745073	XM_001477963	PREDICTED: similar to myocardial vascular inhibition factor (LOC100047353), mRNA. (S)	LOC100047353	B-cell translocation gene 1, anti-proliferative; similar to myocardial vascular inhibition factor	LOC100047353	8.97E-03	-1.429

ILMN_3145331	NM_025284	thymosin, beta 10 (Tmsb10), mRNA. (A)	Tmsb10	predicted gene 3787; predicted gene 9844; predicted gene 8034; similar to thymosin, beta 10; thymosin, beta 10	LOC100048142	3.81E-02	-1.428
ILMN_1237868	NM_009846	CD24a antigen (Cd24a), mRNA. (S)	Cd24a	CD24a antigen	Cd24a	2.10E-04	-1.425
ILMN_2629052	NM_007624	NaN (S)	Cbx3	predicted gene 6917; similar to chromobox homolog 3; predicted gene 5792; predicted gene 7469; predicted gene 6901; predicted gene 7721; predicted gene 5196; complement component 7; chromobox homolog 3 (Drosophila HP1 gamma)	C7	1.71E-02	-1.421
ILMN_2575811	AK047599	NaN (S)	B930096N04Rik	nucleolar protein 9	NOL9	5.67E-03	-1.421
ILMN_2609615	NM_025875	RNA binding motif protein 8a (Rbm8a), mRNA. (S)	Rbm8a	RNA binding motif protein 8a; RIKEN cDNA B020018G12 gene	RBM8A	4.60E-02	-1.420
ILMN_2749583	NM_025995	F-box protein 5 (Fbxo5), mRNA. (S)	Fbxo5	F-box protein 5	Fbxo5	3.23E-02	-1.418
ILMN_1259528	XM_356174	NaN (S)	LOC382092	n/a	n/a	2.62E-03	-1.418
ILMN_1242935	XM_921371	PREDICTED: similar to Chromobox homolog 3 (HP1 gamma homolog, Drosophila), transcript variant 2 (LOC633016), mRNA. (S)	LOC633016	predicted gene 6917; similar to chromobox homolog 3; predicted gene 5792; predicted gene 7469; predicted gene 6901; predicted gene 7721; predicted gene 5196; complement component 7; chromobox homolog 3 (Drosophila HP1 gamma)	C7	2.35E-02	-1.417
ILMN_1221192	XM_915908	PREDICTED: WD repeat domain 7, transcript variant 5 (Wdr7), mRNA. (S)	Wdr7	WD repeat domain 7	WDR7	2.37E-02	-1.416
ILMN_2774140	NM_013686	t-complex protein 1 (Tcp1), mRNA. (S)	Tcp1	t-complex protein 1	TCP1	4.13E-02	-1.416
ILMN_2665516	NM_023871	SET translocation (Set), mRNA. (S)	Set	predicted gene, EG625349; predicted gene 5789; predicted gene 7085; predicted gene 5708; predicted gene 6847; SET translocation; cDNA sequence BC085271; predicted gene 7239; similar to protein phosphatase 2A inhibitor-2 I-2PP2A; predicted gene 9531	Gm5789	3.05E-02	-1.415
ILMN_2538208	XM_359080	NaN (S)	LOC386124	n/a	n/a	3.68E-02	-1.414
ILMN_3029953	NM_145556	TAR DNA binding protein (Tardbp), transcript variant 1, mRNA. (I)	Tardbp	predicted gene 13886; TAR DNA binding protein	TARDBP	1.70E-02	-1.409

ILMN_3084883	NR_002883	predicted gene, EG434858 (EG434858), non-coding RNA. (A)	EG434858	heterogeneous nuclear ribonucleoprotein A1-like 2; similar to heterogeneous nuclear ribonucleoprotein A1; predicted gene 13418; predicted gene 5461; predicted gene 8652; predicted gene 5643; similar to Heterogeneous nuclear ribonucleoprotein A1 (Helix-destabilizing protein) (Single-strand RNA-binding protein) (hnRNP core protein A1) (HDP); heterogeneous nuclear ribonucleoprotein A1; predicted gene 6296	HNRNPA1L2	1.74E-02	-1.407
ILMN_2419650	NM_015753	zinc finger E-box binding homeobox 2 (Zeb2), transcript variant 2, mRNA. (S)	Zeb2	zinc finger E-box binding homeobox 2	Zeb2	2.99E-02	-1.407
ILMN_2727618	NM_009227	small nuclear ribonucleoprotein E (Snrpe), mRNA. (S)	Snrpe	small nuclear ribonucleoprotein E; predicted gene 6487	Gm6487	2.74E-03	-1.407
ILMN_1233606	NM_027324	sideroflexin 1 (Sfxn1), mRNA. (S)	Sfxn1	sideroflexin 1	SFXN1	1.16E-02	-1.404
ILMN_2735438	NM_027432	WD repeat domain 77 (Wdr77), mRNA. (S)	Wdr77	WD repeat domain 77; predicted gene 15528	WDR77	1.32E-02	-1.402
ILMN_2685806	NM_011793	barrier to autointegration factor 1 (Banf1), transcript variant 1, mRNA. (S)	Banf1	barrier to autointegration factor 1	BANF1	3.87E-02	-1.402
ILMN_2699003	NM_013929	SIVA1, apoptosis-inducing factor (Siva1), mRNA. (S)	Siva1	SIVA1, apoptosis-inducing factor pseudogene; similar to CD27-binding (Siva) protein isoform 2; SIVA1, apoptosis-inducing factor	SIVA1	3.85E-02	-1.401
ILMN_1239021	NM_029804	heterogeneous nuclear ribonucleoprotein M (Hnrpm), mRNA. (S)	Hnrpm	heterogeneous nuclear ribonucleoprotein M	hnrnpm	3.64E-04	-1.401
ILMN_2661412	NM_029617	cancer susceptibility candidate 5 (Casc5), mRNA. (S)	Casc5	cancer susceptibility candidate 5	CASC5	4.14E-02	-1.400
ILMN_1230201	AK048863	NaN (S)	C230077B03Rik	n/a	n/a	3.08E-02	-1.399
ILMN_2423789	NM_019748	SUMO1 activating enzyme subunit 1 (Sae1), mRNA. (S)	Sae1	SUMO1 activating enzyme subunit 1	SAE1	4.26E-03	-1.395
ILMN_3136638	NM_009221	synuclein, alpha (Snca), transcript variant 2, mRNA. (A)	Snca	synuclein, alpha	Snca	2.17E-02	-1.394
ILMN_2682522	NM_133729	RIKEN cDNA 2610018G03 gene (2610018G03Rik), mRNA. (S)	2610018G03Rik	RIKEN cDNA 2610018G03 gene	2610018G03Rik	1.17E-02	-1.394

ILMN_3014419	NM_001081092	TAF4A RNA polymerase II, TATA box binding protein (TBP)-associated factor (Taf4a), mRNA. (I)	Taf4a	TAF4A RNA polymerase II, TATA box binding protein (TBP)-associated factor; similar to TAF4A RNA polymerase II, TATA box binding protein (TBP)-associated factor	LOC100046932	2.04E-02	-1.393
ILMN_2918987	NM_024471	lipoic acid synthetase (Lias), mRNA. (S)	Lias	lipoic acid synthetase	LIAS	4.91E-02	-1.391
ILMN_2785454	NM_178213	histone cluster 2, H2ab (Hist2h2ab), mRNA. (S)	Hist2h2ab	histone cluster 2, H2ab	HIST2H2AB	6.73E-03	-1.391
ILMN_1229971	NM_016686	vascular endothelial zinc finger 1 (Vezf1), mRNA. (S)	Vezf1	vascular endothelial zinc finger 1	vezf1	2.25E-02	-1.388
ILMN_1250117	NM_018785	NaN (S)	Fnbp3	PRP40 pre-mRNA processing factor 40 homolog A (yeast)	Prpf40a	2.40E-02	-1.386
ILMN_1238368	XM_358166	NaN (S)	LOC385279	n/a	n/a	1.33E-02	-1.386
ILMN_2921095	NM_029804	heterogeneous nuclear ribonucleoprotein M (Hnrpm), mRNA. (S)	Hnrpm	heterogeneous nuclear ribonucleoprotein M	hnrnpm	1.82E-03	-1.385
ILMN_2591814	NM_173374	splicing factor, arginine/serine-rich 1 (ASF/SF2) (Sfrs1), transcript variant 1, mRNA. (S)	Sfrs1	splicing factor, arginine/serine-rich 1 (ASF/SF2); similar to splicing factor, arginine/serine-rich 1 (splicing factor 2, alternate splicing factor)	SFRS1	1.52E-02	-1.384
ILMN_1212636	NM_011499	serine/threonine kinase receptor associated protein (Strap), mRNA. (S)	Strap	serine/threonine kinase receptor associated protein	strap	2.53E-02	-1.381
ILMN_1216697	XM_001006885	PREDICTED: similar to A-kinase anchor protein 5 (LOC674008), mRNA. (S)	LOC674008	n/a	n/a	1.22E-02	-1.380
ILMN_1244135	NM_026369	actin related protein 2/3 complex, subunit 5 (Arpc5), mRNA. (S)	Arpc5	predicted gene 16372; actin related protein 2/3 complex, subunit 5	ARPC5	4.54E-03	-1.380
ILMN_2513173	NaN	NaN (S)	2810026P18Rik	n/a	n/a	2.41E-02	-1.379
ILMN_1233989	NM_016904	CDC28 protein kinase 1b (Cks1b), mRNA. (S)	Cks1b	predicted gene 10124; predicted gene 6340; CDC28 protein kinase 1b	Gm6340	2.82E-02	-1.378
ILMN_1218504	NM_172264	choline dehydrogenase (Chdh), mRNA. (S)	Chdh	choline dehydrogenase	chdh	3.88E-02	-1.378
ILMN_1219716	XR_033256	PREDICTED: similar to ENSANGP00000012700 (LOC665672), misc RNA. (S)	LOC665672	predicted gene 7743; calmodulin 3; calmodulin 2; calmodulin 1; predicted gene 7308	Gm7308	5.79E-04	-1.377

ILMN_2537961	XM_358862	NaN (S)	LOC385659	n/a	n/a	2.14E-02	-1.377
ILMN_2832808	NM_011694	voltage-dependent anion channel 1 (Vdac1), mRNA. (S)	Vdac1	voltage-dependent anion channel 1	VDAC1	4.87E-02	-1.376
ILMN_2698589	NM_133196	cleavage stimulation factor, 3' pre-RNA subunit 2 (Cstf2), mRNA. (S)	Cstf2	cleavage stimulation factor, 3' pre-RNA subunit 2	cstf2	1.95E-02	-1.375
ILMN_2970473	NM_008722	nucleophosmin 1 (Npm1), mRNA. (S)	Npm1	predicted gene 6477; predicted gene 9118; nucleophosmin 1; similar to Nucleophosmin (NPM) (Nucleolar phosphoprotein B23) (Numatrin) (Nucleolar protein NO38); predicted gene 7289; predicted gene 5611	LOC100046628	1.49E-02	-1.373
ILMN_2624622	NM_031185	A kinase (PRKA) anchor protein (gravin) 12 (Akap12), mRNA. (S)	Akap12	A kinase (PRKA) anchor protein (gravin) 12	AKAP12	3.03E-03	-1.371
ILMN_2444316	NM_022314	tropomyosin 3, gamma (Tpm3), mRNA. (S)	Tpm3	predicted gene 7848; predicted gene 7839; predicted gene 4157; similar to tropomyosin 3, gamma; tropomyosin 3, gamma; predicted gene 4903	Gm7839	2.08E-02	-1.369
ILMN_2442516	NM_080561	ring finger protein 216 (Rnf216), mRNA. (S)	Rnf216	ring finger protein 216	rnf216	4.73E-02	-1.369
ILMN_1234231	XM_356518	NaN (S)	LOC382461	n/a	n/a	4.10E-04	-1.368
ILMN_1220514	XM_358357	NaN (S)	9030416H16Rik	n/a	n/a	4.37E-02	-1.366
ILMN_2862111	NM_016957	high mobility group nucleosomal binding domain 2 (Hmgn2), mRNA. (S)	Hmgn2	high mobility group nucleosomal binding domain 4; predicted gene 7931; predicted gene 10282; predicted gene 3338; high mobility group nucleosomal binding domain 2; predicted gene 6594; predicted gene 6750; predicted gene 10182; predicted gene 6724; predicted gene 4248; predicted gene 9525; predicted gene 15296; similar to Hmgn2 protein; predicted gene 7125; hypothetical protein LOC638323; predicted gene 6651; predicted gene 5899; predicted gene 14008; predicted gene 16494; similar to high mobility group nucleosomal binding domain 2	Gm10182	1.76E-02	-1.365
ILMN_1221723	AK015874	NaN (S)	Siat8c	ST8 alpha-N-acetyl-neuraminide alpha-2,8-sialyltransferase 3	ST8SIA3	2.47E-02	-1.365
ILMN_1255779	NM_018775	TBC1 domain family, member 8 (Tbc1d8), mRNA. (S)	Tbc1d8	TBC1 domain family, member 8	TBC1D8	3.45E-02	-1.365

ILMN_2775885	NM_007589	NaN (S)	Calm2	predicted gene 7743; calmodulin 3; calmodulin 2; calmodulin 1; predicted gene 7308	Gm7308	8.82E-05	-1.364
ILMN_1221606	AK049601	NaN (S)	Rrm1	ribonucleotide reductase M1	Rrm1	4.79E-02	-1.364
ILMN_1238284	NM_001030307	dyskeratosis congenita 1, dyskerin homolog (human) (Dkc1), mRNA. (S)	Dkc1	dyskeratosis congenita 1, dyskerin homolog (human)	DKC1	3.31E-02	-1.364
ILMN_1213625	NM_001080130	thymopoietin (Tmpo), transcript variant 4, mRNA. (S)	Tmpo	thymopoietin	TMPO	4.47E-02	-1.364
ILMN_1249762	XM_137113	NaN (S)	LOC211870	n/a	n/a	1.45E-02	-1.363
ILMN_2708776	NM_025279	heterogeneous nuclear ribonucleoprotein K (Hnrnpk), mRNA. (S)	Hnrnpk	heterogeneous nuclear ribonucleoprotein K; predicted gene 7964	Gm7964	9.46E-03	-1.363
ILMN_1213167	NM_013507	NaN (S)	Eif4g2	eukaryotic translation initiation factor 4, gamma 2	EIF4G2	4.59E-02	-1.363
ILMN_1237890	XM_357322	NaN (S)	LOC383916	n/a	n/a	1.08E-03	-1.362
ILMN_2521356	NM_021278	NaN (S)	Tmsb4x	thymosin, beta 4, X chromosome; similar to thymosin beta-4	LOC100047211	1.08E-02	-1.361
ILMN_1245742	AK010648	NaN (S)	2410041F14Rik	minichromosome maintenance deficient 10 (S. cerevisiae)	Mcm10	2.43E-02	-1.360
ILMN_2626779	NM_023144	non-POU-domain-containing, octamer binding protein (Nono), mRNA. (S)	Nono	non-POU-domain-containing, octamer binding protein; predicted gene 8806	nonO	3.45E-03	-1.359
ILMN_2622163	NM_010256	phosphoribosylglycinamide formyltransferase (Gart), mRNA. (S)	Gart	phosphoribosylglycinamide formyltransferase	GART	2.87E-02	-1.356
ILMN_2930843	NM_013823	klotho (Kl), mRNA. (S)	Kl	klotho	KL	1.33E-02	-1.355
ILMN_2570776	AK050697	NaN (S)	D030005C18Rik	n/a	n/a	4.74E-02	-1.353
ILMN_1228832	NM_008694	neutrophilic granule protein (Ngp), mRNA. (S)	Ngp	neutrophilic granule protein	Ngp	6.67E-03	-1.352

ILMN_2680850	NR_002840	growth arrest specific 5 (Gas5), non-coding RNA. XR_000433 XR_000434 XR_000435 XR_000436 XR_000472 XR_000473 XR_000474 XR_000475 XR_000476 (S)	Gas5	growth arrest specific 5	GAS5	4.86E-03	-1.352
ILMN_2900557	NM_010620	kinesin family member 15 (Kif15), mRNA. (S)	Kif15	kinesin family member 15	KIF15	1.76E-02	-1.352
ILMN_1239422	NM_027435	ATPase family, AAA domain containing 2 (Atad2), mRNA. XM_977863 XM_986850 XM_986886 XM_986920 XM_986957 XM_986994 XM_987027 XM_987064 XM_987087 (S)	Atad2	ATPase family, AAA domain containing 2	ATAD2	3.28E-02	-1.351
ILMN_2590950	NM_009642	angiotensin II, type I receptor-associated protein (Agtrap), mRNA. (S)	Agtrap	angiotensin II, type I receptor-associated protein	Agtrap	2.03E-02	-1.350
ILMN_1243471	XM_001480808	PREDICTED: similar to ubiquitin-conjugating enzyme E2 variant 2 (LOC635992), mRNA. (S)	LOC635992	similar to ubiquitin-conjugating enzyme E2 variant 2	LOC635992	2.01E-02	-1.349
ILMN_2939367	NM_175751	zinc finger protein 608 (Zfp608), mRNA. XM_001000874 XM_001000888 XM_001000902 XM_001000914 XM_993937 XM_993952 XM_993993 XM_994038 XM_994057 XM_994076 (S)	Zfp608	zinc finger protein 608	Zfp608	1.42E-02	-1.349
ILMN_2527341	XM_355330	NaN (S)	LOC381365	n/a	n/a	4.26E-02	-1.348
ILMN_2809701	NM_080595	EMI domain containing 1 (Emid1), mRNA. (S)	Emid1	EMI domain containing 1	EMID1	1.71E-02	-1.347
ILMN_1217826	XR_005062	PREDICTED: similar to actin (LOC677448), misc RNA. (S)	LOC677448	predicted gene 8543; actin-like 8; predicted gene 7505; predicted gene 12715; predicted gene 12003; predicted gene 8399; predicted gene 6375; actin, gamma, cytoplasmic 1; similar to gamma-actin; predicted gene 4667; similar to cytoplasmic beta-actin; predicted gene 16385	Actg-ps1	3.79E-02	-1.346

ILMN_2771074	NM_016806	heterogeneous nuclear ribonucleoprotein A2/B1 (Hnrnpa2b1), transcript variant 1, mRNA. (S)	Hnrnpa2b1	predicted gene 5778; similar to heterogeneous nuclear ribonucleoprotein A2/B1; heterogeneous nuclear ribonucleoprotein A2/B1	Gm5778	8.68E-03	-1.346
ILMN_2531259	XR_033415	PREDICTED: similar to mortality factor 4 like 1 (LOC626309), misc RNA. (S)	LOC626309	predicted gene 6663	Gm6663	4.73E-02	-1.345
ILMN_2856166	NM_145605	kelch domain containing 4 (Klhdc4), mRNA. (S)	Klhdc4	kelch domain containing 4	klhdc4	1.30E-03	-1.344
ILMN_1257539	XM_284747	NaN (S)	LOC331539	n/a	n/a	9.06E-03	-1.344
ILMN_1248621	NM_201638	RIKEN cDNA G430022H21 gene (G430022H21Rik), mRNA. (S)	G430022H21Rik	methyltransferase like 14	METTL14	1.85E-02	-1.343
ILMN_1242911	NM_010439	high mobility group box 1 (Hmgb1), mRNA. (S)	Hmgb1	predicted gene 13121; predicted gene 3160; high-mobility group (nonhistone chromosomal) protein 1-like 1; predicted gene 6090; predicted gene 3851; predicted gene 8967; predicted gene 7782; predicted gene 4587; predicted gene 4689; predicted gene 3307; predicted gene 13932; predicted gene 15059; predicted gene 3565; predicted gene 15447; predicted gene 12587; predicted gene 9012; predicted gene 6115; predicted gene 9480; high mobility group box 1; predicted gene 8423; predicted gene 5853; predicted gene 8288; predicted gene 7888; predicted gene 8594; predicted gene 15387; predicted gene 5473; predicted gene 8807; similar to high mobility group box 1; similar to 2810416G20Rik protein; predicted gene 8390; predicted gene, OTTMUSG00000005439; predicted gene 5842; predicted gene 5527; predicted gene 8563; predicted gene 2710; predicted gene 12331; predicted gene 5937; predicted gene 5504; similar to high-mobility group box 1; predicted gene 10361; predicted gene 2607; predicted gene 7422; predicted gene 10075; predicted gene 12568; predicted gene 6589; predicted gene 4383; predicted gene 8031; similar to High mobility group protein 1 (HMG-1) (High mobility group protein B1) (Amphoterin) (Heparin-binding protein p30); predicted gene 7468; predicted gene 8554	LOC674543	1.54E-02	-1.342

ILMN_2780915	NM_145404	protein arginine N-methyltransferase 7 (Prmt7), mRNA. (S)	Prmt7	protein arginine N-methyltransferase 7	prmt7	3.15E-02	-1.341
ILMN_1222860	NM_001081687	predicted gene, 381484 (381484), mRNA. (S)	Gm5150	predicted gene 5150; similar to SIRP beta 1 like 1 protein	LOC100045212	7.69E-03	-1.340
ILMN_2764995	NM_023579	importin 5 (Ipo5), mRNA. (S)	Ipo5	hypothetical protein LOC100044315; importin 5	LOC100044315	1.57E-02	-1.339
ILMN_2636951	NM_016856	cleavage and polyadenylation specific factor 2 (Cpsf2), mRNA. (S)	Cpsf2	cleavage and polyadenylation specific factor 2	cpsf2	3.30E-02	-1.339
ILMN_1253410	NM_028850	cysteine-rich hydrophobic domain 2 (Chic2), mRNA. (S)	Chic2	cysteine-rich hydrophobic domain 2	Chic2	3.22E-02	-1.337
ILMN_1249160	AK035873	NaN (S)	9630013A09Rik	leucine-rich repeat LGI family, member 1; predicted gene 3888	Gm3888	1.49E-02	-1.337
ILMN_1241213	NM_008253	high mobility group box 3 (Hmgb3), mRNA. (S)	Hmgb3	predicted gene 11805; predicted gene 8850; high mobility group box 3; similar to High mobility group protein 4 (HMG-4) (High mobility group protein 2a) (HMG-2a)	Gm11805	3.28E-02	-1.337
ILMN_2934457	NM_009391	RAN, member RAS oncogene family (Ran), mRNA. (S)	Ran	RAS-like, family 2, locus 9; RAN, member RAS oncogene family; similar to RAN, member RAS oncogene family	LOC100045999	4.41E-03	-1.337
ILMN_2578165	AK087698	NaN (S)	E330008M07Rik	n/a	n/a	3.84E-02	-1.336
ILMN_2749954	XR_032130	PREDICTED: similar to high mobility group protein B2 (LOC667519), misc RNA. (S)	LOC667519	predicted gene 13160; predicted gene 8681; predicted gene 13237; predicted gene 4169; predicted gene 8284; similar to High mobility group box 2; predicted gene 13167; high mobility group box 2; predicted gene 13232	Gm13237	8.61E-03	-1.334
ILMN_2538518	XM_359177	NaN (S)	LOC386342	n/a	n/a	1.88E-02	-1.333
ILMN_2547548	AK014104	NaN (S)	3110031I02Rik	Wiskott-Aldrich syndrome-like (human)	WASL	3.19E-02	-1.329
ILMN_1238842	NM_016806	NaN (S)	Hnrpa2b1	predicted gene 5778; similar to heterogeneous nuclear ribonucleoprotein A2/B1; heterogeneous nuclear ribonucleoprotein A2/B1	Gm5778	4.92E-03	-1.328
ILMN_1248842	NM_177733	E2F transcription factor 2 (E2f2), mRNA. (S)	E2f2	E2F transcription factor 2	E2f2	1.27E-02	-1.327

ILMN_1232954	XR_030518	PREDICTED: similar to Polypyrimidine tract binding protein 1 (LOC236294), misc RNA. (S)	LOC236294	polypyrimidine tract binding protein 1; predicted gene 4900	Gm4900	1.03E-02	-1.326
ILMN_2740710	NM_145605	kelch domain containing 4 (Klhdc4), mRNA. (S)	Klhdc4	kelch domain containing 4	klhdc4	1.06E-02	-1.326
ILMN_2900910	NM_010860	myosin, light polypeptide 6, alkali, smooth muscle and non-muscle (Myl6), mRNA. (S)	Myl6	predicted gene 5915; predicted gene 8894; myosin, light polypeptide 6, alkali, smooth muscle and non-muscle; predicted gene 10080	Gm8894	4.51E-02	-1.324
ILMN_2736826	NM_009193	stem-loop binding protein (Slbp), mRNA. (S)	Slbp	stem-loop binding protein; predicted gene 8396	Slbp	1.09E-02	-1.321
ILMN_2893879	NM_024228	glycerophosphodiester phosphodiesterase domain containing 3 (Gdpd3), mRNA. (S)	Gdpd3	glycerophosphodiester phosphodiesterase domain containing 3	Gdpd3	2.53E-02	-1.320
ILMN_1235819	XM_001475422	PREDICTED: similar to RAN, member RAS oncogene family (LOC100045999), mRNA. (S)	LOC100045999	RAS-like, family 2, locus 9; RAN, member RAS oncogene family; similar to RAN, member RAS oncogene family	LOC100045999	1.05E-02	-1.319
ILMN_2617381	NM_175245	RIKEN cDNA 2410129H14 gene (2410129H14Rik), mRNA. (S)	2410129H14Rik	RIKEN cDNA 2410129H14 gene	2410129H14Rik	3.71E-02	-1.318
ILMN_1246536	XR_004022	PREDICTED: similar to DNA replication licensing factor MCM4 (CDC21 homolog) (P1-CDC21) (LOC672822), mRNA. (S)	LOC672822	predicted gene 9577	Gm9577	1.26E-02	-1.316
ILMN_2853360	NM_023651	peroxisomal biogenesis factor 13 (Pex13), mRNA. (S)	Pex13	peroxisomal biogenesis factor 13	Pex13	2.79E-02	-1.315
ILMN_2763651	NM_008722	NaN (S)	Npm1	predicted gene 6477; predicted gene 9118; nucleophosmin 1; similar to Nucleophosmin (NPM) (Nucleolar phosphoprotein B23) (Numatrin) (Nucleolar protein NO38); predicted gene 7289; predicted gene 5611	LOC100046628	3.39E-03	-1.313
ILMN_1219667	NM_011070	prefoldin 2 (Pfdn2), mRNA. (S)	Pfdn2	prefoldin 2	PFDN2	3.09E-02	-1.311
ILMN_2644587	NM_025840	basic leucine zipper and W2 domains 2 (Bzw2), mRNA. (S)	Bzw2	basic leucine zipper and W2 domains 2	BZW2	2.72E-02	-1.310
ILMN_2686463	NM_027860	NaN (S)	0610010F05Rik	RIKEN cDNA 0610010F05 gene	0610010F05Rik	4.43E-02	-1.310

ILMN_1235902	NM_010900	nuclear factor of activated T-cells, cytoplasmic, calcineurin-dependent 2 interacting protein (Nfatc2ip), mRNA. (S)	Nfatc2ip	nuclear factor of activated T-cells, cytoplasmic, calcineurin-dependent 2 interacting protein	NFATC2IP	3.70E-02	-1.308
ILMN_1244536	NM_010447	NaN (S)	Hnrpa1	heterogeneous nuclear ribonucleoprotein A1-like 2; similar to heterogeneous nuclear ribonucleoprotein A1; predicted gene 13418; predicted gene 5461; predicted gene 8652; predicted gene 5643; similar to Heterogeneous nuclear ribonucleoprotein A1 (Helix-destabilizing protein) (Single-strand RNA-binding protein) (hnRNP core protein A1) (HDP); heterogeneous nuclear ribonucleoprotein A1; predicted gene 6296	HNRNPA1L2	9.81E-03	-1.308
ILMN_2520812	NM_025282	NaN (S)	Mef2c	myocyte enhancer factor 2C	MEF2C	1.43E-02	-1.307
ILMN_3030392	NM_029485	RIKEN cDNA 5133400G04 gene (5133400G04Rik), transcript variant 2, mRNA. (I)	5133400G04Rik	RIKEN cDNA 5133400G04 gene	SPATA24	2.30E-02	-1.306
ILMN_2861406	NM_001009947	dedicator of cytokinesis 11 (Dock11), mRNA. (S)	Dock11	dedicator of cytokinesis 11	DOCK11	1.02E-02	-1.306
ILMN_2665369	NM_173861	CKT2 protein (Ckt2), mRNA. (S)	Ckt2	CKT2 protein	Csnka2ip	5.00E-02	-1.305
ILMN_2680424	NM_053124	SWI/SNF related, matrix associated, actin dependent regulator of chromatin, subfamily a, member 5 (Smarca5), mRNA. (S)	Smarca5	predicted gene 13034; SWI/SNF related, matrix associated, actin dependent regulator of chromatin, subfamily a, member 5	SMARCA5	2.76E-02	-1.305
ILMN_1234247	XM_912678	PREDICTED: similar to high mobility group nucleosomal binding domain 2 (LOC637089), mRNA. (S)	LOC637089	high mobility group nucleosomal binding domain 4; predicted gene 7931; predicted gene 10282; predicted gene 3338; high mobility group nucleosomal binding domain 2; predicted gene 6594; predicted gene 6750; predicted gene 10182; predicted gene 6724; predicted gene 4248; predicted gene 9525; predicted gene 15296; similar to Hmgn2 protein; predicted gene 7125; hypothetical protein LOC638323; predicted gene 6651; predicted gene 5899; predicted gene 14008; predicted gene 16494; similar to high mobility group nucleosomal binding domain 2	Gm10182	3.57E-02	-1.304

ILMN_2668319	NM_010480	heat shock protein 90, alpha (cytosolic), class A member 1 (Hsp90aa1), mRNA. (S)	Hsp90aa1	predicted gene 5511; heat shock protein 90, alpha (cytosolic), class A member 1	HSP90AA1	1.99E-02	-1.303
ILMN_2763371	NM_011133	polymerase (DNA directed), epsilon 2 (p59 subunit) (Pole2), mRNA. (S)	Pole2	polymerase (DNA directed), epsilon 2 (p59 subunit)	POLE2	3.93E-03	-1.303
ILMN_1214183	NM_178796	RIKEN cDNA A530064D06 gene (A530064D06Rik), mRNA. (S)	A530064D06Rik	RIKEN cDNA A530064D06 gene	A530064D06Rik	1.84E-02	-1.302
ILMN_2625352	NM_025624	proteasome maturation protein (Pomp), mRNA. (S)	Pomp	similar to proteasome maturation protein; proteasome maturation protein	POMP	1.11E-02	-1.302
ILMN_3156604	NM_008252	high mobility group box 2 (Hmgb2), mRNA. (A)	Hmgb2	predicted gene 13160; predicted gene 8681; predicted gene 13237; predicted gene 4169; predicted gene 8284; similar to High mobility group box 2; predicted gene 13167; high mobility group box 2; predicted gene 13232	Gm13237	1.07E-02	-1.301
ILMN_1260448	NM_152804	polo-like kinase 2 (Drosophila) (Plk2), mRNA. (S)	Plk2	polo-like kinase 2 (Drosophila)	plk2	3.56E-02	-1.300
ILMN_2799969	NM_152810	cell division cycle 5-like (S. pombe) (Cdc5l), mRNA. (S)	Cdc5l	predicted gene 9040; predicted gene 9046; predicted gene 9029; predicted gene 9048; cell division cycle 5-like (S. pombe); predicted gene 9049; predicted gene 9044; predicted gene 9030; predicted gene 9045	Gm9040	1.49E-02	-1.299
ILMN_2976441	NM_007549	B lymphoid kinase (Blk), mRNA. (S)	Blk	B lymphoid kinase	Blk	8.32E-03	-1.298
ILMN_1237333	XM_354567	NaN (S)	LOC380665	n/a	n/a	2.02E-03	-1.296
ILMN_2475585	NM_011694	voltage-dependent anion channel 1 (Vdac1), mRNA. (S)	Vdac1	voltage-dependent anion channel 1	VDAC1	1.31E-02	-1.295
ILMN_1214443	XM_290007	NaN (S)	LOC333756	n/a	n/a	1.62E-02	-1.295
ILMN_2606436	NM_019670	diaphanous homolog 3 (Drosophila) (Diap3), mRNA. (S)	Diap3	diaphanous homolog 3 (Drosophila)	Diap3	3.55E-02	-1.294
ILMN_2643486	XM_129785	NaN (S)	Plcl1	phospholipase C-like 1	PLCL1	4.58E-02	-1.293
ILMN_3000318	NM_130863	adrenergic receptor kinase, beta 1 (Adrbk1), mRNA. (S)	Adrbk1	adrenergic receptor kinase, beta 1	ADRBK1	3.20E-02	-1.293
ILMN_1247047	XM_891442	PREDICTED: heterogeneous nuclear ribonucleoprotein A0, transcript variant 2 (Hnrpa0), mRNA. (S)	Hnrpa0	heterogeneous nuclear ribonucleoprotein A0	HNRNPA0	1.67E-02	-1.293

ILMN_1244949	AK052932	NaN (S)	Snta1	syntrophin, acidic 1	snta1	1.95E-02	-1.291
ILMN_2957614	NM_007422	adenylosuccinate synthetase, non muscle (Adss), mRNA. (S)	Adss	adenylosuccinate synthetase, non muscle	ADSS	2.06E-02	-1.290
ILMN_1220575	AK011368	NaN (S)	2610010G17Rik	RIKEN cDNA 9630014M24 gene; Rho GTPase activating protein 26; predicted gene 5820	Gm5820	1.25E-02	-1.290
ILMN_1212698	NM_009667	adenosine monophosphate deaminase 3 (Ampd3), mRNA. (S)	Ampd3	adenosine monophosphate deaminase 3	AMPD3	4.28E-02	-1.290
ILMN_2706061	XM_001475106	PREDICTED: similar to PTB-associated splicing factor (LOC100045887), mRNA. (S)	LOC100045887	splicing factor proline/glutamine rich (polypyrimidine tract binding protein associated); similar to PTB-associated splicing factor	LOC100045887	4.23E-02	-1.289
ILMN_2576268	AK045865	NaN (S)	Flt3	FMS-like tyrosine kinase 3	FLT3	5.38E-03	-1.288
ILMN_1258435	AK082238	NaN (S)	C230027G13Rik	n/a	n/a	2.38E-02	-1.288
ILMN_2519679	NM_001081079	opioid growth factor receptor-like 1 (Ogfrl1), mRNA. (S)	Ogfrl1	opioid growth factor receptor-like 1	OGFRL1	1.66E-02	-1.288
ILMN_2629582	NM_008873	plasminogen activator, urokinase (Plau), mRNA. (S)	Plau	plasminogen activator, urokinase	PLAU	1.75E-02	-1.287
ILMN_2721439	NM_007792	cysteine and glycine-rich protein 2 (Csrp2), mRNA. (S)	Csrp2	cysteine and glycine-rich protein 2	CSRP2	3.83E-02	-1.287
ILMN_1231087	NM_010703	NaN (S)	Lef1	lymphoid enhancer binding factor 1	Lef1	1.20E-03	-1.286
ILMN_2424756	NM_001045529	microrchidia 3 (Morc3), mRNA. XM_978468 XM_978538 XM_978584 XM_978615 (S)	Morc3	microrchidia 3	MORC3	1.84E-02	-1.286
ILMN_1234698	NM_027533	tetraspanin 2 (Tspan2), mRNA. (S)	Tspan2	tetraspanin 2	Tspan2	2.50E-02	-1.285
ILMN_3002281	NM_026570	YEATS domain containing 4 (Yeats4), mRNA. (S)	Yeats4	YEATS domain containing 4	Yeats4	2.18E-02	-1.283
ILMN_3007072	NM_024427	tropomyosin 1, alpha (Tpm1), mRNA. (S)	Tpm1	tropomyosin 1, alpha	TPM1	9.60E-03	-1.281
ILMN_2654952	XM_001475189	PREDICTED: similar to Hmgcs1 protein, transcript variant 1 (LOC100040592), mRNA. (S)	LOC100040592	similar to Hmgcs1 protein; 3-hydroxy-3-methylglutaryl-Coenzyme A synthase 1	LOC100040592	3.75E-02	-1.279

ILMN_2775450	XM_001480156	PREDICTED: similar to DNA directed RNA polymerase II polypeptide A (LOC100043526), mRNA. (S)	LOC100043526	Rho GTPase activating protein 11A; predicted gene 4500; similar to DNA directed RNA polymerase II polypeptide A	LOC100048579	2.18E-02	-1.279
ILMN_1246490	XM_484778	NaN (S)	Zfp91	n/a	n/a	1.43E-02	-1.279
ILMN_1254513	NM_026541	NaN (S)	4930553M18Rik	n/a	n/a	1.29E-03	-1.277
ILMN_2893564	NM_001033144	RIKEN cDNA 1190007F08 gene (1190007F08Rik), mRNA. XM_921339 XM_921348 XM_921353 (S)	1190007F08Rik	RIKEN cDNA 1190007F08 gene	1190007F08Rik	4.41E-02	-1.275
ILMN_2841289	NM_009396	tumor necrosis factor, alpha-induced protein 2 (Tnfaip2), mRNA. (S)	Tnfaip2	tumor necrosis factor, alpha-induced protein 2	TNFAIP2	4.25E-02	-1.275
ILMN_2741132	NM_008251	high mobility group nucleosomal binding domain 1 (Hmgn1), mRNA. (S)	Hmgn1	predicted gene 11663; predicted gene 2992; predicted gene 7862; high mobility group nucleosomal binding domain 1; similar to high mobility group nucleosomal binding domain 1	HMGN1	1.81E-04	-1.274
ILMN_2703529	NM_009186	splicing factor, arginine/serine-rich 10 (transformer 2 homolog, Drosophila) (Sfrs10), mRNA. (S)	Sfrs10	transformer 2 beta homolog (Drosophila); predicted gene 6439	TRA2B	3.40E-03	-1.274
ILMN_2962840	NM_009838	chaperonin containing Tcp1, subunit 6a (zeta) (Cct6a), mRNA. (S)	Cct6a	chaperonin containing Tcp1, subunit 6a (zeta)	cct6a	3.33E-02	-1.273
ILMN_2919850	NM_008511	lymphoid-restricted membrane protein (Lrmp), mRNA. (S)	Lrmp	lymphoid-restricted membrane protein	LRMP	4.45E-02	-1.273
ILMN_1255013	NM_010561	NaN (S)	Ilf3	interleukin enhancer binding factor 3	ilf3	4.71E-02	-1.272
ILMN_1222059	AK089315	NaN (S)	Thbs1	n/a	n/a	9.84E-03	-1.271
ILMN_1236960	NM_001081652	cDNA sequence AB182283 (AB182283), mRNA. (S)	AB182283	cDNA sequence AB182283	NACAD	4.27E-03	-1.271
ILMN_1223544	AK079205	NaN (S)	Copz1	coatomer protein complex, subunit zeta 1	copz1	4.26E-02	-1.270
ILMN_2771441	NM_173764	transmembrane anterior posterior transformation 1 (Tapt1), mRNA. (S)	Tapt1	transmembrane anterior posterior transformation 1	tapt1	2.39E-02	-1.267

ILMN_2698958	NM_029711	actin related protein 2/3 complex, subunit 2 (Arpc2), mRNA. XM_129773 XM_901710 XM_901712 XM_901713 XM_922647 XM_922652 XM_922657 XM_989557 (S)	Arpc2	predicted gene 5492; actin related protein 2/3 complex, subunit 2	arpc2	3.45E-02	-1.267
ILMN_2711642	XM_918222	PREDICTED: similar to cyclin-dependent kinase 2-interacting protein (LOC640972), mRNA. (S)	LOC640972	RIKEN cDNA 2810452K22 gene; similar to cyclin-dependent kinase 2-interacting protein	CINP	3.98E-02	-1.267
ILMN_1377924	NM_008084	glyceraldehyde-3-phosphate dehydrogenase (Gapdh), mRNA. (S)	Gapdh	gene 10290; predicted gene 10566; predicted gene 10291; predicted gene 3200; predicted gene 12070; predicted gene 7286; predicted gene 6946; predicted gene 8825; predicted gene 9081; glyceraldehyde-3-phosphate dehydrogenase; predicted gene 10359; predicted gene 10358; predicted gene 7784; predicted gene 12416; predicted gene 14148; predicted gene 13882; predicted gene 4217; predicted gene 7183; predicted gene 10313; predicted gene 2467; predicted gene 5787; predicted gene 2193; predicted gene 13292; predicted gene, 380687; predicted gene 2076; predicted gene 3222; predicted gene 7507; predicted gene 7293; predicted gene 15191; predicted gene, 674324; predicted gene 10284; predicted gene 8055; similar to hCG1978856; predicted gene 2574; similar to glyceraldehyde-3-phosphate dehydrogenase; predicted gene 7545; predicted gene 12033; predicted gene 3272; predicted gene 3534; predicted gene 2308; predicted gene 3839; predicted gene 9061; predicted gene 3671; predicted gene 8513; predicted gene 4654; predicted gene 12537; predicted gene 4929; predicted gene 5652; predicted gene 9568; predicted gene 8349; predicted gene 2546; predicted gene 7129; similar to Glyceraldehyde-3-phosphate dehydrogenase (GAPDH); predicted gene 8318; glyceraldehyde-3-phosphate dehydrogenase pseudogene; predicted gene 7611; predicted gene 2445; predicted gene 6283; predicted gene 11557; predicted gene 4335; predicted gene 9127; predicted gene 3695; predicted gene 5507; predicted gene 8174; predicted gene 4609;	Gm4335	3.53E-02	-1.264

ILMN_2852533	NM_016661	S-adenosylhomocysteine hydrolase (Ahcy), mRNA. (S)	Ahcy	similar to Adenosylhomocysteinase (S-adenosyl-L-homocysteine hydrolase) (AdoHcyase) (Liver copper-binding protein) (CUBP); S-adenosylhomocysteine hydrolase	ahcY	2.36E-02	-1.264
ILMN_2772446	XM_001479002	PREDICTED: similar to activating transcription factor 1 (LOC100047421), mRNA. (S)	LOC100047421	predicted gene 1862; activating transcription factor 1; similar to activating transcription factor 1	LOC100047421	1.82E-05	-1.261
ILMN_2674763	XR_033873	PREDICTED: similar to gamma actin-like protein (LOC666979), misc RNA. (S)	LOC666979	predicted gene 8543; actin-like 8; predicted gene 7505; predicted gene 12715; predicted gene 12003; predicted gene 8399; predicted gene 6375; actin, gamma, cytoplasmic 1; similar to gamma-actin; predicted gene 4667; similar to cytoplasmic beta-actin; predicted gene 16385	Actg-ps1	4.12E-02	-1.261
ILMN_2540124	XM_358206	NaN (S)	LOC385360	n/a	n/a	3.91E-02	-1.261
ILMN_2745532	NM_025795	non-SMC condensin II complex, subunit H2 (Ncaph2), mRNA. (S)	Ncaph2	non-SMC condensin II complex, subunit H2	ncaph2	4.75E-02	-1.259
ILMN_1248686	AK044352	NaN (S)	A930008K05Rik	n/a	n/a	4.84E-02	-1.259
ILMN_2784821	NM_025279	heterogeneous nuclear ribonucleoprotein K (Hnrpk), mRNA. (S)	Hnrpk	heterogeneous nuclear ribonucleoprotein K; predicted gene 7964	Gm7964	7.91E-03	-1.257
ILMN_2732718	NM_011625	protein phosphatase 1, regulatory (inhibitor) subunit 13B (Ppp1r13b), mRNA. (S)	Ppp1r13b	protein phosphatase 1, regulatory (inhibitor) subunit 13B	PPP1R13B	3.99E-02	-1.257
ILMN_2509623	NM_176850	bromodomain PHD finger transcription factor (Bptf), mRNA. (S)	Bptf	bromodomain PHD finger transcription factor	BPTF	3.93E-02	-1.256
ILMN_2921103	NM_029804	heterogeneous nuclear ribonucleoprotein M (Hnrpm), mRNA. (S)	Hnrpm	heterogeneous nuclear ribonucleoprotein M	hnrnpm	3.47E-02	-1.256
ILMN_2588955	NM_153071	G protein-coupled receptor, family C, group 6, member A (Gprc6a), mRNA. (S)	Gprc6a	G protein-coupled receptor, family C, group 6, member A	GPRC6A	2.55E-03	-1.255
ILMN_1245872	NM_029649	transmembrane protein 101 (Tmem101), mRNA. (S)	Tmem101	transmembrane protein 101	TMEM101	1.94E-02	-1.255
ILMN_2476139	XM_147357	NaN (S)	Tuba6	n/a	n/a	2.99E-02	-1.254

ILMN_1233175	XM_906862	PREDICTED: similar to PNG protein (LOC632667), mRNA. (S)	LOC632667	similar to protein phosphatase 1, regulatory (inhibitor) subunit 14B	LOC632667	3.34E-02	-1.253
ILMN_2581479	NM_001081363	centromere protein F (Cenpf), mRNA. (S)	Cenpf	centromere protein F	CENPF	2.63E-02	-1.252
ILMN_2542048	XM_356566	NaN (S)	LOC382555	n/a	n/a	4.22E-02	-1.251
ILMN_1237326	XM_193691	NaN (S)	LOC268602	n/a	n/a	2.88E-02	-1.250
ILMN_2622671	NM_007981	acyl-CoA synthetase long-chain family member 1 (Acsl1), mRNA. (S)	Acsl1	acyl-CoA synthetase long-chain family member 1	ACSL1	1.47E-02	-1.249
ILMN_2701485	NM_133501	netrin G2 (Ntng2), transcript variant b, mRNA. (S)	Ntng2	RIKEN cDNA 6530402F18 gene; netrin G2	NTNG2	2.26E-02	-1.249
ILMN_2856861	NR_001572	nuclear distribution gene C homolog (Aspergillus), pseudogene 1 (Nudc-ps1), non-coding RNA. (S)	Nudc-ps1	nuclear distribution gene C homolog (Aspergillus), pseudogene 1; nuclear distribution gene C homolog (Aspergillus)	nudC	1.23E-02	-1.246
ILMN_1251260	AK043273	NaN (S)	Adss2	adenylosuccinate synthetase, non muscle	ADSS	4.72E-02	-1.245
ILMN_2592285	NM_145468	S-phase kinase-associated protein 2 (p45) (Skp2), transcript variant 2, mRNA. (S)	Skp2	S-phase kinase-associated protein 2 (p45)	Skp2	2.11E-02	-1.245
ILMN_3002290	NM_145073	histone cluster 1, H3g (Hist1h3g), mRNA. (S)	Hist1h3g	histone cluster 1, H3i; histone cluster 1, H3h; histone cluster 1, H3g; predicted gene 12260; histone cluster 1, H3a	hist1h3g	1.49E-02	-1.243
ILMN_2505185	XR_003788	PREDICTED: similar to Transcription factor SOX-4 (LOC672274), mRNA. (S)	LOC672274	n/a	n/a	1.92E-02	-1.243
ILMN_1224868	NM_007531	prohibitin 2 (Phb2), mRNA. (S)	Phb2	prohibitin 2	PHB2	3.12E-02	-1.242
ILMN_1250195	NM_008681	N-myc downstream regulated gene 1 (Ndrg1), mRNA. (S)	Ndrg1	N-myc downstream regulated gene 1	ndrg1	3.14E-02	-1.238
ILMN_1228672	XM_358181	NaN (S)	LOC385308	n/a	n/a	1.18E-02	-1.237
ILMN_1253184	NM_001081258	kinesin family member 14 (Kif14), mRNA. (S)	Kif14	kinesin family member 14	KIF14	1.11E-02	-1.236

ILMN_2770739	NM_001038642	E26 avian leukemia oncogene 1, 5' domain (Ets1), transcript variant 2, mRNA. (S)	Ets1	E26 avian leukemia oncogene 1, 5' domain	ETS1	4.56E-02	-1.235
ILMN_2568505	AK041099	NaN (S)	A530083B17Rik	RIKEN cDNA E430029J22 gene; similar to Interferon-activatable protein 203 (Ifi-203) (Interferon-inducible protein p203)	E430029J22Rik	4.33E-02	-1.234
ILMN_2547942	NM_178856	GINS complex subunit 2 (Psf2 homolog) (Gins2), mRNA. (S)	Gins2	GINS complex subunit 2 (Psf2 homolog)	GINS2	2.52E-02	-1.234
ILMN_2618257	NM_144813	solute carrier family 24 (sodium/potassium/calcium exchanger), member 1 (Slc24a1), mRNA. (S)	Slc24a1	solute carrier family 24 (sodium/potassium/calcium exchanger), member 1	SLC24A1	4.19E-02	-1.233
ILMN_2742647	XM_984926	PREDICTED: predicted gene, EG666609 (EG666609), mRNA. (S)	EG666609	predicted gene 8186; small nuclear ribonucleoprotein polypeptide G	Snrpg	2.10E-02	-1.232
ILMN_2539893	XR_004709	PREDICTED: similar to high-mobility group box 1 (LOC674960), misc RNA. (S)	LOC674960	predicted gene 14448	Gm14448	4.69E-02	-1.232
ILMN_1247736	XM_001002943	PREDICTED: hypothetical LOC677375 (LOC677375), mRNA. (S)	LOC677375	n/a	n/a	2.68E-02	-1.231
ILMN_2928599	NM_178706	sialic acid binding Ig-like lectin H (Siglech), mRNA. (S)	Siglech	sialic acid binding Ig-like lectin H	Siglech	2.48E-02	-1.230
ILMN_2762189	NM_026181	G patch domain containing 1 (Gpatch1), mRNA. (S)	Gpatch1	G patch domain containing 1	GPATCH1	3.62E-02	-1.230
ILMN_2844316	NM_134040	DEAD (Asp-Glu-Ala-Asp) box polypeptide 1 (Ddx1), mRNA. (S)	Ddx1	DEAD (Asp-Glu-Ala-Asp) box polypeptide 1	DDX1	2.04E-02	-1.230
ILMN_2985428	NM_031404	actin-like 6B (Actl6b), mRNA. (S)	Actl6b	actin-like 6B	ACTL6B	3.14E-02	-1.229
ILMN_2809656	NM_001033529	RIKEN cDNA 2210038L17 gene (2210038L17Rik), mRNA. (S)	2210038L17Rik	similar to mKIAA1397 protein; tubby like protein 4	TULP4	2.88E-02	-1.229
ILMN_2585829	AK081280	NaN (S)	C030044F15Rik	n/a	n/a	4.92E-02	-1.228
ILMN_2607786	NM_146094	fatty acid desaturase 1 (Fads1), mRNA. (S)	Fads1	fatty acid desaturase 1	Fads1	1.91E-03	-1.227
ILMN_2530276	XM_207121	NaN (S)	LOC280118	n/a	n/a	3.26E-02	-1.227
ILMN_1219432	AK078286	NaN (S)	6430571H07Rik	WD repeat domain 33	WDR33	2.75E-02	-1.226

ILMN_2549257	AK016197	NaN (S)	4930563C04Rik	proline, glutamic acid and leucine rich protein 1	pelp1	3.98E-02	-1.225
ILMN_1241414	AK030636	NaN (S)	4930429A08Rik	S100P binding protein	S100PBP	9.47E-03	-1.225
ILMN_2881864	NM_015780	complement factor H-related 1 (Cfhr1), mRNA. (S)	Cfhr1	complement factor H-related 1	Cfhr1	3.63E-02	-1.223
ILMN_1238117	XM_359045	NaN (S)	LOC386054	n/a	n/a	2.67E-02	-1.222
ILMN_2704237	NM_008300	heat shock protein 4 (Hspa4), mRNA. (S)	Hspa4	heat shock protein 4	HSPA4	2.23E-02	-1.222
ILMN_2725517	NM_134048	Casitas B-lineage lymphoma-like 1 (Cbll1), mRNA. (S)	Cbll1	Casitas B-lineage lymphoma-like 1	CBLL1	1.53E-02	-1.220
ILMN_2594165	XM_135092	NaN (S)	2510040D07Rik	n/a	n/a	2.99E-02	-1.219
ILMN_1259473	NM_007999	flap structure specific endonuclease 1 (Fen1), mRNA. (S)	Fen1	flap structure specific endonuclease 1	FEN1	4.66E-02	-1.219
ILMN_2615096	NM_010074	dipeptidylpeptidase 4 (Dpp4), mRNA. (S)	Dpp4	dipeptidylpeptidase 4	DPP4	4.44E-02	-1.218
ILMN_2567274	AK047228	NaN (S)	1200011A11Rik	vacuolar protein sorting 11 (yeast)	VPS11	3.00E-02	-1.218
ILMN_1218305	XM_358963	NaN (S)	LOC385830	n/a	n/a	9.31E-03	-1.215
ILMN_2758249	NM_029291	activating signal cointegrator 1 complex subunit 2 (Ascc2), mRNA. (S)	Ascc2	activating signal cointegrator 1 complex subunit 2	ASCC2	4.41E-02	-1.214
ILMN_2588055	NM_007393	actin, beta (Actb), mRNA. (S)	Actb	actin, beta	ACTB	2.21E-02	-1.213
ILMN_2518483	NM_023196	NaN (S)	Pla2g12a	phospholipase A2, group XIIA	Pla2g12a	2.94E-02	-1.212
ILMN_2709856	NM_028871	heterogeneous nuclear ribonucleoprotein R (Hnrnpr), mRNA. (S)	Hnrnpr	predicted gene 6159; heterogeneous nuclear ribonucleoprotein R	Gm6159	3.26E-02	-1.212
ILMN_2726315	NM_011341	stromal cell derived factor 4 (Sdf4), mRNA. (S)	Sdf4	stromal cell derived factor 4	SDF4	6.94E-03	-1.212
ILMN_1245003	NM_013615	outer dense fiber of sperm tails 2 (Odf2), mRNA. (S)	Odf2	outer dense fiber of sperm tails 2; similar to outer dense fiber of sperm tails 2	Odf2	3.48E-02	-1.212

ILMN_1233896	XM_916287	PREDICTED: similar to TIFA, transcript variant 1 (LOC637082), mRNA. (S)	LOC637082	TRAF-interacting protein with forkhead-associated domain; similar to Traf2 binding protein	LOC637082	3.48E-02	-1.210
ILMN_2979052	NM_026623	nudix (nucleoside diphosphate linked moiety X)-type motif 21 (Nudt21), mRNA. (S)	Nudt21	nudix (nucleoside diphosphate linked moiety X)-type motif 21	NUDT21	4.68E-02	-1.210
ILMN_2976440	NM_007549	B lymphoid kinase (Blk), mRNA. (S)	Blk	B lymphoid kinase	Blk	3.98E-02	-1.210
ILMN_1220677	XM_203729	NaN (S)	LOC277281	n/a	n/a	4.52E-02	-1.210
ILMN_2933647	NM_019802	gamma-glutamyl carboxylase (Ggcx), mRNA. (S)	Ggcx	gamma-glutamyl carboxylase	GGCX	1.45E-02	-1.208
ILMN_1257181	XR_034668	PREDICTED: similar to high-mobility group box 1 (LOC100048378), misc RNA. (S)	LOC100048378	predicted gene 13121; predicted gene 3160; high-mobility group (nonhistone chromosomal) protein 1-like 1; predicted gene 6090; predicted gene 3851; predicted gene 8967; predicted gene 7782; predicted gene 4587; predicted gene 4689; predicted gene 3307; predicted gene 13932; predicted gene 15059; predicted gene 3565; predicted gene 15447; predicted gene 12587; predicted gene 9012; predicted gene 6115; predicted gene 9480; high mobility group box 1; predicted gene 8423; predicted gene 5853; predicted gene 8288; predicted gene 7888; predicted gene 8594; predicted gene 15387; predicted gene 5473; predicted gene 8807; similar to high mobility group box 1; similar to 2810416G20Rik protein; predicted gene 8390; predicted gene, OTTMUSG00000005439; predicted gene 5842; predicted gene 5527; predicted gene 8563; predicted gene 2710; predicted gene 12331; predicted gene 5937; predicted gene 5504; similar to high-mobility group box 1; predicted gene 10361; predicted gene 2607; predicted gene 7422; predicted gene 10075; predicted gene 12568; predicted gene 6589; predicted gene 4383; predicted gene 8031; similar to High mobility group protein 1 (HMG-1) (High mobility group protein B1) (Amphoterin) (Heparin-binding protein p30); predicted gene 7468; predicted gene 8554	LOC674543	4.08E-02	-1.208

ILMN_2549226	AK016121	NaN (S)	4930554K12Rik	tankyrase, TRF1-interacting ankyrin-related ADP-ribose polymerase	tnks	1.87E-02	-1.207
ILMN_2433075	XM_001478311	PREDICTED: similar to heterochromatin protein 1 beta (LOC100047028), mRNA. (S)	LOC100047028	similar to heterochromatin protein 1 beta; chromobox homolog 1 (Drosophila HP1 beta)	CBX1	3.87E-02	-1.207
ILMN_2552732	AK033474	NaN (S)	9030216K14Rik	acyloxyacyl hydrolase	Aoah	1.77E-02	-1.207
ILMN_1221290	XR_032548	PREDICTED: similar to HIStone family member (his-71) (LOC636952), misc RNA. (S)	LOC636952	predicted gene 14383; predicted gene 3835; predicted gene 14384; predicted gene 12950; predicted gene, 670915; H3 histone, family 3A; predicted gene 12657; predicted gene 6132; predicted gene 10257; predicted gene 7227; H3 histone, family 3B; predicted gene 6128; similar to histone; predicted gene 1986; predicted gene 6186; hypothetical protein LOC676337; predicted gene 6421; predicted gene 2198; predicted gene 6817; predicted gene 8095; predicted gene 12271; predicted gene 13529; predicted gene 8029; predicted gene 4938; predicted gene 7100; predicted gene 9014; similar to Histone H3.4 (Embryonic); predicted gene 7179; similar to H3 histone, family 3B; predicted gene 7900; predicted gene 2099; similar to H3 histone, family 3A; predicted gene 6749; predicted gene 6485; predicted gene 4028; predicted gene 7194	Gm3835	2.06E-02	-1.206
ILMN_1234143	XM_912340	PREDICTED: predicted gene, EG384525 (EG384525), mRNA. (S)	EG384525	predicted gene 10027; hypothetical protein LOC674425; predicted gene 6794; predicted gene 9175; similar to ribosomal protein S18; similar to ribosomal protein; ribosomal protein S18; predicted gene 11230; predicted gene 5321; predicted gene 10260; predicted gene 8599; predicted gene 8268	LOC100047329	2.56E-02	-1.203
ILMN_2417813	NM_009449	tubulin, alpha 3B (Tuba3b), mRNA. (S)	Tuba3b	predicted gene 5366; tubulin, alpha 3B; tubulin, alpha 3A	Tuba3a	4.60E-02	-1.203
ILMN_3011353	NM_001025245	myelin basic protein (Mbp), transcript variant 8, mRNA. (I)	Mbp	myelin basic protein	MBP	7.57E-03	-1.202
ILMN_1220530	NM_019710	structural maintenance of chromosomes 1A (Smc1a), mRNA. (S)	Smc1a	structural maintenance of chromosomes 1A	Smc1a	3.57E-02	-1.201
ILMN_1254276	NM_175662	histone cluster 2, H2ac (Hist2h2ac), mRNA. (S)	Hist2h2ac	histone cluster 2, H2ac	Hist2h2ac	4.85E-02	-1.200

ILMN_1214178	XM_923363	PREDICTED: RIKEN cDNA D430007A19 gene, transcript variant 3 (D430007A19Rik), mRNA. (S)	D430007A19Rik	RIKEN cDNA D430007A19 gene	D430007A19Rik	3.23E-02	-1.200
ILMN_1227643	XM_134614	NaN (S)	LOC234897	n/a	n/a	4.61E-02	-1.199
ILMN_2532998	XM_142564	NaN (S)	LOC245128	n/a	n/a	4.69E-02	-1.198
ILMN_2883990	NM_020296	RNA binding motif, single stranded interacting protein 1 (Rbms1), mRNA. (S)	Rbms1	RNA binding motif, single stranded interacting protein 1	RBMS1	1.59E-02	-1.198
ILMN_1252036	NM_139232	FYVE, RhoGEF and PH domain containing 4 (Fgd4), transcript variant alpha, mRNA. (S)	Fgd4	FYVE, RhoGEF and PH domain containing 4	FGD4	4.72E-02	-1.196
ILMN_2729608	NM_175013	NaN (S)	Pgm5	phosphoglucomutase 5	PGM5	3.89E-02	-1.196
ILMN_1222597	AK082352	NaN (S)	C230040D17Rik	ankyrin 2, brain	ank2	3.18E-02	-1.194
ILMN_2637704	NM_009860	NaN (S)	Cdc25c	cell division cycle 25 homolog C (S. pombe)	CDC25C	4.85E-02	-1.194
ILMN_1249002	XM_990404	PREDICTED: hypothetical LOC676484 (LOC676484), mRNA. (S)	LOC676484	predicted gene 9679	Gm9679	3.06E-02	-1.194
ILMN_2767644	XM_488673	NaN (S)	BC024659	n/a	n/a	1.11E-02	-1.193
ILMN_2574966	AK040144	NaN (S)	A430069L09Rik	predicted gene 9951	Gm9951	3.23E-02	-1.192
ILMN_2971758	NM_028677	peptidyl prolyl isomerase H (Ppih), mRNA. (S)	Ppih	similar to peptidyl prolyl isomerase H; predicted gene 7879; predicted gene 9088; predicted gene 8719; predicted gene 11585; similar to Peptidyl-prolyl cis-trans isomerase H (PPIase H) (Rotamase H); peptidyl prolyl isomerase H	Ppih	5.74E-03	-1.192
ILMN_2529497	XM_144000	PREDICTED: hypothetical LOC230622 (LOC230622), mRNA. (S)	LOC230622	selection and upkeep of intraepithelial T cells 5; similar to Butyrophilin precursor (BT) (Butyrophilin subfamily 1 member A1); selection and upkeep of intraepithelial T cells 6	LOC639920	1.02E-02	-1.192
ILMN_3136397	NM_001039239	predicted gene, EG630579 (EG630579), mRNA. (A)	EG630579	predicted gene 3325; predicted gene 7036	Zfp808	1.56E-02	-1.191

ILMN_2815383	NM_026040	serum response factor binding protein 1 (Srfbp1), mRNA. (S)	Srfbp1	serum response factor binding protein 1; similar to SRF-dependent transcription regulation associated protein	SRFBP1	3.59E-02	-1.190
ILMN_1249522	NM_013853	ATP-binding cassette, sub-family F (GCN20), member 2 (Abcf2), nuclear gene encoding mitochondrial protein, mRNA. (S)	Abcf2	ATP-binding cassette, sub-family F (GCN20), member 2	ABCF2	4.88E-02	-1.190
ILMN_2773099	NM_146141	pyrophosphatase (inorganic) 2 (Ppa2), nuclear gene encoding mitochondrial protein, mRNA. (S)	Ppa2	pyrophosphatase (inorganic) 2	PPA2	1.11E-02	-1.188
ILMN_1251832	AK019209	NaN (S)	Nifk	Mki67 (FHA domain) interacting nucleolar phosphoprotein	Mki67ip	9.91E-03	-1.188
ILMN_2479227	NM_173053	LIM motif-containing protein kinase 2 (Limk2), transcript variant 2, mRNA. (S)	Limk2	LIM motif-containing protein kinase 2	LIMK2	3.10E-02	-1.188
ILMN_2634670	NM_008586	NaN (S)	Mep1b	meprin 1 beta	mep1b	3.28E-02	-1.188
ILMN_1220039	NM_008446	kinesin family member 4 (Kif4), mRNA. (S)	Kif4	kinesin family member 4	Kif4	4.69E-02	-1.187
ILMN_2723955	NM_207573	olfactory receptor 1380 (Olfr1380), mRNA. (S)	Olfr1380	olfactory receptor 1380	Olfr1380	2.66E-02	-1.186
ILMN_2758762	NM_030205	coronin 7 (Coro7), mRNA. (S)	Coro7	coronin 7	CORO7	4.54E-02	-1.186
ILMN_2614477	NM_181417	cysteine and glycine-rich protein 2 binding protein (Csrp2bp), mRNA. (S)	Csrp2bp	cysteine and glycine-rich protein 2 binding protein; similar to cysteine and glycine-rich protein 2 binding protein; similar to CSRP2BP; hypothetical protein LOC100048725	csrp2bp	2.53E-02	-1.184
ILMN_2589785	XM_001479297	PREDICTED: similar to calponin 3, acidic (LOC100047856), mRNA. (S)	LOC100047856	similar to calponin 3, acidic; predicted gene 4815; calponin 3, acidic	Gm4815	2.77E-02	-1.183
ILMN_1215281	XM_001475087	PREDICTED: T cell receptor gamma chain (Tcrg), mRNA. (S)	Tcrg	n/a	n/a	8.78E-03	-1.183
ILMN_2531794	XM_145046	NaN (S)	LOC213440	n/a	n/a	2.41E-03	-1.183
ILMN_2822359	NM_017390	seminal vesicle protein, secretion 2 (Svs2), mRNA. (S)	Svs2	semenogelin I	Semg1	3.49E-02	-1.181
ILMN_1258197	AK051815	NaN (S)	D230004N01Rik	RIKEN cDNA C330023M02 gene	NAA25	3.18E-02	-1.180
ILMN_2950067	NM_001004176	mastermind like 3 (Drosophila) (Maml3), mRNA. (S)	Maml3	mastermind like 3 (Drosophila)	MAML3	1.30E-02	-1.180

ILMN_2734391	NM_016894	receptor (calcitonin) activity modifying protein 1 (Ramp1), mRNA. (S)	Ramp1	receptor (calcitonin) activity modifying protein 1	RAMP1	4.13E-02	-1.179
ILMN_2731774	NM_001002896	beaded filament structural protein 2, phakinin (Bfsp2), mRNA. (S)	Bfsp2	beaded filament structural protein 2, phakinin	BFSP2	1.36E-02	-1.179
ILMN_2588061	XM_204841	NaN (S)	LOC277157	n/a	n/a	1.49E-02	-1.179
ILMN_1252393	XM_001005599	PREDICTED: similar to 60S ribosomal protein L3 (L4) (LOC635340), mRNA. (S)	LOC635340	n/a	n/a	3.76E-02	-1.177
ILMN_2997106	NM_025403	nucleolar protein family A, member 3 (Nola3), mRNA. (S)	Nola3	NOP10 ribonucleoprotein homolog (yeast)	Nop10	4.10E-02	-1.176
ILMN_2587863	XM_146886	NaN (I)	2410091N08Rik	n/a	n/a	2.56E-02	-1.174
ILMN_1225007	XM_986204	PREDICTED: RIKEN cDNA A830010M20 gene, transcript variant 2 (A830010M20Rik), mRNA. (S)	A830010M20Rik	RIKEN cDNA A830010M20 gene	A830010M20Rik	2.50E-02	-1.174
ILMN_1227114	AK051043	NaN (S)	D030059I21Rik	leucine rich repeat containing 1	LRRC1	1.52E-02	-1.174
ILMN_1237452	XR_031052	PREDICTED: similar to hCG2022736 (LOC382722), misc RNA. (S)	LOC382722	predicted gene 11652; predicted gene 5191; basic leucine zipper and W2 domains 1	BZW1	4.55E-02	-1.173
ILMN_3161607	XR_035440	PREDICTED: RIKEN cDNA 9230020A06 gene (9230020A06Rik), misc RNA. (S)	9230020A06Rik	RIKEN cDNA 9230020A06 gene	9230020A06Rik	2.18E-02	-1.173
ILMN_2883907	NM_133939	LSM8 homolog, U6 small nuclear RNA associated (S. cerevisiae) (Lsm8), mRNA. (S)	Lsm8	LSM8 homolog, U6 small nuclear RNA associated (S. cerevisiae)	naa38	3.63E-02	-1.173
ILMN_2426965	NM_022314	tropomyosin 3, gamma (Tpm3), mRNA. (S)	Tpm3	predicted gene 7848; predicted gene 7839; predicted gene 4157; similar to tropomyosin 3, gamma; tropomyosin 3, gamma; predicted gene 4903	Gm7839	3.61E-02	-1.172
ILMN_2639498	NM_026424	NaN (S)	1500041J02Rik	hypothetical protein LOC675736; coenzyme Q10 homolog B (S. cerevisiae); predicted gene 4899	Gm4899	1.78E-02	-1.172
ILMN_2813712	NM_026203	Abelson helper integration site (Ahi1), mRNA. (S)	Ahi1	Abelson helper integration site 1	Ahi1	4.17E-02	-1.172
ILMN_2529177	XM_193784	NaN (S)	LOC268782	n/a	n/a	4.17E-02	-1.172

ILMN_1257510	XM_001479207	PREDICTED: similar to Kifc1 protein (LOC100042970), mRNA. (S)	LOC100042970	similar to Kifc1 protein; kinesin family member C1; predicted gene 4137	KIFC1	3.62E-02	-1.170
ILMN_2846148	NM_133351	protease, serine, 8 (prostasin) (Prss8), mRNA. (S)	Prss8	protease, serine, 8 (prostasin)	PRSS8	3.71E-02	-1.169
ILMN_2762026	NM_011045	proliferating cell nuclear antigen (Pcna), mRNA. (S)	Pcna	proliferating cell nuclear antigen; similar to proliferating cell nuclear antigen (DNA polymerase delta auxiliary protein)	pcnA	2.53E-02	-1.169
ILMN_1239662	NM_009207	solute carrier family 4 (anion exchanger), member 2 (Slc4a2), mRNA. (S)	Slc4a2	solute carrier family 4 (anion exchanger), member 2	SLC4A2	4.55E-02	-1.168
ILMN_1252687	XM_359029	NaN (S)	LOC386002	n/a	n/a	4.36E-02	-1.167
ILMN_2751771	NM_134069	solute carrier family 17 (sodium phosphate), member 3 (Slc17a3), mRNA. (S)	Slc17a3	solute carrier family 17 (sodium phosphate), member 3	SLC17A3	7.87E-03	-1.166
ILMN_2735792	NM_011417	SWI/SNF related, matrix associated, actin dependent regulator of chromatin, subfamily a, member 4 (Smarca4), mRNA. (S)	Smarca4	SWI/SNF related, matrix associated, actin dependent regulator of chromatin, subfamily a, member 4	SMARCA4	3.43E-02	-1.166
ILMN_2687437	NM_021398	NaN (S)	Slc43a3	solute carrier family 43, member 3	Slc43a3	2.50E-02	-1.165
ILMN_2620131	NM_026437	RIKEN cDNA 1810055E12 gene (1810055E12Rik), mRNA. (S)	1810055E12Rik	RIKEN cDNA 1810055E12 gene	FAM45A	8.21E-03	-1.164
ILMN_1220423	NM_176968	5'-nucleotidase domain containing 1 (Nt5dc1), mRNA. (S)	Nt5dc1	5'-nucleotidase domain containing 1	Nt5dc1	3.09E-02	-1.164
ILMN_1244904	AK014118	NaN (S)	3110033D18Rik	methionine aminopeptidase-like 1	Metapl1	2.75E-03	-1.163
ILMN_1249526	AK020411	NaN (S)	Bmp7	bone morphogenetic protein 7	bmp7	2.29E-02	-1.163
ILMN_2736190	NM_146512	olfactory receptor 97 (Olfr97), mRNA. (S)	Olfr97	olfactory receptor 97	Olfr97	4.25E-02	-1.163
ILMN_3125651	NM_001039353	nucleolar and coiled-body phosphoprotein 1 (Nolc1), transcript variant 4, mRNA. (A)	Nolc1	nucleolar and coiled-body phosphoprotein 1	NOLC1	4.33E-02	-1.161
ILMN_1245581	XM_357173	NaN (S)	LOC383654	n/a	n/a	3.92E-03	-1.155

ILMN_2651005	NM_010155	Ets2 repressor factor (Erf), mRNA. (S)	Erf	Ets2 repressor factor	erF	4.55E-02	-1.155
ILMN_2523012	NM_144513	NaN (S)	Gtl2	n/a	n/a	1.22E-02	-1.155
ILMN_3014257	NM_029334	zinc finger CCCH type containing 14 (Zc3h14), transcript variant 1, mRNA. (I)	Zc3h14	zinc finger CCCH type containing 14	Zc3h14	3.94E-02	-1.154
ILMN_1240667	AK041418	NaN (S)	Rin	Ras-like without CAAX 2	RIT2	3.01E-02	-1.154
ILMN_1241993	AK085320	NaN (S)	Vcam1	vascular cell adhesion molecule 1	Vcam1	1.05E-02	-1.152
ILMN_2532269	XR_031704	PREDICTED: similar to S-adenosylhomocysteine hydrolase (LOC434788), misc RNA. (S)	LOC434788	predicted gene 5638	Gm5638	2.77E-02	-1.152
ILMN_1220712	AK034671	NaN (S)	9430022P05Rik	jagunal homolog 1 (Drosophila)	JAGN1	2.27E-02	-1.152
ILMN_1215265	NaN	NaN (S)	5730507C05Rik	n/a	n/a	2.08E-02	-1.152
ILMN_2445926	NM_001033209	xylulokinase homolog (H. influenzae) (Xylb), mRNA. XM_902953 XM_902954 XM_916628 XM_924356 XM_924359 XM_924362 XM_924365 (S)	Xylb	xylulokinase homolog (H. influenzae)	xylB	1.95E-02	-1.151
ILMN_1252301	AK041006	NaN (S)	A530060E10Rik	n/a	n/a	1.39E-02	-1.151
ILMN_2634078	XM_122906	NaN (S)	Racgap1	n/a	n/a	2.02E-02	-1.151
ILMN_1213422	AK033889	NaN (S)	Mmd	monocyte to macrophage differentiation-associated; similar to monocyte to macrophage differentiation-associated	MMD	4.22E-02	-1.150
ILMN_2614203	NM_177054	cancer susceptibility candidate 4 (Casc4), transcript variant 1, mRNA. (S)	Casc4	cancer susceptibility candidate 4	CASC4	2.90E-02	-1.150
ILMN_2616274	NM_199322	DOT1-like, histone H3 methyltransferase (S. cerevisiae) (Dot1l), mRNA. (S)	Dot1l	DOT1-like, histone H3 methyltransferase (S. cerevisiae)	Dot1l	2.48E-02	-1.150

ILMN_1241429	XM_154052	NaN (S)	LOC218165	n/a	n/a	1.73E-02	-1.149
ILMN_2499646	AK129287	NaN (S)	mKIAA1090	Wolf-Hirschhorn syndrome candidate 1 (human)	Whsc1	2.87E-02	-1.149
ILMN_2637203	NM_028128	replication factor C (activator 1) 5 (Rfc5), mRNA. (S)	Rfc5	replication factor C (activator 1) 5; similar to replication factor C 5	RFC5	1.61E-02	-1.149
ILMN_2906855	NM_024291	kyphoscoliosis peptidase (Ky), mRNA. (S)	Ky	kyphoscoliosis peptidase	KY	2.86E-03	-1.149
ILMN_2933022	NM_013746	pleckstrin homology domain containing, family B (evectins) member 1 (Plekhb1), mRNA. (S)	Plekhb1	pleckstrin homology domain containing, family B (evectins) member 1	PLEKHB1	1.60E-02	-1.149
ILMN_2855551	NM_001081387	CCCTC-binding factor (zinc finger protein)-like (Ctcfl), mRNA. (S)	Ctcfl	CCCTC-binding factor (zinc finger protein)-like	CTCFL	2.36E-02	-1.148
ILMN_1245131	NM_198035	zinc finger and BTB domain containing 39 (Zbtb39), mRNA. (S)	Zbtb39	zinc finger and BTB domain containing 39	ZBTB39	1.14E-02	-1.148
ILMN_2704048	NM_025618	sorcin (Sri), transcript variant 2, mRNA. (S)	Sri	sorcin	SRI	3.92E-02	-1.148
ILMN_2628339	NM_001083334	bridging integrator 1 (Bin1), transcript variant 2, mRNA. (S)	Bin1	bridging integrator 1	bin1	8.95E-03	-1.145
ILMN_2940417	NM_001013616	tripartite motif-containing 6 (Trim6), mRNA. (S)	Trim6	tripartite motif-containing 6; similar to Tripartite motif protein 6	TRIM6	1.93E-02	-1.145
ILMN_1243166	XM_981565	PREDICTED: expressed sequence C87414, transcript variant 6 (C87414), mRNA. (S)	C87414	predicted gene 3302; similar to Expressed sequence C87414; predicted gene 6367; predicted gene 3259; predicted gene 7919; similar to PRAME family member 9; expressed sequence C87414; predicted gene 3089	LOC621779	2.55E-02	-1.144
ILMN_1241580	XM_139579	NaN (S)	LOC239392	n/a	n/a	4.06E-02	-1.143
ILMN_1222084	NM_080726	rad and gem related GTP binding protein 2 (Rem2), mRNA. (S)	Rem2	rad and gem related GTP binding protein 2	Rem2	1.97E-02	-1.141
ILMN_1241523	AK032604	NaN (S)	Ptprs	protein tyrosine phosphatase, receptor type, S	Ptprs	4.52E-02	-1.141
ILMN_2795677	NM_001033457	gene model 1040, (NCBI) (Gm1040), mRNA. (S)	Gm1040	nucleolar protein with MIF4G domain 1	NOM1	4.82E-02	-1.140
ILMN_2621588	NM_025533	nitric oxide synthase interacting protein (Nosip), mRNA. (S)	Nosip	nitric oxide synthase interacting protein	nosip	3.46E-02	-1.138
ILMN_2887104	NM_175181	RIKEN cDNA 2600010E01 gene (2600010E01Rik), mRNA. (S)	2600010E01Rik	proline rich 5 like; hypothetical protein LOC100048398	Prr5l	2.23E-02	-1.138

ILMN_1239587	XM_205152	NaN (S)	LOC278041	n/a	n/a	1.79E-02	-1.137
ILMN_1230743	NM_178746	solute carrier family 38, member 9 (Slc38a9), mRNA. (S)	Slc38a9	solute carrier family 38, member 9	slc38a9	3.07E-02	-1.137
ILMN_2510260	NM_025783	vacuolar protein sorting 24 (yeast) (Vps24), mRNA. (S)	Vps24	vacuolar protein sorting 24 (yeast); predicted gene 7464	Gm7464	3.47E-02	-1.137
ILMN_2606198	NM_175275	centlein, centrosomal protein (Cntln), transcript variant 1, mRNA. (S)	Cntln	centlein, centrosomal protein	Cntln	2.80E-02	-1.137
ILMN_2773467	XM_001478948	PREDICTED: similar to Ezh1 protein (LOC100044129), mRNA. (S)	LOC100044129	enhancer of zeste homolog 1 (Drosophila)	EZH1	4.05E-02	-1.137
ILMN_1253915	AK012463	NaN (S)	Psmd7	proteasome (prosome, macropain) 26S subunit, non-ATPase, 7	psmd7	2.97E-02	-1.136
ILMN_3154335	NM_026562	cyclin N-terminal domain containing 1 (Cntd1), mRNA. (A)	Cntd1	similar to cyclin N-terminal domain containing 1; cyclin N-terminal domain containing 1	LOC100044557	4.82E-02	-1.134
ILMN_2478748	NM_001081011	SLIT-ROBO Rho GTPase activating protein 2 (Srgap2), mRNA. (S)	Srgap2	SLIT-ROBO Rho GTPase activating protein 2	srgap2	1.10E-02	-1.134
ILMN_2541970	XM_356536	NaN (S)	LOC382501	n/a	n/a	3.21E-02	-1.134
ILMN_2853212	NM_023712	spinster homolog 1 (Drosophila) (Spns1), mRNA. (S)	Spns1	spinster homolog 1 (Drosophila)	spns1	3.51E-02	-1.133
ILMN_1245507	XM_142805	NaN (S)	LOC236260	n/a	n/a	5.16E-03	-1.133
ILMN_1231517	XM_130845	NaN (S)	E430012K20Rik	n/a	n/a	2.10E-02	-1.132
ILMN_2611205	NM_181818	olfactory receptor 141 (Olfr141), mRNA. (S)	Olfr141	olfactory receptor 141	Olfr141	2.76E-02	-1.132
ILMN_2634358	NM_008628	mutS homolog 2 (E. coli) (Msh2), mRNA. (S)	Msh2	mutS homolog 2 (E. coli)	Msh2	4.75E-02	-1.131
ILMN_2547571	XR_033404	PREDICTED: hypothetical protein LOC100047486 (LOC100047486), misc RNA. (S)	LOC100047486	hypothetical protein LOC100047486; predicted gene 14719; ancreatic progenitor cell differentiation and proliferation factor homolog (zebrafish)RIKEN cDNA 2700038C09 gene	Gm14719	2.51E-02	-1.131
ILMN_3004177	NM_027918	RIKEN cDNA 1300017J02 gene (1300017J02Rik), mRNA. (S)	1300017J02Rik	RIKEN cDNA 1300017J02 gene	1300017J02Rik	2.18E-03	-1.130
ILMN_2486419	NM_134213	vomeronasal 1 receptor, H4 (V1rh4), mRNA. (S)	V1rh4	vomeronasal 1 receptor, H4	Vmn1r199	1.81E-02	-1.130

ILMN_3105313	NM_001034857	RIKEN cDNA C330019L16 gene (C330019L16Rik), mRNA. XM_924349 (A)	C330019L16Rik	RIKEN cDNA C330019L16 gene	C330019L16Rik	2.81E-02	-1.130
ILMN_2798973	NM_008665	myelin transcription factor 1 (Myt1), mRNA. (S)	Myt1	myelin transcription factor 1	Myt1	1.88E-02	-1.130
ILMN_1254421	XM_001476775	PREDICTED: hypothetical protein LOC100046690 (LOC100046690), mRNA. (S)	LOC100046690	ankyrin 1, erythroid; hypothetical protein LOC100046690	ank1	1.34E-02	-1.130
ILMN_1221784	NM_172119	NaN (S)	Dio3	deiodinase, iodothyronine type III	dio3	2.71E-03	-1.129
ILMN_2749280	NM_019985	C-type lectin domain family 1, member b (Clec1b), mRNA. (S)	Clec1b	C-type lectin domain family 1, member b	Clec1b	2.09E-02	-1.129
ILMN_2661168	NM_175308	MOB1, Mps One Binder kinase activator-like 2C (yeast) (Mobkl2c), mRNA. (S)	Mobkl2c	MOB1, Mps One Binder kinase activator-like 2C (yeast)	Mobkl2c	2.49E-02	-1.128
ILMN_1226845	AK079439	NaN (S)	9930022E02Rik	zinc finger protein 81	Zfp81	3.51E-02	-1.127
ILMN_2699078	NM_025323	RIKEN cDNA 0610009D07 gene (0610009D07Rik), mRNA. (S)	0610009D07Rik	RIKEN cDNA 0610009D07 gene	0610009D07Rik	2.72E-02	-1.127
ILMN_1236881	NM_009671	NaN (S)	Ankfy1	ankyrin repeat and FYVE domain containing 1	Ankfy1	4.42E-02	-1.126
ILMN_1225682	NM_009766	bombesin-like receptor 3 (Brs3), mRNA. (S)	Brs3	bombesin-like receptor 3	BRS3	8.55E-03	-1.125
ILMN_3155994	NM_009158	mitogen-activated protein kinase 10 (Mapk10), transcript variant 1, mRNA. (A)	Mapk10	mitogen-activated protein kinase 10	MAPK10	4.77E-02	-1.124
ILMN_1239371	NM_007466	NaN (S)	Api5	apoptosis inhibitor 5	API5	3.43E-02	-1.123
ILMN_2929813	NM_147022	olfactory receptor 381 (Olfr381), mRNA. (S)	Olfr381	olfactory receptor 381	Olfr381	9.38E-03	-1.122
ILMN_1245767	NM_007615	catenin (cadherin associated protein), delta 1 (Ctnnd1), transcript variant 1, mRNA. (S)	Ctnnd1	catenin (cadherin associated protein), delta 1	CTNND1	3.20E-02	-1.121
ILMN_3141738	NM_198311	tetratricopeptide repeat domain 8 (Ttc8), transcript variant 2, mRNA. (A)	Ttc8	tetratricopeptide repeat domain 8	TTC8	4.44E-02	-1.121
ILMN_1241948	AK088875	NaN (S)	Ptprc	protein tyrosine phosphatase, receptor type, C	Ptprc	2.78E-04	-1.121

ILMN_2738750	NM_016677	hippocalcin-like 1 (Hpcal1), mRNA. (S)	Hpcal1	hippocalcin-like 1	hpcal1	1.54E-02	-1.120
ILMN_2473090	NM_025710	ubiquinol-cytochrome c reductase, Rieske iron-sulfur polypeptide 1 (Uqcrfs1), mRNA. (S)	Uqcrfs1	ubiquinol-cytochrome c reductase, Rieske iron-sulfur polypeptide 1	uqcrfs1	4.68E-02	-1.120
ILMN_1229977	NM_009986	cut-like homeobox 1 (Cux1), transcript variant 1, mRNA. (S)	Cux1	cut-like homeobox 1	CUX1	3.64E-02	-1.120
ILMN_1231399	AK045787	NaN (S)	Clcn6	chloride channel 6	CLCN6	3.86E-02	-1.119
ILMN_1248328	NM_009612	activin A receptor, type II-like 1 (Acvrl1), mRNA. (S)	Acvrl1	activin A receptor, type II-like 1	Acvrl1	2.10E-02	-1.119
ILMN_2627503	NM_021882	silver (Si), mRNA. (S)	Si	silver	SI	3.31E-02	-1.119
ILMN_2677824	NM_144796	sushi domain containing 4 (Susd4), mRNA. (S)	Susd4	sushi domain containing 4	Susd4	4.29E-02	-1.119
ILMN_2755241	NM_023284	NaN (S)	Cdca1	similar to Nuf2 protein; NUF2, NDC80 kinetochore complex component, homolog (S. cerevisiae)	NUF2	1.78E-02	-1.119
ILMN_1222328	XM_359039	NaN (S)	LOC386039	n/a	n/a	3.71E-02	-1.119
ILMN_1238243	NM_008626	NaN (S)	Mrc2	mannose receptor, C type 2	Mrc2	2.63E-02	-1.118
ILMN_1256849	NM_178048	ST3 beta-galactoside alpha-2,3-sialyltransferase 2 (St3gal2), transcript variant 2, mRNA. (S)	St3gal2	ST3 beta-galactoside alpha-2,3-sialyltransferase 2	ST3GAL2	2.99E-02	-1.118
ILMN_2759004	NM_172474	tRNA-yW synthesizing protein 3 homolog (S. cerevisiae) (Tyw3), mRNA. (S)	Tyw3	tRNA-yW synthesizing protein 3 homolog (S. cerevisiae)	TYW3	3.27E-02	-1.118
ILMN_1236741	NM_001015876	tRNA-yW synthesizing protein 1 homolog (S. cerevisiae) (Tyw1), transcript variant 1, mRNA. (S)	Tyw1	tRNA-yW synthesizing protein 1 homolog (S. cerevisiae)	TYW1	1.82E-02	-1.118
ILMN_1260567	NM_009391	RAN, member RAS oncogene family (Ran), mRNA. (I)	Ran	RAS-like, family 2, locus 9; RAN, member RAS oncogene family; similar to RAN, member RAS oncogene family	LOC100045999	5.98E-03	-1.118
ILMN_3161245	NM_001003670	predicted gene, EG406223 (EG406223), mRNA. (S)	EG406223	predicted gene 5414	Gm5414	2.11E-02	-1.118
ILMN_2590815	NM_001008231	dishevelled associated activator of morphogenesis 2 (Daam2), mRNA. (S)	Daam2	dishevelled associated activator of morphogenesis 2	DAAM2	3.21E-02	-1.117

ILMN_2988275	NM_029404	PHD finger protein 14 (Phf14), mRNA. (S)	Phf14	PHD finger protein 14	PHF14	2.90E-02	-1.117
ILMN_2873422	NM_146656	olfactory receptor 444 (Olfr444), mRNA. (S)	Olfr444	olfactory receptor 444	Olfr444	2.10E-02	-1.116
ILMN_2655112	NM_008840	phosphatidylinositol 3-kinase catalytic delta polypeptide (Pik3cd), transcript variant 1, mRNA. (S)	Pik3cd	phosphatidylinositol 3-kinase catalytic delta polypeptide; RIKEN cDNA 2610208K16 gene	PIK3CD	8.39E-03	-1.116
ILMN_2780247	NM_010735	lymphotoxin A (Lta), mRNA. (S)	Lta	lymphotoxin A	lta	1.17E-02	-1.116
ILMN_1237285	AK042443	NaN (S)	A630092E18Rik	WD repeat and HMG-box DNA binding protein 1	Wdhd1	3.90E-02	-1.115
ILMN_1254895	AK042147	NaN (S)	Prlr	prolactin receptor	PRLR	1.89E-02	-1.115
ILMN_1237070	XM_359010	NaN (S)	LOC385952	n/a	n/a	1.41E-02	-1.115
ILMN_2932081	NM_008634	microtubule-associated protein 1 B (Mtap1b), mRNA. (S)	Mtap1b	microtubule-associated protein 1B	Mtap1b	2.34E-02	-1.115
ILMN_2772523	NM_029797	NaN (S)	2610034E18Rik	meiotic nuclear divisions 1 homolog (S. cerevisiae); predicted gene 3833; similar to Meiotic nuclear divisions 1 homolog (S. cerevisiae)	Gm3833	1.84E-02	-1.114
ILMN_2646078	NM_201601	fibroblast growth factor receptor 2 (Fgfr2), transcript variant 2, mRNA. (S)	Fgfr2	fibroblast growth factor receptor 2	FGFR2	9.24E-03	-1.114
ILMN_2540212	XM_358223	NaN (S)	LOC385395	n/a	n/a	2.86E-02	-1.114
ILMN_1220900	XM_911699	PREDICTED: WD repeat domain 81 (Wdr81), mRNA. (S)	Wdr81	WD repeat domain 81	WDR81	3.47E-02	-1.113
ILMN_2736639	NM_173185	casein kinase 1, gamma 1 (Csnk1g1), mRNA. (S)	Csnk1g1	casein kinase 1, gamma 1	CSNK1G1	4.71E-02	-1.113
ILMN_2764551	NM_183405	cytochrome c oxidase subunit VIb polypeptide 2 (Cox6b2), mRNA. (S)	Cox6b2	cytochrome c oxidase subunit VIb polypeptide 2	Cox6b2	2.98E-02	-1.113
ILMN_1243845	NM_025581	RIKEN cDNA 2810433K01 gene (2810433K01Rik), mRNA. (S)	2810433K01Rik	RIKEN cDNA 2810433K01 gene	Ska1	3.18E-02	-1.112
ILMN_2755224	NM_028023	cell division cycle associated 4 (Cdca4), mRNA. (S)	Cdca4	cell division cycle associated 4	CDCA4	4.19E-02	-1.112
ILMN_2522532	XM_485619	NaN (S)	D830007B15Rik	n/a	n/a	1.76E-02	-1.111

ILMN_2806315	NM_001037714	gene model 867, (NCBI) (Gm867), mRNA. (S)	Gm867	predicted gene 867	Gm867	1.76E-02	-1.111
ILMN_1241061	XM_001004827	PREDICTED: similar to MAX protein isoform a (LOC546929), mRNA. (S)	LOC546929	n/a	n/a	1.97E-02	-1.110
ILMN_2493231	XM_204114	NaN (S)	Zswim5	n/a	n/a	2.80E-02	-1.110
ILMN_2713055	NM_028216	prostate stem cell antigen (Psca), mRNA. (S)	Psca	prostate stem cell antigen	PSCA	3.03E-02	-1.110
ILMN_1238562	NM_008743	nth (endonuclease III)-like 1 (E.coli) (Nthl1), mRNA. (S)	Nthl1	nth (endonuclease III)-like 1 (E.coli)	nthl1	4.51E-02	-1.109
ILMN_1257750	NM_010838	microtubule-associated protein tau (Mapt), transcript variant 2, mRNA. (S)	Mapt	microtubule-associated protein tau	mapt	2.22E-02	-1.109
ILMN_1255998	XR_032462	PREDICTED: similar to PIRA2 (LOC100041137), misc RNA. (S)	LOC100041137	hypothetical protein LOC100038909; similar to PIRA2; hypothetical protein LOC100038908; predicted gene 15448; paired-Ig-like receptor A11; paired-Ig-like receptor A5; paired-Ig-like receptor A6; paired-Ig-like receptor A4; paired-Ig-like receptor A7; predicted gene 15930; paired-Ig-like receptor A1; paired-Ig-like receptor A2; predicted gene 10693	Pira6	2.15E-02	-1.108
ILMN_1254215	NM_133986	NaN (S)	Tcta	T-cell leukemia translocation altered gene	tctA	3.32E-02	-1.108
ILMN_1256755	NM_177034	amyloid beta (A4) precursor protein binding, family A, member 1 (Apba1), mRNA. (S)	Apba1	amyloid beta (A4) precursor protein binding, family A, member 1	apbA1	3.30E-02	-1.107
ILMN_1225102	NM_011223	paxillin (Pxn), transcript variant alpha, mRNA. (S)	Pxn	paxillin	PXN	1.52E-02	-1.107
ILMN_2659523	NM_153153	supervillin (Svil), transcript variant 1, mRNA. (S)	Svil	supervillin	SVIL	3.73E-02	-1.106
ILMN_2719346	NM_023504	NK2 transcription factor related, locus 4 (Drosophila) (Nkx2-4), mRNA. (S)	Nkx2-4	NK2 transcription factor related, locus 4 (Drosophila)	Nkx2-4	4.86E-02	-1.106
ILMN_2971073	NM_026486	RIKEN cDNA 4432405B04 gene (4432405B04Rik), mRNA. (S)	4432405B04Rik	tectonic family member 2	TCTN2	1.88E-02	-1.105
ILMN_2693509	NM_172509	potassium channel tetramerisation domain containing 7 (Kctd7), mRNA. (S)	Kctd7	potassium channel tetramerisation domain containing 7	KCTD7	3.67E-02	-1.105

ILMN_2803258	NM_027819	gamma-glutamyltransferase 6 (Ggt6), mRNA. (S)	Ggt6	gamma-glutamyltransferase 6	GGT6	5.47E-03	-1.104
ILMN_2548664	NM_053083	lysyl oxidase-like 4 (Loxl4), mRNA. (S)	Loxl4	lysyl oxidase-like 4	Loxl4	7.19E-03	-1.104
ILMN_2620844	NM_133840	CLP1, cleavage and polyadenylation factor I subunit, homolog (S. cerevisiae) (Clp1), mRNA. (S)	Clp1	CLP1, cleavage and polyadenylation factor I subunit, homolog (S. cerevisiae)	clp1	2.69E-02	-1.103
ILMN_1218449	AK080894	NaN (S)	Srb1	scavenger receptor class B, member 1	SCARB1	3.53E-02	-1.103
ILMN_1218833	XM_001471664	PREDICTED: hypothetical protein LOC100038908 (LOC100038908), mRNA. (S)	LOC100038908	hypothetical protein LOC100038909; similar to PIRA2; hypothetical protein LOC100038908; predicted gene 15448; paired-Ig-like receptor A11; paired-Ig-like receptor A5; paired-Ig-like receptor A6; paired-Ig-like receptor A4; paired-Ig-like receptor A7; predicted gene 15930; paired-Ig-like receptor A1; paired-Ig-like receptor A2; predicted gene 10693	Pira6	4.53E-02	-1.103
ILMN_1251978	XM_358249	NaN (S)	LOC385476	n/a	n/a	1.99E-02	-1.102
ILMN_2873220	NM_146797	olfactory receptor 1502 (Olfr1502), mRNA. (S)	Olfr1502	olfactory receptor 1502	Olfr1502	3.89E-02	-1.102
ILMN_1229609	NM_025594	zinc finger, matrin type 2 (Zmat2), mRNA. (S)	Zmat2	zinc finger, matrin type 2	zmat2	3.48E-02	-1.102
ILMN_1252446	NM_031174	Down syndrome cell adhesion molecule (Dscam), mRNA. (S)	Dscam	Down syndrome cell adhesion molecule	dscam	4.75E-02	-1.101
ILMN_1221895	NM_145414	NOL1/NOP2/Sun domain family, member 5 (Nsun5), mRNA. (S)	Nsun5	NOL1/NOP2/Sun domain family, member 5	Nsun5	9.88E-03	-1.101
ILMN_1240857	NM_009944	cytochrome c oxidase, subunit VIIa 1 (Cox7a1), mRNA. (S)	Cox7a1	cytochrome c oxidase, subunit VIIa 1	COX7A1	1.50E-02	-1.100
ILMN_2706410	NM_011028	purinergic receptor P2X, ligand-gated ion channel, 6 (P2rx6), mRNA. (S)	P2rx6	purinergic receptor P2X, ligand-gated ion channel, 6	P2RX6	5.32E-03	-1.098

ILMN_3005175	NM_013794	killer cell lectin-like receptor, subfamily A, member 16 (Klra16), mRNA. (S)	Klra16	killer cell lectin-like receptor, subfamily A, member 4; killer cell lectin-like receptor subfamily A member 33; Ly49p/d; similar to Killer cell lectin-like receptor 4 (T-cell surface glycoprotein Ly-49D) (Ly49-D antigen) (Lymphocyte antigen 49D); killer cell lectin-like receptor, subfamily A, member 18; killer cell lectin-like receptor, subfamily A, member 25; killer cell lectin-like receptor, subfamily A, member 16; hypothetical protein LOC100044241	LOC635513	7.48E-03	-1.098
ILMN_2474515	NM_001081963	RIKEN cDNA 9430020K01 gene (9430020K01Rik), mRNA. (S)	9430020K01Rik	RIKEN cDNA 9430020K01 gene	9430020K01Rik	4.19E-02	-1.098
ILMN_1257845	XM_287254	NaN (S)	LOC329664	n/a	n/a	3.77E-02	-1.098
ILMN_2710597	NM_007567	bassoon (Bsn), mRNA. (S)	Bsn	bassoon	BSN	3.28E-02	-1.097
ILMN_2740838	XM_001476987	PREDICTED: similar to brain protein 44-like protein, transcript variant 1 (LOC100041649), mRNA. (S)	LOC100041649	similar to brain protein 44-like protein; brain protein 44-like; predicted gene 3452; predicted gene 8219	Gm8219	3.36E-02	-1.097
ILMN_2962364	NM_027530	RUN and FYVE domain containing 3 (Rufy3), mRNA. (S)	Rufy3	RUN and FYVE domain containing 3	RUFY3	4.92E-02	-1.097
ILMN_2740062	NM_146912	olfactory receptor 211 (Olfr211), mRNA. (S)	Olfr211	olfactory receptor 211	Olfr211	4.29E-02	-1.097
ILMN_2545184	AK014461	NaN (S)	3930402I10Rik	kinesin family member 15	KIF15	2.67E-02	-1.096
ILMN_2597034	NM_019799	Rhesus blood group-associated C glycoprotein (Rhcg), mRNA. (S)	Rhcg	Rhesus blood group-associated C glycoprotein	RHCG	4.97E-02	-1.094
ILMN_3162152	NM_001013385	glutamate receptor, metabotropic 4 (Grm4), mRNA. (S)	Grm4	glutamate receptor, metabotropic 4	Grm4	2.59E-02	-1.092
ILMN_2808751	NM_029933	B-cell CLL/lymphoma 9 (Bcl9), mRNA. (S)	Bcl9	B-cell CLL/lymphoma 9	BCL9	2.92E-02	-1.092
ILMN_2887075	NM_007387	acid phosphatase 2, lysosomal (Acp2), mRNA. (S)	Acp2	acid phosphatase 2, lysosomal	ACP2	3.58E-02	-1.090
ILMN_3161298	NM_001011859	olfactory receptor 965 (Olfr965), mRNA. (S)	Olfr965	olfactory receptor 965	Olfr965	3.98E-03	-1.089

ILMN_1240997	NM_001039365	myelin-associated oligodendrocytic basic protein (Mobp), transcript variant 3, mRNA. (S)	Mobp	myelin-associated oligodendrocytic basic protein	MOBP	2.43E-02	-1.089
ILMN_2555632	AK046540	NaN (S)	Asah2	N-acylsphingosine amidohydrolase 2	ASAH2	3.91E-02	-1.088
ILMN_3011294	NM_001012517	fucosyltransferase 10 (Fut10), transcript variant A, mRNA. (I)	Fut10	fucosyltransferase 10	fut10	2.88E-02	-1.088
ILMN_2944272	NM_174847	RIKEN cDNA 5830404H04 gene (5830404H04Rik), mRNA. (S)	5830404H04Rik	C2 calcium-dependent domain containing 2	C2CD2	3.14E-02	-1.088
ILMN_1213093	NM_146832	olfactory receptor 134 (Olfr134), mRNA. (S)	Olfr134	olfactory receptor 134	Olfr134	4.82E-02	-1.088
ILMN_2956973	NM_173014	lysophosphatidylcholine acyltransferase 2 (Lpcat2), mRNA. (S)	Lpcat2	lysophosphatidylcholine acyltransferase 2	lpcat2	2.66E-02	-1.087
ILMN_1241057	XM_001004201	PREDICTED: START domain containing 9 (Stard9), mRNA. (S)	Stard9	START domain containing 9	STARD9	4.18E-02	-1.086
ILMN_1258693	AK043289	NaN (S)	Csmd1	n/a	n/a	4.31E-02	-1.086
ILMN_1217702	NM_026148	LIM and senescent cell antigen-like domains 1 (Lims1), mRNA. (S)	Lims1	LIM and senescent cell antigen-like domains 1	LIMS1	1.93E-02	-1.085
ILMN_1255122	AK053504	NaN (S)	P4ha2	procollagen-proline, 2-oxoglutarate 4-dioxygenase (proline 4-hydroxylase), alpha II polypeptide	P4HA2	1.87E-02	-1.085
ILMN_2740622	NM_008760	osteoglycin (Ogn), mRNA. (S)	Ogn	osteoglycin	OGN	2.77E-02	-1.085
ILMN_1257951	XM_286414	PREDICTED: hypothetical LOC328082 (LOC328082), mRNA. (S)	LOC328082	predicted gene, EG328082	EG328082	3.53E-02	-1.085
ILMN_2677217	XM_001477261	PREDICTED: RIKEN cDNA 1700023L04 gene (1700023L04Rik), mRNA. (S)	1700023L04Rik	RIKEN cDNA 1700023L04 gene	1700023L04Rik	4.94E-02	-1.084
ILMN_2540952	XM_356027	NaN (S)	LOC381981	n/a	n/a	7.17E-03	-1.084
ILMN_3042991	NM_001035510	zinc finger, CCHC domain containing 18 (Zcchc18), transcript variant 1, mRNA. (I)	Zcchc18	zinc finger, CCHC domain containing 18	Zcchc18	4.53E-02	-1.083
ILMN_1257212	NM_206882	histone cluster 3, H2bb (Hist3h2bb), mRNA. (S)	Hist3h2bb	histone cluster 3, H2ba; histone cluster 3, H2bb	Hist3h2ba	3.94E-03	-1.083

ILMN_1252865	AK051031	NaN (S)	Zfp46	zinc finger protein 46	Zfp46	1.11E-02	-1.083
ILMN_1253940	XM_143590	NaN (S)	LOC214456	n/a	n/a	2.76E-02	-1.083
ILMN_1233889	NM_025443	partner of NOB1 homolog (S. cerevisiae) (Pno1), mRNA. (S)	Pno1	partner of NOB1 homolog (S. cerevisiae)	pno1	2.05E-02	-1.083
ILMN_2643021	NM_175564	transmembrane protein 169 (Tmem169), mRNA. (S)	Tmem169	transmembrane protein 169	TMEM169	2.83E-02	-1.082
ILMN_2593338	NM_025914	ARP6 actin-related protein 6 homolog (yeast) (Actr6), mRNA. (S)	Actr6	ARP6 actin-related protein 6 homolog (yeast)	ACTR6	3.34E-02	-1.082
ILMN_3137899	NM_178139	otopetrin 1 (Otop1), transcript variant b, mRNA. (A)	Otop1	otopetrin 1	OTOP1	4.63E-02	-1.081
ILMN_2669088	XM_001473469	PREDICTED: RIKEN cDNA 4930461P20 gene (4930461P20Rik), mRNA. (S)	4930461P20Rik	DnaJ (Hsp40) homolog, subfamily C, member 21	Dnajc21	2.25E-02	-1.081
ILMN_2604521	XM_150115	NaN (S)	Cotl1	n/a	n/a	3.90E-02	-1.081
ILMN_1230724	NM_001013759	growth arrest-specific 2 like 2 (Gas2l2), mRNA. (S)	Gas2l2	growth arrest-specific 2 like 2	Gas2l2	1.31E-02	-1.081
ILMN_2704027	NM_001081048	solute carrier family 25 (mitochondrial carrier), member 18 (Slc25a18), mRNA. (S)	Slc25a18	solute carrier family 25 (mitochondrial carrier), member 18	Slc25a18	4.31E-02	-1.080
ILMN_1222051	XM_205168	NaN (S)	LOC278062	n/a	n/a	2.36E-02	-1.080
ILMN_1233385	AK046603	NaN (S)	B430203G13Rik	RIKEN cDNA B430203G13 gene	B430203G13Rik	3.19E-02	-1.080
ILMN_3076219	NM_019585	espin (Espn), transcript variant 6, mRNA. (I)	Espn	espin	Espn	4.13E-02	-1.080
ILMN_2726837	NM_008726	natriuretic peptide precursor type B (Nppb), mRNA. (S)	Nppb	natriuretic peptide precursor type B	nppb	3.93E-02	-1.078
ILMN_2603760	NM_028500	calreticulin 3 (Calr3), transcript variant 1, mRNA. (S)	Calr3	calreticulin 3	CALR3	3.81E-02	-1.078
ILMN_2593515	NM_008877	plasminogen (Plg), mRNA. (S)	Plg	plasminogen	plg	1.12E-02	-1.074
ILMN_1258226	XM_358067	NaN (S)	LOC385118	n/a	n/a	3.40E-02	-1.073

ILMN_3162354	NM_026376	plexin D1 (Plxnd1), mRNA. (S)	Plxnd1	plexin D1	Plxnd1	2.99E-02	-1.073
ILMN_1254958	AK034620	NaN (S)	9430015G10Rik	RIKEN cDNA 9430015G10 gene	9430015G10Rik	4.26E-02	-1.072
ILMN_2611261	NM_016755	ATP synthase, H+ transporting, mitochondrial F0 complex, subunit F (Atp5j), nuclear gene encoding mitochondrial protein, mRNA. (S)	Atp5j	ATP synthase, H+ transporting, mitochondrial F0 complex, subunit F pseudogene; similar to ATP synthase coupling factor 6, mitochondrial precursor (ATPase subunit F6); ATP synthase, H+ transporting, mitochondrial F0 complex, subunit F	LOC674583	1.44E-02	-1.072
ILMN_2941714	NM_175309	uroplakin 3B (Upk3b), mRNA. (S)	Upk3b	uroplakin 3B	UPK3B	4.97E-02	-1.071
ILMN_2524067	XM_130015	NaN (S)	4933428L19Rik	n/a	n/a	3.17E-02	-1.071
ILMN_1225262	AK078240	NaN (S)	6430531H12Rik	plakophilin 4	PKP4	3.81E-02	-1.071
ILMN_2789047	NM_025377	RIKEN cDNA 1110001A07 gene (1110001A07Rik), mRNA. (S)	1110001A07Rik	predicted gene 6597; RIKEN cDNA 1110001A07 gene	Fam33a	4.76E-02	-1.070
ILMN_1232774	AK032686	NaN (S)	Ptdss2	phosphatidylserine synthase 2	PTDSS2	4.93E-02	-1.068
ILMN_2610342	NM_010613	KH-type splicing regulatory protein (Khsrp), mRNA. (S)	Khsrp	KH-type splicing regulatory protein	khsrp	2.99E-02	-1.067
ILMN_2669886	NM_203660	predicted gene, EG368203 (EG368203), mRNA. (S)	EG368203	n/a	n/a	3.31E-02	-1.064
ILMN_1241131	XM_135479	NaN (S)	LOC236069	predicted gene 13238	Gm13238	3.02E-02	-1.063
ILMN_1242787	AK016166	NaN (S)	4930557M22Rik	sulfide quinone reductase-like (yeast)	SQRDL	2.02E-02	-1.062
ILMN_2726595	NM_175558	zinc finger protein 446 (Zfp446), mRNA. (S)	Zfp446	zinc finger protein 446	Zfp446	2.77E-02	-1.061
ILMN_3103689	NM_029485	RIKEN cDNA 5133400G04 gene (5133400G04Rik), transcript variant 2, mRNA. (A)	5133400G04Rik	RIKEN cDNA 5133400G04 gene	SPATA24	2.36E-02	-1.058
ILMN_2651251	XM_001479265	PREDICTED: transmembrane protein 30C (Tmem30c), mRNA. (S)	Tmem30c	transmembrane protein 30C	Tmem30c	3.01E-02	-1.045
ILMN_2462601	NM_178671	UBX domain protein 10 (Ubxn10), mRNA. (S)	Ubxn10	UBX domain protein 10	Ubxn10	2.07E-02	1.057

ILMN_1226546	NM_183148	NaN (S)	C79267	intermediate filament family orphan 2	IFFO2	3.41E-02	1.057
ILMN_2723052	NM_181577	coiled-coil domain containing 85A (Ccdc85a), mRNA. (S)	Ccdc85a	coiled-coil domain containing 85A	CCDC85A	2.68E-02	1.059
ILMN_2719487	NM_198651	RIKEN cDNA 4430402I18 gene (4430402I18Rik), mRNA. (S)	4430402I18Rik	RIKEN cDNA 4430402I18 gene	4430402I18Rik	3.00E-02	1.059
ILMN_2574465	AK053937	NaN (S)	E230003H01Rik	NIPA-like domain containing 3; similar to NIPA-like domain containing 3	NIPAL3	4.62E-02	1.062
ILMN_1230634	NM_198411	RIKEN cDNA 2610204M08 gene (2610204M08Rik), mRNA. (S)	2610204M08Rik	subacute ozone induced inflammation; RIKEN cDNA 2610204M08 gene	INF2	4.09E-02	1.064
ILMN_2775338	NM_183126	RIKEN cDNA 6030498E09 gene (6030498E09Rik), mRNA. (S)	6030498E09Rik	RIKEN cDNA 6030498E09 gene; hypothetical protein LOC100046882; predicted gene 3231	Gm3231	4.93E-02	1.066
ILMN_2877069	NM_009775	translocator protein (Tspo), mRNA. (S)	Tspo	translocator protein	tspO	3.65E-02	1.067
ILMN_2737338	NM_009231	Son of sevenless homolog 1 (Drosophila) (Sos1), mRNA. (S)	Sos1	son of sevenless homolog 1 (Drosophila)	SOS1	1.75E-02	1.069
ILMN_1214175	XM_136954	NaN (S)	LOC237314	n/a	n/a	1.19E-02	1.070
ILMN_2595106	NM_178614	sorting and assembly machinery component 50 homolog (S. cerevisiae) (Samm50), mRNA. (S)	Samm50	sorting and assembly machinery component 50 homolog (S. cerevisiae)	Samm50	4.32E-02	1.070
ILMN_1218383	AK043580	NaN (S)	A830009P14Rik	kin of IRRE like 3 (Drosophila)	KIRREL3	3.21E-02	1.072
ILMN_2484627	NM_011767	NaN (S)	Zfr	zinc finger RNA binding protein	zfr	4.64E-02	1.075
ILMN_2682865	NM_198223	alveolar soft part sarcoma chromosome region, candidate 1 (human) (Aspscr1), transcript variant 2, mRNA. (S)	Aspscr1	alveolar soft part sarcoma chromosome region, candidate 1 (human)	aspscr1	3.97E-02	1.075
ILMN_2995370	NM_001009927	Smith-Magenis syndrome chromosome region, candidate 7 homolog (human) (Smcr7), mRNA. (S)	Smcr7	Smith-Magenis syndrome chromosome region, candidate 7 homolog (human)	smcr7	3.41E-02	1.075
ILMN_1226193	XM_109598	NaN (S)	1200008N06Rik	n/a	n/a	1.60E-02	1.076
ILMN_2622146	NM_033609	mediator complex subunit 15 (Med15), transcript variant 1, mRNA. (S)	Med15	mediator complex subunit 15	Med15	3.36E-02	1.077

ILMN_1239174	AK006979	NaN (S)	1700082G03Rik	glyoxalase domain containing 4	GLOD4	2.87E-02	1.077
ILMN_2527619	XM_127483	NaN (S)	LOC218476	similar to ankyrin repeat domain 11	ANKRD31	1.28E-02	1.079
ILMN_2560306	AK038972	NaN (S)	A230080D12Rik	transmembrane protein 57	tmem57	2.16E-02	1.079
ILMN_1236221	NM_011264	REV3-like, catalytic subunit of DNA polymerase zeta RAD54 like (S. cerevisiae) (Rev3l), mRNA. (S)	Rev3l	REV3-like, catalytic subunit of DNA polymerase zeta RAD54 like (S. cerevisiae)	REV3L	3.60E-02	1.079
ILMN_1219856	XM_143339	PREDICTED: WD repeat domain 49 (Wdr49), mRNA. (S)	Wdr49	WD repeat domain 49	WDR49	4.06E-02	1.080
ILMN_2774370	NM_175473	Fraser syndrome 1 homolog (human) (Fras1), mRNA. (S)	Fras1	Fraser syndrome 1 homolog (human)	FRAS1	3.41E-02	1.080
ILMN_2892441	NM_010357	glutathione S-transferase, alpha 4 (Gsta4), mRNA. (S)	Gsta4	glutathione S-transferase, alpha 4	GSTA4	2.69E-02	1.081
ILMN_2664702	NM_027304	H1 histone family, member N, testis-specific (H1fnt), mRNA. (S)	H1fnt	H1 histone family, member N, testis-specific	H1fnt	3.43E-02	1.081
ILMN_2754148	NM_001048054	dual specificity phosphatase 16 (Dusp16), transcript variant B1, mRNA. (S)	Dusp16	dual specificity phosphatase 16	DUSP16	1.79E-02	1.081
ILMN_2582964	AK087262	NaN (S)	E030040J22Rik	n/a	n/a	4.27E-02	1.082
ILMN_2745151	NM_028120	coiled-coil domain containing 123 (Ccdc123), mRNA. (S)	Ccdc123	similar to RIKEN cDNA 2610507L03 gene; coiled-coil domain containing 123	CCDC123	3.37E-02	1.082
ILMN_2424680	NM_013499	NaN (S)	Crry	complement component (3b/4b) receptor 1-like	Cr1l	3.33E-03	1.083
ILMN_2798964	NM_178677	SEC22 vesicle trafficking protein-like C (S. cerevisiae) (Sec22c), mRNA. (S)	Sec22c	SEC22 vesicle trafficking protein homolog C (S. cerevisiae)	sec22c	1.84E-02	1.084
ILMN_2764127	XM_001478658	PREDICTED: similar to sulfatase modifying factor 2 (LOC100047675), mRNA. (S)	LOC100047675	similar to sulfatase modifying factor 2; sulfatase modifying factor 2	LOC100047675	3.57E-02	1.084
ILMN_1234177	AK035450	NaN (S)	9530050F08Rik	n/a	n/a	3.47E-02	1.085
ILMN_2813487	NM_011065	period homolog 1 (Drosophila) (Per1), mRNA. (S)	Per1	period homolog 1 (Drosophila)	Per1	1.84E-02	1.085
ILMN_2627280	NM_028712	NaN (S)	Rap2b	RAP2B, member of RAS oncogene family	RAP2B	3.58E-02	1.085

ILMN_1226819	NM_009318	NaN (S)	Tapbp	TAP binding protein	Tapbp	5.62E-03	1.087
ILMN_2544692	AK010100	NaN (S)	2310067P03Rik	RIKEN cDNA 2310067P03 gene	2310067P03Rik	4.00E-02	1.087
ILMN_1251315	NM_021449	cereblon (Crbn), transcript variant 1, mRNA. (S)	Crbn	cereblon	CRBN	2.60E-02	1.088
ILMN_1229292	AK038032	NaN (S)	A130072N13Rik	n/a	n/a	3.91E-02	1.088
ILMN_2609600	XM_357628	NaN (S)	LOC384410	n/a	n/a	2.07E-02	1.088
ILMN_1219001	NM_146442	olfactory receptor 934 (Olfr934), mRNA. (S)	Olfr934	olfactory receptor 934	Olfr934	2.25E-02	1.088
ILMN_2909634	NM_013718	trafficking protein particle complex 3 (Trappc3), mRNA. (S)	Trappc3	trafficking protein particle complex 3	trappc3	1.38E-02	1.089
ILMN_1226099	NM_033622	NaN (S)	Tnfsf13b	tumor necrosis factor (ligand) superfamily, member 13b	TNFSF13B	2.39E-02	1.089
ILMN_1237935	AK087270	NaN (S)	E030040P03Rik	palladin, cytoskeletal associated protein	palld	4.49E-02	1.089
ILMN_1259884	XM_910859	PREDICTED: microtubule-actin crosslinking factor 1, transcript variant 21 (Macf1), mRNA. (S)	Macf1	microtubule-actin crosslinking factor 1	MACF1	4.02E-02	1.090
ILMN_2529272	XM_358487	NaN (S)	LOC381017	n/a	n/a	4.86E-02	1.090
ILMN_2822934	NM_001013384	podocan-like 1 (Podnl1), mRNA. (S)	Podnl1	podocan-like 1	PODNL1	4.79E-02	1.091
ILMN_1233905	NM_178050	atlastin GTPase 2 (Atl2), transcript variant 2, mRNA. (S)	Atl2	atlastin GTPase 2	atl2	2.52E-02	1.091
ILMN_2721399	NM_009985	cathepsin W (Ctsw), mRNA. (S)	Ctsw	cathepsin W	CTSW	8.72E-03	1.092
ILMN_2770270	NM_183286	dehydrogenase/reductase (SDR family) member 13 (Dhrs13), mRNA. (S)	Dhrs13	dehydrogenase/reductase (SDR family) member 13	DHRS13	4.48E-02	1.092
ILMN_1250092	AK039553	NaN (S)	A330060E23Rik	ankyrin repeat domain 55	ANKRD55	1.08E-02	1.092
ILMN_2754990	NM_001039150	CD44 antigen (Cd44), transcript variant 2, mRNA. (S)	Cd44	CD44 antigen	CD44	1.78E-02	1.093
ILMN_2495112	NM_028181	NaN (S)	D9Ertd392e	cell cycle progression 1	CCPG1	7.84E-03	1.093

ILMN_3161480	NM_139309	fukutin (Fktn), mRNA. (S)	Fktn	fukutin	FKTN	2.41E-02	1.094
ILMN_2773485	NM_178413	threonine synthase-like 2 (bacterial) (Thnsl2), transcript variant 1, mRNA. (S)	Thnsl2	threonine synthase-like 2 (bacterial)	Thnsl2	4.70E-02	1.094
ILMN_2662264	NM_029815	breast carcinoma amplified sequence 1 (Bcas1), mRNA. (S)	Bcas1	breast carcinoma amplified sequence 1	BCAS1	3.91E-02	1.095
ILMN_1252689	NM_023220	RIKEN cDNA 2010106G01 gene (2010106G01Rik), mRNA. (S)	2010106G01Rik	RIKEN cDNA 2010106G01 gene	2010106G01Rik	1.21E-02	1.095
ILMN_1250149	NM_001081695	DNA (cytosine-5-)-methyltransferase 3-like (Dnmt3l), transcript variant 2, mRNA. (S)	Dnmt3l	similar to DNA cytosine-5 methyltransferase 3-like protein; DNA (cytosine-5-)-methyltransferase 3-like	Dnmt3l	4.27E-02	1.096
ILMN_1220799	NM_011529	TRAF family member-associated Nf-kappa B activator (Tank), mRNA. (S)	Tank	TRAF family member-associated Nf-kappa B activator	TANK	7.80E-03	1.096
ILMN_2653143	NM_175127	F-box protein 28 (Fbxo28), mRNA. (S)	Fbxo28	F-box protein 28	Fbxo28	2.14E-02	1.096
ILMN_2931033	NM_030152	nucleolar protein 3 (apoptosis repressor with CARD domain) (Nol3), mRNA. (S)	Nol3	nucleolar protein 3 (apoptosis repressor with CARD domain)	NOL3	1.65E-02	1.097
ILMN_2760387	NM_001081678	zinc finger protein 800 (Zfp800), mRNA. (S)	Zfp800	zinc finger protein 800	Zfp800	2.43E-02	1.099
ILMN_1249549	XM_127137	NaN (S)	LOC380781	n/a	n/a	4.69E-02	1.100
ILMN_3055445	NM_175454	RIKEN cDNA C630004H02 gene (C630004H02Rik), mRNA. (I)	C630004H02Rik	hypothetical protein LOC100043986; RIKEN cDNA C630004H02 gene	C630004H02Rik	3.77E-02	1.100
ILMN_2593568	NM_172806	BTB (POZ) domain containing 7 (Btbd7), mRNA. (S)	Btbd7	BTB (POZ) domain containing 7	BTBD7	3.27E-02	1.100
ILMN_2774822	NM_201646	NaN (S)	Btbd6	BTB (POZ) domain containing 6	BTBD6	4.33E-02	1.100
ILMN_2912729	NM_022409	zinc finger protein 296 (Zfp296), mRNA. (S)	Zfp296	zinc finger protein 296	Zfp296	3.07E-02	1.100
ILMN_1259494	XM_988059	PREDICTED: sirtuin 4 (silent mating type information regulation 2 homolog) 4 (S. cerevisiae) (Sirt4), mRNA. (S)	Sirt4	sirtuin 4 (silent mating type information regulation 2 homolog) 4 (S. cerevisiae)	SIRT4	4.87E-02	1.100

ILMN_2624193	NM_178780	resistance to inhibitors of cholinesterase 3 homolog (C. elegans) (Ric3), transcript variant 2, mRNA. (S)	Ric3	resistance to inhibitors of cholinesterase 3 homolog (C. elegans)	RIC3	3.95E-02	1.101
ILMN_1225201	NM_019647	ribosomal protein L21 (Rpl21), mRNA. (S)	Rpl21	predicted gene 12618; predicted gene 8724; predicted gene 10155; predicted gene 3355; predicted gene 3713; predicted gene 3201; predicted gene 13641; similar to ribosomal protein L21; predicted gene 12411; predicted gene 5445; predicted gene 5495; predicted gene 13604; predicted gene 10045; predicted gene 14648; predicted gene 15150; predicted gene 8252; predicted gene 8157; predicted gene 8880; predicted gene 6813; predicted gene 15312; predicted gene 5534; predicted gene 8054; predicted gene 10095; predicted gene 5857; predicted gene 8195; predicted gene 8840; predicted gene 10240; predicted gene 5810; predicted gene 11975; predicted gene 5816; predicted gene 7547; predicted gene 7702; predicted gene 8012; predicted gene 8557; predicted gene 12760; predicted gene 2815; predicted gene 14336; predicted gene 7806; predicted gene 7799; predicted gene 5042; predicted gene 7062; similar to 60S ribosomal protein L21; predicted gene 8397; predicted gene 5528; ribosomal protein L21; predicted gene 11703; predicted gene 12944; predicted gene 5502; predicted gene 10163; predicted gene 7218; predicted gene 15309; ribosomal protein L21 pseudogene; predicted gene 8915; predicted gene 16060; predicted gene 13653; predicted gene 6689; predicted gene 8101; predicted gene 9130	LOC100046290	1.69E-02	1.101
ILMN_2428252	NM_053268	RAS p21 protein activator 2 (Rasa2), mRNA. (S)	Rasa2	RAS p21 protein activator 2	RASA2	2.35E-02	1.102
ILMN_2627557	NM_144517	TBC1 domain family, member 19 (Tbc1d19), mRNA. (S)	Tbc1d19	TBC1 domain family, member 19	tbc1d19	4.59E-02	1.103
ILMN_2669215	NM_199465	nexilin (Nexn), mRNA. (S)	Nexn	nexilin	NEXN	3.48E-02	1.103
ILMN_2683794	NM_010952	ornithine decarboxylase antizyme 2 (Oaz2), mRNA. (S)	Oaz2	predicted gene 9329; predicted gene 7543; ornithine decarboxylase antizyme 2	Oaz2	2.83E-02	1.104

ILMN_2734168	NM_177722	NaN (S)	6030422M02Rik	RIKEN cDNA 6030422M02 gene	6030422M02Rik	2.79E-02	1.104
ILMN_1257165	XM_358723	NaN (S)	LOC382262	n/a	n/a	3.71E-02	1.104
ILMN_1227461	AK086619	NaN (S)	Birc6	baculoviral IAP repeat-containing 6	birc6	6.26E-03	1.104
ILMN_2985497	NM_010872	NLR family, apoptosis inhibitory protein 2 (Naip2), mRNA. (S)	Naip2	NLR family, apoptosis inhibitory protein 1, related sequence 1; NLR family, apoptosis inhibitory protein 2	Naip2	1.21E-02	1.105
ILMN_1257241	NM_011616	CD40 ligand (Cd40lg), mRNA. (S)	Cd40lg	CD40 ligand	CD40LG	2.74E-02	1.105
ILMN_2509327	XM_620310	PREDICTED: WAS/WASL interacting protein family, member 3 (Wipf3), mRNA. (S)	Wipf3	WAS/WASL interacting protein family, member 3	WIPF3	9.46E-03	1.106
ILMN_1249297	NM_001039533	pyridoxal-dependent decarboxylase domain containing 1 (Pdxdc1), transcript variant 3, mRNA. (S)	Pdxdc1	pyridoxal-dependent decarboxylase domain containing 1	PDXDC1	4.61E-02	1.108
ILMN_2946288	NM_008432	potassium channel, subfamily U, member 1 (Kcnu1), mRNA. (S)	Kcnu1	potassium channel, subfamily U, member 1	KCNU1	2.80E-02	1.108
ILMN_1248737	XM_001474642	PREDICTED: RIKEN cDNA 4930481A15 gene (4930481A15Rik), mRNA. (S)	4930481A15Rik	RIKEN cDNA 4930481A15 gene	4930481A15Rik	4.67E-02	1.109
ILMN_1222869	XM_907370	PREDICTED: collagen, type XXII, alpha 1, transcript variant 3 (Col22a1), mRNA. (S)	Col22a1	collagen, type XXII, alpha 1	Col22a1	7.11E-03	1.109
ILMN_2421751	NM_153103	kinesin family member 1C (Kif1c), mRNA. (S)	Kif1c	kinesin family member 1C	Kif1c	1.25E-02	1.109
ILMN_2743139	XM_985084	PREDICTED: succinate-Coenzyme A ligase, GDP-forming, beta subunit (Suclg2), mRNA. (S)	Suclg2	succinate-Coenzyme A ligase, GDP-forming, beta subunit	suclg2	2.88E-02	1.109
ILMN_2521147	NM_026424	NaN (S)	1500041J02Rik	hypothetical protein LOC675736; coenzyme Q10 homolog B (S. cerevisiae); predicted gene 4899	Gm4899	1.13E-02	1.110
ILMN_2591908	NM_001039534	phosphoseryl-tRNA kinase (Pstk), mRNA. (S)	Pstk	phosphoseryl-tRNA kinase	PSTK	3.74E-02	1.110
ILMN_1218661	AK050030	NaN (S)	C730004N19Rik	mirror-image polydactyly gene 1 homolog (human); phosphoribosyl pyrophosphate synthetase 1; phosphoribosyl pyrophosphate synthetase 1-like 1	Gm5081	2.98E-02	1.110

ILMN_1216917	NM_183145	zinc finger protein 748 (Zfp748), transcript variant 2, mRNA. (S)	Zfp748	zinc finger protein 748	Zfp748	3.28E-02	1.111
ILMN_2772470	NM_009619	a disintegrin and metallopeptidase domain 3 (cyritestin) (Adam3), mRNA. (S)	Adam3	a disintegrin and metallopeptidase domain 3 (cyritestin)	Adam3	3.14E-02	1.111
ILMN_1238609	NM_026274	ring finger and SPRY domain containing 1 (Rspry1), mRNA. (S)	Rspry1	ring finger and SPRY domain containing 1	rspry1	4.34E-03	1.111
ILMN_2547737	NM_172464	dishevelled associated activator of morphogenesis 1 (Daam1), transcript variant 2, mRNA. (S)	Daam1	dishevelled associated activator of morphogenesis 1	DAAM1	2.94E-03	1.112
ILMN_2752479	XM_354672	NaN (S)	Mipol1	n/a	n/a	1.74E-03	1.112
ILMN_1228762	NM_010091	NaN (S)	Dvl1	dishevelled, dsh homolog 1 (Drosophila)	DVL1	1.54E-04	1.112
ILMN_1234199	NM_177025	Cobl-like 1 (Cobll1), mRNA. (S)	Cobll1	Cobl-like 1	Cobll1	4.30E-02	1.112
ILMN_1224981	XM_900189	PREDICTED: glutaminase, transcript variant 5 (Gls), mRNA. (S)	Gls	glutaminase	GLS	3.74E-02	1.112
ILMN_1224839	XM_001002241	PREDICTED: vacuolar protein sorting 13D (yeast), transcript variant 2 (Vps13d), mRNA. (S)	Vps13d	vacuolar protein sorting 13 D (yeast)	VPS13D	4.21E-02	1.113
ILMN_2602821	NM_025876	CDK5 regulatory subunit associated protein 1 (Cdk5rap1), mRNA. (S)	Cdk5rap1	CDK5 regulatory subunit associated protein 1	CDK5RAP1	3.65E-02	1.113
ILMN_2455575	XM_001003389	PREDICTED: similar to downregulated in ovarian cancer 1 isoform 2 (LOC676704), mRNA. (S)	LOC676704	n/a	n/a	2.91E-02	1.114
ILMN_2572227	XM_978140	PREDICTED: DNA segment, Chr 14, Abbott 1 expressed, transcript variant 4 (D14Abb1e), mRNA. (S)	D14Abb1e	DNA segment, Chr 14, Abbott 1 expressed	D14Abb1e	2.50E-02	1.114
ILMN_1238676	NM_207215	MYC binding protein 2 (Mycbp2), mRNA. (S)	Mycbp2	MYC binding protein 2	MYCBP2	2.30E-03	1.114
ILMN_1252283	NM_054040	tubby like protein 4 (Tulp4), mRNA. (S)	Tulp4	similar to mKIAA1397 protein; tubby like protein 4	TULP4	1.51E-02	1.115
ILMN_2486267	NM_019949	ubiquitin-conjugating enzyme E2L 6 (Ube2l6), mRNA. (S)	Ube2l6	ubiquitin-conjugating enzyme E2L 6	Ube2l6	3.41E-02	1.115

ILMN_1216663	NM_029836	NaN (S)	DXBwg1396e	TSPY-like 2	TSPYL2	2.99E-02	1.115
ILMN_2630029	NM_028149	F-box and leucine-rich repeat protein 20 (Fbxl20), mRNA. XM_903031 XM_903032 XM_903033 XM_915017 XM_923494 XM_923501 XM_923516 XM_923522 XM_923532 XM_923536 XM_982900 XM_982933 XM_982970 XM_983008 XM_983045 XM_983087 XM_983129 XM_983157 XM_992930 (S)	Fbxl20	F-box and leucine-rich repeat protein 20	Fbxl20	8.27E-03	1.115
ILMN_2466809	NM_011864	3'-phosphoadenosine 5'-phosphosulfate synthase 2 (Papss2), mRNA. (S)	Papss2	3'-phosphoadenosine 5'-phosphosulfate synthase 2	PAPSS2	3.81E-02	1.116
ILMN_1221256	NM_152839	immunoglobulin joining chain (Igj), mRNA. (S)	Igj	immunoglobulin joining chain	Igj	4.45E-02	1.116
ILMN_2611767	NM_022420	G protein-coupled receptor, family C, group 5, member B (Gprc5b), mRNA. (S)	Gprc5b	G protein-coupled receptor, family C, group 5, member B	Gprc5b	3.43E-02	1.116
ILMN_1233456	NM_001081378	RIKEN cDNA C330002I19 gene (C330002I19Rik), mRNA. (S)	C330002I19Rik	kinase D-interacting substrate 220	KIDINS220	2.60E-02	1.117
ILMN_1243274	AK002489	NaN (S)	0610010K06Rik	predicted gene 5578; RIKEN cDNA 0610010K06 gene	0610010K06Rik	4.70E-02	1.117
ILMN_1226246	XM_001473279	PREDICTED: hypothetical protein LOC100039660 (LOC100039660), mRNA. (S)	LOC100039660	hypothetical protein LOC100045792; predicted gene 10331	LOC100045792	4.15E-02	1.118
ILMN_2539220	XM_357007	NaN (S)	LOC383363	n/a	n/a	1.06E-02	1.118
ILMN_2775130	NM_011386	SKI-like (Skil), transcript variant 1, mRNA. (S)	Skil	SKI-like	Skil	4.90E-02	1.119
ILMN_1244291	NM_010288	gap junction membrane channel protein alpha 1 (Gja1), mRNA. (S)	Gja1	gap junction protein, alpha 1	GJA1	4.03E-02	1.119
ILMN_1216433	XR_031715	PREDICTED: similar to Ssxb3 protein (LOC100045460), misc RNA. (S)	LOC100045460	similar to Ssxb3 protein	LOC100045460	3.49E-02	1.119

ILMN_1216560	NM_027758	TBC1 domain family, member 9 (Tbc1d9), mRNA. (S)	Tbc1d9	TBC1 domain family, member 9	TBC1D9	8.72E-03	1.119
ILMN_2821916	NM_025449	nicolin 1 (Nicn1), mRNA. (S)	Nicn1	nicolin 1	Nicn1	2.35E-02	1.120
ILMN_3000080	NM_175133	RIKEN cDNA 1110038D17 gene (1110038D17Rik), mRNA. (S)	1110038D17Rik	RIKEN cDNA 1110038D17 gene	1110038D17Rik	3.41E-02	1.120
ILMN_1254199	NM_008064	glucosidase, alpha, acid (Gaa), mRNA. (S)	Gaa	glucosidase, alpha, acid	GAA	1.23E-02	1.121
ILMN_1248388	XM_001473988	PREDICTED: similar to mKIAA1021 protein (LOC100045280), mRNA. (S)	LOC100045280	similar to mKIAA1021 protein	LOC100045280	3.19E-02	1.121
ILMN_3068535	NM_001025106	transmembrane protein 201 (Tmem201), transcript variant 1, mRNA. (I)	Tmem201	transmembrane protein 201	TMEM201	4.89E-02	1.122
ILMN_2576984	AK049052	NaN (S)	C230095J06Rik	propionyl-Coenzyme A carboxylase, alpha polypeptide	pccA	2.12E-02	1.123
ILMN_2631591	NM_001081756	RIKEN cDNA E030049G20 gene (E030049G20Rik), transcript variant 1, mRNA. (S)	E030049G20Rik	RIKEN cDNA E030049G20 gene	NCKAP5	1.54E-02	1.123
ILMN_2718861	NM_001083882	RIKEN cDNA 1600012H06 gene (1600012H06Rik), transcript variant 4, mRNA. (S)	1600012H06Rik	RIKEN cDNA 1600012H06 gene	1600012H06Rik	4.75E-02	1.123
ILMN_1243591	NM_025739	ring finger protein 220 (Rnf220), mRNA. (S)	Rnf220	ring finger protein 220	Rnf220	4.94E-02	1.123
ILMN_3107059	NM_207690	espin (Espn), transcript variant 5, mRNA. (A)	Espn	espin	Espn	2.93E-02	1.124
ILMN_2674132	NM_021438	NaN (S)	Fibp	fibroblast growth factor (acidic) intracellular binding protein	FIBP	4.70E-04	1.125
ILMN_1221943	NM_022324	stromal cell-derived factor 2-like 1 (Sdf2l1), mRNA. (S)	Sdf2l1	stromal cell-derived factor 2-like 1	SDF2L1	2.13E-02	1.125
ILMN_2607547	NM_018794	ATPase, H+ transporting, lysosomal accessory protein 1 (Atp6ap1), mRNA. (S)	Atp6ap1	ATPase, H+ transporting, lysosomal accessory protein 1	ATP6AP1	3.94E-02	1.125
ILMN_2584985	AK089900	NaN (S)	F830048D03Rik	UV radiation resistance associated gene	UVRAG	4.37E-02	1.125
ILMN_2827081	NM_013731	serum/glucocorticoid regulated kinase 2 (Sgk2), mRNA. (S)	Sgk2	serum/glucocorticoid regulated kinase 2	SGK2	3.60E-02	1.126

ILMN_2630641	NM_009272	spermidine synthase (Srm), mRNA. (S)	Srm	spermidine synthase	SRM	1.78E-02	1.126
ILMN_1239720	NM_023423	akirin 1 (Akirin1), mRNA. (S)	Akirin1	akirin 1	AKIRIN1	9.71E-03	1.127
ILMN_2949021	NM_178772	arylacetamide deacetylase-like 1 (Aadacl1), mRNA. (S)	Aadacl1	arylacetamide deacetylase-like 1	NCEH1	4.37E-02	1.127
ILMN_2615145	NM_175401	F-box and WD-40 domain protein 17 (Fbxw17), mRNA. (S)	Fbxw17	F-box and WD-40 domain protein 17	Fbxw17	1.06E-02	1.127
ILMN_2572010	AK051157	NaN (S)	Sas	N-acetylneuraminic acid synthase (sialic acid synthase)	NANS	2.34E-02	1.127
ILMN_1247483	XM_355170	NaN (S)	LOC381234	n/a	n/a	4.48E-02	1.128
ILMN_2708546	NM_029585	de-etiolated homolog 1 (Arabidopsis) (Det1), mRNA. (S)	Det1	de-etiolated homolog 1 (Arabidopsis)	DET1	2.74E-02	1.128
ILMN_2616015	NM_173186	TBC1 domain family, member 24 (Tbc1d24), mRNA. (S)	Tbc1d24	TBC1 domain family, member 24	Tbc1d24	2.77E-02	1.128
ILMN_1215164	AK029140	NaN (S)	Prc1	protein regulator of cytokinesis 1	prc1	2.22E-02	1.128
ILMN_1213149	NaN	NaN (S)	1110029L17Rik	n/a	n/a	4.13E-02	1.128
ILMN_2761169	NM_173181	NaN (S)	3110050N22Rik	family with sequence similarity 164, member A	FAM164A	1.94E-03	1.129
ILMN_2681474	NM_001081109	lemur tyrosine kinase 2 (Lmtk2), mRNA. (S)	Lmtk2	lemur tyrosine kinase 2	LMTK2	1.27E-02	1.129
ILMN_1239029	AK039587	NaN (S)	A330067K20Rik	n/a	n/a	1.26E-02	1.129
ILMN_2617005	NM_011169	prolactin receptor (Prlr), mRNA. (S)	Prlr	prolactin receptor	PRLR	4.27E-02	1.129
ILMN_2651389	NM_010800	basic helix-loop-helix domain containing, class B, 8 (Bhlhb8), mRNA. (S)	Bhlhb8	basic helix-loop-helix family, member a15	bhlha15	8.57E-03	1.129
ILMN_2522750	NaN	NaN (S)	Trio	n/a	n/a	2.74E-02	1.129
ILMN_2665708	NM_178879	UDP-GlcNAc:betaGal beta-1,3-N-acetylglucosaminyltransferase 9 (B3gnt9), mRNA. (S)	B3gnt9	UDP-GlcNAc:betaGal beta-1,3-N-acetylglucosaminyltransferase 9	B3gnt9	7.04E-03	1.130
ILMN_2624497	NM_133900	phosphoserine phosphatase (Psph), mRNA. (S)	Psph	phosphoserine phosphatase	PSPH	7.49E-03	1.130

ILMN_1252447	NM_177128	IQ calmodulin-binding motif containing 1 (Iqcb1), mRNA. (S)	Iqcb1	IQ calmodulin-binding motif containing 1	IQCB1	3.54E-02	1.130
ILMN_1243217	NM_028651	transmembrane and tetratricopeptide repeat containing 4 (Tmtc4), mRNA. (S)	Tmtc4	transmembrane and tetratricopeptide repeat containing 4	Tmtc4	2.79E-02	1.131
ILMN_1213290	NM_001077696	histone deacetylase 5 (Hdac5), transcript variant 1, mRNA. (S)	Hdac5	histone deacetylase 5	HDAC5	3.82E-02	1.132
ILMN_2886896	NM_028725	RIKEN cDNA 4632417N05 gene (4632417N05Rik), mRNA. (S)	4632417N05Rik	short chain dehydrogenase/reductase family 42E, member 1	sdr42e1	1.61E-02	1.132
ILMN_2974720	NM_183029	insulin-like growth factor 2 mRNA binding protein 2 (Igf2bp2), mRNA. (S)	Igf2bp2	insulin-like growth factor 2 mRNA binding protein 2	Igf2bp2	3.39E-02	1.132
ILMN_2639207	XR_034030	PREDICTED: similar to calcium activated potassium channel beta 4 subunit (LOC100047870), misc RNA. (S)	LOC100047870	similar to calcium activated potassium channel beta 4 subunit	LOC100047870	4.64E-02	1.133
ILMN_1215277	XM_135116	PREDICTED: phosphatidylinositol 3 kinase, regulatory subunit, polypeptide 4, p150, transcript variant 1 (Pik3r4), mRNA. (S)	Pik3r4	phosphatidylinositol 3 kinase, regulatory subunit, polypeptide 4, p150	PIK3R4	2.53E-02	1.133
ILMN_2487554	NaN	NaN (S)	A330042I21Rik	n/a	n/a	4.50E-02	1.133
ILMN_2839886	NM_010753	Max dimerization protein 4 (Mxd4), mRNA. (S)	Mxd4	Max dimerization protein 4	MXD4	1.93E-02	1.133
ILMN_2466601	XM_915908	PREDICTED: WD repeat domain 7, transcript variant 5 (Wdr7), mRNA. (S)	Wdr7	WD repeat domain 7	WDR7	3.62E-03	1.134
ILMN_2454649	NaN	NaN (S)	mtDNA_COXIII	n/a	n/a	4.44E-02	1.134
ILMN_2775512	NM_007915	NaN (S)	Ei24	etoposide induced 2.4 mRNA	EI24	3.39E-02	1.134
ILMN_1236383	NM_009582	mitogen-activated protein kinase kinase kinase 12 (Map3k12), mRNA. (S)	Map3k12	mitogen-activated protein kinase kinase kinase 12	MAP3K12	3.74E-02	1.134
ILMN_1243031	AK038676	NaN (S)	Trrp1	transient receptor potential cation channel, subfamily C, member 1	Trpc1	2.88E-02	1.135
ILMN_2736968	NM_029023	serine carboxypeptidase 1 (Scpep1), mRNA. (S)	Scpep1	serine carboxypeptidase 1	Scpep1	4.43E-02	1.136

ILMN_1236070	NM_007780	colony stimulating factor 2 receptor, beta, low-affinity (granulocyte-macrophage) (Csf2rb), mRNA. (S)	Csf2rb	colony stimulating factor 2 receptor, beta, low-affinity (granulocyte-macrophage)	CSF2RB	1.43E-02	1.136
ILMN_1228441	AK048752	NaN (S)	C230043G09Rik	pogo transposable element with KRAB domain	Pogk	2.00E-02	1.136
ILMN_2712427	NM_019927	ariadne ubiquitin-conjugating enzyme E2 binding protein homolog 1 (Drosophila) (Arih1), mRNA. (S)	Arih1	ariadne ubiquitin-conjugating enzyme E2 binding protein homolog 1 (Drosophila)	ARIH1	2.37E-02	1.136
ILMN_2662192	NM_026240	GRAM domain containing 3 (Gramd3), mRNA. (S)	Gramd3	GRAM domain containing 3	GRAMD3	2.09E-02	1.137
ILMN_2956381	NM_026780	SYF2 homolog, RNA splicing factor (S. cerevisiae) (Syf2), mRNA. (S)	Syf2	SYF2 homolog, RNA splicing factor (S. cerevisiae)	Syf2	1.04E-02	1.137
ILMN_1243381	NM_175288	NaN (S)	C230066G23Rik	n/a	n/a	2.44E-02	1.137
ILMN_1224339	NM_198295	thioredoxin domain containing 10 (Txndc10), mRNA. (S)	Txndc10	thioredoxin-related transmembrane protein 3	TMX3	3.51E-02	1.138
ILMN_1212991	AK014071	NaN (S)	3110023E09Rik	chitinase domain containing 1	CHID1	1.71E-03	1.138
ILMN_2674032	NM_025480	transmembrane protein 128 (Tmem128), mRNA. (S)	Tmem128	transmembrane protein 128	Tmem128	3.66E-02	1.139
ILMN_3035795	NM_027326	myeloid/lymphoid or mixed-lineage leukemia (trithorax homolog, Drosophila); translocated to, 3 (Mllt3), transcript variant 1, mRNA. (I)	Mllt3	myeloid/lymphoid or mixed-lineage leukemia (trithorax homolog, Drosophila); translocated to, 3	MLLT3	2.65E-03	1.139
ILMN_1251494	AK042081	NaN (S)	A630056H20Rik	phosphodiesterase 4D interacting protein (myomegalin)	PDE4DIP	3.23E-03	1.141
ILMN_2710665	NM_026214	potassium channel tetramerisation domain containing 4 (Kctd4), mRNA. (S)	Kctd4	potassium channel tetramerisation domain containing 4	Kctd4	4.58E-02	1.141
ILMN_1250956	NaN	NaN (S)	6330414G02Rik	n/a	n/a	3.74E-02	1.141
ILMN_2659168	NM_019686	calcium and integrin binding family member 2 (Cib2), mRNA. (S)	Cib2	calcium and integrin binding family member 2	cib2	8.96E-03	1.141

ILMN_1225129	XM_150337	NaN (S)	Pip5k1c	n/a	n/a	2.92E-02	1.141
ILMN_2611422	XM_489058	NaN (S)	2700033B16Rik	n/a	n/a	4.50E-02	1.141
ILMN_1244653	AK054453	NaN (S)	E330027G05Rik	enhancer trap locus 4	Etl4	1.13E-02	1.142
ILMN_3162476	NM_016906	Sec61 alpha 1 subunit (S. cerevisiae) (Sec61a1), mRNA. (S)	Sec61a1	Sec61 alpha 1 subunit (S. cerevisiae)	SEC61A1	1.80E-02	1.143
ILMN_2760977	NM_008378	imprinted and ancient (Impact), mRNA. (S)	Impact	imprinted and ancient	impact	2.41E-02	1.143
ILMN_1256085	AK040938	NaN (S)	Il10rb	interleukin 10 receptor, beta	Il10rb	2.49E-02	1.143
ILMN_2707043	NM_007898	phenylalkylamine Ca2+ antagonist (emopamil) binding protein (Ebp), mRNA. (S)	Ebp	phenylalkylamine Ca2+ antagonist (emopamil) binding protein	EBP	1.31E-02	1.144
ILMN_1245825	XM_134800	NaN (S)	1810046K07Rik	RIKEN cDNA 1810046K07 gene	1810046K07Rik	2.46E-03	1.146
ILMN_1234503	AK029353	NaN (S)	4833403D03Rik	signal sequence receptor, gamma	SSR3	8.55E-03	1.146
ILMN_2636005	NM_175279	Ras association (RalGDS/AF-6) domain family (N-terminal) member 10 (Rassf10), mRNA. (S)	Rassf10	Ras association (RalGDS/AF-6) domain family (N-terminal) member 10	rassf10	1.65E-02	1.146
ILMN_2691243	NM_026604	family with sequence similarity 135, member A (Fam135a), mRNA. (S)	Fam135a	family with sequence similarity 135, member A	FAM135A	1.46E-02	1.147
ILMN_2444023	NM_016800	vesicle transport through interaction with t-SNAREs 1B homolog (Vti1b), mRNA. (S)	Vti1b	vesicle transport through interaction with t-SNAREs 1B homolog	VTI1B	1.96E-03	1.147
ILMN_1250826	XM_488744	NaN (S)	Pvt1	n/a	n/a	2.74E-02	1.147
ILMN_2740520	NM_146207	cullin 4A (Cul4a), mRNA. (S)	Cul4a	cullin 4A	cul4a	1.22E-02	1.148
ILMN_2757838	NM_199056	inositol 1,3,4,5,6-pentakisphosphate 2-kinase (Ippk), mRNA. (S)	Ippk	inositol 1,3,4,5,6-pentakisphosphate 2-kinase	IPPK	8.60E-03	1.148
ILMN_2710112	NM_010261	NaN (S)	Rabac1	Rab acceptor 1 (prenylated)	rabac1	9.66E-04	1.148

ILMN_2614324	NM_007561	bone morphogenic protein receptor, type II (serine/threonine kinase) (Bmpr2), mRNA. (S)	Bmpr2	bone morphogenic protein receptor, type II (serine/threonine kinase)	bmpr2	1.58E-02	1.149
ILMN_2681583	NM_028995	NIPA-like domain containing 3 (Npal3), mRNA. (S)	Npal3	NIPA-like domain containing 3; similar to NIPA-like domain containing 3	NIPAL3	4.50E-02	1.150
ILMN_1221011	NM_173181	family with sequence similarity 164, member A (Fam164a), mRNA. (S)	Fam164a	family with sequence similarity 164, member A	FAM164A	2.97E-02	1.151
ILMN_2800358	NM_177821	E1A binding protein p300 (Ep300), mRNA. (S)	Ep300	E1A binding protein p300	EP300	3.31E-02	1.151
ILMN_3014674	NM_001013811	family with sequence similarity 169, member B (Fam169b), mRNA. (I)	Fam169b	family with sequence similarity 169, member B	FAM169B	4.50E-03	1.151
ILMN_1255046	NM_172588	serine incorporator 5 (Serinc5), mRNA. (S)	Serinc5	serine incorporator 5	SERINC5	2.53E-02	1.152
ILMN_1244746	AK046198	NaN (S)	B230350I06Rik	aldehyde dehydrogenase 1 family, member L2	Aldh1l2	1.24E-02	1.152
ILMN_1238654	NM_027352	golgi reassembly stacking protein 2 (Gorasp2), mRNA. (S)	Gorasp2	golgi reassembly stacking protein 2	gorasp2	3.94E-02	1.152
ILMN_1220973	NM_146016	RIKEN cDNA C230094A16 gene (C230094A16Rik), mRNA. (S)	C230094A16Rik	RIKEN cDNA C230094A16 gene	Eml6	1.25E-02	1.152
ILMN_3072676	NM_001025572	ankyrin repeat domain 12 (Ankrd12), mRNA. (I)	Ankrd12	ankyrin repeat domain 12; similar to Ankrd12 protein	ANKRD12	1.25E-02	1.152
ILMN_2982781	NM_021542	potassium channel, subfamily K, member 5 (Kcnk5), mRNA. (S)	Kcnk5	potassium channel, subfamily K, member 5	KCNK5	6.76E-03	1.153
ILMN_2662329	NM_146197	acyl-CoA synthetase medium-chain family member 2 (Acsm2), nuclear gene encoding mitochondrial protein, mRNA. (S)	Acsm2	acyl-CoA synthetase medium-chain family member 2	Acsm2	3.55E-02	1.153
ILMN_2503893	NM_016703	NaN (S)	Preb	prolactin regulatory element binding	PREB	4.66E-02	1.153
ILMN_1222000	NM_026942	stomatin-like 1 (Stoml1), mRNA. (S)	Stoml1	stomatin-like 1	stoml1	4.94E-02	1.154
ILMN_2769971	NM_153408	lung-inducible neuralized-related C3HC4 RING domain protein (Lincr), mRNA. (S)	Lincr	neuralized homolog 3 homolog (Drosophila)	neurl3	4.53E-02	1.154

ILMN_3150658	NM_008705	non-metastatic cells 2, protein (NM23B) expressed in (Nme2), transcript variant 1, mRNA. (A)	Nme2	predicted gene 7730; non-metastatic cells 2, protein (NM23B) expressed in; predicted gene 5566; predicted gene 5425; similar to Nucleoside diphosphate kinase B (NDK B) (NDP kinase B) (P18)	NME2	3.67E-02	1.155
ILMN_2914347	NM_023220	RIKEN cDNA 2010106G01 gene (2010106G01Rik), mRNA. (S)	2010106G01Rik	RIKEN cDNA 2010106G01 gene	2010106G01Rik	4.12E-02	1.156
ILMN_2558455	AK031307	NaN (S)	6030405B10Rik	sema domain, transmembrane domain (TM), and cytoplasmic domain, (semaphorin) 6D	SEMA6D	4.36E-03	1.156
ILMN_3056417	NM_001033243	cDNA sequence BC013491 (BC013491), mRNA. XM_925312 (I)	BC013491	coiled-coil domain containing 114	Ccdc114	1.28E-02	1.156
ILMN_2772623	NM_145820	NaN (S)	2810471M23Rik	ventricular zone expressed PH domain homolog 1 (zebrafish)	VEPH1	4.19E-02	1.157
ILMN_2701339	NM_022423	NaN (S)	2410016F01Rik	ring finger protein 187; predicted gene 9686	Rnf187	2.70E-02	1.157
ILMN_2595477	NM_016887	claudin 7 (Cldn7), mRNA. (S)	Cldn7	claudin 7	cldn7	4.54E-02	1.157
ILMN_2425048	NaN	NaN (S)	IGKV2-112_J00562_Ig_kappa_variable_2-112_55	n/a	n/a	4.63E-03	1.158
ILMN_1236613	NM_030168	RIKEN cDNA 4921505C17 gene (4921505C17Rik), mRNA. (S)	4921505C17Rik	RPTOR independent companion of MTOR, complex 2	RICTOR	4.31E-02	1.158
ILMN_2632230	NM_145528	DNA segment, Chr 2, ERATO Doi 391, expressed (D2Ertd391e), mRNA. (S)	D2Ertd391e	DNA segment, Chr 2, ERATO Doi 391, expressed	D2Ertd391e	5.48E-03	1.159
ILMN_1258855	NaN	NaN (S)	9430069I07Rik	n/a	n/a	1.36E-02	1.159
ILMN_2702600	NM_001033450	myeloid cell nuclear differentiation antigen (Mnda), mRNA. (S)	Mnda	myeloid cell nuclear differentiation antigen	MNDA	1.27E-02	1.159
ILMN_2876794	NM_001005227	olfactory receptor 1200 (Olfr1200), mRNA. (S)	Olfr1200	olfactory receptor 1200	Olfr1200	1.49E-02	1.160
ILMN_1242158	XM_989935	PREDICTED: PHD finger protein 20-like 1, transcript variant 6 (Phf20l1), mRNA. (S)	Phf20l1	PHD finger protein 20-like 1	PHF20L1	2.45E-02	1.161

ILMN_1217316	AK017705	NaN (S)	5730478M09Rik	TBCC domain containing 1	TBCCD1	5.33E-03	1.162
ILMN_2459071	NM_020256	zinc finger and BTB domain containing 33 (Zbtb33), transcript variant 1, mRNA. (S)	Zbtb33	zinc finger and BTB domain containing 33	ZBTB33	3.48E-02	1.163
ILMN_1227642	AK050233	NaN (S)	C730029F17Rik	WD repeat domain 51B	poc1b	4.02E-02	1.163
ILMN_2936517	NM_007537	BCL2-like 2 (Bcl2l2), mRNA. (S)	Bcl2l2	BCL2-like 2	Bcl2l2	3.84E-04	1.164
ILMN_2778789	NM_144906	SH3-domain GRB2-like (endophilin) interacting protein 1 (Sgip1), mRNA. (S)	Sgip1	SH3-domain GRB2-like (endophilin) interacting protein 1	Sgip1	3.48E-02	1.164
ILMN_2661744	NM_001080941	zinc finger protein 429 (Zfp429), mRNA. XM_985158 XM_985191 XM_985227 XM_985266 XM_990399 (S)	Zfp429	zinc finger protein 429	Zfp429	9.31E-03	1.164
ILMN_1223010	AK086955	NaN (S)	Syn3	synapsin III	SYN3	4.27E-02	1.164
ILMN_2744752	NM_024182	RIO kinase 3 (yeast) (Riok3), mRNA. (S)	Riok3	RIO kinase 3 (yeast)	Riok3	3.29E-02	1.164
ILMN_2685985	XM_194337	PREDICTED: multiple EGF-like-domains 8 (Megf8), mRNA. (S)	Megf8	multiple EGF-like-domains 8; similar to coiled-coil domain containing 72; coiled-coil domain containing 72; predicted gene 7526; predicted gene 9613; predicted gene 15235; predicted gene 14200	CCDC72	3.90E-02	1.165
ILMN_2687503	NM_001081046	EF-hand calcium binding domain 3 (Efcab3), mRNA. (S)	Efcab3	EF-hand calcium binding domain 3	EFCAB3	2.15E-02	1.165
ILMN_2970241	NM_133818	expressed sequence AI597479 (AI597479), mRNA. (S)	AI597479	expressed sequence AI597479	AI597479	4.34E-02	1.165
ILMN_1252698	NM_025295	biotinidase (Btd), mRNA. (S)	Btd	biotinidase	BTD	1.17E-02	1.165
ILMN_2860649	NM_145545	guanylate binding protein 6 (Gbp6), mRNA. (S)	Gbp6	guanylate binding protein 6	Gbp6	3.25E-02	1.165
ILMN_2707188	NM_172688	mitogen-activated protein kinase kinase kinase 7 (Map3k7), mRNA. (S)	Map3k7	mitogen-activated protein kinase kinase kinase 7; predicted gene 8188	MAP3K7	4.51E-02	1.166
ILMN_2798255	NM_019543	phosphatidylinositol glycan anchor biosynthesis, class P (Pigp), mRNA. (S)	Pigp	phosphatidylinositol glycan anchor biosynthesis, class P; predicted gene 9001	Gm9001	4.70E-02	1.166

ILMN_2456243	NM_011749	zinc finger protein 148 (Zfp148), mRNA. (S)	Zfp148	zinc finger protein 148	Zfp148	3.27E-03	1.166
ILMN_2503318	NM_019490	USO1 homolog, vesicle docking protein (yeast) (Uso1), mRNA. (S)	Uso1	USO1 homolog, vesicle docking protein (yeast)	USO1	3.54E-03	1.166
ILMN_1224129	NM_001081066	DENN/MADD domain containing 3 (Dennd3), mRNA. (S)	Dennd3	DENN/MADD domain containing 3	DENND3	1.91E-03	1.167
ILMN_2503492	XM_489360	NaN (S)	5530601H04Rik	n/a	n/a	4.92E-03	1.167
ILMN_1235131	NM_172670	glycosyltransferase-like 1B (Gyltl1b), mRNA. (S)	Gyltl1b	glycosyltransferase-like 1B	Gyltl1b	2.46E-02	1.167
ILMN_2672190	NM_010495	inhibitor of DNA binding 1 (Id1), mRNA. (S)	Id1	inhibitor of DNA binding 1	id1	4.15E-02	1.167
ILMN_1236569	XM_356096	NaN (S)	LOC382026	n/a	n/a	4.70E-02	1.167
ILMN_2872698	NM_172406	trafficking protein, kinesin binding 2 (Trak2), mRNA. (S)	Trak2	trafficking protein, kinesin binding 2	TRAK2	3.82E-02	1.169
ILMN_2588682	NM_177583	anterior pharynx defective 1b homolog (C. elegans) (Aph1b), mRNA. (S)	Aph1b	anterior pharynx defective 1b homolog (C. elegans)	APH1B	7.49E-03	1.169
ILMN_2886646	NM_013563	interleukin 2 receptor, gamma chain (Il2rg), mRNA. (S)	Il2rg	predicted gene 614; interleukin 2 receptor, gamma chain	Gm614	1.28E-02	1.169
ILMN_1238331	NM_009073	rod outer segment membrane protein 1 (Rom1), mRNA. (S)	Rom1	rod outer segment membrane protein 1	Rom1	4.79E-02	1.169
ILMN_2698239	NM_145959	NaN (S)	D15Ertd621e	DNA segment, Chr 15, ERATO Doi 621, expressed	D15Ertd621e	4.71E-02	1.170
ILMN_2808485	NM_001039646	guanylate-binding protein 10 (Gbp10), mRNA. XM_987647 XM_987685 XM_987723 XM_987759 XM_987793 XM_987820 (S)	Gbp10	predicted gene, EG634650; guanylate-binding protein 10; RIKEN cDNA 5830443L24 gene	Gbp8	1.80E-02	1.170
ILMN_1213457	NM_029721	sorting nexin family member 27 (Snx27), transcript variant 2, mRNA. (S)	Snx27	sorting nexin family member 27	SNX27	4.31E-02	1.170
ILMN_2652857	NM_008330	interferon gamma inducible protein 47 (Ifi47), mRNA. (S)	Ifi47	interferon gamma inducible protein 47	Ifi47	9.73E-03	1.170
ILMN_2926198	NM_023516	RIKEN cDNA 2310016C08 gene (2310016C08Rik), mRNA. (S)	2310016C08Rik	RIKEN cDNA 2310016C08 gene	2310016C08Rik	4.70E-02	1.170

ILMN_1251724	AK082529	NaN (S)	C230059I24Rik	ring finger protein 20	rnf20	4.56E-03	1.170
ILMN_1243812	NM_031256	pleckstrin homology domain-containing, family A (phosphoinositide binding specific) member 3 (Plekha3), mRNA. (S)	Plekha3	pleckstrin homology domain-containing, family A (phosphoinositide binding specific) member 3	PLEKHA3	3.23E-02	1.171
ILMN_2636853	NM_010135	NaN (S)	Enah	enabled homolog (Drosophila)	Enah	7.32E-03	1.171
ILMN_1258982	AK080830	NaN (S)	Srr	serine racemase	srr	1.45E-02	1.171
ILMN_2821254	NM_145627	RNA binding motif protein 10 (Rbm10), mRNA. (S)	Rbm10	RNA binding motif protein 10; predicted gene 12799	Gm12799	2.80E-02	1.172
ILMN_1238246	NM_026194	RIKEN cDNA 1810074P20 gene (1810074P20Rik), mRNA. (S)	1810074P20Rik	RIKEN cDNA 1810074P20 gene	1810074P20Rik	7.70E-03	1.173
ILMN_1236168	AK081262	NaN (S)	C030034J23Rik	family with sequence similarity 135, member A	FAM135A	4.26E-02	1.173
ILMN_1223190	NM_133917	MLX interacting protein (Mlxip), transcript variant 2, mRNA. (S)	Mlxip	MLX interacting protein	MLXIP	4.54E-03	1.173
ILMN_1247096	AK028914	NaN (S)	Agl	amylo-1,6-glucosidase, 4-alpha-glucanotransferase	agl	5.47E-03	1.175
ILMN_2761128	NM_001081960	CLIP associating protein 2 (Clasp2), transcript variant 2, mRNA. (S)	Clasp2	CLIP associating protein 2	CLASP2	1.10E-02	1.175
ILMN_2757356	NM_183174	homeodomain leucine zipper-encoding gene (Homez), mRNA. (S)	Homez	homeodomain leucine zipper-encoding gene	homez	3.92E-02	1.176
ILMN_3138157	NM_019717	atlastin GTPase 2 (Atl2), transcript variant 1, mRNA. (A)	Atl2	atlastin GTPase 2	atl2	2.76E-02	1.176
ILMN_1242705	AK014735	NaN (S)	4833420G17Rik	RIKEN cDNA 4833420G17 gene	4833420G17Rik	4.66E-02	1.178
ILMN_2689346	NM_001039094	neuronal growth regulator 1 (Negr1), transcript variant 1, mRNA. (S)	Negr1	neuronal growth regulator 1	NEGR1	4.15E-02	1.179
ILMN_2865016	NM_009856	CD83 antigen (Cd83), mRNA. (S)	Cd83	CD83 antigen	CD83	6.26E-03	1.179

ILMN_2675697	NM_134090	KDEL (Lys-Asp-Glu-Leu) endoplasmic reticulum protein retention receptor 3 (Kdelr3), mRNA. (S)	Kdelr3	KDEL (Lys-Asp-Glu-Leu) endoplasmic reticulum protein retention receptor 3	KDELR3	2.43E-02	1.179
ILMN_1240332	NM_146073	zinc finger, DHHC domain containing 14 (Zdhhc14), mRNA. (S)	Zdhhc14	zinc finger, DHHC domain containing 14	Zdhhc14	3.87E-02	1.180
ILMN_2890238	NM_008477	kinectin 1 (Ktn1), mRNA. (S)	Ktn1	kinectin 1	Ktn1	1.03E-03	1.180
ILMN_2629528	NM_015731	ATPase, class II, type 9A (Atp9a), mRNA. (S)	Atp9a	ATPase, class II, type 9A	ATP9A	3.30E-02	1.181
ILMN_2929526	NM_007645	CD37 antigen (Cd37), mRNA. (S)	Cd37	CD37 antigen	CD37	3.06E-02	1.182
ILMN_2764325	NM_175682	RIKEN cDNA 9930021D14 gene (9930021D14Rik), mRNA. (S)	9930021D14Rik	RIKEN cDNA 9930021D14 gene	9930021D14Rik	1.28E-02	1.182
ILMN_2696375	NM_207625	acyl-CoA synthetase long-chain family member 4 (Acsl4), transcript variant 1, mRNA. (S)	Acsl4	acyl-CoA synthetase long-chain family member 4	Acsl4	4.39E-02	1.183
ILMN_1218536	AK005113	NaN (S)	Cops5	COP9 (constitutive photomorphogenic) homolog, subunit 5 (Arabidopsis thaliana)	Cops5	4.99E-02	1.183
ILMN_2976448	NM_144915	RIKEN cDNA E330036I19 gene (E330036I19Rik), mRNA. (S)	E330036I19Rik	diacylglycerol lipase, beta	daglb	4.29E-02	1.183
ILMN_1254855	NM_021461	MAP kinase-interacting serine/threonine kinase 1 (Mknk1), mRNA. (S)	Mknk1	MAP kinase-interacting serine/threonine kinase 1	mknk1	1.10E-02	1.183
ILMN_2766253	NM_020007	muscleblind-like 1 (Drosophila) (Mbnl1), mRNA. (S)	Mbnl1	muscleblind-like 1 (Drosophila)	Mbnl1	1.86E-02	1.183
ILMN_1226017	XM_902349	PREDICTED: RIKEN cDNA 2610035D17 gene (2610035D17Rik), mRNA. (S)	2610035D17Rik	RIKEN cDNA 2610035D17 gene	2610035D17Rik	4.86E-02	1.183
ILMN_2784950	NM_011116	phospholipase D family, member 3 (Pld3), mRNA. (S)	Pld3	phospholipase D family, member 3	Pld3	1.28E-02	1.184
ILMN_2838629	NM_173445	RCC1 domain containing 1 (Rccd1), mRNA. (S)	Rccd1	similar to MYC binding protein 2; RCC1 domain containing 1	RCCD1	3.29E-02	1.185
ILMN_2598585	NM_178590	inhibitor of kappaB kinase gamma (Ikbkg), transcript variant 2, mRNA. (S)	Ikbkg	inhibitor of kappaB kinase gamma	ikbkg	3.69E-02	1.186

ILMN_2834622	NM_007391	acrosomal vesicle protein 1 (Acrv1), mRNA. (S)	Acrv1	acrosomal vesicle protein 1	Acrv1	2.46E-02	1.186
ILMN_1240590	NM_026267	NECAP endocytosis associated 1 (Necap1), mRNA. (S)	Necap1	NECAP endocytosis associated 1	Necap1	2.17E-03	1.186
ILMN_2978533	NM_026485	TraB domain containing (Trabd), mRNA. (S)	Trabd	TraB domain containing	TRABD	3.70E-02	1.187
ILMN_1239040	NaN	NaN (S)	mtDNA_CytB	n/a	n/a	3.13E-02	1.187
ILMN_2630768	NM_021538	coatomer protein complex, subunit epsilon (Cope), mRNA. (S)	Cope	coatomer protein complex, subunit epsilon	cope	8.86E-03	1.187
ILMN_2695047	NM_146251	patatin-like phospholipase domain containing 7 (Pnpla7), mRNA. (S)	Pnpla7	patatin-like phospholipase domain containing 7	PNPLA7	2.80E-02	1.187
ILMN_2645719	NM_199199	transmembrane protein 199 (Tmem199), mRNA. (S)	Tmem199	transmembrane protein 199	TMEM199	4.14E-02	1.187
ILMN_1239479	NaN	NaN (S)	mt-Cytb	n/a	n/a	8.19E-03	1.187
ILMN_2636443	NM_007537	BCL2-like 2 (Bcl2l2), mRNA. (S)	Bcl2l2	BCL2-like 2	Bcl2l2	1.68E-03	1.188
ILMN_2998630	NM_029626	glycosyltransferase 8 domain containing 1 (Glt8d1), mRNA. (S)	Glt8d1	glycosyltransferase 8 domain containing 1	glt8d1	1.10E-04	1.188
ILMN_1231367	NM_153599	cyclin-dependent kinase 8 (Cdk8), mRNA. (S)	Cdk8	predicted gene 7107; cyclin-dependent kinase 8	cdk8	2.99E-02	1.188
ILMN_2613738	NM_026339	NaN (S)	4932415G12Rik	n/a	n/a	5.83E-03	1.188
ILMN_2507044	XM_001002437	PREDICTED: host cell factor C2, transcript variant 1 (Hcfc2), mRNA. (S)	Hcfc2	host cell factor C2	Hcfc2	1.27E-03	1.188
ILMN_2657994	NM_146239	PCTAIRE-motif protein kinase 2 (Pctk2), mRNA. (S)	Pctk2	PCTAIRE-motif protein kinase 2	Cdk17	3.72E-02	1.192
ILMN_2682788	NM_010592	Jun proto-oncogene related gene d1 (Jund1), mRNA. (S)	Jund1	Jun proto-oncogene related gene d	JUND	1.53E-02	1.192
ILMN_2603439	NM_026126	FUN14 domain containing 2 (Fundc2), mRNA. (S)	Fundc2	FUN14 domain containing 2	FUNDC2	1.27E-02	1.192
ILMN_2500548	NM_175394	Wilms' tumour 1-associating protein (Wtap), mRNA. (S)	Wtap	predicted gene 14292; Wilms' tumour 1-associating protein	WTAP	3.38E-02	1.192
ILMN_1230765	NM_013837	protein-tyrosine sulfotransferase 1 (Tpst1), transcript variant 2, mRNA. (S)	Tpst1	protein-tyrosine sulfotransferase 1	TPST1	3.65E-02	1.193

ILMN_2713173	NM_026887	adaptor-related protein complex 1, sigma 2 subunit (Ap1s2), mRNA. (S)	Ap1s2	adaptor-related protein complex 1, sigma 2 subunit	Ap1s2	2.71E-02	1.193
ILMN_1224712	NM_183142	asparagine-linked glycosylation 11 homolog (yeast, alpha-1,2-mannosyltransferase) (Alg11), mRNA. (S)	Alg11	asparagine-linked glycosylation 11 homolog (yeast, alpha-1,2-mannosyltransferase)	alg11	8.38E-03	1.194
ILMN_2700050	NM_178899	HEPACAM family member 2 (Hepacam2), mRNA. (S)	Hepacam2	HEPACAM family member 2	HEPACAM2	1.10E-02	1.194
ILMN_2792351	NM_024442	cytochrome P450, family 4, subfamily f, polypeptide 16 (Cyp4f16), mRNA. (S)	Cyp4f16	cytochrome P450, family 4, subfamily f, polypeptide 16	Cyp4f16	1.19E-02	1.195
ILMN_1218131	NM_183028	protein-L-isoaspartate (D-aspartate) O-methyltransferase domain containing 1 (Pcmtd1), mRNA. (S)	Pcmtd1	protein-L-isoaspartate (D-aspartate) O-methyltransferase domain containing 1	PCMTD1	1.50E-02	1.195
ILMN_2576431	AK048657	NaN (S)	Lpin2	lipin 2	LPIN2	8.30E-03	1.195
ILMN_2666830	NM_007840	NaN (S)	Ddx5	DEAD (Asp-Glu-Ala-Asp) box polypeptide 5; predicted gene 12183	Gm12183	3.75E-02	1.196
ILMN_2446047	NM_027045	granule cell antiserum positive 14 (Gcap14), transcript variant 1, mRNA. (S)	Gcap14	granule cell antiserum positive 14	Gcap14	4.50E-04	1.197
ILMN_2997256	NM_026176	phosducin-like (Pdcl), mRNA. (S)	Pdcl	phosducin-like	PDCL	6.17E-03	1.198
ILMN_1232025	NM_028036	transmembrane and coiled-coil domains 6 (Tmco6), mRNA. (S)	Tmco6	transmembrane and coiled-coil domains 6	TMCO6	1.44E-02	1.198
ILMN_2741277	NM_033174	SNRPN upstream reading frame (Snurf), mRNA. (S)	Snurf	small nuclear ribonucleoprotein N; SNRPN upstream reading frame; predicted gene 5802; similar to SNRPN upstream reading frame protein	Gm5802	4.56E-02	1.198
ILMN_1212838	XM_194114	NaN (S)	Lrig2	n/a	n/a	2.51E-02	1.199
ILMN_3151840	NM_026447	protein phosphatase 1M (Ppm1m), transcript variant 1, mRNA. (A)	Ppm1m	protein phosphatase 1M	PPM1M	1.73E-02	1.199
ILMN_2658610	XM_194372	NaN (S)	AI849286	n/a	n/a	4.05E-02	1.199

ILMN_2557426	AK088592	NaN (S)	E430021A19Rik	solute carrier family 7 (cationic amino acid transporter, y+ system), member 6	SLC7A6	1.44E-02	1.199
ILMN_1250073	NM_023554	nucleolar protein 7 (Nol7), mRNA. (S)	Nol7	nucleolar protein 7	NOL7	9.10E-03	1.199
ILMN_2541708	XM_358711	NaN (S)	LOC382138	n/a	n/a	7.72E-04	1.200
ILMN_2732907	NM_024226	reticulon 4 (Rtn4), transcript variant 4, mRNA. (S)	Rtn4	reticulon 4	rtn4	2.88E-02	1.200
ILMN_2478143	NM_177228	NaN (S)	A130004G07Rik	n/a	n/a	1.88E-02	1.200
ILMN_2913590	NM_025827	lon peptidase 2, peroxisomal (Lonp2), mRNA. XM_001000568 (S)	Lonp2	lon peptidase 2, peroxisomal	LONP2	4.90E-02	1.200
ILMN_2669604	XM_001475233	PREDICTED: RIKEN cDNA 4932438A13 gene (4932438A13Rik), mRNA. (S)	4932438A13Rik	RIKEN cDNA 4932438A13 gene	4932438A13Rik	2.18E-02	1.200
ILMN_1260380	XR_033751	PREDICTED: similar to Orphan nuclear receptor NR1D1 (V-erbA-related protein EAR-1) (Rev-erbA-alpha) (LOC545289), misc RNA. (S)	LOC545289	nuclear receptor subfamily 1, group D, member 2; predicted gene 5827	NR1D2	1.49E-02	1.200
ILMN_1259897	AK035119	NaN (S)	2400010G15Rik	lipase maturation factor 1	LMF1	2.31E-02	1.201
ILMN_1249233	AK038699	NaN (S)	Birc6	baculoviral IAP repeat-containing 6	birc6	2.25E-03	1.201
ILMN_2619574	NM_029875	solute carrier family 35, member E3 (Slc35e3), mRNA. (S)	Slc35e3	solute carrier family 35, member E3; predicted gene 7341	Gm7341	2.64E-02	1.201
ILMN_2496644	XM_001481017	PREDICTED: hypothetical protein LOC100043821 (LOC100043821), mRNA. (S)	LOC100043821	predicted gene 4671	Gm4671	4.38E-02	1.201
ILMN_2901626	NM_178589	tumor necrosis factor receptor superfamily, member 21 (Tnfrsf21), mRNA. (S)	Tnfrsf21	tumor necrosis factor receptor superfamily, member 21	TNFRSF21	2.73E-02	1.202
ILMN_2594926	NM_009999	cytochrome P450, family 2, subfamily b, polypeptide 10 (Cyp2b10), transcript variant 2, mRNA. (S)	Cyp2b10	cytochrome P450, family 2, subfamily b, polypeptide 10	Cyp2b10	3.83E-02	1.202

ILMN_2596887	NM_010747	Yamaguchi sarcoma viral (v-yes-1) oncogene homolog (Lyn), mRNA. (S)	Lyn	Yamaguchi sarcoma viral (v-yes-1) oncogene homolog	LYN	4.03E-02	1.202
ILMN_2460041	NM_001011721	olfactory receptor 102 (Olfr102), mRNA. (S)	Olfr102	olfactory receptor 102; olfactory receptor 100	Olfr102	2.98E-02	1.203
ILMN_2812935	NM_133826	ATPase, H+ transporting, lysosomal V1 subunit H (Atp6v1h), mRNA. (S)	Atp6v1h	ATPase, H+ transporting, lysosomal V1 subunit H	ATP6V1H	1.23E-02	1.203
ILMN_2446383	NM_001025439	calcium/calmodulin-dependent protein kinase II, delta (Camk2d), transcript variant 1, mRNA. (S)	Camk2d	calcium/calmodulin-dependent protein kinase II, delta	CAMK2D	4.36E-02	1.203
ILMN_2479024	NM_153489	ubiquitin associated protein 2-like (Ubap2l), transcript variant 2, mRNA. (S)	Ubap2l	ubiquitin associated protein 2-like	Ubap2l	4.18E-02	1.203
ILMN_1218890	XM_355056	NaN (S)	LOC381140	n/a	n/a	6.70E-03	1.204
ILMN_1229583	NR_003619	RIKEN cDNA 6330549D23 gene (6330549D23Rik), non-coding RNA. (S)	6330549D23Rik	RIKEN cDNA 6330549D23 gene	6330549D23Rik	4.36E-02	1.204
ILMN_2609101	XM_001478484	PREDICTED: hect domain and RLD 5, transcript variant 1 (Herc5), mRNA. (S)	Herc5	hect domain and RLD 5	HERC5	2.12E-02	1.205
ILMN_1218670	AK011501	NaN (S)	Csnk2a1-rs4	casein kinase 2, alpha 1 polypeptide; predicted gene 10031; similar to casein kinase II, alpha 1 polypeptide	LOC677611	8.37E-03	1.205
ILMN_1234929	XM_001473421	PREDICTED: similar to modulator recognition factor 2 (LOC100044968), mRNA. (S)	LOC100044968	similar to modulator recognition factor 2; AT rich interactive domain 5B (MRF1-like)	LOC100044968	2.73E-02	1.206
ILMN_2433676	NM_027314	NaN (S)	2700055A20Rik	predicted gene 7684; membrane-associated ring finger (C3HC4) 5	Gm7684	5.22E-03	1.206
ILMN_2596713	NM_010241	thymoma viral proto-oncogene 1 interacting protein (Aktip), mRNA. (S)	Aktip	thymoma viral proto-oncogene 1 interacting protein	AKTIP	4.58E-02	1.207
ILMN_3104462	NM_001039710	coenzyme Q10 homolog B (S. cerevisiae) (Coq10b), transcript variant 1, mRNA. (A)	Coq10b	hypothetical protein LOC675736; coenzyme Q10 homolog B (S. cerevisiae); predicted gene 4899	Gm4899	1.71E-02	1.208
ILMN_1243370	NM_028057	cytochrome b5 reductase 1 (Cyb5r1), mRNA. (S)	Cyb5r1	cytochrome b5 reductase 1	CYB5R1	2.85E-02	1.208

ILMN_2836924	NM_172372	WD repeat domain 45 (Wdr45), mRNA. (S)	Wdr45	WD repeat domain 45	wdr45	1.99E-02	1.208
ILMN_2636169	NM_001034030	LIM motif-containing protein kinase 2 (Limk2), transcript variant 3, mRNA. (S)	Limk2	LIM motif-containing protein kinase 2	LIMK2	1.95E-02	1.208
ILMN_1246038	NM_144791	torsin A interacting protein 1 (Tor1aip1), mRNA. (S)	Tor1aip1	torsin A interacting protein 1	TOR1AIP1	2.87E-02	1.208
ILMN_2646976	NM_020566	DnaJ (Hsp40) homolog, subfamily C, member 4 (Dnajc4), mRNA. (S)	Dnajc4	DnaJ (Hsp40) homolog, subfamily C, member 4	dnajc4	4.41E-03	1.209
ILMN_1230339	NM_178371	solute carrier family 9 (sodium/hydrogen exchanger), member 8 (Slc9a8), transcript variant 2, mRNA. (S)	Slc9a8	solute carrier family 9 (sodium/hydrogen exchanger), member 8	SLC9A8	2.25E-03	1.209
ILMN_2564084	AK029033	NaN (S)	4732483F24Rik	cyclin L2	Ccnl2	4.69E-02	1.210
ILMN_2864906	NM_026637	RIKEN cDNA A030007L17 gene (A030007L17Rik), mRNA. (S)	A030007L17Rik	gamma-glutamyl cyclotransferase	Ggct	1.92E-02	1.210
ILMN_2525029	NM_138679	ash1 (absent, small, or homeotic)-like (Drosophila) (Ash1l), mRNA. (S)	Ash1l	ash1 (absent, small, or homeotic)-like (Drosophila)	ASH1L	2.64E-02	1.210
ILMN_2648746	NM_009739	branched chain ketoacid dehydrogenase kinase (Bckdk), nuclear gene encoding mitochondrial protein, mRNA. (S)	Bckdk	branched chain ketoacid dehydrogenase kinase	BCKDK	1.80E-02	1.211
ILMN_2854497	NM_011608	tumor necrosis factor receptor superfamily, member 17 (Tnfrsf17), mRNA. (S)	Tnfrsf17	tumor necrosis factor receptor superfamily, member 17	TNFRSF17	1.78E-02	1.212
ILMN_2645165	NM_028643	EF hand domain family A1 (Efha1), mRNA. (S)	Efha1	EF hand domain family A1	Efha1	4.52E-02	1.212
ILMN_2724483	NM_030113	NaN (S)	Arhgap10	Rho GTPase activating protein 10	arhgap10	4.15E-02	1.212
ILMN_1232821	NM_146104	anterior pharynx defective 1a homolog (C. elegans) (Aph1a), transcript variant 1, mRNA. (S)	Aph1a	predicted gene 15429; anterior pharynx defective 1a homolog (C. elegans)	Gm15429	3.59E-02	1.213
ILMN_2621622	NM_007786	NaN (S)	Csnk	casein kappa	CSN3	2.50E-03	1.214
ILMN_2627205	NM_027230	protein kinase C binding protein 1 (Prkcbp1), mRNA. (S)	Prkcbp1	zinc finger, MYND-type containing 8	Zmynd8	2.88E-02	1.214

ILMN_2681984	NM_028791	RIKEN cDNA 1300018I05 gene (1300018I05Rik), mRNA. (S)	1300018I05Rik	RIKEN cDNA 1300018I05 gene; ring finger protein 8	Rnf8	1.34E-02	1.214
ILMN_2635850	NM_026832	cell growth regulator with ring finger domain 1 (Cgrrf1), mRNA. (S)	Cgrrf1	cell growth regulator with ring finger domain 1	cgrrf1	4.14E-02	1.215
ILMN_2553280	AK028922	NaN (S)	4732470M22Rik	RIKEN cDNA 4930523C07 gene	4930523C07Rik	4.46E-03	1.215
ILMN_1222890	XM_001478884	PREDICTED: similar to RAP1, GTP-GDP dissociation stimulator 1 (LOC100047800), mRNA. (S)	LOC100047800	similar to RAP1, GTP-GDP dissociation stimulator 1; RAP1, GTP-GDP dissociation stimulator 1	LOC100047800	3.37E-02	1.215
ILMN_3026397	NM_001025566	choline kinase alpha (Chka), transcript variant 2, mRNA. (I)	Chka	choline kinase alpha	CHKA	3.76E-02	1.216
ILMN_2679662	NM_030245	transcriptional adaptor 1 (HFI1 homolog, yeast) like (Tada1l), mRNA. (S)	Tada1l	transcriptional adaptor 1 (HFI1 homolog, yeast) like	TADA1	1.80E-02	1.216
ILMN_2987564	NM_175212	transmembrane protein 65 (Tmem65), mRNA. (S)	Tmem65	transmembrane protein 65	TMEM65	4.71E-02	1.218
ILMN_2769843	NM_027154	NaN (S)	2310061B02Rik	transmembrane BAX inhibitor motif containing 1	TMBIM1	4.18E-02	1.218
ILMN_2810624	NM_144942	cysteine sulfinic acid decarboxylase (Csad), mRNA. (S)	Csad	cysteine sulfinic acid decarboxylase	CSAD	1.52E-02	1.218
ILMN_2757924	NM_027061	zona pellucida binding protein 2 (Zpbp2), transcript variant 1, mRNA. (S)	Zpbp2	zona pellucida binding protein 2	ZPBP2	4.22E-02	1.219
ILMN_2709525	NM_027276	CDC16 cell division cycle 16 homolog (S. cerevisiae) (Cdc16), mRNA. (S)	Cdc16	CDC16 cell division cycle 16 homolog (S. cerevisiae)	CDC16	4.60E-02	1.220
ILMN_1247302	NM_026543	RIKEN cDNA 3010026O09 gene (3010026O09Rik), mRNA. (S)	3010026O09Rik	RIKEN cDNA 3010026O09 gene	3010026O09Rik	5.23E-04	1.221
ILMN_2941992	NM_011183	presenilin 2 (Psen2), mRNA. (S)	Psen2	presenilin 2	psen2	8.56E-03	1.221
ILMN_2803921	NM_010742	lymphocyte antigen 6 complex, locus D (Ly6d), mRNA. (S)	Ly6d	lymphocyte antigen 6 complex, locus D	LY6D	3.47E-03	1.221
ILMN_2796557	NM_025614	RWD domain containing 1 (Rwdd1), mRNA. (S)	Rwdd1	predicted gene 12693; RWD domain containing 1; predicted gene 13743	Gm13743	9.21E-03	1.221
ILMN_1226541	AK030671	NaN (S)	5430417J04Rik	protease, serine, 35	PRSS35	1.39E-02	1.221

ILMN_1254152	AK036485	NaN (S)	Itgav	integrin alpha V	Itgav	1.08E-02	1.221
ILMN_1220374	XM_910556	PREDICTED: RIKEN cDNA 0610030E20 gene, transcript variant 6 (0610030E20Rik), mRNA. (S)	0610030E20Rik	RIKEN cDNA 0610030E20 gene	0610030E20Rik	7.61E-03	1.221
ILMN_1258453	NM_026664	vacuolar protein sorting 53 (yeast) (Vps53), mRNA. (S)	Vps53	vacuolar protein sorting 53 (yeast)	VPS53	4.31E-02	1.222
ILMN_2683424	NM_008088	growth arrest specific 7 (Gas7), mRNA. (S)	Gas7	growth arrest specific 7	gas7	3.01E-02	1.222
ILMN_2820814	NM_025706	TBC1 domain family, member 15 (Tbc1d15), mRNA. (S)	Tbc1d15	TBC1 domain family, member 15	TBC1D15	1.28E-02	1.223
ILMN_1242794	NaN	NaN (S)	D630014A15Rik	n/a	n/a	2.82E-02	1.223
ILMN_2740069	NM_028478	Ras association (RalGDS/AF-6) domain family member 6 (Rassf6), mRNA. (S)	Rassf6	Ras association (RalGDS/AF-6) domain family member 6	rassf6	1.15E-02	1.223
ILMN_1216953	NM_001077638	protein arginine N-methyltransferase 2 (Prmt2), transcript variant 2, mRNA. (S)	Prmt2	protein arginine N-methyltransferase 2	PRMT2	1.69E-02	1.224
ILMN_3111298	NM_139295	multiple coagulation factor deficiency 2 (Mcfd2), mRNA. (A)	Mcfd2	multiple coagulation factor deficiency 2	MCFD2	4.24E-02	1.225
ILMN_2731940	NM_011508	NaN (S)	Sui1-rs1	predicted gene 5265; similar to suppressor of initiator codon mutations, related sequence 1; predicted gene 4017; predicted gene 6913; predicted gene 7688; predicted gene 6535; predicted gene 6900; predicted gene 5471; predicted gene 7845; predicted gene 5450; predicted gene 6155; predicted gene 7253; predicted gene 10713; similar to translation initiation factor SUI1; predicted gene 6428; similar to Eukaryotic translation initiation factor 1 (eIF1) (Protein translation factor SUI1 homolog); predicted gene, EG434356; similar to isolog of yeast sui1 and rice gos2; putative; eukaryotic translation initiation factor 1	Gm5265	2.39E-02	1.225
ILMN_2676615	NM_013867	breast cancer anti-estrogen resistance 3 (Bcar3), mRNA. (S)	Bcar3	breast cancer anti-estrogen resistance 3	bcar3	2.76E-03	1.225
ILMN_2457194	NM_026123	unc-50 homolog (C. elegans) (Unc50), mRNA. (S)	Unc50	unc-50 homolog (C. elegans)	UNC50	7.98E-03	1.225

ILMN_1238739	XM_283218	NaN (S)	Kif13b	n/a	n/a	2.04E-03	1.225
ILMN_1253258	AK052304	NaN (S)	Cradd	CASP2 and RIPK1 domain containing adaptor with death domain	CRADD	3.64E-02	1.226
ILMN_2777668	NM_015801	NaN (S)	Nte	patatin-like phospholipase domain containing 6	PNPLA6	2.95E-02	1.226
ILMN_1236317	NM_033078	killer cell lectin-like receptor subfamily K, member 1 (Klrk1), mRNA. (S)	Klrk1	killer cell lectin-like receptor subfamily K, member 1	KLRK1	3.00E-02	1.226
ILMN_2523794	NM_177055	NaN (S)	A630001G21Rik	RIKEN cDNA A630001G21 gene	A630001G21Rik	7.39E-05	1.226
ILMN_1217118	NM_032003	ectonucleotide pyrophosphatase/phosphodiesterase 5 (Enpp5), mRNA. (S)	Enpp5	ectonucleotide pyrophosphatase/phosphodiesterase 5	Enpp5	2.58E-02	1.226
ILMN_2998230	NM_016722	galactosamine (N-acetyl)-6-sulfate sulfatase (Galns), mRNA. (S)	Galns	galactosamine (N-acetyl)-6-sulfate sulfatase	GALNS	3.68E-02	1.227
ILMN_2878274	NM_019877	coatomer protein complex, subunit zeta 2 (Copz2), mRNA. (S)	Copz2	coatomer protein complex, subunit zeta 2	copz2	2.27E-02	1.227
ILMN_2594525	NM_010941	NAD(P) dependent steroid dehydrogenase-like (Nsdhl), mRNA. (S)	Nsdhl	NAD(P) dependent steroid dehydrogenase-like	nsdhl	3.54E-02	1.227
ILMN_1247789	NM_145546	general transcription factor IIB (Gtf2b), mRNA. (S)	Gtf2b	general transcription factor IIB	Gtf2b	1.40E-02	1.227
ILMN_2512849	NM_025641	ubiquinol-cytochrome c reductase hinge protein (Uqcrh), mRNA. (S)	Uqcrh	predicted gene 14088; ubiquinol-cytochrome c reductase hinge protein	Uqcrh	1.21E-02	1.228
ILMN_1213882	XR_034180	PREDICTED: similar to RCC1 domain containing 1 (LOC675567), misc RNA. (S)	LOC675567	similar to MYC binding protein 2; RCC1 domain containing 1	RCCD1	2.79E-02	1.228
ILMN_1247075	NM_008720	Niemann Pick type C1 (Npc1), mRNA. (S)	Npc1	Niemann Pick type C1	npc1	4.29E-02	1.229
ILMN_1214450	NM_001081054	glutaminyl-tRNA synthase (glutamine-hydrolyzing)-like 1 (Qrsl1), mRNA. (S)	Qrsl1	glutaminyl-tRNA synthase (glutamine-hydrolyzing)-like 1	qrsl1	4.98E-02	1.229
ILMN_3161289	NM_010311	guanine nucleotide binding protein, alpha z subunit (Gnaz), mRNA. (S)	Gnaz	guanine nucleotide binding protein, alpha z subunit	GNAZ	1.07E-02	1.230
ILMN_2516531	NM_009535	NaN (S)	Yes	Yamaguchi sarcoma viral (v-yes) oncogene homolog 1	YES1	7.18E-03	1.231

ILMN_2783852	NM_181039	latrophilin 1 (Lphn1), mRNA. (S)	Lphn1	latrophilin 1	LPHN1	3.91E-02	1.233
ILMN_2774456	NM_026452	coenzyme Q9 homolog (yeast) (Coq9), mRNA. (S)	Coq9	coenzyme Q9 homolog (yeast)	Coq9	2.02E-02	1.233
ILMN_2747912	NM_009000	RAB24, member RAS oncogene family (Rab24), mRNA. (S)	Rab24	RAB24, member RAS oncogene family	RAB24	1.29E-02	1.234
ILMN_2822071	NM_175194	solute carrier family 25 (mitochondrial carrier, Graves disease autoantigen), member 16 (Slc25a16), nuclear gene encoding mitochondrial protein, mRNA. (S)	Slc25a16	solute carrier family 25 (mitochondrial carrier, Graves disease autoantigen), member 16	slc25a16	1.56E-02	1.236
ILMN_1240829	AK018753	NaN (S)	0610040O15Rik	NADH-ubiquinone oxidoreductase chain 1	ND1	1.58E-02	1.238
ILMN_1239181	NM_026393	NmrA-like family domain containing 1 (Nmral1), mRNA. (S)	Nmral1	NmrA-like family domain containing 1	NMRAL1	1.46E-02	1.238
ILMN_1219253	NM_172907	olfactomedin-like 1 (Olfml1), mRNA. (S)	Olfml1	olfactomedin-like 1	OLFML1	2.92E-02	1.238
ILMN_1244857	NM_010261	Rab acceptor 1 (prenylated) (Rabac1), mRNA. (S)	Rabac1	Rab acceptor 1 (prenylated)	rabac1	3.19E-03	1.238
ILMN_1239906	NM_023872	potassium voltage-gated channel, subfamily Q, member 5 (Kcnq5), mRNA. (S)	Kcnq5	potassium voltage-gated channel, subfamily Q, member 5	KCNQ5	2.73E-02	1.238
ILMN_2677056	XM_001480443	PREDICTED: similar to EF-hand Ca2+ binding protein p22 (LOC100048622), mRNA. (S)	LOC100048622	RIKEN cDNA 1500003O03 gene; similar to EF-hand Ca2+ binding protein p22	LOC100048622	4.97E-02	1.238
ILMN_2482896	NM_025476	NaN (S)	2410005O16Rik	family with sequence similarity 82, member B	Fam82b	3.41E-02	1.238
ILMN_2619316	NM_011170	prion protein (Prnp), mRNA. (S)	Prnp	prion protein	PRNP	4.05E-02	1.239
ILMN_2771201	NM_016722	NaN (S)	Galns	galactosamine (N-acetyl)-6-sulfate sulfatase	GALNS	4.34E-02	1.239
ILMN_2747897	NM_026688	NADH dehydrogenase (ubiquinone) Fe-S protein 3 (Ndufs3), mRNA. (S)	Ndufs3	similar to NADH dehydrogenase (ubiquinone) Fe-S protein 3; NADH dehydrogenase (ubiquinone) Fe-S protein 3; predicted gene 12251	Gm12251	1.36E-02	1.239

ILMN_1224252	XM_001003228	PREDICTED: RIKEN cDNA 9030624G23 gene (9030624G23Rik), mRNA. (S)	9030624G23Rik	RIKEN cDNA 9030624G23 gene; RIKEN cDNA 6030426L16 gene; similar to Hippocalcin-like protein 1 (Visinin-like protein 3) (VILIP-3) (Neural visinin-like protein 3) (NVL-3) (NVP-3); similar to development and differentiation enhancing factor 2; predicted gene 5784; predicted gene 9295	Gm9295	3.90E-02	1.239
ILMN_1254426	XM_284098	NaN (S)	1110003E08Rik	n/a	n/a	6.91E-03	1.240
ILMN_2633223	XM_484351	NaN (S)	1810063B07Rik	n/a	n/a	6.13E-03	1.240
ILMN_2721692	NM_175121	solute carrier family 38, member 2 (Slc38a2), mRNA. (S)	Slc38a2	solute carrier family 38, member 2	slc38a2	2.97E-02	1.240
ILMN_1252725	AK087438	NaN (S)	E130118D18Rik	family with sequence similarity 120, member B	Fam120b	2.34E-02	1.240
ILMN_1221142	NM_011145	peroxisome proliferator activator receptor delta (Ppard), mRNA. (S)	Ppard	peroxisome proliferator activator receptor delta	Ppard	9.02E-03	1.240
ILMN_1242508	XM_135990	NaN (S)	Pin4	n/a	n/a	4.79E-02	1.241
ILMN_2707107	NM_178879	UDP-GlcNAc:betaGal beta-1,3-N-acetylglucosaminyltransferase 9 (B3gnt9), mRNA. (S)	B3gnt9	UDP-GlcNAc:betaGal beta-1,3-N-acetylglucosaminyltransferase 9	B3gnt9	2.67E-03	1.241
ILMN_1248555	NM_029364	glucosamine (N-acetyl)-6-sulfatase (Gns), mRNA. (S)	Gns	glucosamine (N-acetyl)-6-sulfatase	GNS	4.16E-02	1.241
ILMN_1213681	NM_024179	RIKEN cDNA 0610009O20 gene (0610009O20Rik), mRNA. (S)	0610009O20Rik	RIKEN cDNA 0610009O20 gene	0610009O20Rik	3.14E-02	1.241
ILMN_2649172	NM_025893	zinc finger, CCHC domain containing 18 (Zcchc18), transcript variant 3, mRNA. (S)	Zcchc18	zinc finger, CCHC domain containing 18	Zcchc18	4.81E-02	1.242
ILMN_2954195	NM_023912	SCY1-like 1 (S. cerevisiae) (Scyl1), mRNA. (S)	Scyl1	SCY1-like 1 (S. cerevisiae)	scyl1	4.96E-02	1.242
ILMN_1230916	XR_033167	PREDICTED: similar to HSPC008 (LOC433955), misc RNA. (S)	LOC433955	predicted gene 5564	Gm5564	2.79E-02	1.242
ILMN_1232272	NM_133708	GDP-mannose pyrophosphorylase A (Gmppa), mRNA. (S)	Gmppa	GDP-mannose pyrophosphorylase A	Gmppa	7.19E-03	1.242
ILMN_1241902	NM_138952	receptor (TNFRSF)-interacting serine-threonine kinase 2 (Ripk2), mRNA. (S)	Ripk2	receptor (TNFRSF)-interacting serine-threonine kinase 2	RIPK2	9.23E-03	1.242

ILMN_2601488	NM_207678	cyclin L2 (Ccnl2), mRNA. (S)	Ccnl2	cyclin L2	Ccnl2	2.14E-02	1.242
ILMN_2680415	NM_172537	sema domain, transmembrane domain (TM), and cytoplasmic domain, (semaphorin) 6D (Sema6d), transcript variant 1, mRNA. (S)	Sema6d	sema domain, transmembrane domain (TM), and cytoplasmic domain, (semaphorin) 6D	SEMA6D	2.27E-02	1.243
ILMN_2630769	NM_021538	coatomer protein complex, subunit epsilon (Cope), mRNA. (S)	Cope	coatomer protein complex, subunit epsilon	cope	1.97E-02	1.243
ILMN_2690804	XM_489220	NaN (S)	4933430N04Rik	n/a	n/a	2.95E-02	1.244
ILMN_1216097	XM_924862	PREDICTED: gene model 684, (NCBI) (Gm684), mRNA. (S)	Gm684	predicted gene 684	Gm684	7.54E-03	1.244
ILMN_2613731	NM_026339	NaN (S)	4932415G12Rik	n/a	n/a	7.88E-04	1.244
ILMN_2699645	NM_194054	reticulon 4 (Rtn4), transcript variant 1, mRNA. (S)	Rtn4	reticulon 4	rtn4	3.84E-02	1.244
ILMN_2631143	NM_011444	SRY-box containing gene 5 (Sox5), mRNA. (S)	Sox5	SRY-box containing gene 5	SOX5	3.20E-02	1.244
ILMN_2493030	NR_003513	RIKEN cDNA 2310043N10 gene (2310043N10Rik), non-coding RNA. (S)	2310043N10Rik	RIKEN cDNA 2310043N10 gene	Neat1	3.92E-03	1.244
ILMN_2701855	NM_029629	fumarylacetoacetate hydrolase domain containing 2A (Fahd2a), mRNA. (S)	Fahd2a	fumarylacetoacetate hydrolase domain containing 2A	Fahd2a	7.63E-03	1.244
ILMN_2741114	NM_010368	glucuronidase, beta (Gusb), mRNA. (S)	Gusb	glucuronidase, beta	GUSB	6.35E-03	1.246
ILMN_2757224	NM_025527	signal recognition particle 19 (Srp19), mRNA. (S)	Srp19	signal recognition particle 19	SRP19	4.85E-02	1.246
ILMN_2679139	XM_129331	NaN (S)	Rab11fip2	n/a	n/a	2.69E-02	1.246
ILMN_1236461	NM_001081225	family with sequence similarity 178, member A (Fam178a), mRNA. (S)	Fam178a	family with sequence similarity 178, member A	Fam178a	3.43E-02	1.246
ILMN_2844370	NM_173751	ilvB (bacterial acetolactate synthase)-like (Ilvbl), mRNA. (S)	Ilvbl	ilvB (bacterial acetolactate synthase)-like	ILVBL	4.77E-02	1.246
ILMN_2499166	NM_175212	transmembrane protein 65 (Tmem65), mRNA. (S)	Tmem65	transmembrane protein 65	TMEM65	3.61E-02	1.247

ILMN_1240074	NM_001025599	tripartite motif-containing 26 (Trim26), transcript variant 1, mRNA. (S)	Trim26	tripartite motif-containing 26	TRIM26	1.90E-02	1.248
ILMN_1228783	XR_031250	PREDICTED: hypothetical protein LOC100043986 (LOC100043986), misc RNA. (S)	LOC100043986	hypothetical protein LOC100043986; RIKEN cDNA C630004H02 gene	C630004H02Rik	3.59E-02	1.249
ILMN_1226293	NM_028064	solute carrier family 39 (zinc transporter), member 4 (Slc39a4), mRNA. (S)	Slc39a4	solute carrier family 39 (zinc transporter), member 4	SLC39A4	1.83E-02	1.249
ILMN_2519780	NaN	NaN (S)	Fin14	n/a	n/a	3.50E-02	1.250
ILMN_2781878	NM_033607	ubiquitin carboxyl-terminal esterase L4 (Uchl4), mRNA. (S)	Uchl4	ubiquitin carboxyl-terminal esterase L4	Uchl4	1.39E-02	1.250
ILMN_2487934	NM_028173	translocating chain-associating membrane protein 1 (Tram1), mRNA. (S)	Tram1	translocating chain-associating membrane protein 1	TRAM1	2.00E-02	1.250
ILMN_2669813	NM_133865	DNA cross-link repair 1B, PSO2 homolog (S. cerevisiae) (Dclre1b), transcript variant 1, mRNA. (S)	Dclre1b	DNA cross-link repair 1B, PSO2 homolog (S. cerevisiae)	DCLRE1B	2.00E-02	1.250
ILMN_1256023	AK039083	NaN (S)	A230093N12Rik	fatty acyl CoA reductase 1	far1	4.58E-03	1.250
ILMN_1253618	NM_016892	copper chaperone for superoxide dismutase (Ccs), mRNA. (S)	Ccs	copper chaperone for superoxide dismutase	Ccs	2.82E-02	1.252
ILMN_2771087	NM_053090	NaN (S)	Drctnnb1a	family with sequence similarity 126, member A	FAM126A	1.44E-02	1.252
ILMN_2459291	BC059847	NaN (S)	scl0001562.1_73	Rap guanine nucleotide exchange factor (GEF) 6	RAPGEF6	4.66E-02	1.253
ILMN_2705166	NM_145933	beta galactoside alpha 2,6 sialyltransferase 1 (St6gal1), mRNA. (S)	St6gal1	beta galactoside alpha 2,6 sialyltransferase 1	ST6GAL1	4.58E-02	1.253
ILMN_2694245	NM_009273	signal recognition particle 14 (Srp14), mRNA. (S)	Srp14	signal recognition particle 14	SRP14	4.32E-02	1.254
ILMN_2740852	NM_010169	coagulation factor II (thrombin) receptor (F2r), mRNA. (S)	F2r	coagulation factor II (thrombin) receptor	F2R	6.72E-03	1.254
ILMN_3161066	NM_019403	ring finger protein 5 (Rnf5), mRNA. (S)	Rnf5	ring finger protein 5	Rnf5	4.47E-02	1.255
ILMN_2482897	NM_025476	NaN (S)	2410005O16Rik	family with sequence similarity 82, member B	Fam82b	4.50E-02	1.255

ILMN_1255974	NM_001038700	formin binding protein 1 (Fnbp1), transcript variant 1, mRNA. (S)	Fnbp1	formin binding protein 1	Fnbp1	4.07E-02	1.255
ILMN_1253692	NM_009149	golgi apparatus protein 1 (Glg1), mRNA. (S)	Glg1	golgi apparatus protein 1	GLG1	4.24E-02	1.256
ILMN_2764920	NM_024180	ORM1-like 2 (S. cerevisiae) (Ormdl2), mRNA. (S)	Ormdl2	ORM1-like 2 (S. cerevisiae); predicted gene 5553	Gm5553	3.08E-02	1.257
ILMN_2755424	NM_178782	BCL6 co-repressor-like 1 (Bcorl1), mRNA. (S)	Bcorl1	BCL6 co-repressor-like 1	BCORL1	1.01E-02	1.258
ILMN_1247704	NM_175074	high mobility group nucleosomal binding domain 3 (Hmgn3), transcript variant 2, mRNA. (S)	Hmgn3	high mobility group nucleosomal binding domain 3	HMGN3	4.53E-02	1.258
ILMN_1228937	NM_180962	cysteine and histidine rich 1 (Cyhr1), transcript variant 2, mRNA. (S)	Cyhr1	cysteine and histidine rich 1	CYHR1	3.95E-02	1.258
ILMN_2702406	NM_008842	proviral integration site 1 (Pim1), mRNA. (S)	Pim1	proviral integration site 1	Pim1	2.93E-02	1.258
ILMN_2687799	NM_138607	DNA segment, human DXS9928E (D0HXS9928E), mRNA. (S)	D0HXS9928E	family with sequence similarity 50, member A	Fam50a	9.78E-03	1.259
ILMN_3048492	NM_176839	ATP-binding cassette, sub-family C (CFTR/MRP), member 5 (Abcc5), transcript variant 2, mRNA. (I)	Abcc5	ATP-binding cassette, sub-family C (CFTR/MRP), member 5	abcc5	3.44E-02	1.260
ILMN_2974064	NM_027881	oxysterol binding protein-like 3 (Osbpl3), mRNA. (S)	Osbpl3	oxysterol binding protein-like 3	OSBPL3	1.85E-02	1.260
ILMN_2781602	NM_025409	immediate early response 3 interacting protein 1 (Ier3ip1), mRNA. (S)	Ier3ip1	immediate early response 3 interacting protein 1; haloacid dehalogenase-like hydrolase domain containing 2; predicted gene 10784	Gm10784	3.19E-03	1.260
ILMN_1247777	AK010307	NaN (S)	2400010C15Rik	NADH dehydrogenase (ubiquinone) 1, alpha/beta subcomplex, 1; predicted gene 4459	NDUFAB1	2.21E-02	1.261
ILMN_2564650	AK037457	NaN (S)	Sin3a	n/a	n/a	1.58E-02	1.261
ILMN_2597778	NM_008137	guanine nucleotide binding protein, alpha 14 (Gna14), mRNA. (S)	Gna14	guanine nucleotide binding protein, alpha 14	GNA14	1.97E-02	1.261
ILMN_3138057	NM_183172	resistance to inhibitors of cholinesterase 8 homolog B (C. elegans) (Ric8b), transcript variant 2, mRNA. (A)	Ric8b	resistance to inhibitors of cholinesterase 8 homolog B (C. elegans)	RIC8B	3.01E-02	1.261

ILMN_2812087	NM_134083	regulator of chromosome condensation (RCC1) and BTB (POZ) domain containing protein 2 (Rcbtb2), mRNA. (S)	Rcbtb2	regulator of chromosome condensation (RCC1) and BTB (POZ) domain containing protein 2	rcbtb2	4.85E-02	1.261
ILMN_2608622	NM_178879	UDP-GlcNAc:betaGal beta-1,3-N-acetylglucosaminyltransferase 9 (B3gnt9), mRNA. (S)	B3gnt9	UDP-GlcNAc:betaGal beta-1,3-N-acetylglucosaminyltransferase 9	B3gnt9	7.23E-03	1.261
ILMN_1225552	NM_009713	arylsulfatase A (Arsa), mRNA. (S)	Arsa	arylsulfatase A	arsA	1.61E-04	1.262
ILMN_2650299	XM_001481049	PREDICTED: similar to SEC23B (LOC100048726), mRNA. (S)	LOC100048726	similar to SEC23B; SEC23B (S. cerevisiae)	LOC100048726	6.33E-04	1.263
ILMN_1216674	XM_125096	NaN (S)	LOC234882	n/a	n/a	3.77E-02	1.264
ILMN_2605601	NM_134111	ELL associated factor 2 (Eaf2), transcript variant 1, mRNA. (S)	Eaf2	ELL associated factor 2	EAF2	1.41E-02	1.264
ILMN_1237338	NM_013932	DEAD (Asp-Glu-Ala-Asp) box polypeptide 25 (Ddx25), mRNA. (S)	Ddx25	DEAD (Asp-Glu-Ala-Asp) box polypeptide 25	DDX25	3.55E-02	1.265
ILMN_2751215	NM_144791	torsin A interacting protein 1 (Tor1aip1), mRNA. (S)	Tor1aip1	torsin A interacting protein 1	TOR1AIP1	3.82E-02	1.265
ILMN_2969919	NM_013876	ring finger protein 11 (Rnf11), mRNA. (S)	Rnf11	ring finger protein 11	RNF11	1.86E-02	1.267
ILMN_2489405	NM_021421	NaN (S)	D1Ertd396e	angel homolog 2 (Drosophila)	ANGEL2	7.89E-04	1.267
ILMN_2746107	NM_175145	transmembrane protein 127 (Tmem127), mRNA. (S)	Tmem127	transmembrane protein 127	Tmem127	4.03E-02	1.267
ILMN_2623855	NM_133236	glucocorticoid induced transcript 1 (Glcci1), transcript variant 1, mRNA. (S)	Glcci1	similar to glucocorticoid induced transcript 1; predicted gene 5815; glucocorticoid induced transcript 1	LOC100046012	2.90E-02	1.269
ILMN_2745860	XM_354966	NaN (S)	1700116B05Rik	n/a	n/a	3.20E-02	1.269
ILMN_2440803	NM_019776	staphylococcal nuclease and tudor domain containing 1 (Snd1), mRNA. (S)	Snd1	staphylococcal nuclease and tudor domain containing 1	SND1	1.45E-03	1.270
ILMN_1249290	NM_009620	a disintegrin and metallopeptidase domain 4 (Adam4), mRNA. (S)	Adam4	predicted gene 5746; a disintegrin and metallopeptidase domain 4	Adam4	4.96E-02	1.270

ILMN_2644719	NM_026122	high mobility group nucleosomal binding domain 3 (Hmgn3), transcript variant 1, mRNA. (S)	Hmgn3	high mobility group nucleosomal binding domain 3	HMGN3	4.15E-02	1.270
ILMN_2817151	NM_183270	coiled-coil-helix-coiled-coil-helix domain containing 8 (Chchd8), mRNA. (S)	Chchd8	coiled-coil-helix-coiled-coil-helix domain containing 8	CHCHD8	2.70E-02	1.270
ILMN_2682307	NM_028793	acyl-Coenzyme A binding domain containing 5 (Acbd5), mRNA. (S)	Acbd5	acyl-Coenzyme A binding domain containing 5	acbd5	2.69E-02	1.271
ILMN_1219581	XM_484944	NaN (S)	B230369F24Rik	n/a	n/a	1.65E-02	1.271
ILMN_2604383	NM_007824	cytochrome P450, family 7, subfamily a, polypeptide 1 (Cyp7a1), mRNA. (S)	Cyp7a1	cytochrome P450, family 7, subfamily a, polypeptide 1	Cyp7a1	4.43E-02	1.271
ILMN_2606619	NM_153197	C-type lectin domain family 4, member a3 (Clec4a3), mRNA. (S)	Clec4a3	C-type lectin domain family 4, member a3	Clec4a3	3.19E-02	1.271
ILMN_1225658	NM_011908	ubiquitin-like 3 (Ubl3), mRNA. (S)	Ubl3	ubiquitin-like 3	ubl3	3.27E-02	1.271
ILMN_1222379	NM_009848	NaN (S)	Entpd1	ectonucleoside triphosphate diphosphohydrolase 1	ENTPD1	1.48E-02	1.271
ILMN_1237280	NM_018822	N-sulfoglucosamine sulfohydrolase (sulfamidase) (Sgsh), mRNA. (S)	Sgsh	N-sulfoglucosamine sulfohydrolase (sulfamidase)	SGSH	4.42E-02	1.272
ILMN_1257445	NM_001081326	amylo-1,6-glucosidase, 4-alpha-glucanotransferase (Agl), mRNA. (S)	Agl	amylo-1,6-glucosidase, 4-alpha-glucanotransferase	agl	8.05E-04	1.272
ILMN_1216099	NM_028651	transmembrane and tetratricopeptide repeat containing 4 (Tmtc4), mRNA. (S)	Tmtc4	transmembrane and tetratricopeptide repeat containing 4	Tmtc4	3.45E-04	1.273
ILMN_2432092	NM_013852	ATP-binding cassette, sub-family F (GCN20), member 3 (Abcf3), mRNA. (S)	Abcf3	ATP-binding cassette, sub-family F (GCN20), member 3	ABCF3	1.26E-02	1.273
ILMN_1214335	XR_033995	PREDICTED: predicted gene, EG666160 (EG666160), misc RNA. (S)	EG666160	predicted gene 7957	Gm7957	3.30E-02	1.274
ILMN_3008406	NM_013614	ornithine decarboxylase, structural 1 (Odc1), mRNA. (S)	Odc1	predicted gene 6742; ornithine decarboxylase, structural 1; similar to Ornithine decarboxylase (ODC); predicted gene 7993; predicted gene 15645; predicted gene 9115; predicted gene 7278	Gm7278	4.98E-02	1.274

ILMN_2639291	XM_127882	NaN (S)	Cln5	n/a	n/a	2.71E-02	1.274
ILMN_2576317	AK048383	NaN (S)	C130054H24Rik	ring finger and CCCH-type zinc finger domains 2	RC3H2	4.91E-02	1.275
ILMN_2770414	NM_017475	Ras-related GTP binding C (Rragc), mRNA. (S)	Rragc	Ras-related GTP binding C	RRAGC	3.52E-02	1.275
ILMN_2597272	XM_001476583	PREDICTED: similar to Bcl2-like protein (LOC100046608), mRNA. (S)	LOC100046608	predicted gene 3655; B-cell leukemia/lymphoma 2	BCL2	2.51E-02	1.276
ILMN_2756414	NM_009782	NaN (S)	Cacna1e	calcium channel, voltage-dependent, R type, alpha 1E subunit	CACNA1E	4.75E-03	1.276
ILMN_2984462	NM_011729	excision repair cross-complementing rodent repair deficiency, complementation group 5 (Ercc5), mRNA. (S)	Ercc5	excision repair cross-complementing rodent repair deficiency, complementation group 5	ERCC5	4.16E-02	1.276
ILMN_2522759	XR_034929	PREDICTED: similar to E74-like factor 1 (LOC626061), misc RNA. (S)	LOC626061	predicted gene 6649	Gm6649	3.30E-02	1.277
ILMN_2511971	NM_001001735	Wolf-Hirschhorn syndrome candidate 1-like 1 (human) (Whsc1l1), transcript variant 1, mRNA. (S)	Whsc1l1	Wolf-Hirschhorn syndrome candidate 1-like 1 (human)	WHSC1L1	2.96E-02	1.278
ILMN_2636403	NM_153287	AXIN1 up-regulated 1 (Axud1), mRNA. (S)	Axud1	cysteine-serine-rich nuclear protein 1	CSRNP1	3.92E-02	1.279
ILMN_1219572	NM_001024622	PEST proteolytic signal containing nuclear protein (Pcnp), mRNA. (S)	Pcnp	PEST proteolytic signal containing nuclear protein	PCNP	2.59E-02	1.279
ILMN_3129526	NM_133247	ubiquitin specific peptidase 33 (Usp33), transcript variant 1, mRNA. (A)	Usp33	ubiquitin specific peptidase 33	usp33	2.81E-02	1.279
ILMN_1244219	XM_133608	NaN (S)	LOC233637	n/a	n/a	4.48E-03	1.280
ILMN_1237028	NM_145537	ER degradation enhancer, mannosidase alpha-like 2 (Edem2), mRNA. (S)	Edem2	ER degradation enhancer, mannosidase alpha-like 2	EDEM2	4.38E-04	1.280
ILMN_2660715	NM_177020	NaN (S)	E030011O05Rik	RIKEN cDNA E030011O05 gene	E030011O05Rik	2.23E-04	1.281
ILMN_2952841	NM_133758	ubiquitin specific peptidase 47 (Usp47), mRNA. (S)	Usp47	ubiquitin specific peptidase 47	usp47	3.25E-02	1.281

ILMN_2755059	NM_133749	RIKEN cDNA 2900064A13 gene (2900064A13Rik), mRNA. (S)	2900064A13Rik	RIKEN cDNA 2900064A13 gene	2900064A13Rik	2.64E-02	1.282
ILMN_1250418	NM_008720	Niemann Pick type C1 (Npc1), mRNA. (S)	Npc1	Niemann Pick type C1	npc1	3.85E-02	1.282
ILMN_2633062	NM_025782	NaN (S)	9130422G05Rik	n/a	n/a	2.83E-02	1.282
ILMN_2649286	XM_991819	PREDICTED: similar to Ig kappa chain V-V region MOPC 41 precursor (LOC243431), mRNA. (S)	LOC243431	predicted gene 4966	Gm4966	2.81E-02	1.282
ILMN_2643377	NM_133221	solute carrier family 24 (sodium/potassium/calcium exchanger), member 6 (Slc24a6), mRNA. (S)	Slc24a6	solute carrier family 24 (sodium/potassium/calcium exchanger), member 6	SLC24A6	1.32E-02	1.283
ILMN_1241535	NM_019786	TANK-binding kinase 1 (Tbk1), mRNA. (S)	Tbk1	TANK-binding kinase 1	Tbk1	2.35E-02	1.285
ILMN_2962273	NM_008020	FK506 binding protein 2 (Fkbp2), mRNA. (S)	Fkbp2	FK506 binding protein 2	Fkbp2	1.72E-02	1.285
ILMN_2851710	NM_007714	CDC like kinase 4 (Clk4), mRNA. (S)	Clk4	CDC like kinase 4	clk4	3.87E-02	1.285
ILMN_1259949	NM_028027	DNA segment, Chr 10, ERATO Doi 610, expressed (D10Ertd610e), mRNA. (S)	D10Ertd610e	DNA segment, Chr 10, ERATO Doi 610, expressed	D10Ertd610e	3.81E-02	1.285
ILMN_2925169	NM_013548	histone cluster 1, H3f (Hist1h3f), mRNA. (S)	Hist1h3f	histone cluster 2, H3b; histone cluster 1, H3f; histone cluster 1, H3e; histone cluster 2, H3c1; histone cluster 1, H3d; histone cluster 1, H3c; histone cluster 1, H3b; histone cluster 2, H3c2; histone cluster 2, H2aa1; histone cluster 2, H2aa2	HIST2H3B	3.40E-02	1.286
ILMN_2618634	XM_207079	NaN (S)	Spna2	n/a	n/a	4.25E-02	1.286
ILMN_2638404	NM_133865	DNA cross-link repair 1B, PSO2 homolog (S. cerevisiae) (Dclre1b), transcript variant 1, mRNA. (S)	Dclre1b	DNA cross-link repair 1B, PSO2 homolog (S. cerevisiae)	DCLRE1B	1.37E-02	1.286
ILMN_1222492	NM_027436	mitochondrial intermediate peptidase (Mipep), mRNA. (S)	Mipep	mitochondrial intermediate peptidase; similar to Mipep protein	LOC100047713	3.73E-02	1.287
ILMN_2717199	NM_009143	stromal cell derived factor 2 (Sdf2), mRNA. (S)	Sdf2	stromal cell derived factor 2	SDF2	3.04E-03	1.287
ILMN_2724530	NM_016881	NaN (S)	Pmm2	phosphomannomutase 2	PMM2	2.04E-02	1.287

ILMN_2457727	NM_007419	NaN (S)	Adrb1	adrenergic receptor, beta 1	ADRB1	4.36E-02	1.289
ILMN_1220032	AK080545	NaN (S)	A730082L10Rik	zinc finger protein 692	Zfp692	1.58E-03	1.289
ILMN_1234702	NM_173866	glutamic pyruvate transaminase (alanine aminotransferase) 2 (Gpt2), mRNA. (S)	Gpt2	glutamic pyruvate transaminase (alanine aminotransferase) 2	GPT2	7.24E-04	1.289
ILMN_2706176	NM_133236	glucocorticoid induced transcript 1 (Glcci1), transcript variant 1, mRNA. (S)	Glcci1	similar to glucocorticoid induced transcript 1; predicted gene 5815; glucocorticoid induced transcript 1	LOC100046012	2.78E-02	1.289
ILMN_1232766	NM_178653	saccharopine dehydrogenase (putative) (Sccpdh), mRNA. (S)	Sccpdh	similar to Saccharopine dehydrogenase (putative); saccharopine dehydrogenase (putative)	sccpdh	4.60E-02	1.290
ILMN_2754551	NM_007876	dipeptidase 1 (renal) (Dpep1), mRNA. (S)	Dpep1	dipeptidase 1 (renal)	DPEP1	4.27E-02	1.291
ILMN_1220791	NM_011074	PFTAIRE protein kinase 1 (Pftk1), mRNA. (S)	Pftk1	PFTAIRE protein kinase 1	cdk14	2.04E-02	1.291
ILMN_2665266	NM_010395	NaN (S)	H2-T10	histocompatibility 2, T region locus 9; hypothetical protein LOC100044191; histocompatibility 2, T region locus 10; hypothetical protein LOC100044190; histocompatibility 2, T region locus 22	LOC100044190	1.99E-02	1.291
ILMN_2504686	NaN	NaN (S)	mt-Atp6	n/a	n/a	5.69E-03	1.292
ILMN_1225681	NM_020256	zinc finger and BTB domain containing 33 (Zbtb33), transcript variant 1, mRNA. (S)	Zbtb33	zinc finger and BTB domain containing 33	ZBTB33	3.29E-02	1.292
ILMN_1251679	NM_133807	leucine rich repeat containing 59 (Lrrc59), mRNA. (S)	Lrrc59	leucine rich repeat containing 59	LRRC59	5.60E-03	1.292
ILMN_3121255	NM_001025250	vascular endothelial growth factor A (Vegfa), transcript variant 1, mRNA. (A)	Vegfa	vascular endothelial growth factor A	VEGFA	1.92E-02	1.293
ILMN_1219647	NM_001081109	lemur tyrosine kinase 2 (Lmtk2), mRNA. (S)	Lmtk2	lemur tyrosine kinase 2	LMTK2	1.88E-03	1.293
ILMN_2529932	XR_034995	PREDICTED: similar to proteasome alpha7/C8 subunit (LOC385905), misc RNA. (S)	LOC385905	proteasome (prosome, macropain) subunit, alpha type 3; predicted gene 5406	Psma3	8.04E-03	1.294
ILMN_2523948	XM_147931	NaN (S)	9130416J18Rik	n/a	n/a	2.06E-03	1.294

ILMN_1226186	NM_007465	baculoviral IAP repeat-containing 2 (Birc2), mRNA. (S)	Birc2	baculoviral IAP repeat-containing 2	birc2	2.95E-02	1.297
ILMN_2621596	XM_886229	PREDICTED: similar to Proteasome activator complex subunit 2 (Proteasome activator 28-beta subunit) (PA28beta) (PA28b) (Activator of multicatalytic protease subunit 2) (11S regulator complex beta subunit) (REG-beta) (LOC621823), mRNA. (S)	LOC621823	protease (prosome, macropain) 28 subunit beta B, pseudogene; similar to Proteasome activator complex subunit 2 (Proteasome activator 28-beta subunit) (PA28beta) (PA28b) (Activator of multicatalytic protease subunit 2) (11S regulator complex beta subunit) (REG-beta); proteasome (prosome, macropain) 28 subunit, beta; predicted gene 7928	Psme2b-ps	3.47E-02	1.297
ILMN_1242571	NM_153805	protein kinase N3 (Pkn3), mRNA. (S)	Pkn3	protein kinase N3	Pkn3	3.22E-02	1.298
ILMN_2820379	NM_028019	ring finger protein 135 (Rnf135), mRNA. (S)	Rnf135	ring finger protein 135	RNF135	1.55E-02	1.299
ILMN_2790241	NM_022331	homocysteine-inducible, endoplasmic reticulum stress-inducible, ubiquitin-like domain member 1 (Herpud1), mRNA. (S)	Herpud1	homocysteine-inducible, endoplasmic reticulum stress-inducible, ubiquitin-like domain member 1	HERPUD1	4.58E-02	1.299
ILMN_2508350	NM_001025572	ankyrin repeat domain 12 (Ankrd12), mRNA. (S)	Ankrd12	ankyrin repeat domain 12; similar to Ankrd12 protein	ANKRD12	2.07E-02	1.301
ILMN_1243813	XR_034503	PREDICTED: similar to Odc1 protein (LOC627245), misc RNA. (S)	LOC627245	predicted gene 6742; ornithine decarboxylase, structural 1; similar to Ornithine decarboxylase (ODC); predicted gene 7993; predicted gene 15645; predicted gene 9115; predicted gene 7278	Gm7278	3.20E-02	1.301
ILMN_1238758	AK050726	NaN (S)	D030011M22Rik	n/a	n/a	2.06E-02	1.301
ILMN_2595612	NM_173011	isocitrate dehydrogenase 2 (NADP+), mitochondrial (Idh2), nuclear gene encoding mitochondrial protein, mRNA. (S)	Idh2	isocitrate dehydrogenase 2 (NADP+), mitochondrial	IDH2	3.34E-02	1.301
ILMN_2735615	NM_020583	interferon-stimulated protein (Isg20), mRNA. (S)	Isg20	interferon-stimulated protein	ISG20	4.29E-02	1.302
ILMN_1231937	NM_027349	NaN (S)	2600011C06Rik	RNA binding motif protein 25	RBM25	2.68E-02	1.302
ILMN_2706269	NM_013560	heat shock protein 1 (Hspb1), mRNA. (S)	Hspb1	heat shock protein 1	Hspb1	2.40E-02	1.302
ILMN_2707063	NM_133352	transmembrane 9 superfamily member 3 (Tm9sf3), mRNA. (S)	Tm9sf3	transmembrane 9 superfamily member 3	TM9SF3	3.55E-02	1.303

ILMN_2744146	XM_110503	NaN (S)	Macf1	n/a	n/a	2.69E-02	1.303
ILMN_2906847	NM_011342	SEC22 vesicle trafficking protein homolog B (S. cerevisiae) (Sec22b), mRNA. (S)	Sec22b	SEC22 vesicle trafficking protein homolog B (S. cerevisiae)	SEC22B	1.01E-02	1.304
ILMN_1221592	NM_025468	SEC11 homolog C (S. cerevisiae) (Sec11c), mRNA. (S)	Sec11c	SEC11 homolog C (S. cerevisiae)	sec11c	2.90E-04	1.305
ILMN_2673668	XR_033663	PREDICTED: similar to MGC69457 protein (LOC624662), misc RNA. (S)	LOC624662	RIKEN cDNA 2900073G15 gene; predicted gene 6517	2900073G15Rik	4.88E-02	1.306
ILMN_2633229	NM_007505	ATP synthase, H+ transporting, mitochondrial F1 complex, alpha subunit, isoform 1 (Atp5a1), nuclear gene encoding mitochondrial protein, mRNA. (S)	Atp5a1	ATP synthase, H+ transporting, mitochondrial F1 complex, alpha subunit, isoform 1	Atp5a1	3.43E-02	1.307
ILMN_1218494	NM_133247	ubiquitin specific peptidase 33 (Usp33), transcript variant 1, mRNA. (S)	Usp33	ubiquitin specific peptidase 33	usp33	1.52E-02	1.307
ILMN_2690061	NM_016690	heterogeneous nuclear ribonucleoprotein D-like (Hnrpdl), mRNA. (S)	Hnrpdl	heterogeneous nuclear ribonucleoprotein D-like	HNRPDL	6.03E-03	1.308
ILMN_2753196	NM_015827	coatomer protein complex, subunit beta 2 (beta prime) (Copb2), mRNA. (S)	Copb2	coatomer protein complex, subunit beta 2 (beta prime)	copb2	2.69E-03	1.309
ILMN_2582791	AK079926	NaN (S)	Cybb	cytochrome b-245, beta polypeptide	CYBB	1.64E-05	1.309
ILMN_1251984	NaN	NaN (S)	C730026J16	n/a	n/a	4.00E-02	1.309
ILMN_2745251	NM_007714	CDC like kinase 4 (Clk4), mRNA. (S)	Clk4	CDC like kinase 4	clk4	3.91E-02	1.310
ILMN_2676662	NM_181409	myotubularin related protein 11 (Mtmr11), mRNA. (S)	Mtmr11	myotubularin related protein 11	MTMR11	3.16E-02	1.311
ILMN_2753021	XM_134599	NaN (S)	Rnf166	n/a	n/a	2.14E-03	1.311
ILMN_2705935	NM_008705	non-metastatic cells 2, protein (NM23B) expressed in (Nme2), transcript variant 1, mRNA. (S)	Nme2	predicted gene 7730; non-metastatic cells 2, protein (NM23B) expressed in; predicted gene 5566; predicted gene 5425; similar to Nucleoside diphosphate kinase B (NDK B) (NDP kinase B) (P18)	NME2	1.59E-02	1.311

ILMN_2639397	NM_026819	dehydrogenase/reductase (SDR family) member 1 (Dhrs1), mRNA. (S)	Dhrs1	dehydrogenase/reductase (SDR family) member 1	dhrs1	4.03E-02	1.311
ILMN_1231168	XM_915849	PREDICTED: H3 histone, family 3B (H3f3b), mRNA. (S)	H3f3b	predicted gene 14383; predicted gene 3835; predicted gene 14384; predicted gene 12950; predicted gene, 670915; H3 histone, family 3A; predicted gene 12657; predicted gene 6132; predicted gene 10257; predicted gene 7227; H3 histone, family 3B; predicted gene 6128; similar to histone; predicted gene 1986; predicted gene 6186; hypothetical protein LOC676337; predicted gene 6421; predicted gene 2198; predicted gene 6817; predicted gene 8095; predicted gene 12271; predicted gene 13529; predicted gene 8029; predicted gene 4938; predicted gene 7100; predicted gene 9014; similar to Histone H3.4 (Embryonic); predicted gene 7179; similar to H3 histone, family 3B; predicted gene 7900; predicted gene 2099; similar to H3 histone, family 3A; predicted gene 6749; predicted gene 6485; predicted gene 4028; predicted gene 7194	Gm3835	4.22E-02	1.312
ILMN_2876575	NM_024432	UBX domain protein 6 (Ubxn6), mRNA. (S)	Ubxn6	UBX domain protein 6	ubxn6	3.06E-03	1.313
ILMN_2684370	XM_132633	NaN (S)	Igk-C	n/a	n/a	2.50E-02	1.313
ILMN_2981871	NM_026211	transmembrane emp24 protein transport domain containing 9 (Tmed9), mRNA. (S)	Tmed9	transmembrane emp24 protein transport domain containing 9	tmed9	6.79E-03	1.313
ILMN_2810405	NM_178440	myosin IG (Myo1g), mRNA. (S)	Myo1g	myosin IG	MYO1G	1.46E-03	1.313
ILMN_1216732	NM_008562	myeloid cell leukemia sequence 1 (Mcl1), mRNA. (S)	Mcl1	similar to myeloid cell leukemia sequence 1; myeloid cell leukemia sequence 1	mcl1	7.64E-03	1.314
ILMN_1258340	AK078415	NaN (S)	Tcf12	transcription factor 12	TCF12	1.05E-02	1.315
ILMN_2647188	XM_001480380	PREDICTED: similar to cytochrome c oxidase, subunit VIIc, transcript variant 1 (LOC100048613), mRNA. (S)	LOC100048613	similar to cytochrome c oxidase, subunit VIIc; predicted gene 3386; cytochrome c oxidase, subunit VIIc	Cox7c	5.99E-03	1.315
ILMN_2419841	NaN	NaN (S)	Silg111	n/a	n/a	2.74E-02	1.315

ILMN_1231873	NM_019770	transmembrane emp24 domain trafficking protein 2 (Tmed2), mRNA. (S)	Tmed2	transmembrane emp24 domain trafficking protein 2; predicted gene 10698; predicted gene 7318	Tmed2	2.61E-02	1.316
ILMN_2682271	NM_021550	C1GALT1-specific chaperone 1 (C1galt1c1), mRNA. (S)	C1galt1c1	C1GALT1-specific chaperone 1	C1galt1c1	1.14E-02	1.317
ILMN_2861787	NM_029478	transmembrane protein 49 (Tmem49), mRNA. (S)	Tmem49	transmembrane protein 49	TMEM49	4.31E-02	1.317
ILMN_2582122	AK089147	NaN (S)	Copg1	n/a	n/a	1.19E-02	1.318
ILMN_2519676	NM_138579	NaN (S)	Triobp	TRIO and F-actin binding protein	Triobp	1.72E-02	1.318
ILMN_1250067	AK028911	NaN (S)	4732469G06Rik	family with sequence similarity 120, member B	Fam120b	4.59E-02	1.318
ILMN_2845272	NM_027117	kelch domain containing 2 (Klhdc2), mRNA. (S)	Klhdc2	kelch domain containing 2	Klhdc2	1.14E-02	1.318
ILMN_1224318	NM_172650	potassium channel tetramerisation domain containing 3 (Kctd3), mRNA. (S)	Kctd3	predicted gene 7553; potassium channel tetramerisation domain containing 3	Gm7553	1.60E-02	1.318
ILMN_1258205	AK047931	NaN (S)	C130022E19Rik	zinc finger protein 826	Zfp826	5.36E-04	1.319
ILMN_1260112	NM_028018	RIKEN cDNA 2400003C14 gene (2400003C14Rik), mRNA. (S)	2400003C14Rik	RIKEN cDNA 2400003C14 gene	2400003C14Rik	3.86E-02	1.319
ILMN_2740432	XM_001479903	PREDICTED: similar to QM protein (LOC100043391), mRNA. (S)	LOC100043391	predicted gene 14460; predicted gene 13891; predicted gene 2387; ribosomal protein 10; predicted gene 7476; predicted gene 4167; predicted gene 5621; predicted gene 3379; similar to QM protein; predicted gene 11450; predicted gene 6564; predicted gene 3405; predicted gene 10041; predicted gene 4892; ribosomal protein L10-like	Gm3379	2.02E-02	1.319
ILMN_2976120	NM_028018	RIKEN cDNA 2400003C14 gene (2400003C14Rik), mRNA. (S)	2400003C14Rik	RIKEN cDNA 2400003C14 gene	2400003C14Rik	1.31E-02	1.320
ILMN_2704769	NM_080446	helicase (DNA) B (Helb), mRNA. (S)	Helb	helicase (DNA) B	helB	2.57E-02	1.321

ILMN_2450019	XM_001476703	PREDICTED: similar to Chain L, Structural Basis Of Antigen Mimicry In A Clinically Relevant Melanoma Antigen System, transcript variant 3 (LOC100047628), mRNA. (S)	LOC100047628	immunoglobulin kappa chain variable 21 (V21)-1; similar to Chain L, Structural Basis Of Antigen Mimicry In A Clinically Relevant Melanoma Antigen System	LOC100047628	3.45E-02	1.321
ILMN_1216190	NM_009025	NaN (S)	Rasa3	RAS p21 protein activator 3	Rasa3	4.35E-02	1.322
ILMN_1228852	XR_035219	PREDICTED: RIKEN cDNA C330011M18 gene (C330011M18Rik), misc RNA. (S)	C330011M18Rik	RIKEN cDNA C330011M18 gene	C330011M18Rik	5.84E-04	1.323
ILMN_2554110	NM_026174	ectonucleoside triphosphate diphosphohydrolase 4 (Entpd4), mRNA. (S)	Entpd4	ectonucleoside triphosphate diphosphohydrolase 4	entpd4	6.01E-03	1.324
ILMN_1244864	NM_026450	zinc finger protein 169 (Zfp169), mRNA. (S)	Zfp169	zinc finger protein 169	Zfp169	7.15E-03	1.324
ILMN_2860645	NM_145545	guanylate binding protein 6 (Gbp6), mRNA. (S)	Gbp6	guanylate binding protein 6	Gbp6	1.60E-02	1.324
ILMN_2869312	NM_134099	F-box protein 4 (Fbxo4), mRNA. (S)	Fbxo4	F-box protein 4	FBXO4	2.21E-02	1.325
ILMN_2678200	NM_180678	glycyl-tRNA synthetase (Gars), mRNA. (S)	Gars	glycyl-tRNA synthetase	Gars	9.26E-03	1.325
ILMN_1239754	XM_148953	NaN (S)	Ppp1r15b	n/a	n/a	2.51E-02	1.325
ILMN_2491831	NaN	NaN (S)	D11Bwg0414e	n/a	n/a	7.84E-03	1.325
ILMN_2668197	NM_011490	staufen (RNA binding protein) homolog 1 (Drosophila) (Stau1), mRNA. (S)	Stau1	staufen (RNA binding protein) homolog 1 (Drosophila)	STAU1	7.87E-03	1.326
ILMN_2837006	NM_001080924	zinc and ring finger 3 (Znrf3), mRNA. (S)	Znrf3	similar to Goliath homolog precursor (Ring finger protein 130) (R-goliath); zinc and ring finger 3	LOC631806	3.36E-02	1.326
ILMN_2734789	NM_024480	SH3 binding domain protein 5 like (Sh3bp5l), mRNA. (S)	Sh3bp5l	SH3 binding domain protein 5 like	SH3BP5L	3.96E-02	1.327

ILMN_2951592	NM_023124	histocompatibility 2, Q region locus 8 (H2-Q8), mRNA. (S)	H2-Q8	histocompatibility 2, Q region locus 1; histocompatibility 2, Q region locus 9; similar to H-2 class I histocompatibility antigen, L-D alpha chain precursor; histocompatibility 2, Q region locus 8; histocompatibility 2, Q region locus 2; similar to MHC class Ib antigen; histocompatibility 2, Q region locus 7; histocompatibility 2, Q region locus 6; hypothetical protein LOC100044307; similar to H-2 class I histocompatibility antigen, Q7 alpha chain precursor (QA-2 antigen); RIKEN cDNA 0610037M15 gene	LOC676708	1.11E-02	1.329
ILMN_1221167	XM_355794	NaN (S)	LOC381793	n/a	n/a	2.79E-02	1.329
ILMN_1228557	NM_010496	inhibitor of DNA binding 2 (Id2), mRNA. (S)	Id2	inhibitor of DNA binding 2	ID2	2.17E-02	1.331
ILMN_1253317	NM_133216	X-prolyl aminopeptidase (aminopeptidase P) 1, soluble (Xpnpep1), mRNA. (S)	Xpnpep1	X-prolyl aminopeptidase (aminopeptidase P) 1, soluble	XPNPEP1	4.85E-02	1.331
ILMN_2517290	NM_008378	imprinted and ancient (Impact), mRNA. (S)	Impact	imprinted and ancient	impact	4.39E-02	1.331
ILMN_1233816	AK004985	NaN (S)	Faah	fatty acid amide hydrolase	Faah	2.72E-02	1.331
ILMN_1241044	AK039536	NaN (S)	A330057G13Rik	zinc finger protein 826	Zfp826	2.80E-02	1.331
ILMN_2764923	NM_024180	ORM1-like 2 (S. cerevisiae) (Ormdl2), mRNA. (S)	Ormdl2	ORM1-like 2 (S. cerevisiae); predicted gene 5553	Gm5553	1.20E-02	1.331
ILMN_2534803	XM_135650	NaN (S)	LOC212970	n/a	n/a	1.49E-02	1.331
ILMN_2668849	NM_027230	protein kinase C binding protein 1 (Prkcbp1), mRNA. (S)	Prkcbp1	zinc finger, MYND-type containing 8	Zmynd8	2.62E-02	1.331
ILMN_2855334	NM_010368	glucuronidase, beta (Gusb), mRNA. (S)	Gusb	glucuronidase, beta	GUSB	1.89E-02	1.331
ILMN_3132223	NM_175454	RIKEN cDNA C630004H02 gene (C630004H02Rik), mRNA. (A)	C630004H02Rik	hypothetical protein LOC100043986; RIKEN cDNA C630004H02 gene	C630004H02Rik	2.86E-02	1.334
ILMN_3139380	NM_133917	MLX interacting protein (Mlxip), transcript variant 2, mRNA. (A)	Mlxip	MLX interacting protein	MLXIP	3.02E-02	1.334
ILMN_1223697	NM_001039150	CD44 antigen (Cd44), transcript variant 2, mRNA. (S)	Cd44	CD44 antigen	CD44	1.14E-02	1.335

ILMN_1238357	NM_213615	RIKEN cDNA A530032D15Rik gene (A530032D15Rik), mRNA. (S)	A530032D15Rik	RIKEN cDNA A530032D15Rik gene; similar to RIKEN cDNA C130026I21 gene	LOC100041746	2.40E-02	1.335
ILMN_2711948	XR_033736	PREDICTED: similar to solute carrier family 7 (cationic amino acid transporter, y+ system), member 5 (LOC100047619), misc RNA. (S)	LOC100047619	similar to solute carrier family 7 (cationic amino acid transporter, y+ system), member 5; similar to Solute carrier family 7 (cationic amino acid transporter, y+ system), member 5; solute carrier family 7 (cationic amino acid transporter, y+ system), member 5	LOC100047619	1.66E-02	1.335
ILMN_1260061	NM_033075	DNA segment, Chr 17, human D6S56E 5 (D17H6S56E-5), mRNA. (S)	D17H6S56E-5	DNA segment, Chr 17, human D6S56E 5	D17H6S56E-5	1.11E-02	1.336
ILMN_1255728	XM_122711	NaN (S)	LOC219049	n/a	n/a	9.49E-03	1.336
ILMN_2532759	XM_141972	NaN (S)	LOC245533	n/a	n/a	4.93E-02	1.338
ILMN_2614706	NM_023799	meningioma expressed antigen 5 (hyaluronidase) (Mgea5), mRNA. (S)	Mgea5	meningioma expressed antigen 5 (hyaluronidase)	mgea5	1.27E-02	1.339
ILMN_2501340	XM_917753	PREDICTED: similar to Ig heavy chain V-I region V35 precursor (LOC640696), mRNA. (S)	LOC640696	similar to Ig heavy chain V-I region V35 precursor	LOC640696	2.61E-02	1.339
ILMN_2730425	NM_009109	ryanodine receptor 1, skeletal muscle (Ryr1), mRNA. (S)	Ryr1	ryanodine receptor 1, skeletal muscle	RYR1	1.26E-04	1.340
ILMN_2432886	XM_001480068	PREDICTED: similar to novel KRAB box and zinc finger, C2H2 type domain containing protein (LOC100043126), mRNA. (S)	LOC100043126	ethanol induced 1; predicted gene 4245; predicted gene 8898; predicted gene 8923; predicted gene 14420; predicted gene 4723	Gm4245	2.03E-02	1.340
ILMN_1216211	NM_025894	proteasome (prosome, macropain) 26S subunit, non-ATPase, 12 (Psmd12), mRNA. (S)	Psmd12	proteasome (prosome, macropain) 26S subunit, non-ATPase, 12	PSMD12	1.16E-03	1.340
ILMN_2503190	NM_016800	vesicle transport through interaction with t-SNAREs 1B homolog (Vti1b), mRNA. (S)	Vti1b	vesicle transport through interaction with t-SNAREs 1B homolog	VTI1B	3.18E-03	1.342
ILMN_2723040	NM_172435	purinergic receptor P2Y, G-protein coupled 10 (P2ry10), mRNA. (S)	P2ry10	purinergic receptor P2Y, G-protein coupled 10	P2RY10	1.56E-03	1.343
ILMN_2727490	NM_021428	dexamethasone-induced transcript (Dexi), mRNA. (S)	Dexi	dexamethasone-induced transcript	DEXI	1.51E-02	1.343
ILMN_2761487	NM_053102	selenoprotein (Sep15), mRNA. (S)	Sep15	selenoprotein	Sep15	2.65E-02	1.343

ILMN_1253412	XM_152907	NaN (S)	5430405G24Rik	G patch domain containing 8	GPATCH8	1.36E-02	1.344
ILMN_2998870	NM_001001983	phosphatidylinositol 4-kinase, catalytic, alpha polypeptide (Pi4ka), mRNA. (S)	Pi4ka	phosphatidylinositol 4-kinase, catalytic, alpha polypeptide	PI4KA	7.28E-03	1.344
ILMN_2955452	NM_011950	mitogen-activated protein kinase 13 (Mapk13), mRNA. (S)	Mapk13	mitogen-activated protein kinase 13	MAPK13	1.99E-02	1.344
ILMN_2871412	NM_011319	seryl-aminoacyl-tRNA synthetase (Sars), mRNA. (S)	Sars	seryl-aminoacyl-tRNA synthetase	SARS	4.03E-02	1.344
ILMN_2989257	NM_133794	glutaminyl-tRNA synthetase (Qars), mRNA. (S)	Qars	glutaminyl-tRNA synthetase	QARS	4.09E-02	1.345
ILMN_1224859	NM_153175	GTPase, IMAP family member 6 (Gimap6), mRNA. (S)	Gimap6	GTPase, IMAP family member 6	GIMAP6	1.64E-02	1.346
ILMN_2588505	NM_134017	methionine adenosyltransferase II, beta (Mat2b), mRNA. (S)	Mat2b	methionine adenosyltransferase II, beta	MAT2B	2.99E-02	1.346
ILMN_2956932	NM_017366	acyl-Coenzyme A dehydrogenase, very long chain (Acadvl), mRNA. (S)	Acadvl	acyl-Coenzyme A dehydrogenase, very long chain	ACADVL	8.45E-03	1.347
ILMN_2652470	XM_485005	NaN (S)	1110059H15Rik	n/a	n/a	3.75E-02	1.347
ILMN_1243345	XM_001476583	PREDICTED: similar to Bcl2-like protein (LOC100046608), mRNA. (S)	LOC100046608	predicted gene 3655; B-cell leukemia/lymphoma 2	BCL2	3.79E-02	1.347
ILMN_2704562	XM_001476703	PREDICTED: similar to Chain L, Structural Basis Of Antigen Mimicry In A Clinically Relevant Melanoma Antigen System, transcript variant 3 (LOC100047628), mRNA. (S)	LOC100047628	immunoglobulin kappa chain variable 21 (V21)-1; similar to Chain L, Structural Basis Of Antigen Mimicry In A Clinically Relevant Melanoma Antigen System	LOC100047628	3.68E-02	1.348
ILMN_2738548	XM_134478	NaN (S)	2010007L18Rik	n/a	n/a	4.83E-02	1.349
ILMN_2639373	NM_025301	NaN (S)	Mrpl17	mitochondrial ribosomal protein L17	MRPL17	4.25E-02	1.349
ILMN_1216930	NM_001081225	family with sequence similarity 178, member A (Fam178a), mRNA. (S)	Fam178a	family with sequence similarity 178, member A	Fam178a	2.39E-02	1.349

ILMN_2633386	XM_001471649	PREDICTED: hypothetical protein LOC100044190 (LOC100044190), mRNA. (S)	LOC100044190	histocompatibility 2, T region locus 9; hypothetical protein LOC100044191; histocompatibility 2, T region locus 10; hypothetical protein LOC100044190; histocompatibility 2, T region locus 22	LOC100044190	1.39E-02	1.349
ILMN_2715195	NM_011504	syntaxin binding protein 3A (Stxbp3a), mRNA. (S)	Stxbp3a	syntaxin binding protein 3A	Stxbp3a	9.93E-03	1.350
ILMN_1224158	XM_147236	NaN (S)	LOC224163	n/a	n/a	3.75E-02	1.350
ILMN_3000008	NM_026776	vacuolar protein sorting 25 (yeast) (Vps25), mRNA. (S)	Vps25	similar to vacuolar protein sorting 25; vacuolar protein sorting 25 (yeast)	vps25	1.95E-02	1.350
ILMN_2641431	NM_172406	trafficking protein, kinesin binding 2 (Trak2), mRNA. (S)	Trak2	trafficking protein, kinesin binding 2	TRAK2	2.52E-03	1.350
ILMN_1227277	NM_028181	NaN (S)	D9Ertd392e	cell cycle progression 1	CCPG1	2.48E-02	1.350
ILMN_2659528	NM_013836	transcription factor 20 (Tcf20), mRNA. (S)	Tcf20	transcription factor 20	TCF20	4.30E-02	1.350
ILMN_1213331	NM_001081378	RIKEN cDNA C330002I19 gene (C330002I19Rik), mRNA. (S)	C330002I19Rik	kinase D-interacting substrate 220	KIDINS220	3.81E-03	1.350
ILMN_2589583	NM_146093	UBX domain protein 1 (Ubxn1), mRNA. (S)	Ubxn1	UBX domain protein 1	Ubxn1	9.91E-03	1.350
ILMN_2522884	NaN	NaN (S)	9930105H17Rik	n/a	n/a	1.03E-02	1.351
ILMN_2617228	NM_011497	aurora kinase A (Aurka), mRNA. (S)	Aurka	aurora kinase A	AURKA	3.12E-02	1.351
ILMN_2726717	NM_133803	dipeptidylpeptidase 3 (Dpp3), mRNA. (S)	Dpp3	dipeptidylpeptidase 3	DPP3	9.44E-04	1.352
ILMN_1212975	NM_133734	WD repeat domain 23 (Wdr23), mRNA. (S)	Wdr23	WD repeat domain 23	dcaf11	4.99E-03	1.353
ILMN_2495446	NaN	NaN (S)	0610037M15Rik	n/a	n/a	3.99E-02	1.353
ILMN_2820877	NM_011285	retinitis pigmentosa GTPase regulator (Rpgr), mRNA. (S)	Rpgr	sushi-repeat-containing protein; retinitis pigmentosa GTPase regulator	SRPX	5.37E-03	1.354
ILMN_1241854	NM_133807	leucine rich repeat containing 59 (Lrrc59), mRNA. (S)	Lrrc59	leucine rich repeat containing 59	LRRC59	2.34E-02	1.354
ILMN_1242881	NM_019953	canopy 2 homolog (zebrafish) (Cnpy2), mRNA. (S)	Cnpy2	canopy 2 homolog (zebrafish)	Cnpy2	3.93E-02	1.355
ILMN_1217128	XM_354717	NaN (S)	Igh-VS107	n/a	n/a	4.88E-02	1.356

ILMN_2689569	NM_028389	transmembrane protein 219 (Tmem219), transcript variant 2, mRNA. (S)	Tmem219	transmembrane protein 219	TMEM219	9.29E-03	1.356
ILMN_1231851	NM_008813	ectonucleotide pyrophosphatase/phosphodiesterase 1 (Enpp1), mRNA. (S)	Enpp1	ectonucleotide pyrophosphatase/phosphodiesterase 1	Enpp1	2.36E-03	1.356
ILMN_2623929	NM_021428	dexamethasone-induced transcript (Dexi), mRNA. (S)	Dexi	dexamethasone-induced transcript	DEXI	3.88E-02	1.357
ILMN_1236411	NM_145940	WD repeat domain, phosphoinositide interacting 1 (Wipi1), mRNA. (S)	Wipi1	WD repeat domain, phosphoinositide interacting 1	WIPI1	1.91E-02	1.357
ILMN_3135107	NM_027379	fatty acyl CoA reductase 1 (Far1), transcript variant 2, mRNA. (A)	Far1	fatty acyl CoA reductase 1	far1	1.01E-02	1.358
ILMN_2941677	NM_007838	dolichyl-di-phosphooligosaccharide-protein glycotransferase (Ddost), mRNA. (S)	Ddost	dolichyl-di-phosphooligosaccharide-protein glycotransferase	DDOST	7.50E-03	1.359
ILMN_2627638	NM_013836	transcription factor 20 (Tcf20), mRNA. (S)	Tcf20	transcription factor 20	TCF20	3.19E-02	1.360
ILMN_2877165	NM_030750	sphingosine-1-phosphate phosphatase 1 (Sgpp1), mRNA. (S)	Sgpp1	sphingosine-1-phosphate phosphatase 1	Sgpp1	4.95E-02	1.360
ILMN_1215807	NM_028608	GLI pathogenesis-related 1 (glioma) (Glipr1), mRNA. (S)	Glipr1	GLI pathogenesis-related 1 (glioma)	GLIPR1	3.70E-02	1.360
ILMN_1260381	NM_173369	cylindromatosis (turban tumor syndrome) (Cyld), mRNA. (S)	Cyld	cylindromatosis (turban tumor syndrome)	CYLD	7.15E-05	1.361
ILMN_1250981	AK052794	NaN (S)	D730006F06Rik	tocopherol (alpha) transfer protein-like	ttpal	2.46E-02	1.361
ILMN_1253985	AK039311	NaN (S)	A330021D07Rik	splicing factor, arginine/serine-rich 12	SFRS12	3.72E-02	1.362
ILMN_2660477	NM_023472	ankyrin repeat, family A (RFXANK-like), 2 (Ankra2), mRNA. (S)	Ankra2	ankyrin repeat, family A (RFXANK-like), 2	ANKRA2	2.50E-02	1.362
ILMN_1227540	NM_024250	PHD finger protein 10 (Phf10), mRNA. (S)	Phf10	PHD finger protein 10	Phf10	1.88E-02	1.362
ILMN_2593554	NM_018738	interferon gamma induced GTPase (Igtp), mRNA. (S)	Igtp	interferon gamma induced GTPase	Igtp	2.73E-02	1.362
ILMN_2666007	NM_022325	cathepsin Z (Ctsz), mRNA. (S)	Ctsz	cathepsin Z	CTSZ	1.58E-02	1.363

ILMN_2654554	NM_008013	fibrinogen-like protein 2 (Fgl2), mRNA. (S)	Fgl2	fibrinogen-like protein 2	FGL2	1.28E-02	1.364
ILMN_1260536	NM_0010811055	trafficking protein particle complex 10 (Trappc10), mRNA. (I)	Trappc10	trafficking protein particle complex 10	TRAPPC10	1.36E-02	1.364
ILMN_2526836	XM_204369	NaN (S)	LOC278105	n/a	n/a	2.19E-02	1.365
ILMN_1244338	XM_127388	NaN (S)	2300006M17Rik	n/a	n/a	1.79E-02	1.365
ILMN_2487787	NM_0010811055	trafficking protein particle complex 10 (Trappc10), mRNA. (S)	Trappc10	trafficking protein particle complex 10	TRAPPC10	7.97E-03	1.365
ILMN_2749327	NM_172574	PQ loop repeat containing (Pqlc3), mRNA. (S)	Pqlc3	PQ loop repeat containing	PQLC3	1.03E-03	1.366
ILMN_1236822	NM_024192	CUE domain containing 2 (Cuedc2), mRNA. (S)	Cuedc2	CUE domain containing 2	CUEDC2	3.44E-02	1.366
ILMN_1258238	AK034574	NaN (S)	Zfp288	zinc finger and BTB domain containing 20	ZBTB20	3.76E-02	1.367
ILMN_1241658	AK038954	NaN (S)	A230078H02Rik	n/a	n/a	6.50E-03	1.367
ILMN_3153207	NM_0010177959	lysosomal-associated membrane protein 2 (Lamp2), transcript variant 1, mRNA. (A)	Lamp2	lysosomal-associated membrane protein 2	lamp2	4.45E-04	1.367
ILMN_2467429	NaN	NaN (S)	D6Mit97	n/a	n/a	3.46E-02	1.369
ILMN_2723826	NM_011405	NaN (S)	Slc7a7	solute carrier family 7 (cationic amino acid transporter, y+ system), member 7	SLC7A7	6.69E-03	1.369
ILMN_1231503	NM_024188	3-oxoacid CoA transferase 1 (Oxct1), mRNA. (S)	Oxct1	3-oxoacid CoA transferase 1	oxct1	2.77E-02	1.371
ILMN_1232251	NM_009408	topoisomerase (DNA) I (Top1), mRNA. (S)	Top1	topoisomerase (DNA) I	TOP1	1.14E-02	1.371
ILMN_2637714	NM_009025	RAS p21 protein activator 3 (Rasa3), mRNA. (S)	Rasa3	RAS p21 protein activator 3	Rasa3	4.05E-02	1.371
ILMN_2744587	NM_019686	calcium and integrin binding family member 2 (Cib2), mRNA. (S)	Cib2	calcium and integrin binding family member 2	cib2	4.87E-02	1.371
ILMN_2882820	NM_028184	oral cancer overexpressed 1 (Oraov1), mRNA. (S)	Oraov1	oral cancer overexpressed 1	ORAOV1	2.55E-02	1.372
ILMN_2771951	NM_172930	NaN (S)	6430550H21Rik	family with sequence similarity 70, member A	fam70a	9.85E-03	1.373

ILMN_2719794	NM_145511	NaN (S)	BC003331	similar to odorant response abnormal 4; cDNA sequence BC003331	BC003331	3.74E-03	1.373
ILMN_2437470	NaN	NaN (S)	2810423A18Rik	n/a	n/a	1.65E-02	1.373
ILMN_2621909	NM_027078	NaN (S)	1700023M03Rik	RIKEN cDNA 1200009F10 gene	ikbip	1.33E-02	1.374
ILMN_1255561	NM_022310	heat shock protein 5 (Hspa5), mRNA. (S)	Hspa5	heat shock protein 5	HSPA5	4.89E-02	1.374
ILMN_2775386	NM_008929	DnaJ (Hsp40) homolog, subfamily C, member 3 (Dnajc3), mRNA. (S)	Dnajc3	DnaJ (Hsp40) homolog, subfamily C, member 3	DNAJC3	5.37E-03	1.374
ILMN_2421580	NM_173395	family with sequence similarity 132, member B (Fam132b), mRNA. (S)	Fam132b	family with sequence similarity 132, member B	FAM132B	4.18E-03	1.374
ILMN_2957543	NM_001033978	RIKEN cDNA A130038H09 gene (A130038H09Rik), mRNA. (S)	A130038H09Rik	histocompatibility 2, class II antigen E beta2	H2-Eb2	4.98E-02	1.374
ILMN_2795520	NM_001001892	histocompatibility 2, K1, K region (H2-K1), mRNA. (S)	H2-K1	histocompatibility 2, K1, K region; similar to H-2K(d) antigen	H2-K1	2.14E-02	1.374
ILMN_2904339	NM_026626	EF-hand calcium binding domain 2 (Efcab2), mRNA. (S)	Efcab2	EF-hand calcium binding domain 2	Efcab2	9.12E-03	1.374
ILMN_2441724	NaN	NaN (S)	9430014F16Rik	n/a	n/a	1.19E-02	1.375
ILMN_1233076	NM_008925	protein kinase C substrate 80K-H (Prkcsh), mRNA. (S)	Prkcsh	protein kinase C substrate 80K-H	prkcsh	2.08E-02	1.375
ILMN_2720930	NM_030749	endoplasmic reticulum chaperone SIL1 homolog (S. cerevisiae) (Sil1), mRNA. (S)	Sil1	endoplasmic reticulum chaperone SIL1 homolog (S. cerevisiae)	SIL1	7.53E-03	1.375
ILMN_1249517	NM_028990	transmembrane protein 168 (Tmem168), mRNA. (S)	Tmem168	transmembrane protein 168	TMEM168	2.45E-02	1.376
ILMN_1213883	NM_021518	RAB2A, member RAS oncogene family (Rab2a), mRNA. (S)	Rab2a	predicted gene 5865; RAB2A, member RAS oncogene family	RAB2A	3.08E-02	1.377
ILMN_2935796	NM_026395	RER1 retention in endoplasmic reticulum 1 homolog (S. cerevisiae) (Rer1), mRNA. (S)	Rer1	RER1 retention in endoplasmic reticulum 1 homolog (S. cerevisiae)	RER1	1.50E-02	1.377
ILMN_2722469	NM_026101	hect domain and RLD 4 (Herc4), mRNA. (S)	Herc4	hect domain and RLD 4	herc4	2.70E-02	1.378

ILMN_2874554	NM_175438	aldehyde dehydrogenase 4 family, member A1 (Aldh4a1), nuclear gene encoding mitochondrial protein, mRNA. (S)	Aldh4a1	aldehyde dehydrogenase 4 family, member A1	ALDH4A1	4.42E-02	1.378
ILMN_2787085	NM_029701	signal peptidase complex subunit 3 homolog (S. cerevisiae) (Spcs3), mRNA. (S)	Spcs3	signal peptidase complex subunit 3 homolog (S. cerevisiae)	SPCS3	3.29E-03	1.378
ILMN_1219939	NM_145491	ras homolog gene family, member Q (Rhoq), mRNA. (S)	Rhoq	ras homolog gene family, member Q	rhoq	4.89E-02	1.378
ILMN_1253539	XR_031980	PREDICTED: similar to Ddx5 protein (LOC432554), misc RNA. (S)	LOC432554	DEAD (Asp-Glu-Ala-Asp) box polypeptide 5; predicted gene 12183	Gm12183	4.72E-03	1.379
ILMN_2673959	NM_010281	gamma-glutamyl hydrolase (Ggh), mRNA. (S)	Ggh	gamma-glutamyl hydrolase	GGH	4.51E-02	1.379
ILMN_2583439	AK087122	NaN (S)	E030028L09Rik	LIM domain containing preferred translocation partner in lipoma	lpp	3.74E-02	1.380
ILMN_1259359	AK089228	NaN (S)	Dock2	dedicator of cyto-kinesis 2	DOCK2	3.78E-02	1.380
ILMN_1258687	XM_909747	PREDICTED: zinc finger protein 292, transcript variant 4 (Zfp292), mRNA. (S)	Zfp292	zinc finger protein 292	Zfp292	1.01E-02	1.380
ILMN_2857580	NM_181406	arginyl-tRNA synthetase 2, mitochondrial (Rars2), nuclear gene encoding mitochondrial protein, mRNA. (S)	Rars2	arginyl-tRNA synthetase 2, mitochondrial	rars2	3.63E-02	1.380
ILMN_2418143	NaN	NaN (S)	5330401F18Rik	n/a	n/a	5.00E-02	1.381
ILMN_2467044	XM_973700	PREDICTED: similar to Ig heavy chain V region 441 precursor (LOC674072), mRNA. (S)	LOC674072	similar to Ig heavy chain V region 441 precursor	LOC674072	1.85E-02	1.381
ILMN_2473718	NM_001033439	leucine-rich repeats and calponin homology (CH) domain containing 1 (Lrch1), mRNA. (S)	Lrch1	leucine-rich repeats and calponin homology (CH) domain containing 1	Lrch1	1.71E-02	1.381

ILMN_1237074	NM_026071	solute carrier family 25 (mitochondrial thiamine pyrophosphate carrier), member 19 (Slc25a19), nuclear gene encoding mitochondrial protein, mRNA. (S)	Slc25a19	solute carrier family 25 (mitochondrial thiamine pyrophosphate carrier), member 19	SLC25A19	1.60E-02	1.381
ILMN_3001598	NM_011202	protein tyrosine phosphatase, non-receptor type 11 (Ptpn11), mRNA. (S)	Ptpn11	protein tyrosine phosphatase, non-receptor type 11	PTPN11	4.79E-02	1.382
ILMN_1213448	XM_976371	PREDICTED: similar to melanoma antigen (LOC669658), mRNA. (S)	LOC669658	n/a	n/a	2.35E-03	1.382
ILMN_2777471	NM_010398	histocompatibility 2, T region locus 23 (H2-T23), mRNA. (S)	H2-T23	histocompatibility 2, T region locus 23; similar to RT1 class Ib, locus H2-Q-like, grc region	LOC677644	2.55E-02	1.382
ILMN_2742215	NM_134111	ELL associated factor 2 (Eaf2), transcript variant 1, mRNA. (S)	Eaf2	ELL associated factor 2	EAF2	4.01E-02	1.383
ILMN_2729171	NM_172699	forkhead box J3 (Foxj3), mRNA. (S)	Foxj3	forkhead box J3	Foxj3	4.22E-02	1.384
ILMN_2810539	NM_001081015	predicted gene, EG630499 (EG630499), mRNA. (S)	EG630499	predicted gene 7035	Gm7035	4.37E-02	1.386
ILMN_2771182	NM_023124	NaN (S)	H2-Q8	histocompatibility 2, Q region locus 1; histocompatibility 2, Q region locus 9; similar to H-2 class I histocompatibility antigen, L-D alpha chain precursor; histocompatibility 2, Q region locus 8; histocompatibility 2, Q region locus 2; similar to MHC class Ib antigen; histocompatibility 2, Q region locus 7; histocompatibility 2, Q region locus 6; hypothetical protein LOC100044307; similar to H-2 class I histocompatibility antigen, Q7 alpha chain precursor (QA-2 antigen); RIKEN cDNA 0610037M15 gene	LOC676708	2.83E-02	1.386
ILMN_1224483	NM_172435	purinergic receptor P2Y, G-protein coupled 10 (P2ry10), mRNA. (S)	P2ry10	purinergic receptor P2Y, G-protein coupled 10	P2RY10	8.68E-03	1.386
ILMN_1259927	AK054000	NaN (S)	E230011J22Rik	similar to phosphofurin acidic cluster sorting protein 2; phosphofurin acidic cluster sorting protein 2	LOC100048879	2.99E-02	1.387
ILMN_2644469	XR_005113	PREDICTED: similar to Ribosomal protein L8 (LOC675377), misc RNA. (S)	LOC675377	ribosomal protein L8; similar to 60S ribosomal protein L8	rpl8	3.99E-04	1.387
ILMN_2588130	NM_001081015	predicted gene, EG630499 (EG630499), mRNA. (S)	EG630499	predicted gene 7035	Gm7035	2.98E-02	1.388

ILMN_2803627	NM_025460	transmembrane protein 126A (Tmem126a), mRNA. (S)	Tmem126a	transmembrane protein 126A	Tmem126a	3.77E-02	1.388
ILMN_2694579	NM_153055	SEC63-like (S. cerevisiae) (Sec63), mRNA. (S)	Sec63	SEC63-like (S. cerevisiae)	SEC63	1.37E-02	1.389
ILMN_2798803	NM_126165	vacuolar protein sorting 4a (yeast) (Vps4a), mRNA. (S)	Vps4a	vacuolar protein sorting 4a (yeast)	vps4a	4.60E-02	1.389
ILMN_2621752	NM_012057	interferon regulatory factor 5 (Irf5), mRNA. (S)	Irf5	interferon regulatory factor 5	IRF5	5.53E-03	1.389
ILMN_1255679	XM_357613	NaN (S)	LOC384382	n/a	n/a	1.98E-02	1.389
ILMN_2712731	NM_024250	PHD finger protein 10 (Phf10), mRNA. (S)	Phf10	PHD finger protein 10	Phf10	6.23E-03	1.390
ILMN_2714534	NM_008927	mitogen-activated protein kinase kinase 1 (Map2k1), mRNA. (S)	Map2k1	mitogen-activated protein kinase kinase 1	MAP2K1	3.05E-02	1.391
ILMN_2707137	NM_172303	PHD finger protein 17 (Phf17), mRNA. (S)	Phf17	PHD finger protein 17	phf17	1.17E-02	1.391
ILMN_1251419	NM_008581	NaN (S)	Mela	melanoma antigen	melA	1.57E-02	1.393
ILMN_2771991	NM_013864	NaN (S)	Ndrg2	N-myc downstream regulated gene 2	Ndrg2	3.53E-03	1.393
ILMN_2431007	XM_127049	NaN (S)	Alkbh	n/a	n/a	3.15E-02	1.393
ILMN_2712507	XM_130011	NaN (S)	2810455F06Rik	n/a	n/a	2.09E-02	1.393
ILMN_2806676	NM_007383	acyl-Coenzyme A dehydrogenase, short chain (Acads), nuclear gene encoding mitochondrial protein, mRNA. (S)	Acads	acyl-Coenzyme A dehydrogenase, short chain	Acads	1.19E-02	1.393
ILMN_2765032	NM_008433	potassium intermediate/small conductance calcium-activated channel, subfamily N, member 4 (Kcnn4), mRNA. (S)	Kcnn4	potassium intermediate/small conductance calcium-activated channel, subfamily N, member 4	KCNN4	2.78E-04	1.393
ILMN_2684402	XM_485210	NaN (S)	1810022K09Rik	n/a	n/a	2.39E-02	1.394
ILMN_2852904	NM_024475	ubiquitin-like domain containing CTD phosphatase 1 (Ublcp1), mRNA. (S)	Ublcp1	ubiquitin-like domain containing CTD phosphatase 1; predicted gene 12663; similar to ubiquitin-like domain containing CTD phosphatase 1	Gm12663	2.21E-02	1.394

ILMN_2777474	NM_010398	histocompatibility 2, T region locus 23 (H2-T23), mRNA. (S)	H2-T23	histocompatibility 2, T region locus 23; similar to RT1 class Ib, locus H2-Q-like, grc region	LOC677644	3.21E-02	1.394
ILMN_2617865	NM_172529	N-acetylglucosamine-1-phosphotransferase, gamma subunit (Gnptg), mRNA. (S)	Gnptg	similar to N-acetylglucosamine-1-phosphotransferase, gamma subunit; N-acetylglucosamine-1-phosphotransferase, gamma subunit	LOC100047632	3.92E-02	1.394
ILMN_1249598	NM_173369	cylindromatosis (turban tumor syndrome) (Cyld), mRNA. (S)	Cyld	cylindromatosis (turban tumor syndrome)	CYLD	1.94E-04	1.397
ILMN_1254927	NM_010741	lymphocyte antigen 6 complex, locus C1 (Ly6c1), mRNA. (S)	Ly6c1	lymphocyte antigen 6 complex, locus C2; lymphocyte antigen 6 complex, locus C1	Ly6c1	1.69E-02	1.398
ILMN_2498813	NM_001024205	nuclear fragile X mental retardation protein interacting protein 2 (Nufip2), mRNA. (S)	Nufip2	nuclear fragile X mental retardation protein interacting protein 2	NUFIP2	1.56E-02	1.399
ILMN_2736430	NM_024233	REX2, RNA exonuclease 2 homolog (S. cerevisiae) (Rexo2), mRNA. (S)	Rexo2	REX2, RNA exonuclease 2 homolog (S. cerevisiae)	Rexo2	4.14E-02	1.400
ILMN_2496534	NM_027357	proteasome (prosome, macropain) 26S subunit, non-ATPase, 1 (Psmd1), mRNA. (S)	Psmd1	proteasome (prosome, macropain) 26S subunit, non-ATPase, 1	PSMD1	1.39E-02	1.400
ILMN_1212836	NM_011210	protein tyrosine phosphatase, receptor type, C (Ptprc), mRNA. (S)	Ptprc	protein tyrosine phosphatase, receptor type, C	Ptprc	2.55E-02	1.401
ILMN_1232692	XM_911155	PREDICTED: similar to putative translation initiation factor A121/Sui1 (LOC383712), mRNA. (S)	LOC383712	predicted gene 13637	Gm13637	2.33E-03	1.401
ILMN_2772920	NM_134099	NaN (S)	Fbxo4	F-box protein 4	FBXO4	1.16E-02	1.402
ILMN_2493464	NM_010806	myeloid/lymphoid or mixed-lineage leukemia (trithorax homolog, Drosophila); translocated to, 4 (Mllt4), mRNA. (S)	Mllt4	myeloid/lymphoid or mixed-lineage leukemia (trithorax homolog, Drosophila); translocated to, 4	mllt4	4.77E-02	1.402
ILMN_2669146	NM_194341	AP1 gamma subunit binding protein 1 (Ap1gbp1), transcript variant 2, mRNA. (S)	Ap1gbp1	AP1 gamma subunit binding protein 1	SYNRG	2.73E-02	1.404
ILMN_1233446	AK048183	NaN (S)	C130039D01Rik	cylindromatosis (turban tumor syndrome)	CYLD	3.98E-02	1.404

ILMN_2722902	NM_011787	autocrine motility factor receptor (Amfr), mRNA. (S)	Amfr	autocrine motility factor receptor	Amfr	4.85E-03	1.404
ILMN_1252817	NM_133807	leucine rich repeat containing 59 (Lrrc59), mRNA. (S)	Lrrc59	leucine rich repeat containing 59	LRRC59	9.81E-03	1.404
ILMN_2696518	XM_001001365	PREDICTED: similar to Ig heavy chain V region 441 precursor (LOC668418), mRNA. (S)	LOC668418	n/a	n/a	3.70E-02	1.404
ILMN_2714822	NM_153055	SEC63-like (S. cerevisiae) (Sec63), mRNA. (S)	Sec63	SEC63-like (S. cerevisiae)	SEC63	1.88E-02	1.404
ILMN_2425415	NM_054093	ubiquitin protein ligase E3B (Ube3b), mRNA. (S)	Ube3b	ubiquitin protein ligase E3B	UBE3B	1.15E-02	1.405
ILMN_2803920	NM_010742	lymphocyte antigen 6 complex, locus D (Ly6d), mRNA. (S)	Ly6d	lymphocyte antigen 6 complex, locus D	LY6D	3.26E-02	1.405
ILMN_2891688	NM_001033140	RIKEN cDNA 0610010E21 gene (0610010E21Rik), mRNA. (S)	0610010E21Rik	succinate dehydrogenase complex assembly factor 1	SDHAF1	2.19E-02	1.405
ILMN_2603781	NM_080837	NaN (S)	D17Wsu104e	DNA segment, Chr 17, Wayne State University 104, expressed	D17Wsu104e	4.23E-02	1.406
ILMN_2428807	NaN	NaN (S)	A030011A13Rik	n/a	n/a	3.52E-02	1.406
ILMN_2773113	NM_007599	capping protein (actin filament), gelsolin-like (Capg), transcript variant 1, mRNA. (S)	Capg	capping protein (actin filament), gelsolin-like	capG	1.18E-02	1.406
ILMN_2703159	NM_009269	serine palmitoyltransferase, long chain base subunit 1 (Sptlc1), mRNA. (S)	Sptlc1	serine palmitoyltransferase, long chain base subunit 1	sptlc1	2.34E-02	1.406
ILMN_2940440	NM_008060	alpha glucosidase 2 alpha neutral subunit (Ganab), mRNA. (S)	Ganab	alpha glucosidase 2 alpha neutral subunit	GANAB	1.81E-03	1.407
ILMN_2752074	NM_011151	protein phosphatase 1B, magnesium dependent, beta isoform (Ppm1b), mRNA. XM_925494 XM_925495 XM_925496 (S)	Ppm1b	similar to serine/threonine phosphatase; protein phosphatase 1B, magnesium dependent, beta isoform	PPM1B	3.03E-02	1.408
ILMN_1245362	XM_111637	NaN (S)	LOC193690	n/a	n/a	3.34E-02	1.408
ILMN_1243964	XM_001478436	PREDICTED: F-box and leucine-rich repeat protein 17 (Fbxl17), mRNA. (S)	Fbxl17	F-box and leucine-rich repeat protein 17	FBXL17	2.73E-02	1.409

ILMN_3151345	NM_173363	eukaryotic translation initiation factor 5 (Eif5), transcript variant 1, mRNA. (A)	Eif5	similar to Eukaryotic translation initiation factor 5; eukaryotic translation initiation factor 5	LOC100047658	2.20E-02	1.410
ILMN_2742861	NM_001033335	serine (or cysteine) peptidase inhibitor, clade A, member 3F (Serpina3f), mRNA. (S)	Serpina3f	serine (or cysteine) peptidase inhibitor, clade A, member 3F	Serpina3f	2.46E-02	1.410
ILMN_2791578	NM_146066	G1 to S phase transition 1 (Gspt1), mRNA. (S)	Gspt1	G1 to S phase transition 1	GSPT1	1.63E-03	1.410
ILMN_1225386	XM_898537	PREDICTED: RIKEN cDNA 2300009A05 gene, transcript variant 3 (2300009A05Rik), mRNA. (S)	2300009A05Rik	RIKEN cDNA 2300009A05 gene	2300009A05Rik	2.91E-02	1.410
ILMN_2721761	NM_008667	Ngfi-A binding protein 1 (Nab1), mRNA. (S)	Nab1	Ngfi-A binding protein 1	NAB1	1.13E-03	1.411
ILMN_2790188	NM_026604	family with sequence similarity 135, member A (Fam135a), mRNA. (S)	Fam135a	family with sequence similarity 135, member A	FAM135A	3.39E-04	1.412
ILMN_1259092	NM_177149	NaN (S)	D930030O05Rik	n/a	n/a	7.62E-04	1.413
ILMN_1257184	NM_026252	NaN (S)	Cpeb4	cytoplasmic polyadenylation element binding protein 4	CPEB4	2.32E-02	1.413
ILMN_2829636	NM_013760	DnaJ (Hsp40) homolog, subfamily B, member 9 (Dnajb9), mRNA. (S)	Dnajb9	predicted gene 6568; DnaJ (Hsp40) homolog, subfamily B, member 9	dnajb9	4.03E-03	1.414
ILMN_2611256	NM_021485	ribosomal protein S6 kinase, polypeptide 2 (Rps6kb2), mRNA. (S)	Rps6kb2	ribosomal protein S6 kinase, polypeptide 2	RPS6KB2	1.31E-02	1.414
ILMN_2710705	NM_011018	sequestosome 1 (Sqstm1), mRNA. (S)	Sqstm1	sequestosome 1	sqstm1	4.83E-02	1.414
ILMN_2771360	NM_008193	NaN (S)	Guk1	guanylate kinase 1	GUK1	3.42E-03	1.414
ILMN_2757599	NM_025982	NaN (S)	Sas	tetraspanin 31	Tspan31	4.63E-03	1.415
ILMN_2801404	NM_007713	CDC-like kinase 3 (Clk3), mRNA. (S)	Clk3	CDC-like kinase 3	clk3	6.86E-03	1.415
ILMN_1251729	NM_008686	nuclear factor, erythroid derived 2,-like 1 (Nfe2l1), mRNA. (S)	Nfe2l1	nuclear factor, erythroid derived 2,-like 1	NFE2L1	3.85E-03	1.416
ILMN_2624544	NM_080446	helicase (DNA) B (Helb), mRNA. (S)	Helb	helicase (DNA) B	helB	4.09E-02	1.416

ILMN_2833441	NM_172275	TRAF type zinc finger domain containing 1 (Trafd1), mRNA. (S)	Trafd1	TRAF type zinc finger domain containing 1	trafd1	6.56E-03	1.416
ILMN_2635888	NM_024203	family with sequence similarity 120, member B (Fam120b), mRNA. (S)	Fam120b	family with sequence similarity 120, member B	Fam120b	2.33E-02	1.417
ILMN_2766296	XM_992400	PREDICTED: similar to Ig kappa chain V-V region MOPC 173 (LOC434031), mRNA. (S)	LOC434031	predicted gene 5573	Gm5573	3.42E-02	1.417
ILMN_2616309	NM_145925	pituitary tumor-transforming 1 interacting protein (Pttg1ip), mRNA. (S)	Pttg1ip	pituitary tumor-transforming 1 interacting protein	Pttg1ip	3.96E-02	1.418
ILMN_2591381	NM_011151	protein phosphatase 1B, magnesium dependent, beta isoform (Ppm1b), mRNA. XM_925494 XM_925495 XM_925496 (S)	Ppm1b	similar to serine/threonine phosphatase; protein phosphatase 1B, magnesium dependent, beta isoform	PPM1B	3.92E-02	1.418
ILMN_2694578	NM_153055	NaN (S)	Sec63	SEC63-like (S. cerevisiae)	SEC63	1.05E-02	1.419
ILMN_2643423	XM_001478799	PREDICTED: similar to transmembrane emp24 domain-containing protein 10 (LOC100042773), mRNA. (S)	LOC100042773	transmembrane emp24-like trafficking protein 10 (yeast); predicted gene 4024	tmed10	1.93E-02	1.419
ILMN_1258366	AK013891	NaN (S)	3010002L02Rik	n/a	n/a	1.90E-02	1.419
ILMN_2657628	NM_011343	SEC61, gamma subunit (Sec61g), mRNA. (S)	Sec61g	predicted gene 11575; predicted gene 10177; predicted gene 4184; SEC61, gamma subunit; similar to Sec61-complex gamma-subunit	Gm16412	3.37E-02	1.420
ILMN_1248468	XR_031426	PREDICTED: hypothetical LOC632684 (LOC632684), misc RNA. (S)	LOC632684	La ribonucleoprotein domain family, member 4; predicted gene 14373; predicted gene 8177	Larp4	4.60E-02	1.421
ILMN_2566822	AK038836	NaN (S)	A230067E15Rik	Sec24 related gene family, member A (S. cerevisiae)	Sec24a	2.49E-03	1.421
ILMN_2466637	XM_620754	PREDICTED: unc-51-like kinase 3 (C. elegans), transcript variant 1 (Ulk3), mRNA. (S)	Ulk3	unc-51-like kinase 3 (C. elegans)	ulk3	4.16E-02	1.421
ILMN_2667889	NM_017406	cAMP responsive element binding protein-like 1 (Crebl1), mRNA. (S)	Crebl1	activating transcription factor 6 beta	ATF6B	4.85E-02	1.422

ILMN_2466655	NM_028130	NaN (S)	2610020C11Rik	zinc finger protein 157	Zfp157	4.89E-02	1.422
ILMN_2691832	NM_177186	RIKEN cDNA A530082C11 gene (A530082C11Rik), mRNA. (S)	A530082C11Rik	RIKEN cDNA A530082C11 gene	SLC35E2	1.12E-02	1.423
ILMN_1236157	NM_001081956	splicing factor, arginine/serine-rich 17b (Sfrs17b), mRNA. (S)	Sfrs17b	splicing factor, arginine/serine-rich 17b	Sfrs17b	2.96E-02	1.423
ILMN_1257759	NM_144812	trinucleotide repeat containing 6b (Tnrc6b), transcript variant 1, mRNA. (S)	Tnrc6b	trinucleotide repeat containing 6b	TNRC6B	3.84E-02	1.424
ILMN_1254987	NM_175341	muscleblind-like 2 (Mbnl2), transcript variant 1, mRNA. (S)	Mbnl2	muscleblind-like 2	mbnl2	1.69E-02	1.425
ILMN_1240567	NaN	NaN (S)	1700060J05Rik	n/a	n/a	1.84E-02	1.425
ILMN_2704979	NM_023232	diablo homolog (Drosophila) (Diablo), nuclear gene encoding mitochondrial protein, mRNA. (S)	Diablo	diablo homolog (Drosophila)	Diablo	3.46E-02	1.426
ILMN_2640971	NM_008776	platelet-activating factor acetylhydrolase, isoform 1b, alpha1 subunit (Pafah1b3), mRNA. (S)	Pafah1b3	platelet-activating factor acetylhydrolase, isoform 1b, subunit 3	PAFAH1B3	1.40E-02	1.426
ILMN_2918018	NM_011188	proteasome (prosome, macropain) 26S subunit, ATPase 2 (Psmc2), mRNA. (S)	Psmc2	proteasome (prosome, macropain) 26S subunit, ATPase 2	PSMC2	1.63E-02	1.426
ILMN_2634603	NM_019909	MHC (A.CA/J(H-2K-f) class I antigen (LOC56628), mRNA. (S)	LOC56628	MHC (A.CA/J(H-2K-f) class I antigen	LOC56628	4.53E-04	1.427
ILMN_2716935	NM_007807	cytochrome b-245, beta polypeptide (Cybb), mRNA. (S)	Cybb	cytochrome b-245, beta polypeptide	CYBB	1.62E-02	1.427
ILMN_1249366	XM_001476583	PREDICTED: similar to Bcl2-like protein (LOC100046608), mRNA. (S)	LOC100046608	predicted gene 3655; B-cell leukemia/lymphoma 2	BCL2	1.02E-02	1.428
ILMN_2762925	XM_001476332	PREDICTED: similar to Cacna2d2 protein (LOC100046259), mRNA. (S)	LOC100046259	calcium channel, voltage-dependent, alpha 2/delta subunit 2; similar to Cacna2d2 protein	LOC100046259	3.14E-02	1.428
ILMN_1216435	XM_359086	NaN (S)	LOC386135	n/a	n/a	2.59E-03	1.428
ILMN_2455501	NM_009190	vacuolar protein sorting 4b (yeast) (Vps4b), mRNA. (S)	Vps4b	similar to vacuolar protein sorting 4b; vacuolar protein sorting 4b (yeast)	VPS4B	1.18E-02	1.428

ILMN_2441326	NM_011981	NaN (S)	Zfp260	zinc finger protein 260	Zfp260	2.92E-03	1.430
ILMN_2842877	NM_008853	praja1, RING-H2 motif containing (Pja1), mRNA. (S)	Pja1	praja1, RING-H2 motif containing	PJA1	4.82E-02	1.430
ILMN_2593134	NM_173742	ribonuclease, RNase K (Rnasek), mRNA. (S)	Rnasek	ribonuclease, RNase K	Rnasek	3.30E-03	1.430
ILMN_2744045	XM_001477596	PREDICTED: similar to proteasome alpha7/C8 subunit (LOC100047184), mRNA. (S)	LOC100047184	predicted gene 13835	Gm13835	1.71E-03	1.431
ILMN_2749604	XM_126906	NaN (S)	Scfd1	n/a	n/a	7.77E-03	1.431
ILMN_2762956	NM_025668	signal peptidase complex subunit 2 homolog (S. cerevisiae) (Spcs2), mRNA. (S)	Spcs2	signal peptidase complex subunit 2 homolog (S. cerevisiae); predicted gene 14045	Gm14045	3.43E-03	1.432
ILMN_2429800	NM_009395	tumor necrosis factor, alpha-induced protein 1 (endothelial) (Tnfaip1), mRNA. (S)	Tnfaip1	tumor necrosis factor, alpha-induced protein 1 (endothelial)	TNFAIP1	1.89E-02	1.433
ILMN_2673077	NM_001005847	aspartylglucosaminidase (Aga), mRNA. (S)	Aga	aspartylglucosaminidase	aga	2.89E-03	1.433
ILMN_2483811	NaN	NaN (S)	2210408F11Rik	n/a	n/a	3.49E-02	1.433
ILMN_2833985	NM_030259	Rab interacting lysosomal protein-like 2 (Rilpl2), mRNA. (S)	Rilpl2	Rab interacting lysosomal protein-like 2	Rilpl2	3.07E-02	1.435
ILMN_2701779	NM_145988	NaN (S)	1700108L22Rik	n/a	n/a	1.40E-02	1.435
ILMN_1220975	NM_008979	protein tyrosine phosphatase, non-receptor type 22 (lymphoid) (Ptpn22), mRNA. (S)	Ptpn22	protein tyrosine phosphatase, non-receptor type 22 (lymphoid)	PTPN22	3.97E-02	1.436
ILMN_1251912	NM_024468	tripartite motif-containing 39 (Trim39), mRNA. (S)	Trim39	tripartite motif-containing 39	TRIM39	1.95E-02	1.436
ILMN_2821501	NM_022985	zinc finger, AN1-type domain 6 (Zfand6), mRNA. (S)	Zfand6	zinc finger, AN1-type domain 6	zfand6	1.90E-02	1.437
ILMN_2769393	NM_145124	mindbomb homolog 2 (Drosophila) (Mib2), mRNA. (S)	Mib2	mindbomb homolog 2 (Drosophila)	mib2	1.48E-02	1.438
ILMN_2721503	NM_013792	alpha-N-acetylglucosaminidase (Sanfilippo disease IIIB) (Naglu), mRNA. (S)	Naglu	alpha-N-acetylglucosaminidase (Sanfilippo disease IIIB)	Naglu	5.31E-03	1.439

ILMN_2660851	NM_009740	B-cell leukemia/lymphoma 10 (Bcl10), mRNA. (S)	Bcl10	B-cell leukemia/lymphoma 10; predicted gene 6141	BCL10	1.82E-04	1.440
ILMN_2828687	NM_001003913	methionine-tRNA synthetase (Mars), mRNA. (S)	Mars	methionine-tRNA synthetase	mars	4.88E-02	1.440
ILMN_2589318	NM_010127	POU domain, class 6, transcription factor 1 (Pou6f1), mRNA. (S)	Pou6f1	POU domain, class 6, transcription factor 1	POU6F1	1.63E-04	1.441
ILMN_2773491	XM_983803	PREDICTED: RIKEN cDNA 1810006K21 gene (1810006K21Rik), mRNA. (S)	1810006K21Rik	RIKEN cDNA 1810006K21 gene	1810006K21Rik	2.99E-02	1.441
ILMN_2815107	NM_019650	golgi SNAP receptor complex member 2 (Gosr2), mRNA. (S)	Gosr2	golgi SNAP receptor complex member 2	GOSR2	1.90E-02	1.443
ILMN_1224016	XM_194592	NaN (S)	5830417I10Rik	n/a	n/a	3.37E-02	1.443
ILMN_2476341	NaN	NaN (S)	2310051F07Rik	n/a	n/a	4.08E-02	1.443
ILMN_2493249	NaN	NaN (S)	IGHV1S14_K00707$X00161_Ig_heavy_variable_1S14_164	n/a	n/a	2.20E-02	1.443
ILMN_1212637	NM_027400	lectin, mannose-binding, 1 (Lman1), mRNA. (S)	Lman1	lectin, mannose-binding, 1	Lman1	1.29E-02	1.445
ILMN_2643067	NM_145355	ring finger protein 185 (Rnf185), mRNA. (S)	Rnf185	ring finger protein 185	RNF185	4.41E-02	1.446
ILMN_2773012	NM_153198	NaN (S)	Hbp1	high mobility group box transcription factor 1	HBP1	3.38E-02	1.447
ILMN_2901284	NM_013758	adducin 3 (gamma) (Add3), mRNA. (S)	Add3	adducin 3 (gamma)	ADD3	1.49E-02	1.447
ILMN_2588882	NM_177388	solute carrier family 41, member 2 (Slc41a2), mRNA. (S)	Slc41a2	solute carrier family 41, member 2	SLC41A2	3.93E-02	1.448
ILMN_1216085	AK046455	NaN (S)	B230387C07Rik	ankyrin repeat domain 12; similar to Ankrd12 protein	ANKRD12	4.32E-02	1.448
ILMN_3160517	NM_001017429	cytochrome c oxidase, subunit XVII assembly protein homolog (yeast) (Cox17), mRNA. (S)	Cox17	cytochrome c oxidase, subunit XVII assembly protein homolog (yeast)	cox17	3.28E-03	1.449

ILMN_2675337	NM_207648	histocompatibility 2, Q region locus 6 (H2-Q6), mRNA. (S)	H2-Q6	histocompatibility 2, Q region locus 1; histocompatibility 2, Q region locus 9; similar to H-2 class I histocompatibility antigen, L-D alpha chain precursor; histocompatibility 2, Q region locus 8; histocompatibility 2, Q region locus 2; similar to MHC class Ib antigen; histocompatibility 2, Q region locus 7; histocompatibility 2, Q region locus 6; hypothetical protein LOC100044307; similar to H-2 class I histocompatibility antigen, Q7 alpha chain precursor (QA-2 antigen); RIKEN cDNA 0610037M15 gene	LOC676708	8.49E-03	1.449
ILMN_1218668	NM_007594	calumenin (Calu), transcript variant 1, mRNA. (S)	Calu	calumenin	CALU	1.19E-02	1.450
ILMN_1239946	XM_001473491	PREDICTED: similar to vacuolar H(+)-ATPase (LOC100039636), mRNA. (S)	LOC100039636	ATPase, H+ transporting, lysosomal V0 subunit C, pseudogene 2; ATPase, H+ transporting, lysosomal V0 subunit C	ATP6V0C	1.33E-03	1.450
ILMN_2781798	NM_001033297	gene model 561, (NCBI) (Gm561), mRNA. (S)	Gm561	predicted gene 561	Gm561	3.26E-02	1.450
ILMN_2790636	NM_009120	SAR1 gene homolog A (S. cerevisiae) (Sar1a), mRNA. (S)	Sar1a	SAR1 gene homolog A (S. cerevisiae)	sar1a	3.63E-02	1.450
ILMN_2963634	NM_138680	LUC7-like 2 (S. cerevisiae) (Luc7l2), mRNA. (S)	Luc7l2	predicted gene 11889; LUC7-like 2 (S. cerevisiae)	Gm11889	7.90E-03	1.451
ILMN_2967576	NM_008193	guanylate kinase 1 (Guk1), mRNA. (S)	Guk1	guanylate kinase 1	GUK1	2.45E-03	1.451
ILMN_2683620	NM_016703	NaN (S)	Preb	prolactin regulatory element binding	PREB	3.95E-03	1.452
ILMN_2473620	NM_019765	NaN (S)	Rsn	CAP-GLY domain containing linker protein 1	CLIP1	3.89E-02	1.453
ILMN_2665131	NM_013785	inositol hexaphosphate kinase 1 (Ihpk1), mRNA. (S)	Ihpk1	inositol hexaphosphate kinase 1	IP6K1	1.48E-02	1.453
ILMN_2835683	NM_001081032	predicted gene, EG667977 (EG667977), mRNA. (S)	EG667977	predicted gene 8909	Gm8909	6.83E-03	1.453
ILMN_2668977	NM_001083891	RIKEN cDNA 1500032D16 gene (1500032D16Rik), transcript variant 2, mRNA. (S)	1500032D16Rik	NADH dehydrogenase (ubiquinone) flavoprotein 3	NDUFV3	1.57E-03	1.454
ILMN_2698115	NM_181470	LTV1 homolog (S. cerevisiae) (Ltv1), mRNA. (S)	Ltv1	LTV1 homolog (S. cerevisiae)	LTV1	2.78E-02	1.454
ILMN_2984828	NM_008529	lymphocyte antigen 6 complex, locus E (Ly6e), mRNA. (S)	Ly6e	lymphocyte antigen 6 complex, locus E	Ly6e	5.85E-04	1.455

ILMN_2591791	NM_023311	Yip1 domain family, member 5 (Yipf5), mRNA. (S)	Yipf5	Yip1 domain family, member 5; predicted gene 5738	yipf5	6.65E-04	1.455
ILMN_2881054	NM_001018013	izumo sperm-egg fusion 1 (Izumo1), mRNA. (S)	Izumo1	izumo sperm-egg fusion 1	Izumo1	2.69E-02	1.455
ILMN_1240274	NM_172661	RIKEN cDNA 5830434P21 gene (5830434P21Rik), mRNA. (S)	5830434P21Rik	HLA-B associated transcript 2-like	Bat2l	1.48E-02	1.456
ILMN_2805945	NM_030685	DNA segment, Chr 3, University of California at Los Angeles 1 (D3Ucla1), mRNA. (S)	D3Ucla1	stress-associated endoplasmic reticulum protein 1	serp1	1.66E-03	1.456
ILMN_2612079	NM_133626	ribosome binding protein 1 (Rrbp1), transcript variant 2, mRNA. (S)	Rrbp1	ribosome binding protein 1	RRBP1	3.13E-02	1.457
ILMN_2773422	NM_001078649	transmembrane protein 134 (Tmem134), transcript variant 1, mRNA. (S)	Tmem134	transmembrane protein 134	Tmem134	9.43E-03	1.458
ILMN_2585683	AK078639	NaN (S)	Nek7	NIMA (never in mitosis gene a)-related expressed kinase 7	NEK7	8.31E-03	1.459
ILMN_2623040	NM_177662	cathepsin O (Ctso), mRNA. (S)	Ctso	cathepsin O	Ctso	5.66E-04	1.462
ILMN_1215711	XM_357852	NaN (S)	LOC384766	n/a	n/a	1.89E-02	1.462
ILMN_2935898	NM_019760	serine incorporator 1 (Serinc1), mRNA. (S)	Serinc1	serine incorporator 1	SERINC1	3.25E-02	1.463
ILMN_1233607	XM_911184	PREDICTED: similar to Ig kappa chain V-II region 17S29.1 (LOC434026), mRNA. (S)	LOC434026	predicted gene 5572	Gm5572	1.29E-02	1.463
ILMN_2644061	NM_177325	TSR1, 20S rRNA accumulation, homolog (yeast) (Tsr1), mRNA. (S)	Tsr1	similar to CG7338-PA; TSR1, 20S rRNA accumulation, homolog (yeast)	tsr1	1.20E-03	1.463
ILMN_2761120	NM_009011	NaN (S)	Rad23b	RAD23b homolog (S. cerevisiae)	RAD23B	3.19E-02	1.463
ILMN_1234720	NM_175219	RIKEN cDNA C130026I21 gene (C130026I21Rik), transcript variant 1, mRNA. (S)	C130026I21Rik	RIKEN cDNA C130026I21 gene; similar to SP140 nuclear body protein family member; similar to RIKEN cDNA C130026I21 gene	C130026I21Rik	3.50E-02	1.464
ILMN_2706514	XM_001476583	PREDICTED: similar to Bcl2-like protein (LOC100046608), mRNA. (S)	LOC100046608	predicted gene 3655; B-cell leukemia/lymphoma 2	BCL2	1.32E-03	1.467
ILMN_2735253	NM_145541	RAS-related protein-1a (Rap1a), mRNA. (S)	Rap1a	predicted gene 9392; similar to Raichu404X; RAS-related protein-1a	LOC100046373	1.31E-02	1.468

ILMN_2607408	NM_021567	poly(rC) binding protein 4 (Pcbp4), mRNA. (S)	Pcbp4	poly(rC) binding protein 4	PCBP4	4.77E-02	1.468
ILMN_1220793	NM_025717	RNA binding motif protein 4B (Rbm4b), mRNA. (S)	Rbm4b	RNA binding motif protein 4B	rbm4b	4.23E-02	1.469
ILMN_2759598	NM_026871	histidine triad nucleotide binding protein 2 (Hint2), mRNA. (S)	Hint2	histidine triad nucleotide binding protein 2	HINT2	9.55E-03	1.469
ILMN_2854811	NM_007786	casein kappa (Csn3), mRNA. (S)	Csn3	casein kappa	CSN3	2.74E-03	1.469
ILMN_2661125	NM_019791	melanoma antigen, family D, 1 (Maged1), mRNA. (S)	Maged1	melanoma antigen, family D, 1	MAGED1	4.64E-03	1.469
ILMN_1244439	XM_979562	PREDICTED: ethanolamine kinase 1 (Etnk1), mRNA. (S)	Etnk1	ethanolamine kinase 1	etnk1	2.76E-02	1.469
ILMN_2671747	NM_027044	prefoldin 5 (Pfdn5), mRNA. (S)	Pfdn5	prefoldin 5	PFDN5	1.09E-02	1.470
ILMN_2795791	NM_028053	transmembrane protein 38B (Tmem38b), mRNA. (S)	Tmem38b	transmembrane protein 38B	tmem38b	9.79E-03	1.470
ILMN_1214368	XR_035177	PREDICTED: cDNA sequence AB041803 (AB041803), misc RNA. (S)	AB041803	cDNA sequence AB041803	AB041803	4.04E-02	1.470
ILMN_2920708	NM_145931	zinc finger CCCH type containing 7 A (Zc3h7a), mRNA. (S)	Zc3h7a	zinc finger CCCH type containing 7 A	ZC3H7A	4.53E-02	1.472
ILMN_2680799	NM_134077	NaN (S)	1700009P03Rik	RNA binding motif protein 26	RBM26	1.04E-02	1.472
ILMN_1242617	NM_175271	NaN (S)	Gpr23	lysophosphatidic acid receptor 4	lpar4	3.15E-02	1.472
ILMN_1256488	NM_026168	ERGIC and golgi 2 (Ergic2), transcript variant 1, mRNA. (S)	Ergic2	ERGIC and golgi 2	ergic2	2.48E-02	1.473
ILMN_1218128	AK010232	NaN (S)	2310079P03Rik	TatD DNase domain containing 1	TATDN1	4.64E-02	1.474
ILMN_2971171	NM_145141	Fc receptor-like A (Fcrla), mRNA. (S)	Fcrla	Fc receptor-like A	FCRLA	1.54E-02	1.475
ILMN_2732757	XM_126365	NaN (S)	9830002I17Rik	n/a	n/a	2.03E-02	1.476
ILMN_1222500	XM_001477552	PREDICTED: similar to immunoglobulin kappa-chain (LOC100047162), mRNA. (S)	LOC100047162	immunoglobulin kappa chain variable 19 (V19)-15; similar to immunoglobulin kappa-chain	LOC100047162	3.49E-02	1.477
ILMN_1239088	NM_175451	cytoskeleton-associated protein 4 (Ckap4), mRNA. (S)	Ckap4	cytoskeleton-associated protein 4	Ckap4	1.16E-02	1.477

ILMN_1227814	AK043819	NaN (S)	Srr	serine racemase	srr	3.11E-02	1.479
ILMN_2809239	NM_026519	transmembrane protein 85 (Tmem85), mRNA. (S)	Tmem85	transmembrane protein 85	TMEM85	6.28E-03	1.479
ILMN_2839569	NM_022325	cathepsin Z (Ctsz), mRNA. (S)	Ctsz	cathepsin Z	CTSZ	1.85E-03	1.481
ILMN_1224942	NM_028230	serine hydroxymethyltransferase 2 (mitochondrial) (Shmt2), nuclear gene encoding mitochondrial protein, mRNA. (S)	Shmt2	serine hydroxymethyltransferase 2 (mitochondrial)	SHMT2	3.94E-02	1.481
ILMN_1229173	NM_008207	histocompatibility 2, T region locus 24 (H2-T24), mRNA. (S)	H2-T24	similar to histocompatibility 2, T region locus 24; histocompatibility 2, T region locus 24	H2-T24	3.95E-02	1.482
ILMN_2699222	XM_972773	PREDICTED: similar to Ig kappa chain V-V region MPC11 precursor (LOC669053), mRNA. (S)	LOC669053	n/a	n/a	4.40E-02	1.484
ILMN_2636463	NM_013685	transcription factor 4 (Tcf4), transcript variant 1, mRNA. (S)	Tcf4	transcription factor 4	TCF4	4.65E-02	1.485
ILMN_1224992	NM_027045	granule cell antiserum positive 14 (Gcap14), transcript variant 1, mRNA. (S)	Gcap14	granule cell antiserum positive 14	Gcap14	7.76E-03	1.485
ILMN_2870549	NM_008929	DnaJ (Hsp40) homolog, subfamily C, member 3 (Dnajc3), mRNA. (S)	Dnajc3	DnaJ (Hsp40) homolog, subfamily C, member 3	DNAJC3	1.21E-02	1.485
ILMN_1237036	NM_010923	NaN (S)	Nnat	neuronatin	Nnat	3.10E-02	1.485
ILMN_2555484	XM_903904	PREDICTED: enhancer of zeste homolog 1 (Drosophila), transcript variant 4 (Ezh1), mRNA. (S)	Ezh1	enhancer of zeste homolog 1 (Drosophila)	EZH1	4.97E-02	1.486
ILMN_1232991	NM_025952	RIKEN cDNA 2610529C04 gene (2610529C04Rik), mRNA. (S)	2610529C04Rik	magnesium transporter 1	MAGT1	1.01E-04	1.487
ILMN_2748837	NM_080633	aconitase 2, mitochondrial (Aco2), nuclear gene encoding mitochondrial protein, mRNA. (S)	Aco2	aconitase 2, mitochondrial	ACO2	1.59E-02	1.487
ILMN_1226851	NM_013454	ATP-binding cassette, sub-family A (ABC1), member 1 (Abca1), mRNA. (S)	Abca1	ATP-binding cassette, sub-family A (ABC1), member 1	ABCA1	2.12E-02	1.487
ILMN_2683621	NM_016703	prolactin regulatory element binding (Preb), mRNA. (S)	Preb	prolactin regulatory element binding	PREB	1.48E-03	1.489

ILMN_2504433	NM_011631	heat shock protein 90, beta (Grp94), member 1 (Hsp90b1), mRNA. (S)	Hsp90b1	heat shock protein 90, beta (Grp94), member 1	Hsp90b1	1.08E-02	1.490
ILMN_1230423	NM_177364	SH3 and PX domains 2B (Sh3pxd2b), mRNA. (S)	Sh3pxd2b	SH3 and PX domains 2B	SH3PXD2B	3.18E-02	1.490
ILMN_2694257	NM_025455	coiled coil domain containing 28B (Ccdc28b), mRNA. (S)	Ccdc28b	coiled coil domain containing 28B	Ccdc28b	1.02E-02	1.491
ILMN_1225994	NM_009500	vav 2 oncogene (Vav2), mRNA. (S)	Vav2	vav 2 oncogene	VAV2	2.28E-02	1.491
ILMN_2870443	NM_010236	folylpolyglutamyl synthetase (Fpgs), mRNA. (S)	Fpgs	folylpolyglutamyl synthetase	FPGS	1.87E-03	1.492
ILMN_2510152	NaN	NaN (S)	5830411O09Rik	n/a	n/a	3.80E-03	1.492
ILMN_2853744	NM_026396	brix domain containing 2 (Bxdc2), mRNA. (S)	Bxdc2	brix domain containing 2	BRIX1	4.23E-03	1.492
ILMN_1215212	NM_007483	ras homolog gene family, member B (Rhob), mRNA. (S)	Rhob	ras homolog gene family, member B	RHOB	2.46E-02	1.492
ILMN_1236759	NM_025455	coiled coil domain containing 28B (Ccdc28b), mRNA. (S)	Ccdc28b	coiled coil domain containing 28B	Ccdc28b	4.40E-02	1.493
ILMN_2665535	NM_145584	spondin 1, (f-spondin) extracellular matrix protein (Spon1), mRNA. (S)	Spon1	spondin 1, (f-spondin) extracellular matrix protein	SPON1	2.41E-05	1.494
ILMN_1243030	NM_022985	zinc finger, AN1-type domain 6 (Zfand6), mRNA. (S)	Zfand6	zinc finger, AN1-type domain 6	zfand6	1.36E-02	1.495
ILMN_2675464	NM_198010	ankyrin repeat domain 17 (Ankrd17), transcript variant 2, mRNA. (S)	Ankrd17	ankyrin repeat domain 17	ANKRD17	1.84E-02	1.495
ILMN_2733576	NM_133236	glucocorticoid induced transcript 1 (Glcci1), transcript variant 1, mRNA. (S)	Glcci1	similar to glucocorticoid induced transcript 1; predicted gene 5815; glucocorticoid induced transcript 1	LOC100046012	1.11E-02	1.498
ILMN_2479530	NM_009572	zinc fingers and homeoboxes 1 (Zhx1), transcript variant 1, mRNA. (S)	Zhx1	zinc fingers and homeoboxes 1	ZHX1	2.78E-02	1.498
ILMN_1244794	NM_175255	Sec24 related gene family, member A (S. cerevisiae) (Sec24a), mRNA. (S)	Sec24a	Sec24 related gene family, member A (S. cerevisiae)	Sec24a	9.54E-04	1.499
ILMN_2700292	NM_010376	histocompatibility 13 (H13), mRNA. (S)	H13	histocompatibility 13	H13	7.92E-03	1.500

ILMN_1259905	NM_001093753	splicing factor, arginine/serine-rich 11 (Sfrs11), transcript variant 1, mRNA. (S)	Sfrs11	splicing factor, arginine/serine-rich 11	SFRS11	3.02E-02	1.500
ILMN_1230777	NM_212470	RIKEN cDNA 0610007C21 gene (0610007C21Rik), transcript variant 2, mRNA. (S)	0610007C21Rik	RIKEN cDNA 0610007C21 gene	0610007C21Rik	2.64E-02	1.500
ILMN_2416628	NM_028195	cytohesin 4 (Cyth4), mRNA. (S)	Cyth4	cytohesin 4	CYTH4	4.59E-02	1.500
ILMN_1249821	NM_026009	coiled-coil domain containing 47 (Ccdc47), mRNA. (S)	Ccdc47	coiled-coil domain containing 47	Ccdc47	4.73E-03	1.502
ILMN_2988480	NM_021301	solute carrier family 15 (H+/peptide transporter), member 2 (Slc15a2), mRNA. (S)	Slc15a2	solute carrier family 15 (H+/peptide transporter), member 2	Slc15a2	4.19E-02	1.503
ILMN_3048509	NM_001013774	expressed sequence AW146299 (AW146299), mRNA. (I)	AW146299	karyopherin alpha 7 (importin alpha 8)	KPNA7	3.19E-02	1.504
ILMN_2689056	NM_027353	CD2 antigen (cytoplasmic tail) binding protein 2 (Cd2bp2), mRNA. (S)	Cd2bp2	CD2 antigen (cytoplasmic tail) binding protein 2	CD2BP2	9.17E-03	1.506
ILMN_2705131	NM_139063	muted (Muted), mRNA. (S)	Muted	muted	MUTED	4.77E-02	1.506
ILMN_1237208	NM_010684	lysosomal-associated membrane protein 1 (Lamp1), mRNA. (S)	Lamp1	lysosomal-associated membrane protein 1	lamp1	4.15E-04	1.507
ILMN_1258732	NM_021351	crystallin, beta A4 (Cryba4), mRNA. (S)	Cryba4	crystallin, beta A4	CRYBA4	2.89E-03	1.507
ILMN_2683128	NM_009908	cytidine monophospho-N-acetylneuraminic acid synthetase (Cmas), mRNA. (S)	Cmas	cytidine monophospho-N-acetylneuraminic acid synthetase	CMAS	7.63E-03	1.507
ILMN_2791326	NM_009143	stromal cell derived factor 2 (Sdf2), mRNA. (S)	Sdf2	stromal cell derived factor 2	SDF2	1.29E-03	1.507
ILMN_2700354	NM_177192	DENN/MADD domain containing 5B (Dennd5b), mRNA. (S)	Dennd5b	DENN/MADD domain containing 5B	DENND5B	2.21E-02	1.507
ILMN_2466965	XM_484191	PREDICTED: similar to Ig heavy chain V-II region SESS precursor (LOC432709), mRNA. (S)	LOC432709	predicted gene 5440	Gm5440	4.17E-02	1.508
ILMN_2758878	NM_026432	transmembrane protein 66 (Tmem66), mRNA. (S)	Tmem66	transmembrane protein 66	TMEM66	7.81E-03	1.508
ILMN_1252288	NaN	NaN (S)	2010007H06Rik	n/a	n/a	1.06E-02	1.508

ILMN_2897476	NM_025969	RIKEN cDNA 1700034H14 gene (1700034H14Rik), mRNA. (S)	1700034H14Rik	RIKEN cDNA 1700034H14 gene	1700034H14Rik	5.38E-04	1.510
ILMN_2514967	XM_129836	NaN (S)	Phf3	n/a	n/a	1.41E-02	1.511
ILMN_3008277	NM_030087	RIKEN cDNA 1500032D16 gene (1500032D16Rik), mRNA. (S)	1500032D16Rik	NADH dehydrogenase (ubiquinone) flavoprotein 3	NDUFV3	7.42E-03	1.511
ILMN_1235782	NM_001045520	clathrin interactor 1 (Clint1), mRNA. (S)	Clint1	clathrin interactor 1	clint1	9.67E-03	1.511
ILMN_2944011	NM_001034908	similar to H-2 class I histocompatibility antigen, L-D alpha chain precursor (LOC547343), mRNA. (S)	LOC547343	similar to H-2 class I histocompatibility antigen, L-D alpha chain precursor	LOC547343	4.26E-03	1.512
ILMN_2955671	NM_175121	solute carrier family 38, member 2 (Slc38a2), mRNA. (S)	Slc38a2	solute carrier family 38, member 2	slc38a2	6.74E-05	1.513
ILMN_2514225	NM_138680	NaN (S)	Luc7l2	predicted gene 11889; LUC7-like 2 (S. cerevisiae)	Gm11889	2.71E-02	1.513
ILMN_2784019	NM_010283	glycoprotein galactosyltransferase alpha 1, 3 (Ggta1), mRNA. (S)	Ggta1	glycoprotein galactosyltransferase alpha 1, 3	GGTA1	2.65E-03	1.514
ILMN_1220230	NM_153198	NaN (S)	Hbp1	high mobility group box transcription factor 1	HBP1	1.45E-02	1.515
ILMN_2684205	XR_002338	PREDICTED: RIKEN cDNA 4930431B09 gene (4930431B09Rik), misc RNA. (S)	4930431B09Rik	family with sequence similarity 46, member C	FAM46C	2.84E-02	1.516
ILMN_2939294	NM_025562	fission 1 (mitochondrial outer membrane) homolog (yeast) (Fis1), mRNA. (S)	Fis1	fission 1 (mitochondrial outer membrane) homolog (yeast)	FIS1	3.96E-02	1.516
ILMN_2750515	NM_010234	FBJ osteosarcoma oncogene (Fos), mRNA. (S)	Fos	FBJ osteosarcoma oncogene	FOS	1.07E-02	1.516
ILMN_1226208	AK080527	NaN (S)	Pipk5k3	phosphoinositide kinase, FYVE finger containing	Pikfyve	1.59E-02	1.516
ILMN_2506012	NM_021897	transformation related protein 53 inducible nuclear protein 1 (Trp53inp1), mRNA. (S)	Trp53inp1	transformation related protein 53 inducible nuclear protein 1	Trp53inp1	1.79E-02	1.518
ILMN_1237089	NM_008060	alpha glucosidase 2 alpha neutral subunit (Ganab), mRNA. (S)	Ganab	alpha glucosidase 2 alpha neutral subunit	GANAB	2.43E-03	1.518
ILMN_1234318	NM_024432	UBX domain protein 6 (Ubxn6), mRNA. (S)	Ubxn6	UBX domain protein 6	ubxn6	3.14E-03	1.520

ILMN_2779746	NM_012058	signal recognition particle 9 (Srp9), mRNA. (S)	Srp9	signal recognition particle 9	srp9	2.42E-03	1.520
ILMN_1234842	AK051269	NaN (S)	Msi2h	Musashi homolog 2 (Drosophila)	MSI2	2.04E-02	1.520
ILMN_2949266	NM_019642	ribophorin II (Rpn2), mRNA. (S)	Rpn2	ribophorin II	Rpn2	6.57E-04	1.521
ILMN_2449466	XR_033090	PREDICTED: similar to protein kinase Myak-S (LOC100046825), misc RNA. (S)	LOC100046825	homeodomain interacting protein kinase 1; similar to protein kinase Myak-S	hipk1	2.16E-03	1.523
ILMN_2642197	NM_026693	gamma-aminobutyric acid (GABA-A) receptor-associated protein-like 2 (Gabarapl2), mRNA. (S)	Gabarapl2	gamma-aminobutyric acid (GABA) A receptor-associated protein-like 2; predicted gene 3724	GABARAPL2	4.22E-02	1.524
ILMN_1253449	NaN	NaN (S)	IGHV8S6_U23021_Ig_heavy_variable_8S6_61	n/a	n/a	3.55E-02	1.524
ILMN_1246611	NM_016898	CD164 antigen (Cd164), mRNA. (S)	Cd164	CD164 antigen	CD164	2.07E-03	1.525
ILMN_2588002	XM_207856	NaN (I)	LOC280487	n/a	n/a	4.24E-02	1.526
ILMN_2495068	AK087931	NaN (S)	scl000854.1_75	predicted gene 2785	Mndal	1.11E-02	1.528
ILMN_1216117	AK029530	NaN (S)	4921514E18Rik	large tumor suppressor	lats1	3.59E-02	1.528
ILMN_2758029	NM_011178	proteinase 3 (Prtn3), mRNA. (S)	Prtn3	proteinase 3	PRTN3	6.78E-03	1.529
ILMN_2777059	NM_144942	NaN (S)	Csad	cysteine sulfinic acid decarboxylase	CSAD	2.48E-03	1.530
ILMN_2928050	NM_023126	RAB8A, member RAS oncogene family (Rab8a), mRNA. (S)	Rab8a	RAB8A, member RAS oncogene family	RAB8A	2.03E-02	1.531
ILMN_2666406	NM_028447	proline-rich coiled-coil 1 (Prrc1), mRNA. (S)	Prrc1	proline-rich coiled-coil 1	PRRC1	2.03E-02	1.531
ILMN_1238847	NM_007648	CD3 antigen, epsilon polypeptide (Cd3e), mRNA. (S)	Cd3e	CD3 antigen, epsilon polypeptide	CD3E	1.21E-03	1.531
ILMN_2741402	NM_010656	sarcospan (Sspn), mRNA. (S)	Sspn	sarcospan	sspN	1.71E-03	1.532

ILMN_2511768	NM_183106	tetratricopeptide repeat domain 17 (Ttc17), mRNA. (S)	Ttc17	tetratricopeptide repeat domain 17	ttc17	1.85E-03	1.533
ILMN_2712018	NM_009736	Bcl2-associated athanogene 1 (Bag1), mRNA. (S)	Bag1	BCL2-associated athanogene 1	bag1	4.13E-04	1.534
ILMN_1251748	NM_139200	cytohesin 1 interacting protein (Cytip), mRNA. (S)	Cytip	cytohesin 1 interacting protein	CYTIP	4.52E-02	1.536
ILMN_2670561	NM_009735	beta-2 microglobulin (B2m), mRNA. (S)	B2m	beta-2 microglobulin	B2M	6.21E-03	1.536
ILMN_2678355	NM_178114	adhesion molecule with Ig like domain 2 (Amigo2), mRNA. (S)	Amigo2	adhesion molecule with Ig like domain 2	AMIGO2	9.64E-05	1.538
ILMN_2518457	NM_008363	interleukin-1 receptor-associated kinase 1 (Irak1), mRNA. (S)	Irak1	interleukin-1 receptor-associated kinase 1	Irak1	5.21E-03	1.538
ILMN_3106053	NM_008202	solute carrier family 39 (zinc transporter), member 7 (Slc39a7), transcript variant 1, mRNA. (A)	Slc39a7	solute carrier family 39 (zinc transporter), member 7	Slc39a7	3.66E-02	1.540
ILMN_2736875	NM_007952	protein disulfide isomerase associated 3 (Pdia3), mRNA. (S)	Pdia3	protein disulfide isomerase associated 3	PDIA3	2.21E-02	1.540
ILMN_3162887	NM_001013817	Sp140 nuclear body protein (Sp140), mRNA. (I)	Sp140	predicted gene 2389; Sp140 nuclear body protein	Gm2389	4.93E-03	1.542
ILMN_1222584	NM_001093753	splicing factor, arginine/serine-rich 11 (Sfrs11), transcript variant 1, mRNA. (S)	Sfrs11	splicing factor, arginine/serine-rich 11	SFRS11	2.72E-02	1.544
ILMN_2488997	NM_145512	NaN (S)	2010005O13Rik	SFT2 domain containing 2	SFT2D2	1.32E-02	1.546
ILMN_2986899	NM_134079	adenosine kinase (Adk), mRNA. (S)	Adk	adenosine kinase	adk	1.56E-03	1.546
ILMN_1260582	XM_203701	NaN (I)	LOC280487	n/a	n/a	5.81E-03	1.548
ILMN_2844848	NM_026423	RIKEN cDNA 2410018C20 gene (2410018C20Rik), mRNA. (S)	2410018C20Rik	methylthioribose-1-phosphate isomerase homolog (S. cerevisiae)	mri1	1.00E-02	1.549
ILMN_3094013	NM_175341	muscleblind-like 2 (Mbnl2), transcript variant 1, mRNA. (A)	Mbnl2	muscleblind-like 2	mbnl2	1.04E-02	1.549
ILMN_2641569	NM_019425	glucosamine-phosphate N-acetyltransferase 1 (Gnpnat1), mRNA. (S)	Gnpnat1	glucosamine-phosphate N-acetyltransferase 1; predicted gene 7623	Gnpnat1	3.70E-03	1.551
ILMN_2632276	NM_145937	sulfatase modifying factor 1 (Sumf1), mRNA. (S)	Sumf1	sulfatase modifying factor 1	sumf1	8.48E-03	1.551

ILMN_2588094	XM_978742	PREDICTED: RIKEN cDNA 2810409K11 gene (2810409K11Rik), mRNA. (S)	2810409K11Rik	RIKEN cDNA 2810409K11 gene	Zfp773	1.73E-02	1.551
ILMN_2659006	NM_173006	paraoxonase 3 (Pon3), mRNA. (S)	Pon3	paraoxonase 3	PON3	2.64E-05	1.552
ILMN_2729386	NM_008200	histocompatibility 2, D region locus 4 (H2-D4), mRNA. (S)	H2-D4	histocompatibility 2, D region locus 4	H2-D4	1.47E-02	1.552
ILMN_2881296	NM_026432	transmembrane protein 66 (Tmem66), mRNA. (S)	Tmem66	transmembrane protein 66	TMEM66	2.54E-02	1.553
ILMN_3002943	NM_026775	transmembrane emp24-like trafficking protein 10 (yeast) (Tmed10), mRNA. (S)	Tmed10	transmembrane emp24-like trafficking protein 10 (yeast); predicted gene 4024	tmed10	6.00E-03	1.553
ILMN_1233836	NM_183308	paraoxonase 2 (Pon2), mRNA. (S)	Pon2	paraoxonase 2	PON2	1.90E-04	1.555
ILMN_1237573	NM_145452	RAS p21 protein activator 1 (Rasa1), mRNA. (S)	Rasa1	RAS p21 protein activator 1	Rasa1	3.92E-02	1.556
ILMN_1240318	NM_011405	solute carrier family 7 (cationic amino acid transporter, y+ system), member 7 (Slc7a7), mRNA. (S)	Slc7a7	solute carrier family 7 (cationic amino acid transporter, y+ system), member 7	SLC7A7	2.32E-03	1.558
ILMN_2898319	NM_153543	aldehyde dehydrogenase 1 family, member L2 (Aldh1l2), mRNA. (S)	Aldh1l2	aldehyde dehydrogenase 1 family, member L2	Aldh1l2	1.46E-02	1.559
ILMN_2677696	NM_013852	ATP-binding cassette, sub-family F (GCN20), member 3 (Abcf3), mRNA. (S)	Abcf3	ATP-binding cassette, sub-family F (GCN20), member 3	ABCF3	1.24E-02	1.560
ILMN_2476267	NM_010380	histocompatibility 2, D region locus 1 (H2-D1), mRNA. (S)	H2-D1	histocompatibility 2, D region; histocompatibility 2, D region locus 1	H2-L	4.89E-03	1.561
ILMN_1224487	NM_172779	DEAD/H (Asp-Glu-Ala-Asp/His) box polypeptide 26B (Ddx26b), mRNA. (S)	Ddx26b	DEAD/H (Asp-Glu-Ala-Asp/His) box polypeptide 26B	ddx26b	9.34E-03	1.562
ILMN_2687905	NM_054043	Musashi homolog 2 (Drosophila) (Msi2), mRNA. (S)	Msi2	Musashi homolog 2 (Drosophila)	MSI2	4.30E-02	1.562
ILMN_1259022	NM_001081006	enhancer trap locus 4 (Etl4), mRNA. (S)	Etl4	enhancer trap locus 4	Etl4	2.29E-03	1.562
ILMN_2503393	NM_133252	translocating chain-associating membrane protein 2 (Tram2), mRNA. (S)	Tram2	translocating chain-associating membrane protein 2	Tram2	4.29E-02	1.562

ILMN_1259668	NM_001033297	gene model 561, (NCBI) (Gm561), mRNA. (S)	Gm561	predicted gene 561	Gm561	3.47E-02	1.563
ILMN_2977350	NM_146061	proline rich 5 (renal) (Prr5), mRNA. (S)	Prr5	Rho GTPase activating protein 8; proline rich 5 (renal)	prr5	1.42E-02	1.564
ILMN_2815024	NM_016893	fucosyltransferase 8 (Fut8), mRNA. (S)	Fut8	fucosyltransferase 8	fut8	3.23E-04	1.564
ILMN_2751194	NM_199079	DEAD (Asp-Glu-Ala-Asp) box polypeptide 17 (Ddx17), transcript variant 2, mRNA. (S)	Ddx17	DEAD (Asp-Glu-Ala-Asp) box polypeptide 17	DDX17	3.28E-02	1.566
ILMN_2949605	NM_026861	ubiquitin associated domain containing 2 (Ubac2), mRNA. (S)	Ubac2	ubiquitin associated domain containing 2	UBAC2	9.05E-03	1.567
ILMN_2652628	NM_010432	homeodomain interacting protein kinase 1 (Hipk1), mRNA. (S)	Hipk1	homeodomain interacting protein kinase 1; similar to protein kinase Myak-S	hipk1	7.64E-03	1.567
ILMN_1239742	NM_009722	ATPase, Ca++ transporting, cardiac muscle, slow twitch 2 (Atp2a2), mRNA. (S)	Atp2a2	ATPase, Ca++ transporting, cardiac muscle, slow twitch 2	ATP2A2	1.16E-02	1.568
ILMN_2623216	NM_016783	progesterone receptor membrane component 1 (Pgrmc1), mRNA. (S)	Pgrmc1	progesterone receptor membrane component 1	PGRMC1	1.12E-03	1.568
ILMN_2795412	NM_025326	transmembrane protein 176A (Tmem176a), mRNA. (S)	Tmem176a	transmembrane protein 176A	Tmem176a	3.70E-02	1.569
ILMN_2718681	XM_001473166	PREDICTED: ADP-ribosylation factor guanine nucleotide-exchange factor 1(brefeldin A-inhibited) (Arfgef1), mRNA. (S)	Arfgef1	ADP-ribosylation factor guanine nucleotide-exchange factor 1(brefeldin A-inhibited)	ARFGEF1	5.78E-06	1.569
ILMN_2805714	NM_020026	UDP-GalNAc:betaGlcNAc beta 1,3-galactosaminyltransferase, polypeptide 1 (B3galnt1), mRNA. (S)	B3galnt1	UDP-GalNAc:betaGlcNAc beta 1,3-galactosaminyltransferase, polypeptide 1	B3galnt1	7.22E-03	1.569
ILMN_2603918	NM_007711	chloride channel 3 (Clcn3), transcript variant a, mRNA. (S)	Clcn3	chloride channel 3	Clcn3	1.23E-03	1.570
ILMN_2606825	NM_007717	NaN (S)	Cmah	cytidine monophospho-N-acetylneuraminic acid hydroxylase	CMAH	2.87E-02	1.570
ILMN_1259355	NM_134033	coiled-coil domain containing 117 (Ccdc117), mRNA. (S)	Ccdc117	coiled-coil domain containing 117	Ccdc117	7.67E-03	1.571
ILMN_1216746	NM_009735	beta-2 microglobulin (B2m), mRNA. (S)	B2m	beta-2 microglobulin	B2M	1.92E-04	1.574
ILMN_2906489	NM_026779	molybdenum cofactor sulfurase (Mocos), mRNA. (S)	Mocos	molybdenum cofactor sulfurase	MOCOS	2.78E-02	1.575

ILMN_1240860	NM_028262	SET domain containing 3 (Setd3), mRNA. (S)	Setd3	SET domain containing 3; similar to CG32732-PA; predicted gene 7114; predicted gene 14026	LOC671440	4.03E-02	1.577
ILMN_2782082	NM_008410	integral membrane protein 2B (Itm2b), mRNA. (S)	Itm2b	integral membrane protein 2B	itm2b	2.82E-03	1.578
ILMN_1239463	NM_010831	SNF1-like kinase (Snf1lk), mRNA. (S)	Snf1lk	salt inducible kinase 1	Sik1	4.68E-02	1.578
ILMN_2701664	NM_010286	TSC22 domain family, member 3 (Tsc22d3), transcript variant 2, mRNA. (S)	Tsc22d3	TSC22 domain family, member 3	TSC22D3	1.89E-02	1.580
ILMN_1253171	NM_138677	ER degradation enhancer, mannosidase alpha-like 1 (Edem1), mRNA. (S)	Edem1	ER degradation enhancer, mannosidase alpha-like 1	EDEM1	1.05E-03	1.583
ILMN_1232948	NaN	NaN (S)	IGKV9-128_AJ231245_Ig_kappa_variable_9-128_15	n/a	n/a	2.12E-04	1.583
ILMN_1256701	XM_001474596	PREDICTED: RIKEN cDNA 2900016B01 gene (2900016B01Rik), mRNA. (S)	2900016B01Rik	n/a	n/a	3.07E-03	1.583
ILMN_1224142	NM_175106	transmembrane protein 177 (Tmem177), mRNA. (S)	Tmem177	transmembrane protein 177	Tmem177	3.79E-02	1.585
ILMN_1226111	XM_001481017	PREDICTED: hypothetical protein LOC100043821 (LOC100043821), mRNA. (S)	LOC100043821	predicted gene 4671	Gm4671	1.38E-02	1.585
ILMN_1243197	XM_358058	NaN (S)	LOC385109	n/a	n/a	3.87E-02	1.586
ILMN_2924419	NM_010394	histocompatibility 2, Q region locus 7 (H2-Q7), mRNA. (S)	H2-Q7	histocompatibility 2, Q region locus 1; histocompatibility 2, Q region locus 9; similar to H-2 class I histocompatibility antigen, L-D alpha chain precursor; histocompatibility 2, Q region locus 8; histocompatibility 2, Q region locus 2; similar to MHC class Ib antigen; histocompatibility 2, Q region locus 7; histocompatibility 2, Q region locus 6; hypothetical protein LOC100044307; similar to H-2 class I histocompatibility antigen, Q7 alpha chain precursor (QA-2 antigen); RIKEN cDNA 0610037M15 gene	LOC676708	4.83E-03	1.587

ILMN_2421179	NM_001081347	Rho-related BTB domain containing 1 (Rhobtb1), mRNA. (S)	Rhobtb1	Rho-related BTB domain containing 1	Rhobtb1	5.63E-03	1.587
ILMN_2537948	XM_992064	PREDICTED: hypothetical protein LOC624610 (LOC624610), mRNA. (S)	LOC624610	predicted gene, ENSMUSG00000061310	ENSMUSG00000061310	4.72E-02	1.589
ILMN_2741590	NM_139269	phospholipase A2, group XVI (Pla2g16), mRNA. (S)	Pla2g16	phospholipase A2, group XVI	Pla2g16	4.59E-03	1.589
ILMN_2753604	NM_178626	CDC42 small effector 2 (Cdc42se2), mRNA. (S)	Cdc42se2	similar to CDC42 small effector 2; CDC42 small effector 2	CDC42SE2	6.07E-04	1.589
ILMN_2431088	NM_021415	calcium channel, voltage-dependent, T type, alpha 1H subunit (Cacna1h), mRNA. (S)	Cacna1h	calcium channel, voltage-dependent, T type, alpha 1H subunit	CACNA1H	3.56E-03	1.590
ILMN_2592313	NM_025498	presenilin enhancer 2 homolog (C. elegans) (Psenen), mRNA. (S)	Psenen	predicted gene 12396; presenilin enhancer 2 homolog (C. elegans)	Gm12396	2.94E-03	1.590
ILMN_2472861	NM_144516	zinc finger, MYND domain containing 11 (Zmynd11), mRNA. (S)	Zmynd11	zinc finger, MYND domain containing 11	zmynd11	7.49E-03	1.591
ILMN_2628426	NM_138953	elongation factor RNA polymerase II 2 (Ell2), mRNA. XM_922000 XM_922005 XM_922010 XM_992760 XM_992784 XM_992820 XM_992847 (S)	Ell2	elongation factor RNA polymerase II 2	ELL2	7.17E-03	1.592
ILMN_1230586	NM_009421	Tnf receptor-associated factor 1 (Traf1), mRNA. (S)	Traf1	TNF receptor-associated factor 1	Traf1	2.96E-02	1.592
ILMN_2716719	XM_111900	NaN (S)	B230333C21Rik	n/a	n/a	8.57E-03	1.593
ILMN_2843029	NM_010763	mannosidase, alpha, class 1A, member 2 (Man1a2), mRNA. (S)	Man1a2	mannosidase, alpha, class 1A, member 2	MAN1A2	4.36E-02	1.593
ILMN_2695035	NM_144859	praja 2, RING-H2 motif containing (Pja2), transcript variant 2, mRNA. (S)	Pja2	praja 2, RING-H2 motif containing	pja2	2.02E-03	1.593
ILMN_2426115	NaN	NaN (S)	2310024N18Rik	n/a	n/a	2.09E-02	1.593
ILMN_1235795	XM_001477076	PREDICTED: similar to very large inducible GTPase 1 (LOC675594), mRNA. (S)	LOC675594	similar to very large inducible GTPase 1 isoform A	LOC675594	2.88E-02	1.594

ILMN_1228718	NM_011519	syndecan 1 (Sdc1), mRNA. (S)	Sdc1	syndecan 1	sdc1	1.40E-03	1.594
ILMN_2745433	NM_026170	endoplasmic reticulum-golgi intermediate compartment (ERGIC) 1 (Ergic1), mRNA. (S)	Ergic1	endoplasmic reticulum-golgi intermediate compartment (ERGIC) 1	ERGIC1	3.12E-03	1.596
ILMN_2521065	NM_009408	topoisomerase (DNA) I (Top1), mRNA. (S)	Top1	topoisomerase (DNA) I	TOP1	1.39E-03	1.596
ILMN_2770492	NM_019998	NaN (S)	Alg2	asparagine-linked glycosylation 2 homolog (yeast, alpha-1,3-mannosyltransferase)	ALG2	3.61E-02	1.597
ILMN_2773244	XM_355811	NaN (S)	2610009E16Rik	n/a	n/a	1.21E-02	1.597
ILMN_2567940	XM_979562	PREDICTED: ethanolamine kinase 1 (Etnk1), mRNA. (S)	Etnk1	ethanolamine kinase 1	etnk1	1.82E-02	1.598
ILMN_1238335	AK034716	NaN (S)	9430028F23Rik	n/a	n/a	3.78E-03	1.599
ILMN_2683080	NM_027016	SEC62 homolog (S. cerevisiae) (Sec62), mRNA. (S)	Sec62	SEC62 homolog (S. cerevisiae)	SEC62	2.82E-02	1.600
ILMN_1224479	NM_178196	histone cluster 1, H2bg (Hist1h2bg), mRNA. (S)	Hist1h2bg	histone cluster 1, H2bg; histone cluster 1, H2be; histone cluster 2, H2bb; histone cluster 1, H2bc	Hist1h2bc	4.28E-02	1.600
ILMN_2605694	NM_025327	keratinocyte associated protein 2 (Krtcap2), mRNA. (S)	Krtcap2	keratinocyte associated protein 2	KRTCAP2	9.43E-03	1.602
ILMN_2631576	XM_148071	NaN (S)	Dnajb11	n/a	n/a	5.97E-04	1.603
ILMN_2739999	NM_009735	beta-2 microglobulin (B2m), mRNA. (S)	B2m	beta-2 microglobulin	B2M	4.64E-05	1.603
ILMN_2696008	XM_001475723	PREDICTED: similar to Nedd4 WW domain-binding protein 5 (LOC100046168), mRNA. (S)	LOC100046168	Nedd4 family interacting protein 1; similar to Nedd4 WW domain-binding protein 5	LOC100046168	3.38E-03	1.604
ILMN_2696629	NM_008400	integrin alpha L (Itgal), mRNA. (S)	Itgal	integrin alpha L	ITGAL	2.14E-03	1.604
ILMN_2730714	NM_026617	transmembrane BAX inhibitor motif containing 4 (Tmbim4), mRNA. (S)	Tmbim4	transmembrane BAX inhibitor motif containing 4	Tmbim4	1.74E-03	1.605
ILMN_3092673	NM_212470	RIKEN cDNA 0610007C21 gene (0610007C21Rik), transcript variant 2, mRNA. (A)	0610007C21Rik	RIKEN cDNA 0610007C21 gene	0610007C21Rik	6.32E-03	1.608

ILMN_1255736	NM_009503	valosin containing protein (Vcp), mRNA. (S)	Vcp	similar to Transitional endoplasmic reticulum ATPase (TER ATPase) (15S Mg(2+)-ATPase p97 subunit) (Valosin-containing protein) (VCP); valosin containing protein	LOC675857	8.58E-03	1.609
ILMN_1253900	XM_489186	NaN (S)	1810026B05Rik	n/a	n/a	1.24E-02	1.610
ILMN_2957070	NM_011368	src homology 2 domain-containing transforming protein C1 (Shc1), mRNA. (S)	Shc1	predicted gene 5500; src homology 2 domain-containing transforming protein C1	SHC1	1.72E-02	1.611
ILMN_2712280	NM_025661	ORM1-like 3 (S. cerevisiae) (Ormdl3), mRNA. (S)	Ormdl3	ORM1-like 3 (S. cerevisiae); predicted gene 12538	Gm12538	4.01E-04	1.613
ILMN_2599400	NM_178661	cAMP responsive element binding protein 3-like 2 (Creb3l2), mRNA. (S)	Creb3l2	cAMP responsive element binding protein 3-like 2	CREB3L2	5.54E-03	1.613
ILMN_2718789	NM_020011	sphingosine kinase 2 (Sphk2), transcript variant 2, mRNA. (S)	Sphk2	sphingosine kinase 2	SPHK2	9.43E-03	1.613
ILMN_2543655	AK007556	NaN (S)	1810020C02Rik	ectonucleoside triphosphate diphosphohydrolase 7	ENTPD7	5.81E-03	1.614
ILMN_2606804	NM_009655	activated leukocyte cell adhesion molecule (Alcam), mRNA. (S)	Alcam	activated leukocyte cell adhesion molecule	Alcam	7.68E-03	1.615
ILMN_1253680	NM_027297	PRP4 pre-mRNA processing factor 4 homolog (yeast) (Prpf4), mRNA. (S)	Prpf4	PRP4 pre-mRNA processing factor 4 homolog (yeast)	PRPF4	4.84E-02	1.616
ILMN_2465985	XM_987671	PREDICTED: similar to Ig kappa chain V-V region MPC11 precursor (LOC676136), mRNA. (S)	LOC676136	similar to Ig kappa chain V-V region MPC11 precursor	LOC676136	2.53E-02	1.616
ILMN_2595973	NM_008175	granulin (Grn), mRNA. (S)	Grn	granulin	grn	1.35E-02	1.618
ILMN_2684279	NM_145537	ER degradation enhancer, mannosidase alpha-like 2 (Edem2), mRNA. (S)	Edem2	ER degradation enhancer, mannosidase alpha-like 2	EDEM2	8.18E-04	1.619
ILMN_1216590	NM_184053	calumenin (Calu), transcript variant 2, mRNA. (S)	Calu	calumenin	CALU	2.32E-02	1.620
ILMN_1215644	XR_032896	PREDICTED: similar to Eukaryotic translation initiation factor 2 alpha kinase 3 (LOC100047634), misc RNA. (S)	LOC100047634	similar to Eukaryotic translation initiation factor 2 alpha kinase 3	LOC100047634	1.93E-04	1.622
ILMN_2623046	NM_007874	receptor accessory protein 5 (Reep5), mRNA. (S)	Reep5	receptor accessory protein 5	REEP5	5.49E-03	1.623

ILMN_2847618	NM_018854	intraflagellar transport 20 homolog (Chlamydomonas) (Ift20), mRNA. (S)	Ift20	intraflagellar transport 20 homolog (Chlamydomonas)	IFT20	4.71E-02	1.623
ILMN_2639805	NM_010956	oxoglutarate dehydrogenase (lipoamide) (Ogdh), nuclear gene encoding mitochondrial protein, mRNA. (S)	Ogdh	oxoglutarate dehydrogenase (lipoamide)	Ogdh	1.72E-02	1.624
ILMN_1229263	XM_001476320	PREDICTED: similar to Ig kappa V-region 24B (LOC100046496), mRNA. (S)	LOC100046496	similar to Ig kappa V-region 24B	LOC100046496	7.56E-03	1.625
ILMN_2725927	NM_009251	serine (or cysteine) peptidase inhibitor, clade A, member 3G (Serpina3g), mRNA. (S)	Serpina3g	serine (or cysteine) peptidase inhibitor, clade A, member 3G	Serpina3g	6.90E-03	1.626
ILMN_3130172	NM_001077411	glucosidase, beta, acid (Gba), mRNA. (A)	Gba	glucosidase, beta, acid	GBA	8.96E-04	1.626
ILMN_1245768	NM_010492	islet cell autoantigen 1 (Ica1), mRNA. (S)	Ica1	islet cell autoantigen 1	Ica1	1.74E-02	1.627
ILMN_1212755	XM_903363	PREDICTED: similar to Ig heavy chain V region MC101 precursor (LOC630242), mRNA. (S)	LOC630242	similar to Ig heavy chain V region MC101 precursor	LOC630242	4.15E-02	1.629
ILMN_2493747	NM_029572	thioredoxin domain containing 4 (endoplasmic reticulum) (Txndc4), mRNA. (S)	Txndc4	endoplasmic reticulum protein 44	ERP44	3.44E-04	1.630
ILMN_1249343	XM_001477847	PREDICTED: similar to Unknown (protein for MGC:41421) (LOC100042270), mRNA. (S)	LOC100042270	predicted gene 8632; predicted gene 3758; predicted gene 5222; similar to Unknown (protein for MGC:41421)	Gm8632	4.31E-02	1.631
ILMN_2458986	NM_025824	NaN (S)	Bzw1	predicted gene 11652; predicted gene 5191; basic leucine zipper and W2 domains 1	BZW1	4.99E-03	1.632
ILMN_1236553	NM_174960	GTPase, IMAP family member 9 (Gimap9), mRNA. (S)	Gimap9	GTPase, IMAP family member 9	Gimap9	4.64E-03	1.633
ILMN_2648991	XM_001477383	PREDICTED: similar to farnesyltransferase alpha subunit (LOC100046996), mRNA. (S)	LOC100046996	farnesyltransferase, CAAX box, alpha; similar to farnesyltransferase alpha subunit	FNTA	1.28E-02	1.635
ILMN_1221935	NM_009752	galactosidase, beta 1 (Glb1), mRNA. (S)	Glb1	galactosidase, beta 1	Glb1	3.90E-04	1.635
ILMN_3136196	NM_008033	farnesyltransferase, CAAX box, alpha (Fnta), mRNA. (A)	Fnta	farnesyltransferase, CAAX box, alpha; similar to farnesyltransferase alpha subunit	FNTA	4.18E-02	1.637

ILMN_1241128	NM_026192	calcium binding and coiled coil domain 1 (Calcoco1), mRNA. (S)	Calcoco1	calcium binding and coiled coil domain 1	CALCOCO1	3.92E-02	1.638
ILMN_2444662	NaN	NaN (S)	2900037O03Rik	n/a	n/a	1.26E-03	1.641
ILMN_1215825	XM_001476590	PREDICTED: similar to anti-MOG Z12 variable light chain (LOC100047316), mRNA. (S)	LOC100047316	predicted gene 1502; similar to anti-MOG Z12 variable light chain	Gm1502	1.14E-02	1.642
ILMN_2607953	NM_027481	splicing factor 4 (Sf4), mRNA. (S)	Sf4	splicing factor 4	SF4	8.91E-03	1.642
ILMN_2431237	NM_013842	X-box binding protein 1 (Xbp1), mRNA. (S)	Xbp1	X-box binding protein 1	Xbp1	7.90E-04	1.643
ILMN_2700168	NM_009829	NaN (S)	Ccnd2	cyclin D2	CCND2	8.53E-03	1.643
ILMN_2660414	NM_025442	asparagine-linked glycosylation 5 homolog (yeast, dolichyl-phosphate beta-glucosyltransferase) (Alg5), mRNA. (S)	Alg5	asparagine-linked glycosylation 5 homolog (yeast, dolichyl-phosphate beta-glucosyltransferase)	Alg5	4.07E-04	1.645
ILMN_2780424	NM_007564	zinc finger protein 36, C3H type-like 1 (Zfp36l1), mRNA. (S)	Zfp36l1	zinc finger protein 36, C3H type-like 1	zfp36l1	7.35E-03	1.647
ILMN_1217886	XM_984967	PREDICTED: similar to Igh-1a protein (LOC675759), mRNA. (S)	LOC675759	similar to Ig heavy chain V-III region J606	LOC676473	3.15E-02	1.650
ILMN_2664628	NM_010015	NaN (S)	Dad1	defender against cell death 1	DAD1	4.78E-05	1.651
ILMN_2500533	NM_178114	adhesion molecule with Ig like domain 2 (Amigo2), mRNA. (S)	Amigo2	adhesion molecule with Ig like domain 2	AMIGO2	2.43E-02	1.654
ILMN_2763739	XM_924235	PREDICTED: cDNA sequence BC032203, transcript variant 4 (BC032203), mRNA. (S)	BC032203	cDNA sequence BC032203	BC032203	2.15E-02	1.654
ILMN_2718791	NM_020011	sphingosine kinase 2 (Sphk2), transcript variant 2, mRNA. (S)	Sphk2	sphingosine kinase 2	SPHK2	1.32E-02	1.655
ILMN_1216473	NM_029582	thioredoxin domain containing 11 (Txndc11), transcript variant 1, mRNA. (S)	Txndc11	thioredoxin domain containing 11	TXNDC11	3.86E-02	1.655
ILMN_1227163	NM_027350	asparaginyl-tRNA synthetase (Nars), mRNA. (S)	Nars	asparaginyl-tRNA synthetase	Nars	9.35E-03	1.657
ILMN_2765524	NM_009302	SWA-70 protein (Swap70), mRNA. (S)	Swap70	SWA-70 protein	SWAP70	1.16E-03	1.657

ILMN_2760430	NM_022993	low-density lipoprotein receptor-related protein 10 (Lrp10), mRNA. (S)	Lrp10	low-density lipoprotein receptor-related protein 10	LRP10	4.74E-03	1.659
ILMN_1225535	NM_008060	alpha glucosidase 2 alpha neutral subunit (Ganab), mRNA. (S)	Ganab	alpha glucosidase 2 alpha neutral subunit	GANAB	1.16E-02	1.660
ILMN_2447875	NaN	NaN (S)	6330509M05Rik	n/a	n/a	1.67E-02	1.661
ILMN_3086889	NM_001038018	G protein-coupled receptor kinase 6 (Gprk6), transcript variant 1, mRNA. (A)	Gprk6	G protein-coupled receptor kinase 6	grk6	1.36E-03	1.662
ILMN_2776278	NM_008529	NaN (S)	Ly6e	lymphocyte antigen 6 complex, locus E	Ly6e	9.68E-04	1.663
ILMN_1245092	NM_011149	peptidylprolyl isomerase B (Ppib), mRNA. (S)	Ppib	peptidylprolyl isomerase B	ppiB	4.31E-03	1.663
ILMN_1247853	NM_007564	zinc finger protein 36, C3H type-like 1 (Zfp36l1), mRNA. (S)	Zfp36l1	zinc finger protein 36, C3H type-like 1	zfp36l1	8.28E-03	1.664
ILMN_2910934	NM_013706	CD52 antigen (Cd52), mRNA. (S)	Cd52	CD52 antigen	Cd52	4.67E-03	1.665
ILMN_3154457	NM_144859	praja 2, RING-H2 motif containing (Pja2), transcript variant 2, mRNA. (A)	Pja2	praja 2, RING-H2 motif containing	pja2	2.38E-03	1.666
ILMN_1228653	NM_021394	Z-DNA binding protein 1 (Zbp1), mRNA. (S)	Zbp1	Z-DNA binding protein 1	Zbp1	3.20E-02	1.666
ILMN_1246861	NM_021281	cathepsin S (Ctss), mRNA. (S)	Ctss	cathepsin S	CTSS	3.55E-03	1.666
ILMN_2884610	NM_028000	phosphatidic acid phosphatase type 2 domain containing 1B (Ppapdc1b), mRNA. (S)	Ppapdc1b	phosphatidic acid phosphatase type 2 domain containing 1B	ppapdc1b	4.10E-02	1.666
ILMN_2588139	NM_001001892	histocompatibility 2, K1, K region (H2-K1), transcript variant 1, mRNA. (S)	H2-K1	histocompatibility 2, K1, K region; similar to H-2K(d) antigen	H2-K1	7.88E-04	1.666
ILMN_1218525	NM_008365	interleukin 18 receptor 1 (Il18r1), mRNA. (S)	Il18r1	interleukin 18 receptor 1	Il18r1	3.26E-02	1.667
ILMN_2766455	NM_178701	leucine rich repeat containing 8D (Lrrc8d), mRNA. (S)	Lrrc8d	leucine rich repeat containing 8D	LRRC8D	2.57E-03	1.667
ILMN_1219017	NaN	NaN (S)	5031436O03Rik	n/a	n/a	1.75E-02	1.670

ILMN_1254173	NM_008640	lysosomal-associated protein transmembrane 4A (Laptm4a), mRNA. (S)	Laptm4a	lysosomal-associated protein transmembrane 4A	LAPTM4A	1.08E-04	1.670
ILMN_1247633	NM_011981	zinc finger protein 260 (Zfp260), mRNA. (S)	Zfp260	zinc finger protein 260	Zfp260	7.98E-05	1.676
ILMN_2782964	NM_008813	ectonucleotide pyrophosphatase/phosphodiesterase 1 (Enpp1), mRNA. (S)	Enpp1	ectonucleotide pyrophosphatase/phosphodiesterase 1	Enpp1	6.79E-03	1.676
ILMN_2892507	NM_010242	fucosyltransferase 4 (Fut4), mRNA. (S)	Fut4	fucosyltransferase 4	FUT4	3.33E-02	1.676
ILMN_2493097	NaN	NaN (S)	IGHV9S5_L14364_Ig_heavy_variable_9S5_82	n/a	n/a	6.23E-03	1.679
ILMN_2677207	XR_033948	PREDICTED: similar to gamma-2a immunoglobulin heavy chain (LOC100047788), misc RNA. (S)	LOC100047788	similar to gamma-2a immunoglobulin heavy chain; immunoglobulin heavy chain 1a (serum IgG2a); immunoglobulin heavy chain 1b (serum IgG2c)	Igh-1b	2.23E-02	1.680
ILMN_1251370	NM_019443	NADH dehydrogenase (ubiquinone) 1 alpha subcomplex, 1 (Ndufa1), nuclear gene encoding mitochondrial protein, mRNA. (S)	Ndufa1	NADH dehydrogenase (ubiquinone) 1 alpha subcomplex, 1	NDUFA1	5.09E-04	1.680
ILMN_1224678	NM_029626	glycosyltransferase 8 domain containing 1 (Glt8d1), mRNA. (S)	Glt8d1	glycosyltransferase 8 domain containing 1	glt8d1	8.64E-03	1.682
ILMN_2712557	NM_024439	histocompatibility 47 (H47), mRNA. (S)	H47	histocompatibility 47	H47	1.16E-02	1.682
ILMN_2681492	NM_025808	leucine-zipper-like transcriptional regulator, 1 (Lztr1), mRNA. (S)	Lztr1	leucine-zipper-like transcriptional regulator, 1	Lztr1	1.36E-02	1.683
ILMN_1218923	XM_001477842	PREDICTED: hypothetical protein LOC100041932 (LOC100041932), mRNA. (S)	LOC100041932	predicted gene 3579	Gm3579	1.14E-02	1.685
ILMN_2475959	XM_484191	PREDICTED: similar to Ig heavy chain V-II region SESS precursor (LOC432709), mRNA. (S)	LOC432709	predicted gene 5440	Gm5440	3.37E-03	1.685
ILMN_1220430	NM_025455	coiled coil domain containing 28B (Ccdc28b), mRNA. (S)	Ccdc28b	coiled coil domain containing 28B	Ccdc28b	1.44E-02	1.685

ILMN_2484527	NM_009505	vascular endothelial growth factor A (Vegfa), transcript variant 2, mRNA. (S)	Vegfa	vascular endothelial growth factor A	VEGFA	1.04E-03	1.685
ILMN_3137920	NM_001039089	sel-1 suppressor of lin-12-like (C. elegans) (Sel1l), transcript variant 1, mRNA. (A)	Sel1l	sel-1 suppressor of lin-12-like (C. elegans)	SEL1L	2.47E-04	1.686
ILMN_2646625	NM_010591	Jun oncogene (Jun), mRNA. (S)	Jun	Jun oncogene	Jun	1.38E-03	1.687
ILMN_2680371	XM_484644	NaN (S)	D130084M03Rik	n/a	n/a	1.65E-02	1.687
ILMN_2735996	NM_011032	prolyl 4-hydroxylase, beta polypeptide (P4hb), mRNA. (S)	P4hb	prolyl 4-hydroxylase, beta polypeptide	p4hb	1.94E-03	1.689
ILMN_1259470	NM_023056	transmembrane protein 176B (Tmem176b), mRNA. (S)	Tmem176b	transmembrane protein 176B	TMEM176B	1.29E-02	1.689
ILMN_2944601	NM_001004146	RIKEN cDNA 4933439C20 gene (4933439C20Rik), mRNA. (S)	4933439C20Rik	phosphatidylserine decarboxylase, pseudogene 1	Pisd-ps1	1.28E-03	1.689
ILMN_2639579	NM_178690	RAB3 GTPase activating protein subunit 1 (Rab3gap1), mRNA. (S)	Rab3gap1	RAB3 GTPase activating protein subunit 1	Rab3gap1	5.56E-03	1.689
ILMN_2481170	NM_028075	tumor necrosis factor receptor superfamily, member 13c (Tnfrsf13c), mRNA. (S)	Tnfrsf13c	tumor necrosis factor receptor superfamily, member 13c	TNFRSF13C	3.51E-02	1.689
ILMN_2773153	NM_008370	interleukin 5 receptor, alpha (Il5ra), mRNA. (S)	Il5ra	interleukin 5 receptor, alpha	Il5ra	1.61E-03	1.689
ILMN_1255148	AK039008	NaN (S)	H13	histocompatibility 13	H13	1.06E-02	1.690
ILMN_2663360	XM_908869	PREDICTED: similar to Ig heavy chain V region VH558 A1/A4 precursor (LOC434609), mRNA. (S)	LOC434609	predicted gene, EG434609	Gm5629	3.58E-02	1.691
ILMN_2469743	XM_001476823	PREDICTED: similar to light chain of the monoclonal antibody MST2 (LOC100046793), mRNA. (S)	LOC100046793	predicted gene 10880	Gm10880	3.20E-02	1.692
ILMN_2440960	NaN	NaN (S)	IGHG1_J00453$V00793_Ig_heavy_constant_gamma_1_792	n/a	n/a	1.15E-02	1.692

ILMN_2708169	NM_018788	exostoses (multiple)-like 3 (Extl3), mRNA. (S)	Extl3	exostoses (multiple)-like 3	extl3	1.42E-02	1.694
ILMN_2498263	NM_010478	heat shock protein 1B (Hspa1b), mRNA. (S)	Hspa1b	heat shock protein 1B; heat shock protein 1A; heat shock protein 1-like	Hspa1l	1.58E-02	1.695
ILMN_2751314	XM_0010011861	PREDICTED: golgi autoantigen, golgin subfamily b, macrogolgin 1, transcript variant 9 (Golgb1), mRNA. (S)	Golgb1	golgi autoantigen, golgin subfamily b, macrogolgin 1	GOLGB1	3.91E-03	1.698
ILMN_2666190	NM_181405	arginyl aminopeptidase (aminopeptidase B)-like 1 (Rnpepl1), mRNA. (S)	Rnpepl1	arginyl aminopeptidase (aminopeptidase B)-like 1	RNPEPL1	4.51E-02	1.698
ILMN_2925350	NM_007437	aldehyde dehydrogenase family 3, subfamily A2 (Aldh3a2), mRNA. (S)	Aldh3a2	aldehyde dehydrogenase family 3, subfamily A2	aldh3a2	1.98E-02	1.705
ILMN_2798993	NM_011584	nuclear receptor subfamily 1, group D, member 2 (Nr1d2), mRNA. (S)	Nr1d2	nuclear receptor subfamily 1, group D, member 2; predicted gene 5827	NR1D2	4.08E-03	1.706
ILMN_2653215	NM_019542	N-acetylglucosamine kinase (Nagk), mRNA. (S)	Nagk	N-acetylglucosamine kinase	nagK	3.83E-02	1.707
ILMN_2834677	NM_145940	WD repeat domain, phosphoinositide interacting 1 (Wipi1), mRNA. (S)	Wipi1	WD repeat domain, phosphoinositide interacting 1	WIPI1	2.34E-04	1.708
ILMN_2543456	NM_026828	DNA segment, Chr 2, Brigham & Women's Genetics 1335 expressed (D2Bwg1335e), mRNA. (S)	D2Bwg1335e	DNL-type zinc finger	DNLZ	4.35E-04	1.709
ILMN_2745775	XM_135590	NaN (S)	LOC213684	n/a	n/a	1.07E-02	1.710
ILMN_2429203	NM_172151	zinc finger, DHHC domain containing 8 (Zdhhc8), mRNA. (S)	Zdhhc8	zinc finger, DHHC domain containing 8	Zdhhc8	1.53E-02	1.715
ILMN_2603837	NM_016865	HIV-1 tat interactive protein 2, homolog (human) (Htatip2), mRNA. (S)	Htatip2	HIV-1 tat interactive protein 2, homolog (human)	htatip2	1.10E-03	1.715
ILMN_1256927	NM_178618	RIKEN cDNA 2310040C09 gene (2310040C09Rik), mRNA. (S)	2310040C09Rik	family with sequence similarity 83, member G	FAM83G	1.91E-02	1.716

ILMN_1245042	NaN	NaN (S)	IGKV9-120_V00804$J00566_Ig_kappa_variable_9-120_12	n/a	n/a	2.85E-02	1.716
ILMN_2548729	AK015410	NaN (S)	Dnm2	dynamin 2	DNM2	4.54E-03	1.721
ILMN_2525980	NM_145933	beta galactoside alpha 2,6 sialyltransferase 1 (St6gal1), mRNA. (S)	St6gal1	beta galactoside alpha 2,6 sialyltransferase 1	ST6GAL1	1.00E-02	1.721
ILMN_2931095	NM_010515	insulin-like growth factor 2 receptor (Igf2r), mRNA. (S)	Igf2r	insulin-like growth factor 2 receptor	Igf2r	3.68E-03	1.723
ILMN_2477221	NM_010828	Cbp/p300-interacting transactivator, with Glu/Asp-rich carboxy-terminal domain, 2 (Cited2), mRNA. (S)	Cited2	Cbp/p300-interacting transactivator, with Glu/Asp-rich carboxy-terminal domain, 2	Cited2	1.05E-02	1.723
ILMN_2611295	NM_025869	dual specificity phosphatase 26 (putative) (Dusp26), mRNA. (S)	Dusp26	dual specificity phosphatase 26 (putative)	DUSP26	3.34E-03	1.724
ILMN_2887065	NM_080638	major vault protein (Mvp), mRNA. (S)	Mvp	major vault protein	MVP	5.78E-03	1.726
ILMN_2550962	AK088947	NaN (S)	E430033B07Rik	SH3-domain GRB2-like (endophilin) interacting protein 1	Sgip1	1.11E-03	1.728
ILMN_2769567	NM_007974	NaN (S)	F2rl1	coagulation factor II (thrombin) receptor-like 1	f2rl1	3.43E-04	1.728
ILMN_3118707	NM_009192	src-like adaptor (Sla), transcript variant 2, mRNA. (A)	Sla	src-like adaptor	SLA	8.37E-04	1.731
ILMN_2419051	XM_355822	NaN (S)	2700089E24Rik	n/a	n/a	9.97E-03	1.732
ILMN_1257575	NM_009976	cystatin C (Cst3), mRNA. (S)	Cst3	cystatin C	Cst3	5.83E-04	1.734
ILMN_2799667	NM_027977	RIKEN cDNA 2310001A20 gene (2310001A20Rik), mRNA. (S)	2310001A20Rik	RIKEN cDNA 2310001A20 gene	2310001A20Rik	2.48E-04	1.734
ILMN_2908687	NM_207246	RAS, guanyl releasing protein 3 (Rasgrp3), mRNA. (S)	Rasgrp3	RAS, guanyl releasing protein 3	rasgrp3	8.82E-04	1.735

ILMN_1233237	NM_018889	phosphatidylinositol glycan anchor biosynthesis, class B (Pigb), mRNA. (S)	Pigb	phosphatidylinositol glycan anchor biosynthesis, class B	PIGB	3.34E-03	1.735
ILMN_2463573	XM_001476823	PREDICTED: similar to light chain of the monoclonal antibody MST2 (LOC100046793), mRNA. (S)	LOC100046793	predicted gene 10880	Gm10880	1.60E-02	1.736
ILMN_2775381	NM_019760	NaN (S)	Tde2	serine incorporator 1	SERINC1	1.96E-03	1.742
ILMN_1241827	NM_133891	solute carrier family 44, member 1 (Slc44a1), mRNA. (S)	Slc44a1	solute carrier family 44, member 1	slc44a1	2.53E-02	1.743
ILMN_1234223	NM_178911	phospholipase D family, member 4 (Pld4), mRNA. (S)	Pld4	phospholipase D family, member 4	PLD4	1.27E-04	1.750
ILMN_1227726	NM_001001295	DIS3 mitotic control homolog (S. cerevisiae)-like (Dis3l), transcript variant 1, mRNA. (S)	Dis3l	DIS3 mitotic control homolog (S. cerevisiae)-like	Dis3l	2.31E-02	1.752
ILMN_1249003	XM_130346	NaN (S)	Gpr155	n/a	n/a	6.47E-03	1.752
ILMN_2603834	NM_016865	HIV-1 tat interactive protein 2, homolog (human) (Htatip2), mRNA. (S)	Htatip2	HIV-1 tat interactive protein 2, homolog (human)	htatip2	1.57E-03	1.754
ILMN_3144164	NM_001081212	insulin receptor substrate 2 (Irs2), mRNA. (A)	Irs2	insulin receptor substrate 2	irs2	4.14E-02	1.757
ILMN_1221719	NM_008144	Bernardinelli-Seip congenital lipodystrophy 2 homolog (human) (Bscl2), transcript variant 2, mRNA. (S)	Bscl2	Bernardinelli-Seip congenital lipodystrophy 2 homolog (human)	BSCL2	3.69E-03	1.757
ILMN_2615739	XM_992644	PREDICTED: gene model 459, (NCBI) (Gm459), mRNA. (S)	Gm459	predicted gene 459	Gm459	1.20E-02	1.760
ILMN_1215967	NM_025442	asparagine-linked glycosylation 5 homolog (yeast, dolichyl-phosphate beta-glucosyltransferase) (Alg5), mRNA. (S)	Alg5	asparagine-linked glycosylation 5 homolog (yeast, dolichyl-phosphate beta-glucosyltransferase)	Alg5	6.05E-03	1.762
ILMN_1249765	NM_024233	REX2, RNA exonuclease 2 homolog (S. cerevisiae) (Rexo2), mRNA. (S)	Rexo2	REX2, RNA exonuclease 2 homolog (S. cerevisiae)	Rexo2	7.36E-03	1.763

ILMN_2422974	NaN	NaN (S)	IGHV1S124_AF025449_Ig_heavy_variable_1S124_11	n/a	n/a	7.50E-03	1.764
ILMN_1250618	NM_175537	zinc finger and BTB domain containing 38 (Zbtb38), mRNA. (S)	Zbtb38	zinc finger and BTB domain containing 38	ZBTB38	1.28E-02	1.764
ILMN_1246201	NM_021415	calcium channel, voltage-dependent, T type, alpha 1H subunit (Cacna1h), mRNA. (S)	Cacna1h	calcium channel, voltage-dependent, T type, alpha 1H subunit	CACNA1H	5.47E-03	1.765
ILMN_1257702	NM_134137	leucyl-tRNA synthetase (Lars), mRNA. XM_901187 XM_913429 XM_922755 XM_922767 XM_922771 XM_922775 XM_922782 XM_922785 XM_989215 (S)	Lars	leucyl-tRNA synthetase	LARS	1.00E-03	1.766
ILMN_1228288	AK038731	NaN (S)	A230057M07Rik	zinc finger and BTB domain containing 20	ZBTB20	3.21E-03	1.767
ILMN_2716098	NM_013559	NaN (S)	Hsp105	heat shock 105kDa/110kDa protein 1	HSPH1	3.75E-02	1.767
ILMN_1255416	NM_010738	lymphocyte antigen 6 complex, locus A (Ly6a), mRNA. (S)	Ly6a	lymphocyte antigen 6 complex, locus A	Ly6a	2.58E-03	1.768
ILMN_2790246	NM_022331	homocysteine-inducible, endoplasmic reticulum stress-inducible, ubiquitin-like domain member 1 (Herpud1), mRNA. (S)	Herpud1	homocysteine-inducible, endoplasmic reticulum stress-inducible, ubiquitin-like domain member 1	HERPUD1	8.03E-05	1.769
ILMN_2971479	NM_021897	transformation related protein 53 inducible nuclear protein 1 (Trp53inp1), mRNA. (S)	Trp53inp1	transformation related protein 53 inducible nuclear protein 1	Trp53inp1	6.03E-04	1.770
ILMN_1247541	XM_147738	NaN (S)	2900016G23Rik	n/a	n/a	6.00E-03	1.771
ILMN_2515956	NaN	NaN (S)	Igh-4	n/a	n/a	3.76E-02	1.775
ILMN_2880052	NM_183094	X-linked lymphocyte-regulated 4C (Xlr4c), mRNA. (S)	Xlr4c	X-linked lymphocyte-regulated 4D; X-linked lymphocyte-regulated 4E, pseudogene; X-linked lymphocyte-regulated 4B; X-linked lymphocyte-regulated 4C; hypothetical protein LOC100044049	Xlr4c	1.86E-03	1.777

ILMN_2867241	NM_173006	paraoxonase 3 (Pon3), mRNA. (S)	Pon3	paraoxonase 3	PON3	1.40E-02	1.777
ILMN_2481117	NaN	NaN (S)	1700052O22Rik	n/a	n/a	3.71E-04	1.778
ILMN_2660803	NM_199195	branched chain ketoacid dehydrogenase E1, beta polypeptide (Bckdhb), nuclear gene encoding mitochondrial protein, mRNA. (S)	Bckdhb	branched chain ketoacid dehydrogenase E1, beta polypeptide; similar to 3-methyl-2-oxobutanoate dehydrogenase	LOC100048676	1.02E-02	1.783
ILMN_2607675	XM_918601	PREDICTED: similar to MHC class II antigen beta chain (LOC641240), mRNA. (S)	LOC641240	histocompatibility 2, class II antigen A, beta 1; response to metastatic cancers 2; similar to H-2 class II histocompatibility antigen, A-D beta chain precursor	Rmcs2	7.72E-03	1.785
ILMN_1258376	NM_001033525	potassium inwardly-rectifying channel, subfamily K, member 6 (Kcnk6), mRNA. (S)	Kcnk6	potassium inwardly-rectifying channel, subfamily K, member 6	kcnk6	1.41E-03	1.792
ILMN_1242466	NM_013585	proteasome (prosome, macropain) subunit, beta type 9 (large multifunctional peptidase 2) (Psmb9), mRNA. (S)	Psmb9	proteasome (prosome, macropain) subunit, beta type 9 (large multifunctional peptidase 2)	psmb9	1.20E-04	1.792
ILMN_2903364	NM_009302	SWA-70 protein (Swap70), mRNA. (S)	Swap70	SWA-70 protein	SWAP70	2.26E-03	1.794
ILMN_1227723	NM_026675	nudix (nucleoside diphosphate linked moiety X)-type motif 22 (Nudt22), mRNA. (S)	Nudt22	nudix (nucleoside diphosphate linked moiety X)-type motif 22	nudt22	2.74E-03	1.797
ILMN_2873988	NM_023386	receptor transporter protein 4 (Rtp4), mRNA. (S)	Rtp4	receptor transporter protein 4	rtp4	1.74E-02	1.798
ILMN_2628258	NM_028769	synovial apoptosis inhibitor 1, synoviolin (Syvn1), mRNA. (S)	Syvn1	synovial apoptosis inhibitor 1, synoviolin	SYVN1	2.40E-02	1.801
ILMN_1255237	NM_009279	signal sequence receptor, delta (Ssr4), mRNA. (S)	Ssr4	signal sequence receptor, delta	Ssr4	9.14E-05	1.802
ILMN_2774462	XM_138373	NaN (S)	LOC238447	n/a	n/a	5.90E-03	1.805
ILMN_2716185	NM_017405	lipolysis stimulated lipoprotein receptor (Lsr), mRNA. (S)	Lsr	lipolysis stimulated lipoprotein receptor	lsr	9.03E-03	1.807

ILMN_1216717	NM_011771	IKAROS family zinc finger 3 (Ikzf3), mRNA. XM_915425 XM_923689 XM_923696 XM_923700 XM_923704 XM_983715 XM_983788 XM_983820 XM_983864 XM_983905 (S)	Ikzf3	IKAROS family zinc finger 3	IKZF3	3.71E-02	1.807
ILMN_2680398	NM_172785	zinc finger CCCH type containing 12D (Zc3h12d), mRNA. (S)	Zc3h12d	zinc finger CCCH type containing 12D	Zc3h12d	1.37E-02	1.808
ILMN_1214934	NM_030248	CDK5 regulatory subunit associated protein 3 (Cdk5rap3), mRNA. (S)	Cdk5rap3	CDK5 regulatory subunit associated protein 3	CDK5RAP3	1.27E-02	1.811
ILMN_2612238	NM_133829	RIKEN cDNA 2210010L05 gene (2210010L05Rik), transcript variant 1, mRNA. (S)	2210010L05Rik	major facilitator superfamily domain containing 6	MFSD6	2.00E-02	1.812
ILMN_1258275	AK014446	NaN (S)	3830421F03Rik	poliovirus receptor	PVR	3.84E-02	1.814
ILMN_1239042	NaN	NaN (S)	Ankhd1	n/a	n/a	2.60E-02	1.818
ILMN_2602938	NM_133888	sphingomyelin phosphodiesterase, acid-like 3B (Smpdl3b), mRNA. (S)	Smpdl3b	sphingomyelin phosphodiesterase, acid-like 3B	Smpdl3b	1.52E-02	1.826
ILMN_3145975	NM_001013786	zinc finger protein 187 (Zfp187), mRNA. (A)	Zfp187	zinc finger protein 187	Zfp187	2.07E-02	1.826
ILMN_1216388	NM_025692	ubiquitin-like modifier activating enzyme 5 (Uba5), mRNA. (S)	Uba5	ubiquitin-like modifier activating enzyme 5	uba5	9.32E-03	1.828
ILMN_1258613	AK050560	NaN (S)	N4wbp5-pending	Nedd4 family interacting protein 1; similar to Nedd4 WW domain-binding protein 5	LOC100046168	2.74E-03	1.829
ILMN_1250947	NM_145367	thioredoxin domain containing 5 (Txndc5), mRNA. (S)	Txndc5	thioredoxin domain containing 5	TXNDC5	1.01E-02	1.833
ILMN_2635572	NM_153288	neuropeptide B (Npb), mRNA. (S)	Npb	neuropeptide B	Npb	4.95E-02	1.837
ILMN_1234931	NM_024440	Der1-like domain family, member 3 (Derl3), mRNA. (S)	Derl3	Der1-like domain family, member 3	Derl3	5.69E-03	1.837
ILMN_2717045	NM_016764	peroxiredoxin 4 (Prdx4), mRNA. (S)	Prdx4	peroxiredoxin 4	Prdx4	2.82E-03	1.846
ILMN_1219154	NM_008630	metallothionein 2 (Mt2), mRNA. (S)	Mt2	metallothionein 2	Mt2	1.10E-02	1.852

ILMN_1232454	NM_010244	NaN (S)	Fv1	Friend virus susceptibility 1	Fv1	2.02E-02	1.853
ILMN_1239535	XM_924800	PREDICTED: pleckstrin homology domain containing, family M (with RUN domain) member 2, transcript variant 7 (Plekhm2), mRNA. (S)	Plekhm2	pleckstrin homology domain containing, family M (with RUN domain) member 2	Plekhm2	2.40E-02	1.857
ILMN_2773447	NM_009279	NaN (S)	Ssr4	signal sequence receptor, delta	Ssr4	1.28E-04	1.859
ILMN_2671767	NM_018854	intraflagellar transport 20 homolog (Chlamydomonas) (Ift20), mRNA. (S)	Ift20	intraflagellar transport 20 homolog (Chlamydomonas)	IFT20	2.62E-02	1.860
ILMN_2750013	XM_985146	PREDICTED: silica-induced gene 111 (Silg111), mRNA. (S)	Silg111	HCLS1 associated X-1; silica-induced gene 111	Gm14270	1.97E-02	1.863
ILMN_2596917	XM_001472256	PREDICTED: similar to SH2/SH3 adaptor protein (LOC100044475), mRNA. (S)	LOC100044475	similar to SH2/SH3 adaptor protein; non-catalytic region of tyrosine kinase adaptor protein 2; predicted gene 6226	NCK2	1.41E-03	1.865
ILMN_2776377	NM_053198	sideroflexin 4 (Sfxn4), mRNA. (S)	Sfxn4	sideroflexin 4	sfxn4	2.67E-02	1.867
ILMN_2667384	NM_008135	solute carrier family 6 (neurotransmitter transporter, glycine), member 9 (Slc6a9), mRNA. (S)	Slc6a9	solute carrier family 6 (neurotransmitter transporter, glycine), member 9	SLC6A9	4.30E-02	1.870
ILMN_2669540	NM_026307	cutA divalent cation tolerance homolog (E. coli) (Cuta), transcript variant 1, mRNA. (S)	Cuta	cutA divalent cation tolerance homolog (E. coli)	cutA	4.69E-03	1.873
ILMN_2761109	NM_013885	chloride intracellular channel 4 (mitochondrial) (Clic4), nuclear gene encoding mitochondrial protein, mRNA. (S)	Clic4	chloride intracellular channel 4 (mitochondrial)	CLIC4	8.30E-04	1.877
ILMN_2701562	NM_026002	NaN (S)	2610103J23Rik	metadherin	MTDH	3.06E-03	1.879
ILMN_1241467	XM_001006762	PREDICTED: similar to Igh protein (LOC677648), mRNA. (S)	LOC677648	predicted gene, 780938; similar to Ig heavy chain V-II region SESS precursor; predicted gene 7178; predicted gene 7297	LOC676347	4.87E-03	1.884

ILMN_2450155	NaN	NaN (S)	IGLC2_J00595_Ig_lambda_constant_2_14	n/a	n/a	1.22E-02	1.886
ILMN_2771380	NM_013706	NaN (S)	Cd52	CD52 antigen	Cd52	1.43E-02	1.887
ILMN_1230183	XM_130346	NaN (S)	Gpr155	n/a	n/a	5.80E-03	1.887
ILMN_1226398	XM_125627	PREDICTED: RIKEN cDNA 9430073N08 gene (9430073N08Rik), mRNA. (S)	9430073N08Rik	family with sequence similarity 162, member B	Fam162b	4.98E-06	1.888
ILMN_1247133	NM_001083587	tensin 3 (Tns3), mRNA. (S)	Tns3	tensin 3	TNS3	6.56E-03	1.889
ILMN_3162925	NM_175219	RIKEN cDNA C130026I21 gene (C130026I21Rik), transcript variant 1, mRNA. (I)	C130026I21Rik	RIKEN cDNA C130026I21 gene; similar to SP140 nuclear body protein family member; similar to RIKEN cDNA C130026I21 gene	C130026I21Rik	1.99E-04	1.890
ILMN_2510875	NM_013760	DnaJ (Hsp40) homolog, subfamily B, member 9 (Dnajb9), mRNA. (S)	Dnajb9	predicted gene 6568; DnaJ (Hsp40) homolog, subfamily B, member 9	dnajb9	2.67E-05	1.893
ILMN_2700166	NM_009829	cyclin D2 (Ccnd2), mRNA. (S)	Ccnd2	cyclin D2	CCND2	7.61E-03	1.894
ILMN_1220104	NM_027349	NaN (S)	2600011C06Rik	RNA binding motif protein 25	RBM25	1.88E-02	1.895
ILMN_2597923	NM_007548	PR domain containing 1, with ZNF domain (Prdm1), mRNA. (S)	Prdm1	PR domain containing 1, with ZNF domain	PRDM1	5.02E-03	1.897
ILMN_1224434	NM_026002	Metadherin (Mtdh), mRNA. (S)	Mtdh	metadherin	MTDH	1.91E-03	1.900
ILMN_1246517	NM_133203	killer cell lectin-like receptor, subfamily A, member 17 (Klra17), mRNA. (S)	Klra17	killer cell lectin-like receptor, subfamily A, member 17; killer cell lectin-like receptor, subfamily A, member 17B	Klra17b	1.55E-02	1.900
ILMN_2589312	NM_026174	ectonucleoside triphosphate diphosphohydrolase 4 (Entpd4), mRNA. (S)	Entpd4	ectonucleoside triphosphate diphosphohydrolase 4	entpd4	2.74E-02	1.904
ILMN_1246495	NM_020050	TMEM9 domain family, member B (Tmem9b), mRNA. (S)	Tmem9b	TMEM9 domain family, member B	TMEM9B	1.33E-02	1.906

ILMN_2470564	NaN	NaN (S)	IGHV1S120_AF025443_Ig_heavy_variable_1S120_8	n/a	n/a	5.67E-03	1.906
ILMN_3060512	NM_001039089	sel-1 suppressor of lin-12-like (C. elegans) (Sel1l), transcript variant 1, mRNA. (I)	Sel1l	sel-1 suppressor of lin-12-like (C. elegans)	SEL1L	2.26E-02	1.906
ILMN_1259003	XM_913557	PREDICTED: similar to Ig heavy chain V region 3 precursor (LOC637785), mRNA. (S)	LOC637785	similar to Ig heavy chain V region 3 precursor	LOC637785	4.88E-03	1.914
ILMN_2705128	NM_139063	muted (Muted), mRNA. (S)	Muted	muted	MUTED	3.11E-02	1.914
ILMN_2699294	NM_026944	alkB, alkylation repair homolog 3 (E. coli) (Alkbh3), mRNA. XM_924276 (S)	Alkbh3	alkB, alkylation repair homolog 3 (E. coli)	Alkbh3	1.20E-03	1.916
ILMN_2879614	NM_021394	Z-DNA binding protein 1 (Zbp1), mRNA. (S)	Zbp1	Z-DNA binding protein 1	Zbp1	3.06E-03	1.924
ILMN_1236354	AK087474	NaN (S)	E130302P19Rik	cDNA sequence BC031353	BC031353	2.40E-03	1.925
ILMN_1222503	NM_025442	asparagine-linked glycosylation 5 homolog (yeast, dolichyl-phosphate beta-glucosyltransferase) (Alg5), mRNA. (S)	Alg5	asparagine-linked glycosylation 5 homolog (yeast, dolichyl-phosphate beta-glucosyltransferase)	Alg5	3.79E-04	1.925
ILMN_2623699	NM_013674	interferon regulatory factor 4 (Irf4), mRNA. (S)	Irf4	interferon regulatory factor 4	IRF4	1.06E-02	1.927
ILMN_1225932	AK077367	NaN (S)	Ccnd2	cyclin D2	CCND2	2.46E-02	1.930
ILMN_1242700	NM_029701	signal peptidase complex subunit 3 homolog (S. cerevisiae) (Spcs3), mRNA. (S)	Spcs3	signal peptidase complex subunit 3 homolog (S. cerevisiae)	SPCS3	9.49E-06	1.935
ILMN_2913716	NM_207105	histocompatibility 2, class II antigen A, beta 1 (H2-Ab1), mRNA. (S)	H2-Ab1	histocompatibility 2, class II antigen A, beta 1; response to metastatic cancers 2; similar to H-2 class II histocompatibility antigen, A-D beta chain precursor	Rmcs2	4.52E-03	1.950

ILMN_2499056	NaN	NaN (S)	IGKV2-137_AJ231263_Ig_kappa_variable_2-137_15	n/a	n/a	1.11E-02	1.952
ILMN_1257107	XM_001481017	PREDICTED: hypothetical protein LOC100043821 (LOC100043821), mRNA. (S)	LOC100043821	predicted gene 4671	Gm4671	7.96E-03	1.953
ILMN_2982316	NM_001005426	zinc finger, CW type with PWWP domain 1 (Zcwpw1), mRNA. (S)	Zcwpw1	paired immunoglobin-like type 2 receptor beta 2; zinc finger, CW type with PWWP domain 1	Pilrb2	5.27E-04	1.960
ILMN_2744660	XM_354710	NaN (S)	Igh-6	n/a	n/a	2.28E-04	1.961
ILMN_2744657	XM_354710	NaN (S)	Igh-6	n/a	n/a	6.26E-04	1.961
ILMN_1229245	NM_001024617	inositol polyphosphate-4-phosphatase, type II (Inpp4b), mRNA. (S)	Inpp4b	inositol polyphosphate-4-phosphatase, type II	INPP4B	1.18E-02	1.968
ILMN_1213332	XM_138309	NaN (S)	LOC380805	n/a	n/a	1.34E-02	1.969
ILMN_1259424	XM_147215	NaN (S)	Zfp288	n/a	n/a	4.64E-03	1.977
ILMN_1226525	NM_207105	histocompatibility 2, class II antigen A, beta 1 (H2-Ab1), mRNA. (S)	H2-Ab1	histocompatibility 2, class II antigen A, beta 1; response to metastatic cancers 2; similar to H-2 class II histocompatibility antigen, A-D beta chain precursor	Rmcs2	8.81E-03	1.978
ILMN_2822825	NM_176848	F-box protein 2 (Fbxo2), mRNA. (S)	Fbxo2	F-box protein 2	Fbxo2	1.94E-02	1.978
ILMN_2432262	NaN	NaN (S)	IGHV1S35_M12376_Ig_heavy_variable_1S35_13	n/a	n/a	4.46E-03	1.979
ILMN_2631093	NM_133655	CD 81 antigen (Cd81), mRNA. (S)	Cd81	CD81 antigen	CD81	8.24E-04	1.983
ILMN_2596970	NM_026407	transmembrane protein 39a (Tmem39a), mRNA. (S)	Tmem39a	transmembrane protein 39a	Tmem39a	2.22E-02	1.984
ILMN_2757807	NM_153584	cDNA sequence BC031353 (BC031353), mRNA. (S)	BC031353	cDNA sequence BC031353	BC031353	8.72E-03	1.985

ILMN_2813594	NM_183140	zinc finger protein 691 (Zfp691), mRNA. (S)	Zfp691	zinc finger protein 691	Zfp691	1.60E-03	1.993
ILMN_1244431	NM_001039089	sel-1 suppressor of lin-12-like (C. elegans) (Sel1l), transcript variant 1, mRNA. (S)	Sel1l	sel-1 suppressor of lin-12-like (C. elegans)	SEL1L	1.14E-03	1.996
ILMN_1238505	NM_010548	interleukin 10 (Il10), mRNA. (S)	Il10	interleukin 10	il10	1.13E-02	1.999
ILMN_2685769	XM_127132	NaN (S)	AW555464	n/a	n/a	1.13E-03	1.999
ILMN_1223179	NM_010398	histocompatibility 2, T region locus 23 (H2-T23), mRNA. (S)	H2-T23	histocompatibility 2, T region locus 23; similar to RT1 class Ib, locus H2-Q-like, grc region	LOC677644	2.17E-03	2.004
ILMN_1226138	NM_206924	jumping translocation breakpoint (Jtb), mRNA. (S)	Jtb	jumping translocation breakpoint	Jtb	9.65E-03	2.005
ILMN_1239921	NM_001029983	mannosidase, alpha, class 1B, member 1 (Man1b1), mRNA. (S)	Man1b1	mannosidase, alpha, class 1B, member 1	Man1b1	1.47E-03	2.009
ILMN_2624713	XM_915307	PREDICTED: microtubule associated serine/threonine kinase family member 4, transcript variant 6 (Mast4), mRNA. (S)	Mast4	microtubule associated serine/threonine kinase family member 4	MAST4	1.25E-03	2.011
ILMN_2998020	NM_026911	signal peptidase complex subunit 1 homolog (S. cerevisiae) (Spcs1), mRNA. (S)	Spcs1	signal peptidase complex subunit 1 homolog (S. cerevisiae)	SPCS1	1.97E-05	2.016
ILMN_1258929	NM_028476	RIKEN cDNA 2610110G12 gene (2610110G12Rik), mRNA. (S)	2610110G12Rik	RIKEN cDNA 2610110G12 gene	2610110G12Rik	8.82E-03	2.017
ILMN_1239448	NM_026968	mannosidase, beta A, lysosomal-like (Manbal), mRNA. (S)	Manbal	similar to Manbal protein; mannosidase, beta A, lysosomal-like	LOC100046016	4.40E-02	2.017
ILMN_1213431	XM_988307	PREDICTED: similar to Ig kappa chain V-III region PC 7132 (LOC626583), mRNA. (S)	LOC626583	immunoglobulin kappa chain variable 21 (V21)-2	Igk-V21-2	3.45E-04	2.019
ILMN_2734729	NM_010378	histocompatibility 2, class II antigen A, alpha (H2-Aa), mRNA. (S)	H2-Aa	histocompatibility 2, class II antigen A, alpha; histocompatibility 2, class II antigen E alpha	H2-Ea	1.00E-02	2.024
ILMN_1242769	NM_017476	A kinase (PRKA) anchor protein 8-like (Akap8l), mRNA. (S)	Akap8l	A kinase (PRKA) anchor protein 8-like	akap8l	1.78E-02	2.024
ILMN_1225733	XR_031047	PREDICTED: similar to monoclonal antibody BBK-2 heavy chain (LOC677643), misc RNA. (S)	LOC677643	similar to Ig heavy chain V region 3 precursor; similar to Ig heavy chain V region 23 precursor	LOC640979	5.25E-03	2.025

ILMN_1255342	NM_011176	suppression of tumorigenicity 14 (colon carcinoma) (St14), mRNA. (S)	St14	suppression of tumorigenicity 14 (colon carcinoma)	ST14	7.21E-03	2.029
ILMN_2437019	NaN	NaN (S)	IGHV1S36_M13788_Ig_heavy_variable_1S36_40	n/a	n/a	4.07E-03	2.034
ILMN_2608133	NM_027897	rhophilin, Rho GTPase binding protein 2 (Rhpn2), mRNA. (S)	Rhpn2	rhophilin, Rho GTPase binding protein 2	rhpn2	2.06E-03	2.045
ILMN_1213809	NM_182806	G protein-coupled receptor 18 (Gpr18), mRNA. (S)	Gpr18	G protein-coupled receptor 18	gpr18	2.13E-02	2.048
ILMN_2715042	NM_011520	syndecan 3 (Sdc3), mRNA. (S)	Sdc3	syndecan 3	sdc3	1.55E-02	2.055
ILMN_1228328	NM_011136	POU domain, class 2, associating factor 1 (Pou2af1), mRNA. (S)	Pou2af1	POU domain, class 2, associating factor 1	POU2AF1	1.12E-03	2.055
ILMN_3089584	NM_010545	CD74 antigen (invariant polypeptide of major histocompatibility complex, class II antigen-associated) (Cd74), transcript variant 2, mRNA. (A)	Cd74	CD74 antigen (invariant polypeptide of major histocompatibility complex, class II antigen-associated)	CD74	2.08E-04	2.055
ILMN_2716622	NM_011161	mitogen-activated protein kinase 11 (Mapk11), mRNA. (S)	Mapk11	mitogen-activated protein kinase 11	Mapk11	7.95E-05	2.059
ILMN_3041149	NM_008288	hydroxysteroid 11-beta dehydrogenase 1 (Hsd11b1), transcript variant 1, mRNA. (I)	Hsd11b1	hydroxysteroid 11-beta dehydrogenase 1	HSD11B1	1.55E-02	2.064
ILMN_2648669	NM_053110	glycoprotein (transmembrane) nmb (Gpnmb), mRNA. (S)	Gpnmb	glycoprotein (transmembrane) nmb	GPNMB	2.44E-02	2.064
ILMN_1255385	NM_023850	carbohydrate (keratan sulfate Gal-6) sulfotransferase 1 (Chst1), mRNA. (S)	Chst1	carbohydrate (keratan sulfate Gal-6) sulfotransferase 1	CHST1	3.98E-04	2.065
ILMN_2627300	NM_194462	A kinase (PRKA) anchor protein (yotiao) 9 (Akap9), mRNA. (S)	Akap9	A kinase (PRKA) anchor protein (yotiao) 9	Akap9	4.94E-04	2.068
ILMN_2621519	XM_001002191	PREDICTED: similar to Ig heavy chain V region 145 precursor (LOC668549), mRNA. (S)	LOC668549	predicted gene 9235	Gm9235	2.36E-02	2.072

ILMN_1256335	NM_144850	Rap guanine nucleotide exchange factor (GEF) 3 (Rapgef3), mRNA. (S)	Rapgef3	Rap guanine nucleotide exchange factor (GEF) 3	Rapgef3	1.57E-03	2.075
ILMN_2700059	NM_009194	solute carrier family 12, member 2 (Slc12a2), mRNA. (S)	Slc12a2	similar to solute carrier family 12, member 2; solute carrier family 12, member 2	SLC12A2	1.37E-04	2.079
ILMN_1245300	AK089525	NaN (S)	F730045P10Rik	SLAM family member 7	SLAMF7	2.45E-03	2.083
ILMN_1229359	XR_034751	PREDICTED: similar to signal recognition particle receptor ('docking protein') (LOC638034), misc RNA. (S)	LOC638034	similar to signal recognition particle receptor (docking protein)	LOC638034	1.88E-04	2.087
ILMN_2597469	XM_914633	PREDICTED: similar to idiotypic anti-NP IgG(1) heavy chain V-D-J (LOC544904), mRNA. (S)	LOC544904	similar to Ig heavy chain V region 186-1 precursor	LOC544904	7.44E-03	2.096
ILMN_1213824	XM_001473420	PREDICTED: similar to SP140 nuclear body protein family member (LOC100039742), mRNA. (S)	LOC100039742	predicted gene 2397	Gm2397	3.83E-04	2.097
ILMN_2619428	XM_354718	NaN (S)	Igh-V11	n/a	n/a	9.11E-03	2.099
ILMN_3120510	NM_001039160	GTPase, very large interferon inducible 1 (Gvin1), transcript variant B, mRNA. (A)	Gvin1	predicted gene 1966; predicted gene 8995; GTPase, very large interferon inducible 1	GVIN1	1.17E-04	2.101
ILMN_1214090	NM_024439	histocompatibility 47 (H47), mRNA. (S)	H47	histocompatibility 47	H47	1.06E-03	2.106
ILMN_2575490	AK040902	NaN (S)	A530041M22Rik	n/a	n/a	2.33E-03	2.108
ILMN_1221817	NM_001042605	CD74 antigen (invariant polypeptide of major histocompatibility complex, class II antigen-associated) (Cd74), transcript variant 1, mRNA. (S)	Cd74	CD74 antigen (invariant polypeptide of major histocompatibility complex, class II antigen-associated)	CD74	3.87E-03	2.113
ILMN_1248860	XM_130951	NaN (S)	Dpm3	n/a	n/a	8.40E-03	2.134
ILMN_2767635	NM_029478	transmembrane protein 49 (Tmem49), mRNA. (S)	Tmem49	transmembrane protein 49	TMEM49	1.41E-05	2.135
ILMN_1249698	NM_016852	WW domain binding protein 2 (Wbp2), mRNA. (S)	Wbp2	WW domain binding protein 2	WBP2	3.50E-03	2.136

ILMN_2704257	NM_011261	reelin (Reln), mRNA. (S)	Reln	reelin	RELN	7.98E-03	2.140
ILMN_2697433	NM_172608	transmembrane protein 184b (Tmem184b), mRNA. (S)	Tmem184b	transmembrane protein 184b	TMEM184B	3.15E-04	2.148
ILMN_2674666	XM_987671	PREDICTED: similar to Ig kappa chain V-V region MPC11 precursor (LOC676136), mRNA. (S)	LOC676136	similar to Ig kappa chain V-V region MPC11 precursor	LOC676136	1.28E-02	2.157
ILMN_2627299	NM_194462	NaN (S)	Akap9	A kinase (PRKA) anchor protein (yotiao) 9	Akap9	2.39E-04	2.170
ILMN_2751471	NM_172510	major facilitator superfamily domain containing 4 (Mfsd4), mRNA. (S)	Mfsd4	major facilitator superfamily domain containing 4	Mfsd4	2.78E-04	2.170
ILMN_2561472	AK040738	NaN (S)	A530021P12Rik	tribbles homolog 1 (Drosophila)	TRIB1	1.40E-03	2.171
ILMN_2695539	NM_199308	NaN (S)	Mast3	microtubule associated serine/threonine kinase 3	MAST3	8.48E-03	2.172
ILMN_2689678	NM_028041	DEAD (Asp-Glu-Ala-Asp) box polypeptide 54 (Ddx54), mRNA. (S)	Ddx54	DEAD (Asp-Glu-Ala-Asp) box polypeptide 54	ddx54	3.74E-02	2.180
ILMN_2606313	XM_991772	PREDICTED: similar to Ig kappa chain V-V region L6 precursor (LOC635815), mRNA. (S)	LOC635815	n/a	n/a	3.76E-03	2.191
ILMN_2617468	NM_026929	ChaC, cation transport regulator-like 1 (E. coli) (Chac1), mRNA. (S)	Chac1	ChaC, cation transport regulator-like 1 (E. coli)	CHAC1	3.45E-02	2.197
ILMN_2699531	NM_026418	regulator of G-protein signalling 10 (Rgs10), mRNA. (S)	Rgs10	regulator of G-protein signalling 10	Rgs10	3.63E-04	2.203
ILMN_1244130	NM_173006	paraoxonase 3 (Pon3), mRNA. (S)	Pon3	paraoxonase 3	PON3	4.14E-04	2.204
ILMN_2593230	NM_010806	myeloid/lymphoid or mixed-lineage leukemia (trithorax homolog, Drosophila); translocated to, 4 (Mllt4), mRNA. (S)	Mllt4	myeloid/lymphoid or mixed-lineage leukemia (trithorax homolog, Drosophila); translocated to, 4	mllt4	6.61E-03	2.218
ILMN_2613601	NM_027222	RIKEN cDNA 2010001M09 gene (2010001M09Rik), mRNA. (S)	2010001M09Rik	RIKEN cDNA 2010001M09 gene	2010001M09Rik	5.81E-05	2.228
ILMN_2759309	NM_030098	ribonuclease, RNase A family, 6 (Rnase6), mRNA. (S)	Rnase6	ribonuclease, RNase A family, 6	RNASE6	4.03E-03	2.231

ILMN_2657980	NM_010173	fatty acid amide hydrolase (Faah), mRNA. (S)	Faah	fatty acid amide hydrolase	Faah	2.15E-03	2.232
ILMN_1220875	NM_008020	FK506 binding protein 2 (Fkbp2), mRNA. (S)	Fkbp2	FK506 binding protein 2	Fkbp2	2.28E-03	2.234
ILMN_3125890	NM_001078649	transmembrane protein 134 (Tmem134), transcript variant 1, mRNA. (A)	Tmem134	transmembrane protein 134	Tmem134	6.12E-03	2.238
ILMN_2593774	NM_025427	RIKEN cDNA 1190002H23 gene (1190002H23Rik), mRNA. (S)	1190002H23Rik	RIKEN cDNA 1190002H23 gene	1190002H23Rik	4.07E-03	2.241
ILMN_1239596	NaN	NaN (S)	IGKV4-91_AJ231229_Ig_kappa_variable_4-91_29	n/a	n/a	4.67E-03	2.242
ILMN_2588815	NM_018870	phosphoglycerate mutase 2 (Pgam2), mRNA. (S)	Pgam2	phosphoglycerate mutase 2	PGAM2	7.86E-05	2.244
ILMN_2757368	NM_029720	cysteine-rich with EGF-like domains 2 (Creld2), mRNA. (S)	Creld2	cysteine-rich with EGF-like domains 2	CRELD2	2.92E-04	2.249
ILMN_2760963	NM_177030	dedicator of cytokinesis 6 (Dock6), mRNA. (S)	Dock6	dedicator of cytokinesis 6	Dock6	4.29E-05	2.253
ILMN_2457614	NaN	NaN (S)	IGHV8S7_U23022_Ig_heavy_variable_8S7_163	n/a	n/a	2.42E-03	2.290
ILMN_2681824	NM_080428	F-box and WD-40 domain protein 7 (Fbxw7), mRNA. (S)	Fbxw7	F-box and WD-40 domain protein 7	FBXW7	2.52E-05	2.290
ILMN_2653735	NM_025745	NaN (S)	4933407N01Rik	RIKEN cDNA 4933407N01 gene	ERLEC1	3.29E-04	2.311
ILMN_2422416	NM_021349	NaN (S)	Tnfrsf13b	tumor necrosis factor receptor superfamily, member 13b	TNFRSF13B	2.05E-03	2.341
ILMN_2633179	XR_033948	PREDICTED: similar to gamma-2a immunoglobulin heavy chain (LOC100047788), misc RNA. (S)	LOC100047788	similar to gamma-2a immunoglobulin heavy chain; immunoglobulin heavy chain 1a (serum IgG2a); immunoglobulin heavy chain 1b (serum IgG2c)	Igh-1b	1.28E-03	2.345
ILMN_2589525	NM_198300	cytoplasmic polyadenylation element binding protein 3 (Cpeb3), mRNA. (S)	Cpeb3	cytoplasmic polyadenylation element binding protein 3	CPEB3	1.01E-03	2.348

ILMN_2829594	NM_010479	heat shock protein 1A (Hspa1a), mRNA. (S)	Hspa1a	heat shock protein 1B; heat shock protein 1A; heat shock protein 1-like	Hspa1l	2.05E-02	2.350
ILMN_2502860	NM_023913	NaN (S)	Ern1	endoplasmic reticulum (ER) to nucleus signalling 1	ERN1	1.57E-06	2.356
ILMN_2595359	NM_008577	solute carrier family 3 (activators of dibasic and neutral amino acid transport), member 2 (Slc3a2), mRNA. (S)	Slc3a2	solute carrier family 3 (activators of dibasic and neutral amino acid transport), member 2	SLC3A2	5.03E-05	2.386
ILMN_1250001	NM_026358	RIKEN cDNA 4930583H14 gene (4930583H14Rik), mRNA. (S)	4930583H14Rik	RIKEN cDNA 4930583H14 gene	4930583H14Rik	1.46E-03	2.397
ILMN_2663249	NM_029612	SLAM family member 9 (Slamf9), mRNA. (S)	Slamf9	SLAM family member 9	SLAMF9	1.42E-03	2.399
ILMN_2965669	NM_001081642	X-linked lymphocyte-regulated 4A (Xlr4a), mRNA. XM_985903 XM_985942 XM_985977 XM_988796 XM_988827 XM_988867 XM_988909 XM_988942 XM_988983 (S)	Xlr4a	X-linked lymphocyte-regulated 4A; hypothetical protein LOC100044048	Xlr4a	2.97E-04	2.410
ILMN_1227508	NM_023377	StAR-related lipid transfer (START) domain containing 5 (Stard5), mRNA. (S)	Stard5	StAR-related lipid transfer (START) domain containing 5	STARD5	2.85E-06	2.414
ILMN_1252437	NM_025745	NaN (S)	4933407N01Rik	RIKEN cDNA 4933407N01 gene	ERLEC1	4.55E-04	2.425
ILMN_1249670	NM_009787	protein disulfide isomerase associated 4 (Pdia4), mRNA. (S)	Pdia4	protein disulfide isomerase associated 4	Pdia4	4.35E-04	2.446
ILMN_2983948	NM_029720	cysteine-rich with EGF-like domains 2 (Creld2), mRNA. (S)	Creld2	cysteine-rich with EGF-like domains 2	CRELD2	1.18E-04	2.474
ILMN_2646985	NM_030693	activating transcription factor 5 (Atf5), mRNA. (S)	Atf5	activating transcription factor 5	ATF5	9.59E-03	2.514
ILMN_1239874	NM_138303	Yip1 domain family, member 2 (Yipf2), mRNA. (S)	Yipf2	Yip1 domain family, member 2	Yipf2	3.90E-02	2.570
ILMN_2909336	NM_153581	glycoprotein m6a (Gpm6a), mRNA. (S)	Gpm6a	glycoprotein m6a	gpm6a	9.43E-05	2.580
ILMN_2974611	NM_145391	TAP binding protein-like (Tapbpl), mRNA. (S)	Tapbpl	TAP binding protein-like	TAPBPL	1.54E-03	2.592

ILMN_1251011	NaN	NaN (S)	IGKV3-2_X16954_Ig_kappa_variable_3-2_18	n/a	n/a	2.62E-03	2.599
ILMN_1257525	NM_198300	cytoplasmic polyadenylation element binding protein 3 (Cpeb3), mRNA. (S)	Cpeb3	cytoplasmic polyadenylation element binding protein 3	CPEB3	1.52E-03	2.617
ILMN_2453303	NaN	NaN (S)	IGHV14S3_X03573$M12991X03573_Ig_heavy_variable_14S3_203	n/a	n/a	3.61E-04	2.646
ILMN_1218006	XM_001477466	PREDICTED: similar to monoclonal antibody 17-1A, light chain (LOC100047132), mRNA. (S)	LOC100047132	similar to monoclonal antibody 17-1A, light chain	LOC100047132	1.59E-03	2.655
ILMN_2707996	NM_133353	oocyte secreted protein 1 (Oosp1), mRNA. (S)	Oosp1	oocyte secreted protein 1	Oosp1	1.36E-03	2.707
ILMN_1244008	XM_988270	PREDICTED: similar to Ig kappa chain V-III region PC 7043 (LOC676222), mRNA. (S)	LOC676222	n/a	n/a	4.77E-03	2.800
ILMN_1256817	NM_011414	secretory leukocyte peptidase inhibitor (Slpi), mRNA. (S)	Slpi	secretory leukocyte peptidase inhibitor	Slpi	3.97E-04	2.897
ILMN_1240539	NM_008532	tumor-associated calcium signal transducer 1 (Tacstd1), mRNA. (S)	Tacstd1	epithelial cell adhesion molecule	epcam	3.21E-04	2.924
ILMN_2636229	NM_029627	lymphocyte antigen 6 complex, locus K (Ly6k), mRNA. (S)	Ly6k	lymphocyte antigen 6 complex, locus K	LY6K	1.59E-04	2.958
ILMN_1231284	XM_001477466	PREDICTED: similar to monoclonal antibody 17-1A, light chain (LOC100047132), mRNA. (S)	LOC100047132	similar to monoclonal antibody 17-1A, light chain	LOC100047132	7.93E-05	2.997
ILMN_2546455	AK005544	NaN (S)	Sel1h	sel-1 suppressor of lin-12-like (C. elegans)	SEL1L	2.20E-03	3.039
ILMN_1252541	XM_359089	NaN (S)	LOC386139	n/a	n/a	9.91E-04	3.070

ILMN_1258953	NM_172537	sema domain, transmembrane domain (TM), and cytoplasmic domain, (semaphorin) 6D (Sema6d), transcript variant 1, mRNA. (S)	Sema6d	sema domain, transmembrane domain (TM), and cytoplasmic domain, (semaphorin) 6D	SEMA6D	3.57E-06	3.447
ILMN_2743883	NM_198640	NaN (S)	AI324046	n/a	n/a	5.49E-03	3.450
ILMN_2523841	NM_198640	NaN (S)	AI324046	n/a	n/a	2.85E-03	3.581
ILMN_2489305	XM_992387	PREDICTED: hypothetical protein LOC207685, transcript variant 3 (LOC207685), mRNA. (S)	LOC207685	n/a	n/a	1.55E-03	3.593
ILMN_2743884	NM_198640	NaN (S)	AI324046	n/a	n/a	7.37E-03	3.754
ILMN_2596522	NM_013602	metallothionein 1 (Mt1), mRNA. (S)	Mt1	metallothionein 1	Mt1	6.32E-06	3.839
ILMN_1249975	XM_001001076	PREDICTED: Immunoglobulin heavy chain (gamma polypeptide), transcript variant 1 (Ighg), mRNA. (S)	Ighg	immunoglobulin heavy chain 3 (serum IgG2b); Immunoglobulin heavy chain (gamma polypeptide)	Igh-3	1.74E-04	3.887
ILMN_2703329	NM_198640	NaN (S)	AI324046	n/a	n/a	1.36E-03	4.163
ILMN_2690611	XM_988229	PREDICTED: similar to Ig kappa chain V-III region MOPC 63 precursor (LOC626347), mRNA. (S)	LOC626347	immunoglobulin kappa chain variable 21 (V21)-4	Igk-V21-4	8.28E-03	4.535
