# Supplementary material for: Age-related changes in the transcriptome of antibody-secreting cells
Source: Oncotarget. 2016 Mar 7;7(12):13340–53. doi: 10.18632/oncotarget.7958 (PMC4924646; doi:10.18632/oncotarget.7958)
Supplement: Supplementary file 3 [file oncotarget-07-13340-s003.pdf]

Supplemental Table 2: Significant Biological Functions from Ingenuity

Category	P-Value	Benjamini	# of Genes	Gene Lists
Cell Death and Survival	1.2E-14	6.65E-11	132	MYB (-2.979), EPB41 (-2.892), UHRF1 (-2.213), H2AFX (-2.115), BACH2 (-2.083), DEK (-2.065), EZH2 (-2.019), CBX5 (-2.011), NUP205 (-1.958), STMN1 (-1.883), RAN (-1.867), HAT1 (-1.866), ZEB2 (-1.864), GADD45A (-1.855), MCM7 (-1.855), SELL (-1.85), RRM2 (-1.803), Calm1 (includes others) (-1.781), RRM1 (-1.741), TOP2A (-1.729), RAD51 (-1.704), BIRC5 (-1.693), SLC25A4 (-1.69), LYZ (-1.689), SATB1 (-1.663), VAV3 (-1.646), CLSPN (-1.597), ACTB (-1.587), HNRNPK (-1.587), PTEN (-1.578), UNG (-1.577), MIF (-1.568), RBL1 (-1.565), DUSP6 (-1.565), CDK2 (-1.552), HSPE1 (-1.54), NCOA4 (-1.534), TENC1 (-1.527), DNMT1 (-1.5), ZBTB18 (-1.495), RAG1 (-1.486), CCT6A (-1.429), Cd24a (-1.425), CLK3 (1.415), NFE2L1 (1.416), AGA (1.433), NAGLU (1.439), BCL10 (1.44), SERP1 (1.456), NEK7 (1.459), BCL2 (1.467), MAGED1 (1.469), FPGS (1.492), CCDC47 (1.502), LAMP1 (1.507), PRTN3 (1.529), CD3E (1.531), SSPN (1.532), BAG1 (1.534), AMIGO2 (1.538), IRAK1 (1.538), ADK (1.546), GNPNAT1 (1.551), TMED10 (1.553), HIPK1 (1.567), PGRMC1 (1.568), CLCN3 (1.57), ITM2B (1.578), PLA2G16 (1.589), PSENEN (1.59), ZMYND11 (1.591), SDC1 (1.594), TOP1 (1.596), B2M (1.603), ITGAL (1.604), TMBIM4 (1.605), VCP (1.609), SPHK2 (1.613), CREB3L2 (1.613), ALCAM (1.615), GBA (1.626), XBP1 (1.643), DAD1 (1.651), GRK6 (1.662), ZFP36L1 (1.664), CTSS (1.666), HLA-A (1.666), VEGFA (1.685), JUN (1.687), P4HB (1.689), IL5RA (1.689), DNM2 (1.721), IGF2R (1.723), MVP (1.726), F2RL1 (1.728), APMAP (1.734), CST3 (1.734), PIGB (1.735), HTATIP2 (1.754), CACNA1H (1.765), HERPUD1 (1.769), TP53INP1 (1.77), SWAP70 (1.794), PRDX4 (1.846), CLIC4 (1.877), CCND2 (1.894), PRDM1 (1.896), MTDH (1.9), ALKBH3 (1.916), CD81 (1.983), HLA-E (2.004), ATAT1 (2.017), ST14 (2.029), POU2AF1 (2.055), MAPK11 (2.059), RAPGEF3 (2.075), SLAMF7 (2.083), RNF19B (2.108), CD74 (2.113), DPM3 (2.134), TRIB1 (2.171), RGS10 (2.203), MZB1 (2.228), FAAH (2.232), FBXW7 (2.29), TNFRSF13B (2.341), ERN1 (2.356), SLC3A2 (2.386), ATF5 (2.514), GPM6A (2.58), SLPI (2.897), Mt1 (3.839)

Cellular Growth and Proliferation	8E-11	1.3E-07	128	NEIL3 (-3.349), MYB (-2.979), UHRF1 (-2.213), H2AFX (-2.115), CDCA7 (-2.113), NASP (-2.106), BACH2 (-2.083), NAP1L1 (-2.033), EZH2 (-2.019), DPYSL2 (-1.916), STMN1 (-1.883), HAT1 (-1.866), ZEB2 (-1.864), IL18RAP (-1.856), GADD45A (-1.855), MCM7 (-1.855), SELL (-1.85), RRM2 (-1.803), SDCBP (-1.801), H2AFY (-1.767), MCM5 (-1.758), RRM1 (-1.741), H2AFZ (-1.738), TOP2A (-1.729), RAD51 (-1.704), BIRC5 (-1.693), SLC25A4 (-1.69), LYZ (-1.689), SATB1 (-1.663), VAV3 (-1.646), CLSPN (-1.597), LARP7 (-1.588), ACTB (-1.587), HNRNPK (-1.587), PTEN (-1.578), MIF (-1.568), RBL1 (-1.565), DUSP6 (-1.565), CDK2 (-1.552), NCOA4 (-1.534), TENC1 (-1.527), DNMT1 (-1.5), ZBTB18 (-1.495), RAG1 (-1.486), SF3A3 (-1.47), Cd24a (-1.425), HNRNPM (-1.401), NAB1 (1.411), TSPAN31 (1.415), AGA (1.433), BCL10 (1.44), COX17 (1.449), BCL2 (1.467), MAGED1 (1.469), CTSZ (1.481), PREB (1.488), FPGS (1.492), CD164 (1.525), PRTN3 (1.529), CD3E (1.531), BAG1 (1.534), IRAK1 (1.538), ADK (1.546), GNPNAT1 (1.551), SLC7A7 (1.558), FUT8 (1.564), HIPK1 (1.567), CLCN3 (1.57), PLA2G16 (1.589), ZMYND11 (1.591), SDC1 (1.594), TOP1 (1.596), B2M (1.603), ITGAL (1.604), SPHK2 (1.613), ALCAM (1.615), Serpina3g (includes others) (1.626), GLB1 (1.635), XBP1 (1.643), ZFP36L1 (1.664), CTSS (1.666), HLA-A (1.666), ENPP1 (1.676), VEGFA (1.685), JUN (1.687), IL5RA (1.689), DNM2 (1.721), IGF2R (1.723), MVP (1.726), F2RL1 (1.728), CST3 (1.734), RASGRP3 (1.735), HTATIP2 (1.754), ZBTB20 (1.767), Ly6a (includes others) (1.768), TP53INP1 (1.77), PSMB9 (1.792), SWAP70 (1.794), NDFIP1 (1.829), PRDX4 (1.846), TNS3 (1.889), CCND2 (1.894), PRDM1 (1.896), ALKBH3 (1.916), HLA-DQB1 (1.978), CD81 (1.983), JTB (2.005), POU2AF1 (2.055), MAPK11 (2.059), RAPGEF3 (2.075), SLC12A2 (2.079), SLAMF7 (2.083), CD74 (2.113), VMP1 (2.135), WBP2 (2.136), RELN (2.14), TRIB1 (2.171), RGCC (2.241), PGAM2 (2.244), FBXW7 (2.29), TNFRSF13B (2.341), SLC3A2 (2.386), ATF5 (2.514), GPM6A (2.58), SLPI (2.897), EPCAM (2.924), SEL1L (3.039), Mt1 (3.839)

Hematological System Development and Function	9.4E-11	1.3E-07	90	NEIL3 (-3.349), DNTT (-3.068), MYB (-2.979), H2AFX (-2.115), BACH2 (-2.083), DEK (-2.065), EZH2 (-2.019), CBX5 (-2.011), DPYSL2 (-1.916), IL18RAP (-1.856), GADD45A (-1.855), SELL (-1.85), HIST2H4B (-1.811), BIRC5 (-1.693), SATB1 (-1.663), VAV3 (-1.646), ACTB (-1.587), PTEN (-1.578), MIF (-1.568), RBL1 (-1.565), CDK2 (-1.552), DNMT1 (-1.5), RAG1 (-1.486), Cd24a (-1.425), NAB1 (1.411), NFE2L1 (1.416), NAGLU (1.439), BCL10 (1.44), BCL2 (1.467), CTSZ (1.481), MAGT1 (1.487), LAMP1 (1.507), PRTN3 (1.529), CD3E (1.531), IRAK1 (1.538), ADK (1.546), PON2 (1.555), FUT8 (1.564), SDC1 (1.594), B2M (1.603), ITGAL (1.604), SPHK2 (1.613), ENTPD7 (1.614), ALCAM (1.615), Serpina3g (includes others) (1.626), GBA (1.626), XBP1 (1.643), NARS (1.656), GRK6 (1.662), PPIB (1.663), ZFP36L1 (1.664), CTSS (1.666), HLA-A (1.666), ENPP1 (1.676), VEGFA (1.685), JUN (1.687), P4HB (1.689), IL5RA (1.689), IGF2R (1.723), MVP (1.726), F2RL1 (1.728), SLA (1.731), CST3 (1.734), RASGRP3 (1.735), Ly6a (includes others) (1.768), TP53INP1 (1.77), PSMB9 (1.792), SWAP70 (1.794), NDFIP1 (1.829), CLIC4 (1.877), CCND2 (1.894), PRDM1 (1.896), HLA-DQB1 (1.978), CD81 (1.983), HLA-E (2.004), ST14 (2.029), POU2AF1 (2.055), MAPK11 (2.059), CHST1 (2.065), RAPGEF3 (2.075), SLAMF7 (2.083), CD74 (2.113), TRIB1 (2.171), RGS10 (2.203), FBXW7 (2.29), TNFRSF13B (2.341), SLC3A2 (2.386), SLPI (2.897), Igl (3.593), Mt1 (3.839)
Tissue Morphology	9.4E-11	1.3E-07	87	NEIL3 (-3.349), MYB (-2.979), UHRF1 (-2.213), H2AFX (-2.115), BACH2 (-2.083), EZH2 (-2.019), STMN1 (-1.883), HAT1 (-1.866), ZEB2 (-1.864), GADD45A (-1.855), SELL (-1.85), H2AFZ (-1.738), RAD51 (-1.704), BIRC5 (-1.693), SATB1 (-1.663), VAV3 (-1.646), LARP7 (-1.588), PTEN (-1.578), UNG (-1.577), MIF (-1.568), RBL1 (-1.565), DUSP6 (-1.565), CDK2 (-1.552), DNMT1 (-1.5), ZBTB18 (-1.495), RAG1 (-1.486), Cd24a (-1.425), NFE2L1 (1.416), AGA (1.433), NAGLU (1.439), BCL10 (1.44), BCL2 (1.467), TMEM38B (1.47), PRTN3 (1.529), CD3E (1.531), IRAK1 (1.538), GNPNAT1 (1.551), CLCN3 (1.57), B2M (1.603), ITGAL (1.604), SPHK2 (1.613), CREB3L2 (1.613), ENTPD7 (1.614), ALCAM (1.615), GBA (1.626), GLB1 (1.635), XBP1 (1.643), DAD1 (1.651), GRK6 (1.662), ZFP36L1 (1.664), CTSS (1.666), HLA-A (1.666), VEGFA (1.685), JUN (1.687), IL5RA (1.689), IGF2R (1.723), F2RL1 (1.728), SLA (1.731), CST3 (1.734), RASGRP3 (1.735), BSCL2 (1.757), CACNA1H (1.765), Ly6a (includes others) (1.768), PSMB9 (1.792), SWAP70 (1.794), NDFIP1 (1.829), CLIC4 (1.877), CCND2 (1.894), PRDM1 (1.896), HLA-DQB1 (1.978), CD81 (1.983), ST14 (2.029), POU2AF1 (2.055), CHST1 (2.065), RAPGEF3 (2.075), SLC12A2 (2.079), CD74 (2.113), RELN (2.14), TRIB1 (2.171), RGS10 (2.203), PON3 (2.204), MLLT4 (2.218), FBXW7 (2.29), TNFRSF13B (2.341), ERN1 (2.356), Igl (3.593), Mt1 (3.839)

Cellular Development	7.2E-10	6.17E-07	115	NEIL3 (-3.349), DNTT (-3.068), MYB (-2.979), UHRF1 (-2.213), NASP (-2.106), BACH2 (-2.083), DEK (-2.065), NAP1L1 (-2.033), EZH2 (-2.019), CBX5 (-2.011), DPYSL2 (-1.916), STMN1 (-1.883), HAT1 (-1.866), ZEB2 (-1.864), GADD45A (-1.855), MCM7 (-1.855), SELL (-1.85), HIST2H4B (-1.811), RRM2 (-1.803), SDCBP (-1.801), H2AFY (-1.767), RRM1 (-1.741), H2AFZ (-1.738), RAD51 (-1.704), BIRC5 (-1.693), SATB1 (-1.663), VAV3 (-1.646), LARP7 (-1.588), ACTB (-1.587), HNRNPK (-1.587), PTEN (-1.578), MIF (-1.568), RBL1 (-1.565), DUSP6 (-1.565), CDK2 (-1.552), HSPE1 (-1.54), TENC1 (-1.527), DNMT1 (-1.5), ZBTB18 (-1.495), RAG1 (-1.486), Cd24a (-1.425), NAB1 (1.411), NFE2L1 (1.416), AGA (1.433), BCL10 (1.44), BCL2 (1.467), MAGED1 (1.469), CTSZ (1.481), CCDC47 (1.502), LAMP1 (1.507), CD164 (1.525), PRTN3 (1.529), CD3E (1.531), BAG1 (1.534), ADK (1.546), GNPNAT1 (1.551), FUT8 (1.564), CLCN3 (1.57), TOP1 (1.596), B2M (1.603), ITGAL (1.604), SPHK2 (1.613), CREB3L2 (1.613), ALCAM (1.615), Serpina3g (includes others) (1.626), GLB1 (1.635), XBP1 (1.643), LY6E (1.663), ZFP36L1 (1.664), CTSS (1.666), ENPP1 (1.676), VEGFA (1.685), JUN (1.687), IL5RA (1.689), IGF2R (1.723), MVP (1.726), F2RL1 (1.728), SLA (1.731), CST3 (1.734), RASGRP3 (1.735), HTATIP2 (1.754), BSCL2 (1.757), ZBTB20 (1.767), Ly6a (includes others) (1.768), TP53INP1 (1.77), SWAP70 (1.794), NDFIP1 (1.829), PRDX4 (1.846), CLIC4 (1.877), CCND2 (1.894), PRDM1 (1.896), ALKBH3 (1.916), ALG5 (1.925), HLA-DQB1 (1.978), CD81 (1.983), ST14 (2.029), POU2AF1 (2.055), MAPK11 (2.059), SLAMF7 (2.083), CD74 (2.113), VMP1 (2.135), WBP2 (2.136), RELN (2.14), TRIB1 (2.171), RGS10 (2.203), PGAM2 (2.244), FBXW7 (2.29), TNFRSF13B (2.341), SLC3A2 (2.386), ATF5 (2.514), GPM6A (2.58), SLPI (2.897), EPCAM (2.924), SEL1L (3.039), Igl (3.593)
**Humoral Immune Response**	3E-09	1.5E-06	44	DNTT (-3.068), MYB (-2.979), H2AFX (-2.115), BACH2 (-2.083), EZH2 (-2.019), GADD45A (-1.855), SELL (-1.85), VAV3 (-1.646), PTEN (-1.578), UNG (-1.577), MIF (-1.568), RAG1 (-1.486), Cd24a (-1.425), BCL10 (1.44), BCL2 (1.467), Ggta1 (1.514), CD3E (1.531), SDC1 (1.594), B2M (1.603), ITGAL (1.604), SPHK2 (1.613), XBP1 (1.643), CTSS (1.666), VEGFA (1.685), IL5RA (1.689), F2RL1 (1.728), SLA (1.731), RASGRP3 (1.735), Ly6a (includes others) (1.768), TP53INP1 (1.77), SWAP70 (1.794), CCND2 (1.894), PRDM1 (1.896), HLA-DQB1 (1.978), CD81 (1.983), POU2AF1 (2.055), CHST1 (2.065), SLAMF7 (2.083), CD74 (2.113), TNFRSF13B (2.341), ERN1 (2.356), SLPI (2.897), Igl (3.593), Mt1 (3.839)

Protein Synthesis	3E-09	1.5E-06	52	H2AFX (-2.115), BACH2 (-2.083), GADD45A (-1.855), H2AFZ (-1.738), HNRNPK (-1.587), PTEN (-1.578), UNG (-1.577), MIF (-1.568), RAG1 (-1.486), AMFR (1.404), BCL10 (1.44), SERP1 (1.456), CTSO (1.462), BCL2 (1.467), CTSZ (1.481), Ggta1 (1.514), EDEM1 (1.583), PSENEN (1.59), SDC1 (1.594), B2M (1.603), VCP (1.609), GBA (1.626), ERP44 (1.63), ZFP36L1 (1.664), CTSS (1.666), VEGFA (1.685), JUN (1.687), IL5RA (1.689), F2RL1 (1.728), CST3 (1.734), RASGRP3 (1.735), SERINC1 (1.742), Ly6a (includes others) (1.768), SWAP70 (1.794), LSR (1.807), NDFIP1 (1.829), HLA-DQB1 (1.978), CD81 (1.983), MAN1B1 (2.009), SPCS1 (2.016), ST14 (2.029), POU2AF1 (2.055), RAPGEF3 (2.075), VIMP (2.106), CD74 (2.113), DPM3 (2.134), RELN (2.14), TNFRSF13B (2.341), ERN1 (2.356), CPEB3 (2.617), Igl (3.593), Mt1 (3.839)
Cell Morphology	7E-09	2.41E-06	67	MYB (-2.979), EPB41 (-2.892), BACH2 (-2.083), DEK (-2.065), STMN1 (-1.883), RAN (-1.867), ZEB2 (-1.864), GADD45A (-1.855), RCC1 (-1.817), SLC25A4 (-1.69), VAV3 (-1.646), PTEN (-1.578), RBL1 (-1.565), CDK2 (-1.552), DNMT1 (-1.5), ZBTB18 (-1.495), RAG1 (-1.486), CDC5L (-1.437), Cd24a (-1.425), NAB1 (1.411), AGA (1.433), NAGLU (1.439), NEK7 (1.459), BCL2 (1.467), TMEM38B (1.47), CTSZ (1.481), LAMP1 (1.507), CD3E (1.531), TMED10 (1.553), PON2 (1.555), CLCN3 (1.57), B2M (1.603), Serpina3g (includes others) (1.626), GBA (1.626), GLB1 (1.635), XBP1 (1.643), PPIB (1.663), HLA-A (1.666), ENPP1 (1.676), VEGFA (1.685), JUN (1.687), IL5RA (1.689), DNM2 (1.721), SLA (1.731), BSCL2 (1.757), CACNA1H (1.765), Ly6a (includes others) (1.768), PSMB9 (1.792), CLIC4 (1.877), CCND2 (1.894), PRDM1 (1.896), HLA-DQB1 (1.978), CD81 (1.983), JTB (2.005), ATAT1 (2.017), ST14 (2.029), POU2AF1 (2.055), RAPGEF3 (2.075), SLC12A2 (2.079), TRIB1 (2.171), MLLT4 (2.218), FBXW7 (2.29), ERN1 (2.356), ATF5 (2.514), EPCAM (2.924), Igl (3.593), Mt1 (3.839)
Cellular Compromise	3.4E-08	1.11E-05	32	EPB41 (-2.892), VAV3 (-1.646), PTEN (-1.578), HSPE1 (-1.54), AMFR (1.404), NFE2L1 (1.416), AGA (1.433), NAGLU (1.439), BCL10 (1.44), SERP1 (1.456), BCL2 (1.467), CCDC47 (1.502), LAMP1 (1.507), SLC38A2 (1.513), IRAK1 (1.538), PON2 (1.555), B2M (1.603), ITGAL (1.604), VCP (1.609), ERP44 (1.63), XBP1 (1.643), HLA-A (1.666), SGIP1 (1.728), HERPUD1 (1.769), DERL3 (1.837), DNAJB9 (1.893), HLA-E (2.004), SLAMF7 (2.083), VIMP (2.106), CD74 (2.113), ERN1 (2.356), Mt1 (3.839)

Cellular Function and Maintenance	3.4E-08	1.11E-05	74	DNTT (-3.068), MYB (-2.979), H2AFX (-2.115), BACH2 (-2.083), CBX5 (-2.011), IL18RAP (-1.856), SELL (-1.85), PYGL (-1.738), BIRC5 (-1.693), SATB1 (-1.663), VAV3 (-1.646), PTEN (-1.578), CDK2 (-1.552), HSPE1 (-1.54), TENC1 (-1.527), RAG1 (-1.486), Cd24a (-1.425), AMFR (1.404), NAB1 (1.411), AGA (1.433), BCL10 (1.44), SERP1 (1.456), BCL2 (1.467), TMEM38B (1.47), CTSZ (1.481), MAGT1 (1.487), CCDC47 (1.502), LAMP1 (1.507), SLC38A2 (1.513), CD3E (1.531), IRAK1 (1.538), CLCN3 (1.57), B2M (1.603), ITGAL (1.604), VCP (1.609), SPHK2 (1.613), ERP44 (1.63), XBP1 (1.643), GRK6 (1.662), PPIB (1.663), CTSS (1.666), HLA-A (1.666), ENPP1 (1.676), VEGFA (1.685), JUN (1.687), IL5RA (1.689), IGF2R (1.723), F2RL1 (1.728), SLA (1.731), RASGRP3 (1.735), BSCL2 (1.757), HERPUD1 (1.769), NDFIP1 (1.829), DERL3 (1.837), CLIC4 (1.877), DNAJB9 (1.893), PRDM1 (1.896), HLA-DQB1 (1.978), CD81 (1.983), HLA-E (2.004), POU2AF1 (2.055), RAPGEF3 (2.075), SLC12A2 (2.079), VIMP (2.106), CD74 (2.113), VMP1 (2.135), RELN (2.14), MLLT4 (2.218), TNFRSF13B (2.341), ERN1 (2.356), SLC3A2 (2.386), SLPI (2.897), Igl (3.593), Mt1 (3.839)

Cancer	9.4E-08	0.000026	181	[illegible] 2.213), H2AFX (-2.115), CDCA7 (-2.113), BACH2 (-2.083), DEK (-2.065), NAP1L1 (-2.033), EZH2 (-2.019), CBX5 (-2.011), NUP205 (-1.958), DPYSL2 (-1.916), STMN1 (-1.883), RAN (-1.867), HAT1 (-1.866), ZEB2 (-1.864), GADD45A (-1.855), MCM7 (-1.855), SELL (-1.85), RCC1 (-1.817), RRM2 (-1.803), SDCBP (-1.801), H2AFY (-1.767), MCM5 (-1.758), HIST2H2AC (-1.753), RRM1 (-1.741), TOP2A (-1.729), UBAC1 (-1.723), RAD51 (-1.704), HIST1H2AJ (-1.702), DDX10 (-1.697), BIRC5 (-1.693), SLC25A4 (-1.69), LYZ (-1.689), ZNF367 (-1.666), SATB1 (-1.663), VAV3 (-1.646), CLSPN (-1.597), LARP7 (-1.588), ACTB (-1.587), HNRNPK (-1.587), OTUD4 (-1.581), PTEN (-1.578), NAT6 (-1.578), UNG (-1.577), MIF (-1.568), RBL1 (-1.565), DUSP6 (-1.565), CDK2 (-1.552), HSPE1 (-1.54), NCOA4 (-1.534), H3F3C (-1.51), DNMT1 (-1.5), RAG1 (-1.486), DDX39A (-1.468), MCM6 (-1.452), GSPT1 (1.41), NAB1 (1.411), FAM135A (1.412), NFE2L1 (1.416), SPCS2 (1.432), BCL10 (1.44), POU6F1 (1.441), COX17 (1.449), LUC7L2 (1.451), GUK1 (1.451), NEK7 (1.459), BCL2 (1.467), MAGED1 (1.469), CTSZ (1.481), FPGS (1.492), SPON1 (1.494), HM13 (1.5), CD2BP2 (1.506), LAMP1 (1.507), TMEM66 (1.508), NDUFV3 (1.511), Ggta1 (1.514), UBXN6 (1.52), RPN2 (1.521), CD3E (1.531), SSPN (1.532), BAG1 (1.534), AMIGO2 (1.538), IRAK1 (1.538), SP140 (1.542), ADK (1.546), PON2 (1.555), DDX26B (1.562), FUT8 (1.564), HIPK1 (1.567), B3GALNT1 (1.569), ARFGEF1 (1.569), CLCN3 (1.57), CCDC117 (1.571), RHOBTB1 (1.587), SDC1 (1.594), ERGIC1 (1.596), TOP1 (1.596), B2M (1.603), ITGAL (1.604), TMBIM4 (1.605), ATRAID (1.608), SPHK2 (1.613), CREB3L2 (1.613), ALCAM (1.615), GBA (1.626), BZW1 (1.632), XBP1 (1.643), LRP10 (1.659), GRK6 (1.662), ZFP36L1 (1.664), CTSS (1.666), HLA-A (1.666), LRRC8D (1.667), ENPP1 (1.676), VEGFA (1.685), JUN (1.687), P4HB (1.689), IL5RA (1.689), GOLGB1 (1.698), DNM2 (1.721), IGF2R (1.723), MVP (1.726), SGIP1 (1.728), F2RL1 (1.728), CST3 (1.734), HTATIP2 (1.754), BSCL2 (1.757), REXO2 (1.763), CACNA1H (1.765), ZBTB20 (1.767), TP53INP1 (1.77), KCNK6 (1.792), PSMB9 (1.792), SWAP70 (1.794), LSR (1.807), DNAJB9 (1.893), CCND2 (1.894), PRDM1 (1.896), MTDH (1.9), ALKBH3 (1.916), ZBP1 (1.924), FAM214A (1.985), ZNF691 (1.993), HLA-E (2.004), MAN1B1 (2.009), MAST4 (2.011), ATAT1 (2.017), ST14 (2.029), RHPN2 (2.045), MAPK11 (2.059), RAPGEF3 (2.075), SLC12A2 (2.079), SLAMF7

Hematological Disease	1.1E-07	0.000028	64	DNTT (-3.068), MYB (-2.979), H2AFX (-2.115), CDCA7 (-2.113), DEK (-2.065), EZH2 (-2.019), STMN1 (-1.883), HAT1 (-1.866), GADD45A (-1.855), SELL (-1.85), RCC1 (-1.817), RRM2 (-1.803), RRM1 (-1.741), TOP2A (-1.729), RAD51 (-1.704), DDX10 (-1.697), BIRC5 (-1.693), LYZ (-1.689), SATB1 (-1.663), PTEN (-1.578), UNG (-1.577), MIF (-1.568), CDK2 (-1.552), DNMT1 (-1.5), RAG1 (-1.486), TRAFD1 (1.416), BCL10 (1.44), LUC7L2 (1.451), BCL2 (1.467), MAGED1 (1.469), MAGT1 (1.487), FPGS (1.492), LAMP1 (1.507), CD3E (1.531), IRAK1 (1.538), SP140 (1.542), ADK (1.546), RHOBTB1 (1.587), SDC1 (1.594), TOP1 (1.596), B2M (1.603), ITGAL (1.604), SPHK2 (1.613), XBP1 (1.643), LRP10 (1.659), HLA-A (1.666), ENPP1 (1.676), VEGFA (1.685), JUN (1.687), DNM2 (1.721), F2RL1 (1.728), BSCL2 (1.757), ZBTB20 (1.767), PSMB9 (1.792), SWAP70 (1.794), CLIC4 (1.877), CCND2 (1.894), PRDM1 (1.896), CD74 (2.113), RELN (2.14), TRIB1 (2.171), FBXW7 (2.29), TNFRSF13B (2.341), CRELD2 (2.474)
Immunological Disease	1.1E-07	0.000028	92	DNTT (-3.068), H2AFX (-2.115), CDCA7 (-2.113), BACH2 (-2.083), DEK (-2.065), EZH2 (-2.019), DPYSL2 (-1.916), STMN1 (-1.883), HAT1 (-1.866), GADD45A (-1.855), SELL (-1.85), RCC1 (-1.817), RRM2 (-1.803), RRM1 (-1.741), H2AFZ (-1.738), TOP2A (-1.729), UBAC1 (-1.723), DDX10 (-1.697), BIRC5 (-1.693), LYZ (-1.689), SATB1 (-1.663), NUDCD2 (-1.617), LSM2 (-1.596), ACTB (-1.587), PTEN (-1.578), UNG (-1.577), MIF (-1.568), CDK2 (-1.552), DNMT1 (-1.5), RAG1 (-1.486), TRAFD1 (1.416), NAGLU (1.439), BCL10 (1.44), BCL2 (1.467), MAGED1 (1.469), CSN3 (1.469), CCSER2 (1.485), MAGT1 (1.487), FPGS (1.492), LAMP1 (1.507), PRTN3 (1.529), CD3E (1.531), IRAK1 (1.538), SP140 (1.542), HIPK1 (1.567), RHOBTB1 (1.587), ZMYND11 (1.591), SDC1 (1.594), TOP1 (1.596), B2M (1.603), ITGAL (1.604), VCP (1.609), SPHK2 (1.613), ENTPD7 (1.614), ALCAM (1.615), XBP1 (1.643), LRP10 (1.659), CTSS (1.666), HLA-A (1.666), ENPP1 (1.676), VEGFA (1.685), JUN (1.687), P4HB (1.689), DNM2 (1.721), F2RL1 (1.728), CST3 (1.734), PLD4 (1.75), BSCL2 (1.757), Ly6a (includes others) (1.768), PSMB9 (1.792), SWAP70 (1.794), C130026I21Rik (includes others) (1.89), CCND2 (1.894), PRDM1 (1.896), ZBP1 (1.924), HLA-DQB1 (1.978), CD81 (1.983), HLA-E (2.004), ATAT1 (2.017), MAPK11 (2.059), SLAMF7 (2.083), CD74 (2.113), RELN (2.14), MZB1 (2.228), RNASE6 (2.231), RGCC (2.241), FBXW7 (2.29), TNFRSF13B (2.341), SLC3A2 (2.386), CRELD2 (2.474), SEL1L (3.039), Mt1 (3.839)

Organismal Injury and Abnormalities	1.1E-07	0.000028	108	NEIL3 (-3.349), DNTT (-3.068), MYB (-2.979), UHRF1 (-2.213), H2AFX (-2.115), CDCA7 (-2.113), EZH2 (-2.019), STMN1 (-1.883), GADD45A (-1.855), MCM7 (-1.855), SELL (-1.85), RRM2 (-1.803), SDCBP (-1.801), H2AFY (-1.767), HIST2H2AC (-1.753), RRM1 (-1.741), TOP2A (-1.729), RAD51 (-1.704), HIST1H2AJ (-1.702), DDX10 (-1.697), BIRC5 (-1.693), SLC25A4 (-1.69), LYZ (-1.689), ZNF367 (-1.666), VAV3 (-1.646), OTUD4 (-1.581), PTEN (-1.578), NAT6 (-1.578), UNG (-1.577), MIF (-1.568), RBL1 (-1.565), DUSP6 (-1.565), CDK2 (-1.552), DNMT1 (-1.5), RAG1 (-1.486), MCM6 (-1.452), GSPT1 (1.41), TRAFD1 (1.416), NFE2L1 (1.416), AGA (1.433), NAGLU (1.439), BCL10 (1.44), GUK1 (1.451), NEK7 (1.459), BCL2 (1.467), FPGS (1.492), LAMP1 (1.507), CD3E (1.531), SSPN (1.532), BAG1 (1.534), IRAK1 (1.538), SP140 (1.542), ADK (1.546), ARFGEF1 (1.569), CLCN3 (1.57), ITM2B (1.578), RHOBTB1 (1.587), SDC1 (1.594), TOP1 (1.596), B2M (1.603), ITGAL (1.604), ATRAID (1.608), ALCAM (1.615), GBA (1.626), GLB1 (1.635), XBP1 (1.643), LRP10 (1.659), ZFP36L1 (1.664), CTSS (1.666), HLA-A (1.666), ENPP1 (1.676), VEGFA (1.685), JUN (1.687), P4HB (1.689), IL5RA (1.689), GOLGB1 (1.698), DNM2 (1.721), F2RL1 (1.728), CST3 (1.734), BSCL2 (1.757), REXO2 (1.763), ZBTB20 (1.767), TP53INP1 (1.77), KCNK6 (1.792), PSMB9 (1.792), SWAP70 (1.794), LSR (1.807), NDFIP1 (1.829), PRDX4 (1.846), CLIC4 (1.877), TNS3 (1.889), CCND2 (1.894), PRDM1 (1.896), MTDH (1.9), HLA-DQB1 (1.978), RHPN2 (2.045), CD74 (2.113), RELN (2.14), TRIB1 (2.171), RGS10 (2.203), PON3 (2.204), RGCC (2.241), FBXW7 (2.29), TNFRSF13B (2.341), CRELD2 (2.474), SLPI (2.897), EPCAM (2.924), Mt1 (3.839)

Organismal Survival	1.1E-07	0.000028	92	MYB (-2.979), UHRF1 (-2.213), H2AFX (-2.115), NASP (-2.106), EZH2 (-2.019), HAT1 (-1.866), ZEB2 (-1.864), GADD45A (-1.855), TOP2A (-1.729), RAD51 (-1.704), BIRC5 (-1.693), LYZ (-1.689), Hmgn2 (includes others) (-1.671), SATB1 (-1.663), VAV3 (-1.646), LARP7 (-1.588), HNRNPK (-1.587), ACTB (-1.587), PTEN (-1.578), UNG (-1.577), MIF (-1.568), RBL1 (-1.565), CDK2 (-1.552), HSPE1 (-1.54), DNMT1 (-1.5), ZBTB18 (-1.495), RAG1 (-1.486), NAB1 (1.411), NFE2L1 (1.416), TRAFD1 (1.416), AGA (1.433), NAGLU (1.439), BCL10 (1.44), COX17 (1.449), SERP1 (1.456), NEK7 (1.459), BCL2 (1.467), TMEM38B (1.47), CCDC47 (1.502), LAMP1 (1.507), CSAD (1.53), CD3E (1.531), BAG1 (1.534), IRAK1 (1.538), ADK (1.546), GNPNAT1 (1.551), TMED10 (1.553), FUT8 (1.564), HIPK1 (1.567), B3GALNT1 (1.569), CLCN3 (1.57), PSENEN (1.59), TOP1 (1.596), B2M (1.603), ITGAL (1.604), VCP (1.609), CREB3L2 (1.613), GBA (1.626), XBP1 (1.643), DAD1 (1.651), PPIB (1.663), LY6E (1.663), ZFP36L1 (1.664), VEGFA (1.685), JUN (1.687), IGF2R (1.723), F2RL1 (1.728), CST3 (1.734), HTATIP2 (1.754), BSCL2 (1.757), ZBTB20 (1.767), Ly6a (includes others) (1.768), TP53INP1 (1.77), LSR (1.807), UBA5 (1.828), CLIC4 (1.877), DNAJB9 (1.893), CCND2 (1.894), PRDM1 (1.896), ALKBH3 (1.916), ST14 (2.029), SLC12A2 (2.079), CD74 (2.113), RGS10 (2.203), MLLT4 (2.218), FBXW7 (2.29), TNFRSF13B (2.341), ERN1 (2.356), SLC3A2 (2.386), ATF5 (2.514), SLPI (2.897), Mt1 (3.839)
Hematopoiesis	3.2E-07	6.06E-05	48	DNTT (-3.068), MYB (-2.979), EPB41 (-2.892), BACH2 (-2.083), DEK (-2.065), EZH2 (-2.019), GADD45A (-1.855), BIRC5 (-1.693), SATB1 (-1.663), VAV3 (-1.646), PTEN (-1.578), RBL1 (-1.565), CDK2 (-1.552), RAG1 (-1.486), Cd24a (-1.425), NAB1 (1.411), BCL10 (1.44), BCL2 (1.467), LAMP1 (1.507), PRTN3 (1.529), CD3E (1.531), B2M (1.603), ITGAL (1.604), XBP1 (1.643), ENPP1 (1.676), VEGFA (1.685), JUN (1.687), IL5RA (1.689), IGF2R (1.723), SLA (1.731), CST3 (1.734), RASGRP3 (1.735), Ly6a (includes others) (1.768), TP53INP1 (1.77), NDFIP1 (1.829), CCND2 (1.894), PRDM1 (1.896), HLA-DQB1 (1.978), CD81 (1.983), ST14 (2.029), POU2AF1 (2.055), CD74 (2.113), TRIB1 (2.171), RGS10 (2.203), TNFRSF13B (2.341), ERN1 (2.356), SLC3A2 (2.386), Igl (3.593)
Lymphoid Tissue Structure and Development	3.2E-07	6.06E-05	44	DNTT (-3.068), MYB (-2.979), EZH2 (-2.019), IL18RAP (-1.856), SELL (-1.85), BIRC5 (-1.693), SATB1 (-1.663), VAV3 (-1.646), PTEN (-1.578), UNG (-1.577), MIF (-1.568), RBL1 (-1.565), CDK2 (-1.552), RAG1 (-1.486), Cd24a (-1.425), NAB1 (1.411), BCL10 (1.44), BCL2 (1.467), LAMP1 (1.507), CD3E (1.531), B2M (1.603), ITGAL (1.604), XBP1 (1.643), ENPP1 (1.676), VEGFA (1.685), JUN (1.687), IL5RA (1.689), IGF2R (1.723), SLA (1.731), RASGRP3 (1.735), BSCL2 (1.757), Ly6a (includes others) (1.768), NDFIP1 (1.829), CCND2 (1.894), PRDM1 (1.896), HLA-DQB1 (1.978), CD81 (1.983), POU2AF1 (2.055), CD74 (2.113), TRIB1 (2.171), TNFRSF13B (2.341), SLC3A2 (2.386), Igl (3.593), Mt1 (3.839)

Cellular Movement	4.7E-07	0.000087	79	MYB (-2.979), BACH2 (-2.083), DEK (-2.065), EZH2 (-2.019), CBX5 (-2.011), DPYSL2 (-1.916), STMN1 (-1.883), ZEB2 (-1.864), GADD45A (-1.855), SELL (-1.85), RRM2 (-1.803), SDCBP (-1.801), LYZ (-1.689), SATB1 (-1.663), VAV3 (-1.646), HNRNPK (-1.587), ACTB (-1.587), PTEN (-1.578), MIF (-1.568), NCOA4 (-1.534), TENC1 (-1.527), ZBTB18 (-1.495), RAG1 (-1.486), Cd24a (-1.425), BCL10 (1.44), BCL2 (1.467), CTSZ (1.481), PRTN3 (1.529), BAG1 (1.534), PON2 (1.555), FUT8 (1.564), ARFGEF1 (1.569), CLCN3 (1.57), SDC1 (1.594), ITGAL (1.604), TMBIM4 (1.605), VCP (1.609), SPHK2 (1.613), ALCAM (1.615), GBA (1.626), NARS (1.656), GRK6 (1.662), PPIB (1.663), CTSS (1.666), HLA-A (1.666), VEGFA (1.685), JUN (1.687), DNM2 (1.721), IGF2R (1.723), F2RL1 (1.728), HTATIP2 (1.754), TP53INP1 (1.77), SWAP70 (1.794), CLIC4 (1.877), TNS3 (1.889), PRDM1 (1.896), ALKBH3 (1.916), CD81 (1.983), ATAT1 (2.017), ST14 (2.029), POU2AF1 (2.055), MAPK11 (2.059), CHST1 (2.065), RAPGEF3 (2.075), SLC12A2 (2.079), CD74 (2.113), WBP2 (2.136), RELN (2.14), TRIB1 (2.171), MLLT4 (2.218), FAAH (2.232), RGCC (2.241), FBXW7 (2.29), SLC3A2 (2.386), GPM6A (2.58), SLPI (2.897), EPCAM (2.924), SEL1L (3.039), SEMA6D (3.447)
Cell-To-Cell Signaling and Interaction	1.7E-06	0.000226	38	IL18RAP (-1.856), GADD45A (-1.855), SELL (-1.85), SATB1 (-1.663), VAV3 (-1.646), PTEN (-1.578), MIF (-1.568), RAG1 (-1.486), Cd24a (-1.425), BCL10 (1.44), BCL2 (1.467), MAGT1 (1.487), PRTN3 (1.529), CD3E (1.531), IRAK1 (1.538), B2M (1.603), ITGAL (1.604), SPHK2 (1.613), ALCAM (1.615), HLA-A (1.666), CTSS (1.666), VEGFA (1.685), F2RL1 (1.728), CST3 (1.734), Ly6a (includes others) (1.768), PSMB9 (1.792), SWAP70 (1.794), NDFIP1 (1.829), CLIC4 (1.877), ZBP1 (1.924), HLA-DQB1 (1.978), CD81 (1.983), RAPGEF3 (2.075), CD74 (2.113), RGCC (2.241), TNFRSF13B (2.341), ERN1 (2.356), SLPI (2.897)
Immune Cell Trafficking	1.7E-06	0.000226	50	BACH2 (-2.083), DEK (-2.065), DPYSL2 (-1.916), IL18RAP (-1.856), GADD45A (-1.855), SELL (-1.85), LYZ (-1.689), SATB1 (-1.663), VAV3 (-1.646), ACTB (-1.587), PTEN (-1.578), MIF (-1.568), RAG1 (-1.486), Cd24a (-1.425), BCL10 (1.44), BCL2 (1.467), CTSZ (1.481), MAGT1 (1.487), PRTN3 (1.529), CD3E (1.531), IRAK1 (1.538), PON2 (1.555), FUT8 (1.564), SDC1 (1.594), B2M (1.603), ITGAL (1.604), SPHK2 (1.613), ALCAM (1.615), GBA (1.626), NARS (1.656), GRK6 (1.662), PPIB (1.663), CTSS (1.666), HLA-A (1.666), VEGFA (1.685), F2RL1 (1.728), CST3 (1.734), Ly6a (includes others) (1.768), PSMB9 (1.792), SWAP70 (1.794), NDFIP1 (1.829), PRDM1 (1.896), HLA-DQB1 (1.978), CD81 (1.983), POU2AF1 (2.055), CHST1 (2.065), RAPGEF3 (2.075), CD74 (2.113), TNFRSF13B (2.341), SLPI (2.897)

Inflammatory Response	1.7E-06	0.000226	74	BACH2 (-2.083), DEK (-2.065), DPYSL2 (-1.916), IL18RAP (-1.856), GADD45A (-1.855), SELL (-1.85), RRM2 (-1.803), RRM1 (-1.741), TOP2A (-1.729), BIRC5 (-1.693), LYZ (-1.689), SATB1 (-1.663), VAV3 (-1.646), ACTB (-1.587), PTEN (-1.578), MIF (-1.568), RBL1 (-1.565), RAG1 (-1.486), NFE2L1 (1.416), NAGLU (1.439), BCL10 (1.44), BCL2 (1.467), CTSZ (1.481), MAGT1 (1.487), FPGS (1.492), Ggta1 (1.514), PRTN3 (1.529), CD3E (1.531), IRAK1 (1.538), ADK (1.546), PON2 (1.555), PSENEN (1.59), SDC1 (1.594), B2M (1.603), ITGAL (1.604), VCP (1.609), SPHK2 (1.613), ENTPD7 (1.614), ALCAM (1.615), GBA (1.626), XBP1 (1.643), NARS (1.656), GRK6 (1.662), PPIB (1.663), CTSS (1.666), HLA-A (1.666), VEGFA (1.685), JUN (1.687), P4HB (1.689), IL5RA (1.689), NR1D2 (1.706), F2RL1 (1.728), CST3 (1.734), RASGRP3 (1.735), Ly6a (includes others) (1.768), TP53INP1 (1.77), PSMB9 (1.792), SWAP70 (1.794), NDFIP1 (1.829), CLIC4 (1.877), PRDM1 (1.896), ZBP1 (1.924), HLA-DQB1 (1.978), CD81 (1.983), HLA-E (2.004), POU2AF1 (2.055), MAPK11 (2.059), RAPGEF3 (2.075), CD74 (2.113), TRIB1 (2.171), FAAH (2.232), TNFRSF13B (2.341), SLPI (2.897), Mt1 (3.839)
Cell-mediated Immune Response	2.1E-06	0.000256	35	DNTT (-3.068), MYB (-2.979), BACH2 (-2.083), DEK (-2.065), DPYSL2 (-1.916), SELL (-1.85), BIRC5 (-1.693), SATB1 (-1.663), VAV3 (-1.646), ACTB (-1.587), PTEN (-1.578), CDK2 (-1.552), RAG1 (-1.486), NAB1 (1.411), BCL10 (1.44), BCL2 (1.467), LAMP1 (1.507), CD3E (1.531), B2M (1.603), ITGAL (1.604), GRK6 (1.662), HLA-A (1.666), VEGFA (1.685), IL5RA (1.689), IGF2R (1.723), F2RL1 (1.728), SLA (1.731), RASGRP3 (1.735), NDFIP1 (1.829), PRDM1 (1.896), HLA-DQB1 (1.978), CD81 (1.983), CD74 (2.113), SLC3A2 (2.386), Igl (3.593)
Embryonic Development	2.1E-06	0.000256	43	NEIL3 (-3.349), MYB (-2.979), UHRF1 (-2.213), H2AFX (-2.115), NASP (-2.106), EZH2 (-2.019), ZEB2 (-1.864), RRM1 (-1.741), RAD51 (-1.704), BIRC5 (-1.693), SATB1 (-1.663), LARP7 (-1.588), PTEN (-1.578), RBL1 (-1.565), DNMT1 (-1.5), ZBTB18 (-1.495), RAG1 (-1.486), Cd24a (-1.425), NAB1 (1.411), BCL10 (1.44), BCL2 (1.467), CD3E (1.531), GNPNAT1 (1.551), B2M (1.603), GLB1 (1.635), ZFP36L1 (1.664), CTSS (1.666), ENPP1 (1.676), VEGFA (1.685), JUN (1.687), F2RL1 (1.728), CST3 (1.734), RASGRP3 (1.735), ZBTB20 (1.767), PRDM1 (1.896), HLA-DQB1 (1.978), CD81 (1.983), POU2AF1 (2.055), CD74 (2.113), RELN (2.14), MLLT4 (2.218), FBXW7 (2.29), EPCAM (2.924)
Organ Development	2.1E-06	0.000256	24	MYB (-2.979), ZEB2 (-1.864), BIRC5 (-1.693), SATB1 (-1.663), PTEN (-1.578), ZBTB18 (-1.495), RAG1 (-1.486), NAB1 (1.411), BCL2 (1.467), CD3E (1.531), B2M (1.603), CTSS (1.666), ENPP1 (1.676), VEGFA (1.685), JUN (1.687), CST3 (1.734), RASGRP3 (1.735), ZBTB20 (1.767), PRDM1 (1.896), HLA-DQB1 (1.978), CD81 (1.983), POU2AF1 (2.055), CD74 (2.113), RELN (2.14)

Organismal Development	2.1E-06	0.000256	74	NEIL3 (-3.349), MYB (-2.979), UHRF1 (-2.213), NASP (-2.106), EZH2 (-2.019), HAT1 (-1.866), ZEB2 (-1.864), GADD45A (-1.855), SELL (-1.85), RAD51 (-1.704), BIRC5 (-1.693), SLC25A4 (-1.69), LYZ (-1.689), SATB1 (-1.663), VAV3 (-1.646), LARP7 (-1.588), PTEN (-1.578), UNG (-1.577), RBL1 (-1.565), CDK2 (-1.552), TENC1 (-1.527), DNMT1 (-1.5), ZBTB18 (-1.495), RAG1 (-1.486), Cd24a (-1.425), NAB1 (1.411), NFE2L1 (1.416), AGA (1.433), NAGLU (1.439), BCL10 (1.44), SERP1 (1.456), BCL2 (1.467), TMEM38B (1.47), CD3E (1.531), GNPNAT1 (1.551), FUT8 (1.564), CLCN3 (1.57), B2M (1.603), CREB3L2 (1.613), GLB1 (1.635), XBP1 (1.643), DAD1 (1.651), LY6E (1.663), ZFP36L1 (1.664), CTSS (1.666), ENPP1 (1.676), VEGFA (1.685), JUN (1.687), IL5RA (1.689), IGF2R (1.723), F2RL1 (1.728), SLA (1.731), CST3 (1.734), RASGRP3 (1.735), BSCL2 (1.757), CACNA1H (1.765), ZBTB20 (1.767), TP53INP1 (1.77), CLIC4 (1.877), DNAJB9 (1.893), CCND2 (1.894), PRDM1 (1.896), HLA-DQB1 (1.978), CD81 (1.983), POU2AF1 (2.055), RAPGEF3 (2.075), SLC12A2 (2.079), CD74 (2.113), RELN (2.14), TRIB1 (2.171), MLLT4 (2.218), FBXW7 (2.29), TNFRSF13B (2.341), Mt1 (3.839)
Tissue Development	2.1E-06	0.000256	54	MYB (-2.979), EZH2 (-2.019), HAT1 (-1.866), ZEB2 (-1.864), GADD45A (-1.855), SELL (-1.85), RRM1 (-1.741), BIRC5 (-1.693), SATB1 (-1.663), VAV3 (-1.646), LARP7 (-1.588), ACTB (-1.587), PTEN (-1.578), MIF (-1.568), RBL1 (-1.565), CDK2 (-1.552), DNMT1 (-1.5), ZBTB18 (-1.495), RAG1 (-1.486), NAB1 (1.411), BCL10 (1.44), BCL2 (1.467), CD3E (1.531), GNPNAT1 (1.551), FUT8 (1.564), B2M (1.603), ITGAL (1.604), SPHK2 (1.613), ALCAM (1.615), ZFP36L1 (1.664), CTSS (1.666), HLA-A (1.666), VEGFA (1.685), JUN (1.687), P4HB (1.689), CST3 (1.734), RASGRP3 (1.735), ZBTB20 (1.767), Ly6a (includes others) (1.768), PSMB9 (1.792), SWAP70 (1.794), PRDX4 (1.846), CLIC4 (1.877), CCND2 (1.894), PRDM1 (1.896), HLA-DQB1 (1.978), CD81 (1.983), POU2AF1 (2.055), CD74 (2.113), RELN (2.14), PGAM2 (2.244), FBXW7 (2.29), SLC3A2 (2.386), EPCAM (2.924)

Gastrointestinal Disease	2.3E-06	0.000273	147	NEIL3 (-3.349), MYB (-2.979), HIST2H2BF (-2.275), UHRF1 (-2.213), H2AFX (-2.115), CDCA7 (-2.113), BACH2 (-2.083), DEK (-2.065), NAP1L1 (-2.033), EZH2 (-2.019), CBX5 (-2.011), NUP205 (-1.958), DPYSL2 (-1.916), RAN (-1.867), HAT1 (-1.866), ZEB2 (-1.864), GADD45A (-1.855), SELL (-1.85), RRM2 (-1.803), H2AFY (-1.767), MCM5 (-1.758), RRM1 (-1.741), TOP2A (-1.729), UBAC1 (-1.723), RAD51 (-1.704), DDX10 (-1.697), BIRC5 (-1.693), SLC25A4 (-1.69), LYZ (-1.689), SATB1 (-1.663), VAV3 (-1.646), CLSPN (-1.597), LSM2 (-1.596), LARP7 (-1.588), PTEN (-1.578), UNG (-1.577), MIF (-1.568), RBL1 (-1.565), DUSP6 (-1.565), CDK2 (-1.552), HSPE1 (-1.54), NCOA4 (-1.534), H3F3C (-1.51), DNMT1 (-1.5), RAG1 (-1.486), DDX39A (-1.468), FAM135A (1.412), TRAFD1 (1.416), NFE2L1 (1.416), SPCS2 (1.432), BCL10 (1.44), POU6F1 (1.441), LUC7L2 (1.451), NEK7 (1.459), BCL2 (1.467), CSN3 (1.469), CTSZ (1.481), SPON1 (1.494), HM13 (1.5), CD2BP2 (1.506), TMEM66 (1.508), NDUFV3 (1.511), UBXN6 (1.52), RPN2 (1.521), CD3E (1.531), BAG1 (1.534), AMIGO2 (1.538), SP140 (1.542), ADK (1.546), PON2 (1.555), DDX26B (1.562), FUT8 (1.564), HIPK1 (1.567), B3GALNT1 (1.569), ARFGEF1 (1.569), CLCN3 (1.57), CCDC117 (1.571), RHOBTB1 (1.587), SDC1 (1.594), ERGIC1 (1.596), TOP1 (1.596), B2M (1.603), TMBIM4 (1.605), SPHK2 (1.613), CREB3L2 (1.613), ALCAM (1.615), BZW1 (1.632), XBP1 (1.643), CTSS (1.666), HLA-A (1.666), LRRC8D (1.667), ENPP1 (1.676), VEGFA (1.685), JUN (1.687), P4HB (1.689), IL5RA (1.689), GOLGB1 (1.698), IGF2R (1.723), MVP (1.726), SGIP1 (1.728), F2RL1 (1.728), CST3 (1.734), PLD4 (1.75), HTATIP2 (1.754), BSCL2 (1.757), CACNA1H (1.765), Ly6a (includes others) (1.768), TP53INP1 (1.77), PSMB9 (1.792), SWAP70 (1.794), LSR (1.807), C130026I21Rik (includes others) (1.89), DNAJB9 (1.893), CCND2 (1.894), PRDM1 (1.896), ZBP1 (1.924), HLA-DQB1 (1.978), FAM214A (1.985), ZNF691 (1.993), HLA-E (2.004), MAN1B1 (2.009), MAST4 (2.011), ATAT1 (2.017), ST14 (2.029), RHPN2 (2.045), MAPK11 (2.059), RAPGEF3 (2.075), SLC12A2 (2.079), SLAMF7 (2.083), CD74 (2.113), VMP1 (2.135), RELN (2.14), PON3 (2.204), MZB1 (2.228), RNASE6 (2.231), FBXW7 (2.29), TNFRSF13B (2.341), ERN1 (2.356), SLC3A2 (2.386), MGARP (2.397), SLAMF9 (2.399), CRELD2 (2.474), ATF5 (2.514), EPCAM (2.924), SEL1L (3.039), SEMA6D (3.447), Mt1 (3.839)
Respiratory Disease	2.3E-06	0.000273	40	EZH2 (-2.019), STMN1 (-1.883), GADD45A (-1.855), RCC1 (-1.817), RRM2 (-1.803), H2AFY (-1.767), HIST2H2AC (-1.753), RRM1 (-1.741), PYGL (-1.738), TOP2A (-1.729), BIRC5 (-1.693), PTEN (-1.578), DUSP6 (-1.565), RBL1 (-1.565), DNMT1 (-1.5), NAB1 (1.411), COX17 (1.449), BCL2 (1.467), MAGED1 (1.469), CTSZ (1.481), Ggta1 (1.514), CD164 (1.525), PRTN3 (1.529), CD3E (1.531), FUT8 (1.564), CLCN3 (1.57), TOP1 (1.596), B2M (1.603), ITGAL (1.604), ALCAM (1.615), XBP1 (1.643), GRK6 (1.662), HLA-A (1.666), VEGFA (1.685), JUN (1.687), HTATIP2 (1.754), PSMB9 (1.792), FBXW7 (2.29), SLPI (2.897), EPCAM (2.924)

Infectious Disease	3.4E-06	0.000373	72	NEIL3 (-3.349), DEK (-2.065), EZH2 (-2.019), NUP205 (-1.958), ZEB2 (-1.864), HIST1H2BN (-1.823), RRM2 (-1.803), RRM1 (-1.741), PYGL (-1.738), TOP2A (-1.729), DDX10 (-1.697), LYZ (-1.689), KIAA0922 (-1.6), HNRNPK (-1.587), PTEN (-1.578), MIF (-1.568), CDK2 (-1.552), RAG1 (-1.486), Cd24a (-1.425), FAM135A (1.412), TRAFD1 (1.416), SCFD1 (1.431), BCL10 (1.44), POU6F1 (1.441), BCL2 (1.467), CTSZ (1.481), MAGT1 (1.487), FPGS (1.492), GANAB (1.518), CD164 (1.525), CD3E (1.531), IRAK1 (1.538), ADK (1.546), HIPK1 (1.567), UBAC2 (1.567), EDEM1 (1.583), KBTBD11 (1.583), PSENEN (1.59), SDC1 (1.594), B2M (1.603), ITGAL (1.604), ATRAID (1.608), ENTPD7 (1.614), XBP1 (1.643), GRK6 (1.662), PPIB (1.663), LY6E (1.663), HLA-A (1.666), LRRC8D (1.667), JUN (1.687), IL5RA (1.689), DNM2 (1.721), IGF2R (1.723), F2RL1 (1.728), CST3 (1.734), LARS (1.766), ZBTB20 (1.767), PSMB9 (1.792), NDFIP1 (1.829), CLIC4 (1.877), ALKBH3 (1.916), SPCS3 (1.935), HLA-DQB1 (1.978), CD81 (1.983), HLA-E (2.004), SPCS1 (2.016), POU2AF1 (2.055), CHST1 (2.065), CD74 (2.113), ERN1 (2.356), SLPI (2.897), SEL1L (3.039)
DNA Replication, Recombination, and Repair	4.4E-06	0.000455	50	NEIL3 (-3.349), DNTT (-3.068), UHRF1 (-2.213), H2AFX (-2.115), NASP (-2.106), DEK (-2.065), NAP1L1 (-2.033), CBX5 (-2.011), STMN1 (-1.883), RAN (-1.867), HAT1 (-1.866), GADD45A (-1.855), MCM7 (-1.855), RRM2 (-1.803), RRM1 (-1.741), TOP2A (-1.729), RAD51 (-1.704), BIRC5 (-1.693), VAV3 (-1.646), CLSPN (-1.597), PTEN (-1.578), UNG (-1.577), MIF (-1.568), RBL1 (-1.565), CDK2 (-1.552), DNMT1 (-1.5), RAG1 (-1.486), MCM6 (-1.452), FAM135A (1.412), NFE2L1 (1.416), BCL2 (1.467), PRTN3 (1.529), HIPK1 (1.567), TOP1 (1.596), VCP (1.609), PPIB (1.663), CTSS (1.666), JUN (1.687), IGF2R (1.723), SLA (1.731), CST3 (1.734), REXO2 (1.763), ZBTB20 (1.767), SWAP70 (1.794), UBA5 (1.828), ALKBH3 (1.916), VMP1 (2.135), RELN (2.14), FBXW7 (2.29), Igl (3.593)
Cell Cycle	8.1E-06	0.000771	53	MYB (-2.979), UHRF1 (-2.213), H2AFX (-2.115), NASP (-2.106), EZH2 (-2.019), STMN1 (-1.883), RAN (-1.867), HAT1 (-1.866), ZEB2 (-1.864), GADD45A (-1.855), MCM7 (-1.855), RCC1 (-1.817), Calm1 (includes others) (-1.781), RRM1 (-1.741), TOP2A (-1.729), RAD51 (-1.704), BIRC5 (-1.693), VAV3 (-1.646), CLSPN (-1.597), HCFC1 (-1.594), PTEN (-1.578), MIF (-1.568), RBL1 (-1.565), CDK2 (-1.552), NCOA4 (-1.534), NUCKS1 (-1.514), DNMT1 (-1.5), RAG1 (-1.486), CDC5L (-1.437), GSPT1 (1.41), CLK3 (1.415), NEK7 (1.459), BCL2 (1.467), PLA2G16 (1.589), TOP1 (1.596), ITGAL (1.604), SPHK2 (1.613), XBP1 (1.643), VEGFA (1.685), JUN (1.687), SLA (1.731), Ly6a (includes others) (1.768), TP53INP1 (1.77), SWAP70 (1.794), CCND2 (1.894), MTDH (1.9), RELN (2.14), RGCC (2.241), PGAM2 (2.244), ERN1 (2.356), ATF5 (2.514), EPCAM (2.924), SEL1L (3.039)

Inflammatory Disease	8.1E-06	0.000771	66	DEK (-2.065), DPYSL2 (-1.916), STMN1 (-1.883), GADD45A (-1.855), SELL (-1.85), RRM2 (-1.803), MCM5 (-1.758), RRM1 (-1.741), TOP2A (-1.729), UBAC1 (-1.723), BIRC5 (-1.693), LYZ (-1.689), VAV3 (-1.646), NUDCD2 (-1.617), PTEN (-1.578), MIF (-1.568), CDK2 (-1.552), DNMT1 (-1.5), RAG1 (-1.486), NAGLU (1.439), BCL2 (1.467), CSN3 (1.469), CCSER2 (1.485), FPGS (1.492), SPON1 (1.494), PRTN3 (1.529), IRAK1 (1.538), ADK (1.546), PSENEN (1.59), ZMYND11 (1.591), SDC1 (1.594), B2M (1.603), ITGAL (1.604), VCP (1.609), ENTPD7 (1.614), ALCAM (1.615), XBP1 (1.643), LY6E (1.663), CTSS (1.666), HLA-A (1.666), VEGFA (1.685), JUN (1.687), P4HB (1.689), IL5RA (1.689), F2RL1 (1.728), CST3 (1.734), RASGRP3 (1.735), TP53INP1 (1.77), PSMB9 (1.792), PRDM1 (1.896), ZBP1 (1.924), HLA-DQB1 (1.978), CD81 (1.983), HLA-E (2.004), ATAT1 (2.017), MAPK11 (2.059), RAPGEF3 (2.075), SLAMF7 (2.083), CD74 (2.113), FAAH (2.232), RGCC (2.241), TNFRSF13B (2.341), ERN1 (2.356), SLPI (2.897), SEL1L (3.039), Mt1 (3.839)
Neurological Disease	8.1E-06	0.000771	69	EZH2 (-2.019), DPYSL2 (-1.916), STMN1 (-1.883), RAN (-1.867), ZEB2 (-1.864), GADD45A (-1.855), SELL (-1.85), RCC1 (-1.817), RRM2 (-1.803), RRM1 (-1.741), TOP2A (-1.729), UBAC1 (-1.723), VAV3 (-1.646), ACTB (-1.587), PTEN (-1.578), UNG (-1.577), MIF (-1.568), CDK2 (-1.552), DNMT1 (-1.5), RAG1 (-1.486), AGA (1.433), NAGLU (1.439), BCL2 (1.467), MAGED1 (1.469), CSN3 (1.469), SPON1 (1.494), SEC24A (1.499), LAMP1 (1.507), IRAK1 (1.538), TMED10 (1.553), PON2 (1.555), PGRMC1 (1.568), CLCN3 (1.57), ITM2B (1.578), SDC1 (1.594), TOP1 (1.596), B2M (1.603), ITGAL (1.604), VCP (1.609), ENTPD7 (1.614), ALCAM (1.615), GBA (1.626), GLB1 (1.635), XBP1 (1.643), LRP10 (1.659), LY6E (1.663), ZFP36L1 (1.664), CTSS (1.666), HLA-A (1.666), VEGFA (1.685), JUN (1.687), F2RL1 (1.728), CST3 (1.734), BSCL2 (1.757), HERPUD1 (1.769), PSMB9 (1.792), MTDH (1.9), HLA-DQB1 (1.978), HLA-E (2.004), MAPK11 (2.059), SLC12A2 (2.079), CD74 (2.113), RELN (2.14), MAST3 (2.172), RGS10 (2.203), FAAH (2.232), PGAM2 (2.244), FBXW7 (2.29), Mt1 (3.839)

Skeletal and Muscular Disorders	8.1E-06	0.000771	68	DEK (-2.065), STMN1 (-1.883), RAN (-1.867), GADD45A (-1.855), RRM2 (-1.803), MCM5 (-1.758), RRM1 (-1.741), TOP2A (-1.729), UBAC1 (-1.723), BIRC5 (-1.693), LYZ (-1.689), NUDCD2 (-1.617), ACTB (-1.587), PTEN (-1.578), MIF (-1.568), CDK2 (-1.552), DNMT1 (-1.5), RAG1 (-1.486), NAGLU (1.439), BCL2 (1.467), CSN3 (1.469), CCSER2 (1.485), FPGS (1.492), SEC24A (1.499), LAMP1 (1.507), PRTN3 (1.529), TMED10 (1.553), PGRMC1 (1.568), ZMYND11 (1.591), SDC1 (1.594), TOP1 (1.596), B2M (1.603), VCP (1.609), ALCAM (1.615), GBA (1.626), XBP1 (1.643), PPIB (1.663), LY6E (1.663), CTSS (1.666), HLA-A (1.666), ENPP1 (1.676), VEGFA (1.685), JUN (1.687), IL5RA (1.689), DNM2 (1.721), F2RL1 (1.728), CST3 (1.734), Ly6a (includes others) (1.768), HERPUD1 (1.769), PSMB9 (1.792), PRDM1 (1.896), MTDH (1.9), ZBP1 (1.924), HLA-DQB1 (1.978), CD81 (1.983), HLA-E (2.004), ATAT1 (2.017), MAPK11 (2.059), SLAMF7 (2.083), CD74 (2.113), MAST3 (2.172), RGS10 (2.203), RGCC (2.241), PGAM2 (2.244), FBXW7 (2.29), TNFRSF13B (2.341), ERN1 (2.356), SEL1L (3.039)
Hepatic System Disease	2.5E-05	0.00192	52	NEIL3 (-3.349), HIST2H2BF (-2.275), EZH2 (-2.019), RAN (-1.867), GADD45A (-1.855), SELL (-1.85), RRM2 (-1.803), H2AFY (-1.767), RRM1 (-1.741), TOP2A (-1.729), UBAC1 (-1.723), BIRC5 (-1.693), VAV3 (-1.646), PTEN (-1.578), MIF (-1.568), CDK2 (-1.552), H3F3C (-1.51), RAG1 (-1.486), NFE2L1 (1.416), LUC7L2 (1.451), BCL2 (1.467), CD2BP2 (1.506), TMEM66 (1.508), UBXN6 (1.52), BAG1 (1.534), B3GALNT1 (1.569), ARFGEF1 (1.569), RHOBTB1 (1.587), SPHK2 (1.613), CREB3L2 (1.613), BZW1 (1.632), XBP1 (1.643), VEGFA (1.685), JUN (1.687), IGF2R (1.723), HTATIP2 (1.754), BSCL2 (1.757), PSMB9 (1.792), LSR (1.807), PRDM1 (1.896), HLA-E (2.004), MAST4 (2.011), MAPK11 (2.059), RAPGEF3 (2.075), RELN (2.14), FBXW7 (2.29), ERN1 (2.356), CRELD2 (2.474), ATF5 (2.514), EPCAM (2.924), SEL1L (3.039), Mt1 (3.839)
Renal and Urological Disease	5.8E-05	0.00314	29	EZH2 (-2.019), STMN1 (-1.883), GADD45A (-1.855), RRM2 (-1.803), SDCBP (-1.801), H2AFY (-1.767), RRM1 (-1.741), TOP2A (-1.729), BIRC5 (-1.693), LYZ (-1.689), VAV3 (-1.646), OTUD4 (-1.581), PTEN (-1.578), RAG1 (-1.486), AGA (1.433), BCL2 (1.467), B2M (1.603), ITGAL (1.604), ZFP36L1 (1.664), HLA-A (1.666), VEGFA (1.685), JUN (1.687), IL5RA (1.689), F2RL1 (1.728), CCND2 (1.894), HLA-DQB1 (1.978), FBXW7 (2.29), TNFRSF13B (2.341), Mt1 (3.839)

Endocrine System Disorders	7.2E-05	0.00371	59	H2AFX (-2.115), BACH2 (-2.083), EZH2 (-2.019), GADD45A (-1.855), MCM7 (-1.855), SELL (-1.85), RRM2 (-1.803), H2AFY (-1.767), RRM1 (-1.741), TOP2A (-1.729), BIRC5 (-1.693), LYZ (-1.689), VAV3 (-1.646), LSM2 (-1.596), PTEN (-1.578), MIF (-1.568), RBL1 (-1.565), CDK2 (-1.552), NCOA4 (-1.534), DNMT1 (-1.5), RAG1 (-1.486), TRAFD1 (1.416), BCL10 (1.44), BCL2 (1.467), CSN3 (1.469), CD3E (1.531), HIPK1 (1.567), TOP1 (1.596), B2M (1.603), ALCAM (1.615), CTSS (1.666), HLA-A (1.666), VEGFA (1.685), JUN (1.687), P4HB (1.689), GOLGB1 (1.698), F2RL1 (1.728), CST3 (1.734), PLD4 (1.75), HTATIP2 (1.754), Ly6a (includes others) (1.768), TP53INP1 (1.77), KCNK6 (1.792), PSMB9 (1.792), C130026I21Rik (includes others) (1.89), CCND2 (1.894), ZBP1 (1.924), HLA-DQB1 (1.978), ATAT1 (2.017), RAPGEF3 (2.075), CD74 (2.113), RELN (2.14), MZB1 (2.228), RNASE6 (2.231), RGCC (2.241), FBXW7 (2.29), SLC3A2 (2.386), SLPI (2.897), EPCAM (2.924)
Skeletal and Muscular System Development and Function	0.00011	0.00493	9	BIRC5 (-1.693), DUSP6 (-1.565), CDK2 (-1.552), BCL2 (1.467), TMEM38B (1.47), CTSS (1.666), VEGFA (1.685), JUN (1.687), RELN (2.14)
**Metabolic Disease**	0.00011	0.00495	55	BACH2 (-2.083), DPYSL2 (-1.916), RAN (-1.867), ZEB2 (-1.864), SELL (-1.85), RCC1 (-1.817), LYZ (-1.689), LSM2 (-1.596), ACTB (-1.587), PTEN (-1.578), UNG (-1.577), MIF (-1.568), CDK2 (-1.552), TRAFD1 (1.416), NAGLU (1.439), BCL2 (1.467), CSN3 (1.469), SPON1 (1.494), CD3E (1.531), PON2 (1.555), HIPK1 (1.567), ITM2B (1.578), SDC1 (1.594), B2M (1.603), XBP1 (1.643), LRP10 (1.659), CTSS (1.666), HLA-A (1.666), PJA2 (1.666), ENPP1 (1.676), VEGFA (1.685), JUN (1.687), CST3 (1.734), PLD4 (1.75), BSCL2 (1.757), ZBTB20 (1.767), Ly6a (includes others) (1.768), PSMB9 (1.792), C130026I21Rik (includes others) (1.89), DNAJB9 (1.893), CCND2 (1.894), ZBP1 (1.924), HLA-DQB1 (1.978), ATAT1 (2.017), RAPGEF3 (2.075), CD74 (2.113), RELN (2.14), TRIB1 (2.171), MZB1 (2.228), RNASE6 (2.231), FAAH (2.232), ERN1 (2.356), SLC3A2 (2.386), SEL1L (3.039), Mt1 (3.839)
Organ Morphology	0.00011	0.005	33	GADD45A (-1.855), SELL (-1.85), SATB1 (-1.663), PTEN (-1.578), UNG (-1.577), RBL1 (-1.565), CDK2 (-1.552), RAG1 (-1.486), Cd24a (-1.425), AGA (1.433), NAGLU (1.439), BCL2 (1.467), LAMP1 (1.507), CD3E (1.531), XBP1 (1.643), LY6E (1.663), VEGFA (1.685), JUN (1.687), IL5RA (1.689), IGF2R (1.723), F2RL1 (1.728), SLA (1.731), BSCL2 (1.757), CCND2 (1.894), PRDM1 (1.896), HLA-DQB1 (1.978), ST14 (2.029), SLC12A2 (2.079), CD74 (2.113), TRIB1 (2.171), MZB1 (2.228), TNFRSF13B (2.341), Mt1 (3.839)

Renal and Urological System Development and Function	0.00011	0.005	6	GADD45A (-1.855), BCL2 (1.467), VEGFA (1.685), IGF2R (1.723), BSCL2 (1.757), Mt1 (3.839)
Cardiovascular System Development and Function	0.00015	0.0059	21	SELL (-1.85), BIRC5 (-1.693), VAV3 (-1.646), MIF (-1.568), CDK2 (-1.552), BCL2 (1.467), TMEM38B (1.47), ALCAM (1.615), GBA (1.626), XBP1 (1.643), LY6E (1.663), CTSS (1.666), VEGFA (1.685), JUN (1.687), IGF2R (1.723), F2RL1 (1.728), CST3 (1.734), CACNA1H (1.765), CCND2 (1.894), ALKBH3 (1.916), CD74 (2.113)
Reproductive System Disease	0.00015	0.0059	63	NEIL3 (-3.349), MYB (-2.979), UHRF1 (-2.213), H2AFX (-2.115), CDCA7 (-2.113), EZH2 (-2.019), GADD45A (-1.855), MCM7 (-1.855), SELL (-1.85), RRM2 (-1.803), H2AFY (-1.767), HIST2H2AC (-1.753), RRM1 (-1.741), TOP2A (-1.729), RAD51 (-1.704), HIST1H2AJ (-1.702), BIRC5 (-1.693), SLC25A4 (-1.69), ZNF367 (-1.666), VAV3 (-1.646), PTEN (-1.578), NAT6 (-1.578), MIF (-1.568), CDK2 (-1.552), DNMT1 (-1.5), MCM6 (-1.452), GSPT1 (1.41), BCL10 (1.44), GUK1 (1.451), NEK7 (1.459), BCL2 (1.467), SSPN (1.532), BAG1 (1.534), ADK (1.546), ARFGEF1 (1.569), SDC1 (1.594), TOP1 (1.596), B2M (1.603), ATRAID (1.608), ALCAM (1.615), GBA (1.626), CTSS (1.666), HLA-A (1.666), VEGFA (1.685), JUN (1.687), P4HB (1.689), GOLGB1 (1.698), REXO2 (1.763), ZBTB20 (1.767), KCNK6 (1.792), PSMB9 (1.792), LSR (1.807), CCND2 (1.894), PRDM1 (1.896), MTDH (1.9), RHPN2 (2.045), RELN (2.14), TRIB1 (2.171), RGCC (2.241), FBXW7 (2.29), TNFRSF13B (2.341), SLPI (2.897), EPCAM (2.924)
Tumor Morphology	0.00024	0.00807	19	GADD45A (-1.855), SELL (-1.85), BIRC5 (-1.693), PTEN (-1.578), MIF (-1.568), CDK2 (-1.552), BCL2 (1.467), BAG1 (1.534), HIPK1 (1.567), B2M (1.603), CTSS (1.666), VEGFA (1.685), JUN (1.687), CST3 (1.734), CD74 (2.113), WBP2 (2.136), TRIB1 (2.171), FBXW7 (2.29), TNFRSF13B (2.341)
Molecular Transport	0.00032	0.00932	27	EPB41 (-2.892), SELL (-1.85), LYZ (-1.689), VAV3 (-1.646), PTEN (-1.578), BCL2 (1.467), SLC38A2 (1.513), CD3E (1.531), B2M (1.603), SPHK2 (1.613), GBA (1.626), GLB1 (1.635), GRK6 (1.662), PPIB (1.663), HLA-A (1.666), F2RL1 (1.728), RASGRP3 (1.735), CACNA1H (1.765), ZBTB20 (1.767), HERPUD1 (1.769), SWAP70 (1.794), MTDH (1.9), RAPGEF3 (2.075), SLC12A2 (2.079), TRIB1 (2.171), SLC3A2 (2.386), Mt1 (3.839)

Dermatological Diseases and Conditions	0.00039	0.0108	45	H2AFX (-2.115), DPYSL2 (-1.916), RAN (-1.867), GADD45A (-1.855), SELL (-1.85), RRM2 (-1.803), H2AFY (-1.767), RRM1 (-1.741), H2AFZ (-1.738), TOP2A (-1.729), PTEN (-1.578), MIF (-1.568), RBL1 (-1.565), CDK2 (-1.552), HSPE1 (-1.54), RAG1 (-1.486), DDX39A (-1.468), NAB1 (1.411), BCL2 (1.467), FPGS (1.492), IRAK1 (1.538), PON2 (1.555), PLA2G16 (1.589), PSENEN (1.59), ELL2 (1.592), TOP1 (1.596), B2M (1.603), ITGAL (1.604), VCP (1.609), GBA (1.626), HLA-A (1.666), CTSS (1.666), VEGFA (1.685), JUN (1.687), P4HB (1.689), NR1D2 (1.706), F2RL1 (1.728), CST3 (1.734), CCND2 (1.894), PRDM1 (1.896), HLA-DQB1 (1.978), HLA-E (2.004), SLAMF7 (2.083), CD74 (2.113), SLPI (2.897)
Nucleic Acid Metabolism	0.00049	0.0122	3	RRM2 (-1.803), RRM1 (-1.741), ADK (1.546)
Small Molecule Biochemistry	0.00049	0.0122	11	RRM2 (-1.803), RRM1 (-1.741), PTEN (-1.578), SLC38A2 (1.513), ADK (1.546), PLA2G16 (1.589), GBA (1.626), GLB1 (1.635), MTDH (1.9), FAAH (2.232), SLC3A2 (2.386)
Endocrine System Development and Function	0.0006	0.014	6	PTEN (-1.578), CTSS (1.666), VEGFA (1.685), CST3 (1.734), CCND2 (1.894), MZB1 (2.228)
Connective Tissue Disorders	0.00065	0.0149	49	DEK (-2.065), RRM2 (-1.803), MCM5 (-1.758), RRM1 (-1.741), UBAC1 (-1.723), BIRC5 (-1.693), LYZ (-1.689), NUDCD2 (-1.617), PTEN (-1.578), MIF (-1.568), CDK2 (-1.552), DNMT1 (-1.5), RAG1 (-1.486), NAGLU (1.439), BCL2 (1.467), CCSER2 (1.485), FPGS (1.492), PRTN3 (1.529), ZMYND11 (1.591), SDC1 (1.594), B2M (1.603), VCP (1.609), GBA (1.626), PPIB (1.663), HLA-A (1.666), CTSS (1.666), ENPP1 (1.676), VEGFA (1.685), JUN (1.687), IL5RA (1.689), DNM2 (1.721), F2RL1 (1.728), CST3 (1.734), Ly6a (includes others) (1.768), PSMB9 (1.792), PRDM1 (1.896), ZBP1 (1.924), HLA-DQB1 (1.978), CD81 (1.983), HLA-E (2.004), ATAT1 (2.017), MAPK11 (2.059), SLAMF7 (2.083), CD74 (2.113), RGS10 (2.203), RGCC (2.241), TNFRSF13B (2.341), ERN1 (2.356), SEL1L (3.039)
Hair and Skin Development and Function	0.00066	0.0149	8	PTEN (-1.578), RBL1 (-1.565), AGA (1.433), NAGLU (1.439), LAMP1 (1.507), VEGFA (1.685), F2RL1 (1.728), ST14 (2.029)

Gene Expression	0.00079	0.0166	65	MYB (-2.979), UHRF1 (-2.213), BACH2 (-2.083), DEK (-2.065), NAP1L1 (-2.033), EZH2 (-2.019), CBX5 (-2.011), GADD45A (-1.855), MCM7 (-1.855), H2AFY (-1.767), MCM5 (-1.758), H2AFZ (-1.738), TOP2A (-1.729), BIRC5 (-1.693), ZNF367 (-1.666), SATB1 (-1.663), KIAA0922 (-1.6), HCFC1 (-1.594), PTEN (-1.578), MIF (-1.568), RBL1 (-1.565), CDK2 (-1.552), NCOA4 (-1.534), DNMT1 (-1.5), ZBTB18 (-1.495), CDC5L (-1.437), NAB1 (1.411), NFE2L1 (1.416), RNASEK (1.43), BCL10 (1.44), POU6F1 (1.441), BCL2 (1.467), MAGED1 (1.469), PREB (1.488), FPGS (1.492), CD3E (1.531), BAG1 (1.534), IRAK1 (1.538), SP140 (1.542), HIPK1 (1.567), ZMYND11 (1.591), ELL2 (1.592), TOP1 (1.596), SPHK2 (1.613), CREB3L2 (1.613), XBP1 (1.643), VEGFA (1.685), JUN (1.687), NR1D2 (1.706), IGF2R (1.723), DUSP26 (1.724), F2RL1 (1.728), SLA (1.731), HTATIP2 (1.754), ZBTB20 (1.767), CCND2 (1.894), PRDM1 (1.896), MTDH (1.9), POU2AF1 (2.055), MAPK11 (2.059), CHST1 (2.065), FBXW7 (2.29), ERN1 (2.356), ATF5 (2.514), EPCAM (2.924)
**Lipid Metabolism**	0.00081	0.0166	4	PLA2G16 (1.589), GBA (1.626), GLB1 (1.635), FAAH (2.232)
Developmental Disorder	0.00085	0.017	33	UHRF1 (-2.213), H2AFX (-2.115), EZH2 (-2.019), GADD45A (-1.855), RRM2 (-1.803), RRM1 (-1.741), BIRC5 (-1.693), SATB1 (-1.663), ACTB (-1.587), PTEN (-1.578), RBL1 (-1.565), CDK2 (-1.552), NFE2L1 (1.416), NEK7 (1.459), BCL2 (1.467), BAG1 (1.534), GNPNAT1 (1.551), FUT8 (1.564), HIPK1 (1.567), CLCN3 (1.57), VCP (1.609), XBP1 (1.643), DAD1 (1.651), ZFP36L1 (1.664), JUN (1.687), ZBTB20 (1.767), LSR (1.807), HLA-DQB1 (1.978), SLC12A2 (2.079), TRIB1 (2.171), RGS10 (2.203), FBXW7 (2.29), ERN1 (2.356)
Post-Translational Modification	0.00095	0.0183	32	EZH2 (-2.019), MCM7 (-1.855), SELL (-1.85), SDCBP (-1.801), MIF (-1.568), DUSP6 (-1.565), CDK2 (-1.552), Cd24a (-1.425), CLK3 (1.415), BCL10 (1.44), SERP1 (1.456), NEK7 (1.459), SDF2 (1.507), CMAS (1.507), RPN2 (1.521), CD3E (1.531), BAG1 (1.534), IRAK1 (1.538), ADK (1.546), FUT8 (1.564), HIPK1 (1.567), SPHK2 (1.613), GRK6 (1.662), VEGFA (1.685), F2RL1 (1.728), CCND2 (1.894), ALG5 (1.925), CD81 (1.983), MAPK11 (2.059), DPM3 (2.134), ERN1 (2.356), SLC3A2 (2.386)
Psychological Disorders	0.00112	0.0206	23	DPYSL2 (-1.916), RAN (-1.867), RCC1 (-1.817), ACTB (-1.587), PTEN (-1.578), UNG (-1.577), CDK2 (-1.552), DNMT1 (-1.5), BCL2 (1.467), SPON1 (1.494), PON2 (1.555), ITM2B (1.578), SDC1 (1.594), VCP (1.609), GBA (1.626), LRP10 (1.659), CTSS (1.666), VEGFA (1.685), JUN (1.687), CST3 (1.734), CD74 (2.113), RELN (2.14), FAAH (2.232)
Connective Tissue Development and Function	0.00138	0.0235	17	EZH2 (-2.019), HAT1 (-1.866), GADD45A (-1.855), ACTB (-1.587), PTEN (-1.578), MIF (-1.568), RBL1 (-1.565), CDK2 (-1.552), GNPNAT1 (1.551), JUN (1.687), BSCL2 (1.757), PRDX4 (1.846), CCND2 (1.894), RAPGEF3 (2.075), CD74 (2.113), TRIB1 (2.171), PGAM2 (2.244)

**Carbohydrate Metabolism**	0.00159	0.0246	22	PYGL (-1.738), MIF (-1.568), DUSP6 (-1.565), GUK1 (1.451), SERP1 (1.456), CMAS (1.507), GNPNAT1 (1.551), FUT8 (1.564), B3GALNT1 (1.569), GLB1 (1.635), XBP1 (1.643), HLA-A (1.666), ENPP1 (1.676), PIGB (1.735), SERINC1 (1.742), ZBTB20 (1.767), CD81 (1.983), MAN1B1 (2.009), CHST1 (2.065), VIMP (2.106), DPM3 (2.134), RGS10 (2.203)
Amino Acid Metabolism	0.0016	0.0246	4	PTEN (-1.578), SLC38A2 (1.513), MTDH (1.9), SLC3A2 (2.386)
Auditory and Vestibular System Development and Function	0.0016	0.0246	2	NAGLU (1.439), SLC12A2 (2.079)
Hereditary Disorder	0.0016	0.0246	10	RRM2 (-1.803), RRM1 (-1.741), TOP2A (-1.729), LYZ (-1.689), BCL2 (1.467), PRTN3 (1.529), B2M (1.603), XBP1 (1.643), JUN (1.687), RGS10 (2.203)
Nervous System Development and Function	0.0016	0.0246	21	NEIL3 (-3.349), ZEB2 (-1.864), LARP7 (-1.588), PTEN (-1.578), MIF (-1.568), DNMT1 (-1.5), ZBTB18 (-1.495), AGA (1.433), NAGLU (1.439), BCL10 (1.44), BCL2 (1.467), ZFP36L1 (1.664), VEGFA (1.685), JUN (1.687), CST3 (1.734), ZBTB20 (1.767), PRDM1 (1.896), RAPGEF3 (2.075), RELN (2.14), RGS10 (2.203), FBXW7 (2.29)
Reproductive System Development and Function	0.00176	0.0261	5	LARP7 (-1.588), PTEN (-1.578), CDK2 (-1.552), BCL2 (1.467), PRDM1 (1.896)
Cellular Response to Therapeutics	0.00222	0.0304	8	GADD45A (-1.855), RAD51 (-1.704), UNG (-1.577), RAG1 (-1.486), NFE2L1 (1.416), HIPK1 (1.567), TOP1 (1.596), SWAP70 (1.794)
Cardiovascular Disease	0.00224	0.0305	6	MIF (-1.568), RAG1 (-1.486), BCL2 (1.467), CTSS (1.666), VEGFA (1.685), PON3 (2.204)
Cell Signaling	0.00232	0.0311	16	EPB41 (-2.892), SELL (-1.85), LYZ (-1.689), VAV3 (-1.646), BCL2 (1.467), CD3E (1.531), SPHK2 (1.613), GRK6 (1.662), PPIB (1.663), F2RL1 (1.728), RASGRP3 (1.735), CACNA1H (1.765), HERPUD1 (1.769), SWAP70 (1.794), RAPGEF3 (2.075), SLC12A2 (2.079)
Vitamin and Mineral Metabolism	0.00232	0.0311	16	EPB41 (-2.892), SELL (-1.85), LYZ (-1.689), VAV3 (-1.646), BCL2 (1.467), CD3E (1.531), SPHK2 (1.613), GRK6 (1.662), PPIB (1.663), F2RL1 (1.728), RASGRP3 (1.735), CACNA1H (1.765), HERPUD1 (1.769), SWAP70 (1.794), RAPGEF3 (2.075), SLC12A2 (2.079)
Hypersensitivit y Response	0.00261	0.0326	7	GADD45A (-1.855), SELL (-1.85), MIF (-1.568), RAG1 (-1.486), ITGAL (1.604), IL5RA (1.689), TRIB1 (2.171)

Cellular Assembly and Organization	0.00264	0.0326	5	AGA (1.433), NAGLU (1.439), LAMP1 (1.507), VCP (1.609), GOLGB1 (1.698)
